# Supplementary material for: Cep120 is essential for kidney stromal progenitor cell growth and differentiation
Source: EMBO Rep. 2023 Dec 20;25(1):24. doi: 10.1038/s44319-023-00019-z (PMC10897188; doi:10.1038/s44319-023-00019-z)
Supplement: Supplementary file 1 — Appendix [file 44319_2023_19_MOESM1_ESM.pdf]

Appendix File Table of Contents

Appendix Figure S1 …………………………………………………………………… 2 - 3

Appendix Figure S1.

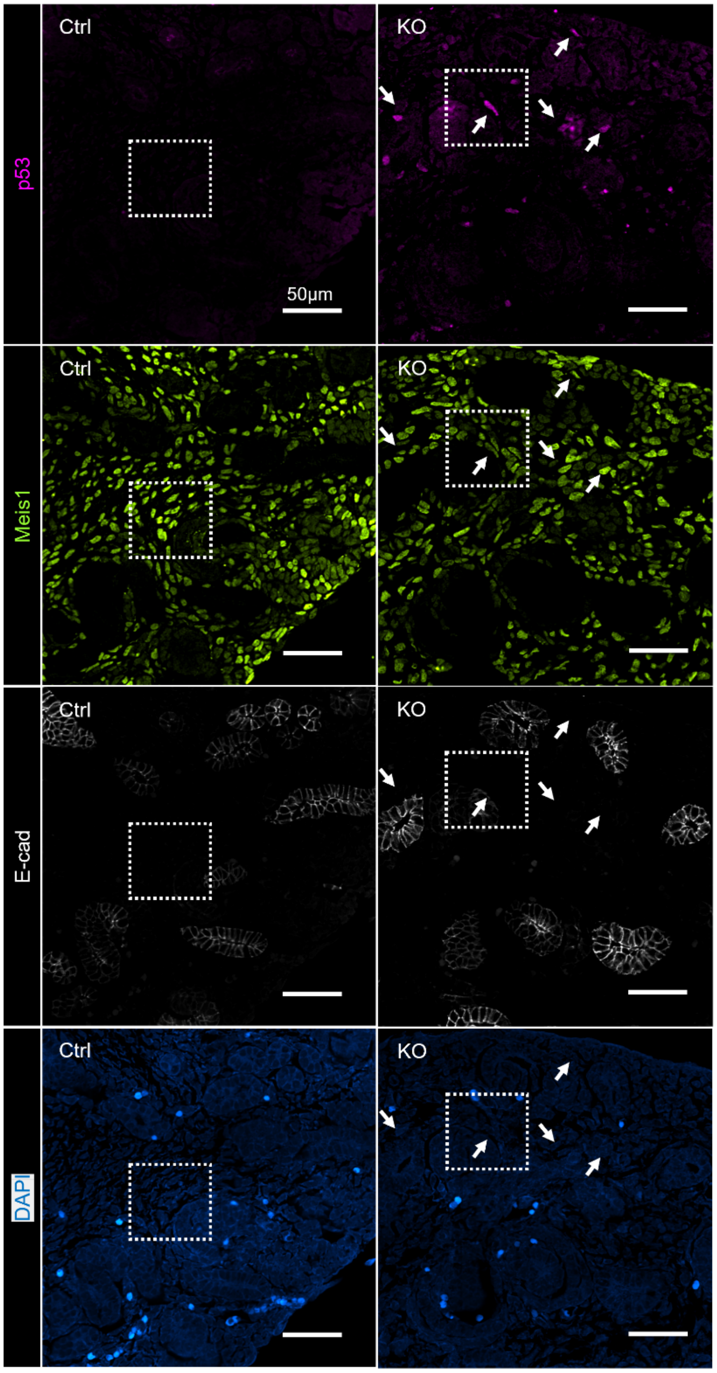

Appendix Figure S1. Immunofluorescence images of kidney sections from control and Cep120-KO mice at E15.5, stained with antibodies against p53, Meis1, E-cadherin and DAPI**.** This is supplementary material for Figure 4G, presented as single channels for each marker separately.
