# Supplementary material for: Cep120 is essential for kidney stromal progenitor cell growth and differentiation
Source: EMBO Rep. 2023 Dec 20;25(1):24. doi: 10.1038/s44319-023-00019-z (PMC10897188; doi:10.1038/s44319-023-00019-z)
Supplement: Supplementary file 3 — Table EV1 [file 44319_2023_19_MOESM3_ESM.docx]

**Table EV1.** Number of animals of each genotype isolated from the respective crosses (percentage of the total is indicated in parentheses).

|  | **Cep120F/F**  **+/+** | **Cep120F/+**  **+/+** | **Cep120F/+**  **FoxD1-Cre/+** | **Cep120F/F**  **FoxD1-Cre/+** | **Total** |
| --- | --- | --- | --- | --- | --- |
| **E15.5** | 9 (34.6%) | 7 (27%) | 5 (19%) | 5 (19%) | 26 |
| **P0** | 12 (25%) | 15 (31%) | 11 (23%) | 10 (21%) | 48 |
| **P15** | 5 (9%) | 15 (28%) | 17 (31.5%) | 17 (31.5%) | 54 |
| **P150** | 7 (20%) | 10 (28%) | 11 (31.5%) | 7 (20%) | 35 |
