# Supplementary material for: Cep120 is essential for kidney stromal progenitor cell growth and differentiation
Source: EMBO Rep. 2023 Dec 20;25(1):24. doi: 10.1038/s44319-023-00019-z (PMC10897188; doi:10.1038/s44319-023-00019-z)
Supplement: Supplementary file 4 — Table EV2 [file 44319_2023_19_MOESM4_ESM.docx]

**Table EV2.** List of primers used for genotyping.

| **Gene Name** | **Forward Primer 5' --> 3'** | **Reverse Primer 5' --> 3'** |
| --- | --- | --- |
| Cep120 WT | ATCACTGTGGAGCCTTGGGCA | TGTTACTCAGCAGCTGGTACC |
| Cep120 flox | CCTCTGCCTCCTTAGTGGATC | TCCATGAGTGAACGAACCTG |
| Cre | TCGATGCAACGAGTGATGAG | TCCATGAGTGAACGAACCTG |
