# Supplementary material for: Cep120 is essential for kidney stromal progenitor cell growth and differentiation
Source: EMBO Rep. 2023 Dec 20;25(1):24. doi: 10.1038/s44319-023-00019-z (PMC10897188; doi:10.1038/s44319-023-00019-z)
Supplement: Supplementary file 5 — Table EV3 [file 44319_2023_19_MOESM5_ESM.docx]

**Table EV3.** Detailed list of antibodies used in the study.

| **Antibodies (species)** | **Source** | **Cat. No.** |
| --- | --- | --- |
| Anti-Cep120 (Rat) | Betleja et al. 2018, eLife (doi.org/10.7554/eLife.35439 |  |
| Anti-Cep135 (Rabbit) | Abcam | ab75005 |
| Anti-Centrin (Mouse IgG2a) | Millipore | 04-1624 |
| Anti-Ninein (Rabbit) | James Sillibourne/Michel Bornens Lab ([doi.org/10.1242/jcs.02302](https://doi.org/10.1242/jcs.02302)) |  |
| Anti–γ-tubulin (GTU-88; Mouse IgG1) | Sigma-Aldrich | T6557 |
| Anti-Aldh1a2 (Rabbit) | Abcam | ab96060 |
| Anti-E-cadherin (Mouse IgG2a) | BD Bioscience | BDB610181 |
| Anti-PDGFR-β (Rabbit) | Cell Signaling | 3169 |
| Anti-Desmin (Mouse IgG1) | Agilent Technologies | M0760 |
| Anti-α-SMA (Mouse IgG2a) | Sigma | A2547 |
| Anti-Synaptopodin (Guinea Pig) | Synaptic Systems | 163004 |
| Anti-GATA3 (Mouse IgG1) | Bio Care Medical | CM405A |
| Anti-pHH3 (Mouse IgG1) | Cell Signaling | 9706 |
| Anti-Cleaved caspase 3 (Rabbit) | Cell Signaling | 9664S |
| Anti-Meis1 (Mouse IgG1) | ACTIVE MOTIF | 39795 |
| Anti-p53 (Rabbit) | Leica | NCL-L-p53-CM5p |
| Anti-WT1 (6F-H2; Mouse IgG1) | Dako | M 3561 |
| Anti–CLC-K (Rabbit) | Alomone Lab | ACL 004 |
| Anti-Aquaporin 2 (C-17; Goat) | Santa Cruz Biotechnology | SC-9882 |
| Anti-Fibronectin (Rabbit) | Abcam | ab2413 |
| Anti-Kim1 (Goat) | R&D Systems | AF1817-SP |
| Anti-Gli1 (Rat) | R&D Systems | MAB3324 |
| Anti-Gli2 (Goat) | R&D Systems | AF3635-SP |
| Anti-pSmad3 (Rabbit) | Rockland | 600-401-919S |
| Anti-GAPDH (Rabbit) | Abcam | ab181602 |
| LTL-FITC | Vector Laboratories | FL-1321 |
