# Supplementary material for: Cep120 is essential for kidney stromal progenitor cell growth and differentiation
Source: EMBO Rep. 2023 Dec 20;25(1):24. doi: 10.1038/s44319-023-00019-z (PMC10897188; doi:10.1038/s44319-023-00019-z)
Supplement: Supplementary file 6 — Table EV4 [file 44319_2023_19_MOESM6_ESM.docx]

**Table EV4.** qPCR primers list used in this study.

| **Gene Name** | **Forward Primer 5' --> 3'** | **Reverse Primer 5' --> 3'** |
| --- | --- | --- |
| Acta2 | AGC CAT CTT TCA TTG GGA TGG A | TAC CCC CTG ACA GGA CGT TG |
| Axin2 | AAAAATAAGCAGCCGTTCGC | CTTAAGTCAGCAGGGGCTCA |
| Col1a1 | CCTGACGCATGGCCAAGAAG | TACCTCGGGTTTCCACGTCT |
| Des | TGGAGCGTGACAACCTGATAG | TTCTCTGCTTCTTCTCTTAGTTGGA |
| FN1 | CCC TAT CTC TGA TAC CGT TGT CC | TGC CGC AAC TAC TGT GAT TCG G |
| Gli1 | TTGAGGTTGGGATGAAGAAGCAG | TCATTGGATTGAACATGGCGTC |
| Gli2 | GTTCCAAGGCCTACTCTCGCCTG | CTTGAGCAGTGGAGCACGGACAT |
| Ihh | CACGTGCATTGCTCTGTCAAG | ACACGCTCCCCGTTCTCTA |
| Havcr1 | ACAACAGCTGAGGTGACAGG | TTCCCTGGAGGGATTGCTTC |
| Lef1 | TGGTCAGCGCGAGACAATTA | AGCTGTCATTCTGGGACCTG |
| Meis1 | CGTGGCATCTTTCCCAAAGTA | CTGTTCTTCAGAAGGGTAAGGG |
| Pdgfrb | CTCCTTCAAGCTGCAGGTCA | CTCTGCAGGTAGACCAGGTG |
| Ptch1 | CGCTCTGGAGCAGATTTCCAA | TGAGGAGACCCACAACCAAA |
| Shh | CGGCAGATATGAAGGGAAGA | TCATCACAGAGATGGCCAAGG |
| Smad3 | GAAGAAGCTCAAGAAGACGGGG | CAGTAGATAACGTGAGGGAGCC |
| Smo | CAAGCTCGTGCTCTGGTCC | GGGCATGTAGACAGCACACA |
| Tgfb1 | TGG CGA GCC TTA GTT TGG A | TCG ACA TGG AGC TGG TGA AA |
| Wnt4 | GCCGGGCACTCATGAATCTT | ACGTCTTTACCTCGCAGGAG |
| Wnt7b | TTTGGCGTCCTCTACGTGAA | GCCAGGCCAGGAATCTTGTT |
| Wnt11 | ACCGCACTCGCAACTCC | GAGCGGAATCCTGTGTTCCC |
