# Supplementary figures and images for: Cep120 is essential for kidney stromal progenitor cell growth and differentiation (part 1 of 2)
Source: EMBO Rep. 2023 Dec 20;25(1):24. doi: 10.1038/s44319-023-00019-z (PMC10897188; doi:10.1038/s44319-023-00019-z)

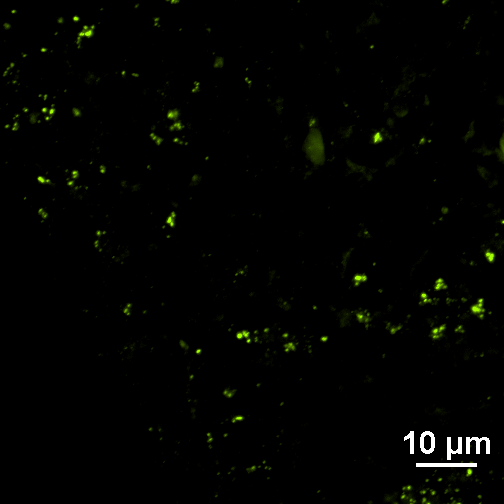

Supplement: Supplementary file 7 — Source Data Fig. 1 [file 44319_2023_19_MOESM7_ESM.zip › Fig.1/1G/Cep120-KO_Centrin_Meis1_Ninein_RGB_488-SD.tif]

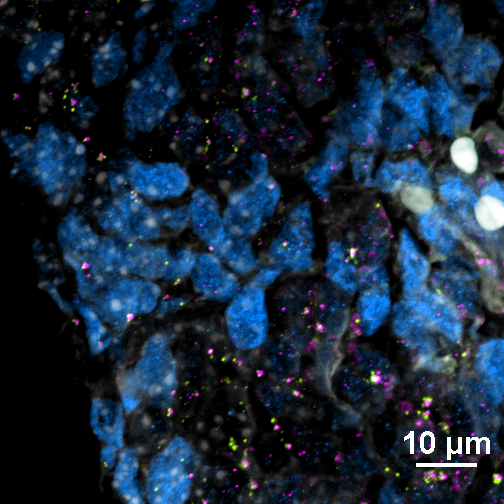

Supplement: Supplementary file 7 — Source Data Fig. 1 [file 44319_2023_19_MOESM7_ESM.zip › Fig.1/1G/Ctrl_Centrin_Meis1_Ninein.tif]

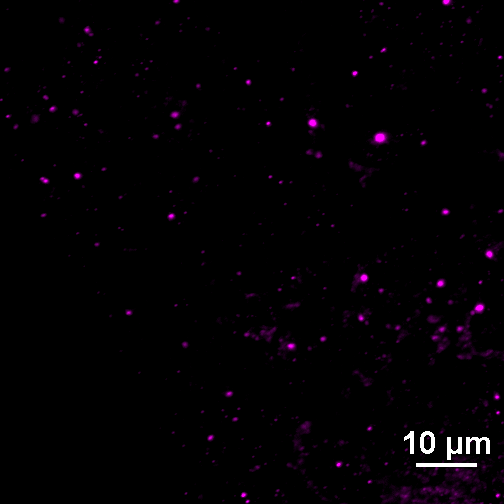

Supplement: Supplementary file 7 — Source Data Fig. 1 [file 44319_2023_19_MOESM7_ESM.zip › Fig.1/1G/Cep120-KO_Centrin_Meis1_Ninein_RGB_640-SD.tif]

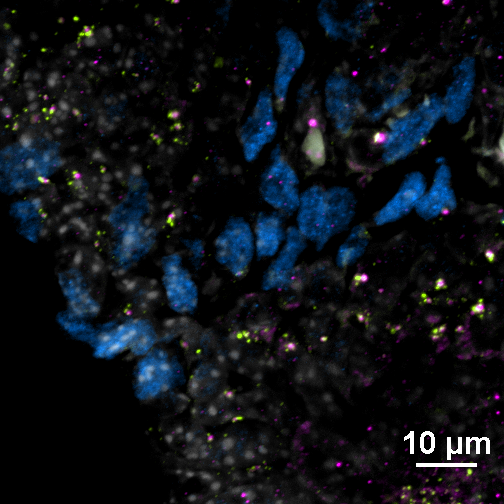

Supplement: Supplementary file 7 — Source Data Fig. 1 [file 44319_2023_19_MOESM7_ESM.zip › Fig.1/1G/Cep120-KO_Centrin_Meis1_Ninein.tif]

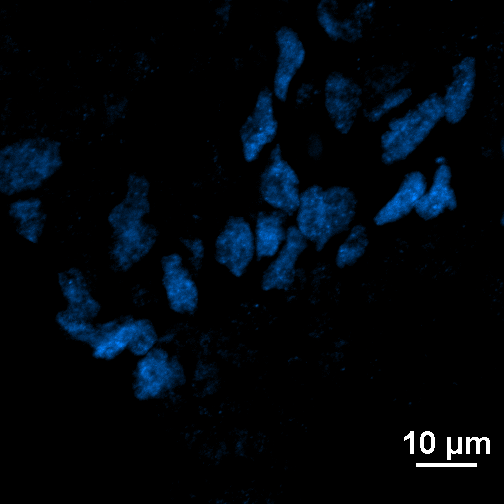

Supplement: Supplementary file 7 — Source Data Fig. 1 [file 44319_2023_19_MOESM7_ESM.zip › Fig.1/1G/Cep120-KO_Centrin_Meis1_Ninein_RGB_561-SD.tif]

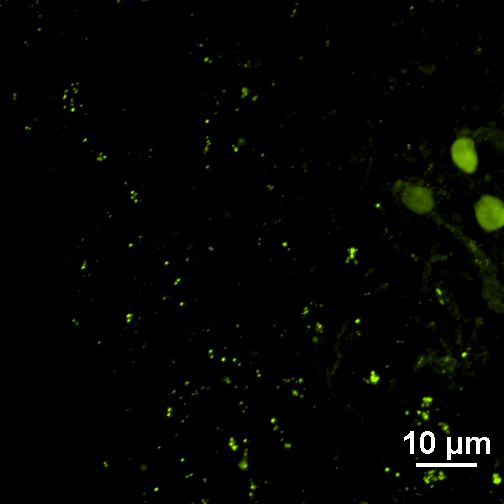

Supplement: Supplementary file 7 — Source Data Fig. 1 [file 44319_2023_19_MOESM7_ESM.zip › Fig.1/1G/Ctrl_Centrin_Meis1_Ninein_RGB_488-SD.tif]

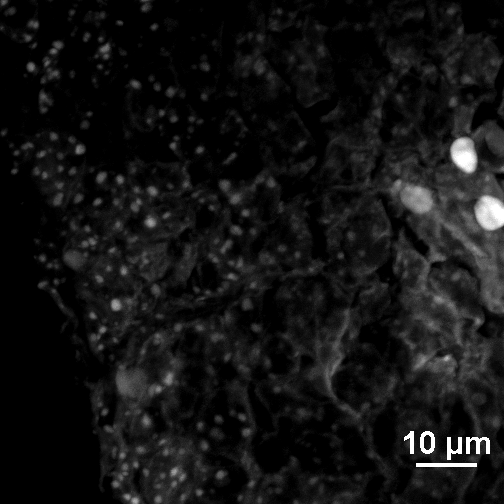

Supplement: Supplementary file 7 — Source Data Fig. 1 [file 44319_2023_19_MOESM7_ESM.zip › Fig.1/1G/Ctrl_Centrin_Meis1_Ninein_RGB_405-SD .tif]

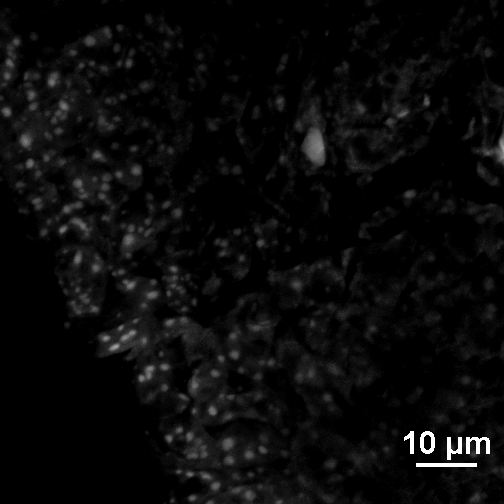

Supplement: Supplementary file 7 — Source Data Fig. 1 [file 44319_2023_19_MOESM7_ESM.zip › Fig.1/1G/Cep120-KO_Centrin_Meis1_Ninein_RGB_405-SD .tif]

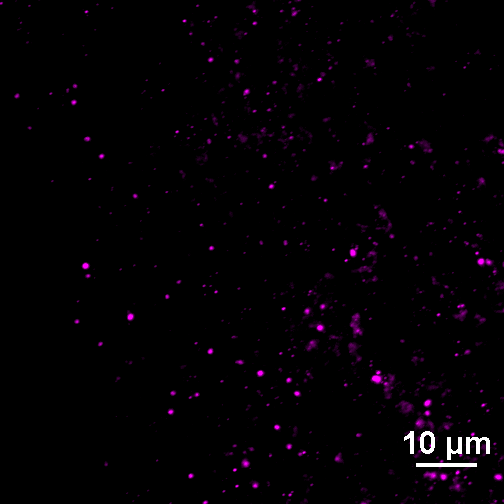

Supplement: Supplementary file 7 — Source Data Fig. 1 [file 44319_2023_19_MOESM7_ESM.zip › Fig.1/1G/Ctrl_Centrin_Meis1_Ninein_RGB_640-SD.tif]

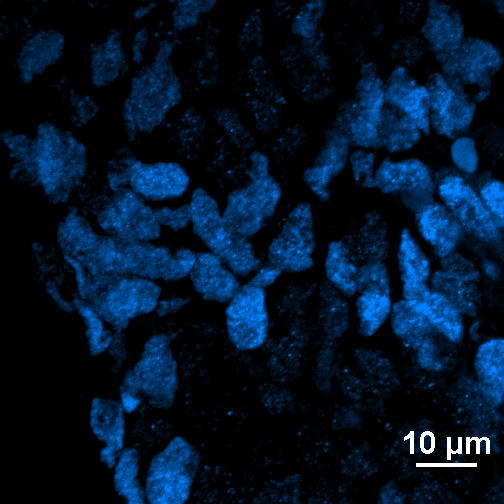

Supplement: Supplementary file 7 — Source Data Fig. 1 [file 44319_2023_19_MOESM7_ESM.zip › Fig.1/1G/Ctrl_Centrin_Meis1_Ninein_RGB_561-SD.tif]

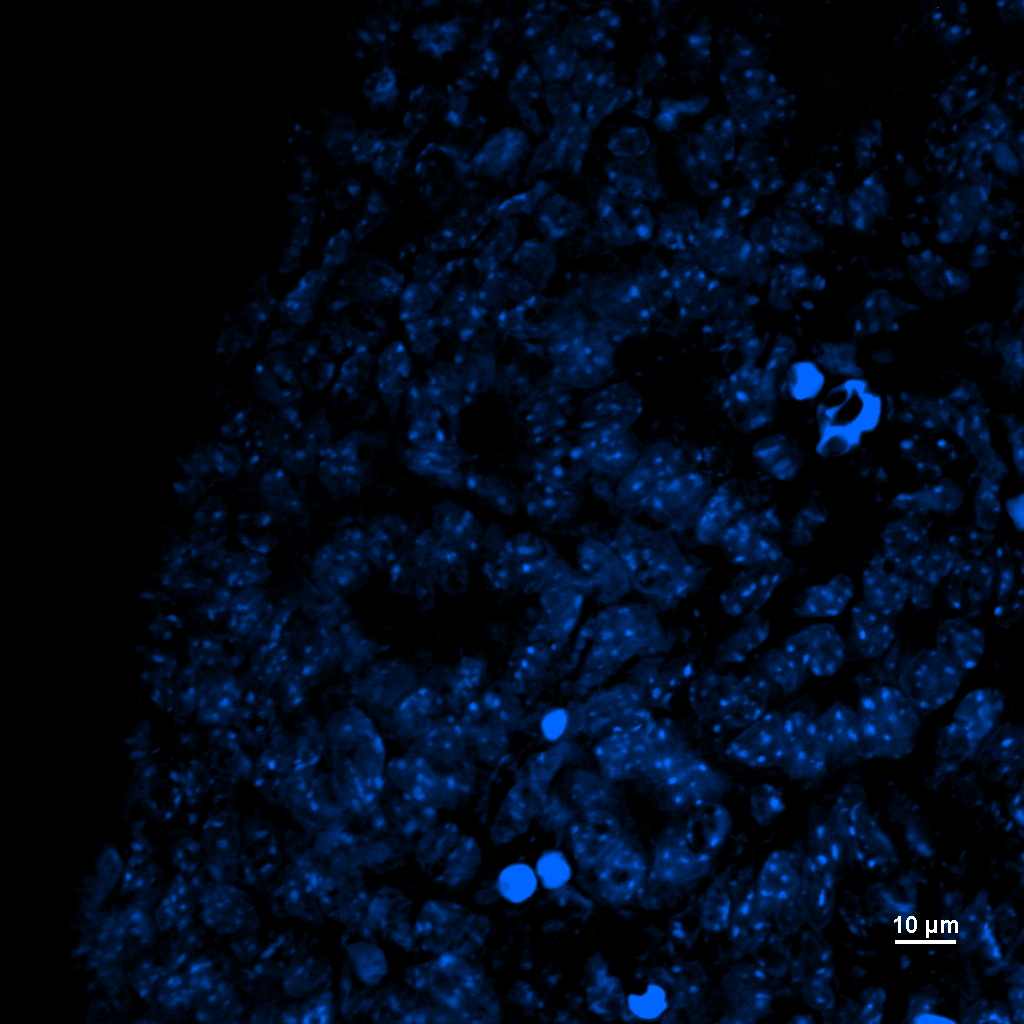

Supplement: Supplementary file 7 — Source Data Fig. 1 [file 44319_2023_19_MOESM7_ESM.zip › Fig.1/1E/Cep120-KO_Aldh1_g-tubulin_Ecad-MaxIP_RGB_405-SD .tif]

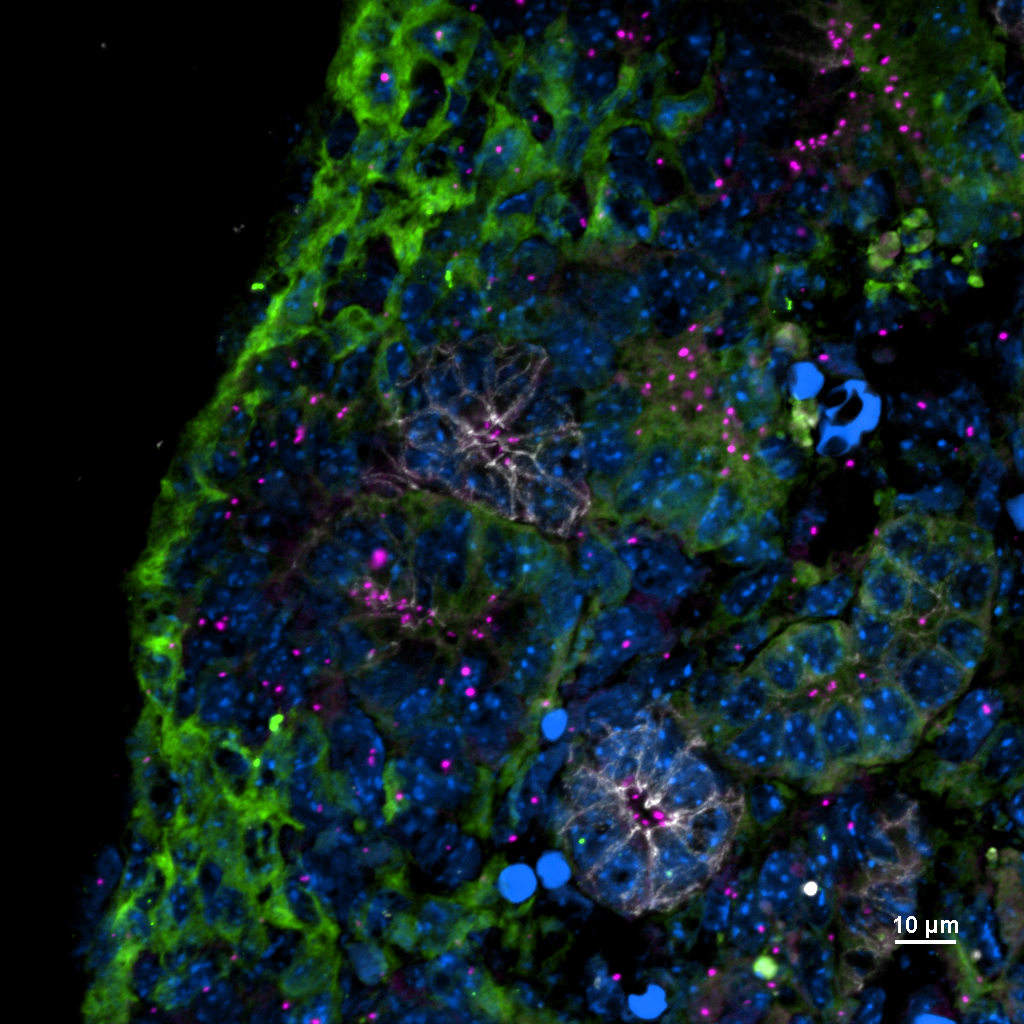

Supplement: Supplementary file 7 — Source Data Fig. 1 [file 44319_2023_19_MOESM7_ESM.zip › Fig.1/1E/Cep120-KO_Aldh1_g-tubulin_Ecad-MaxIP_RGB.tif]

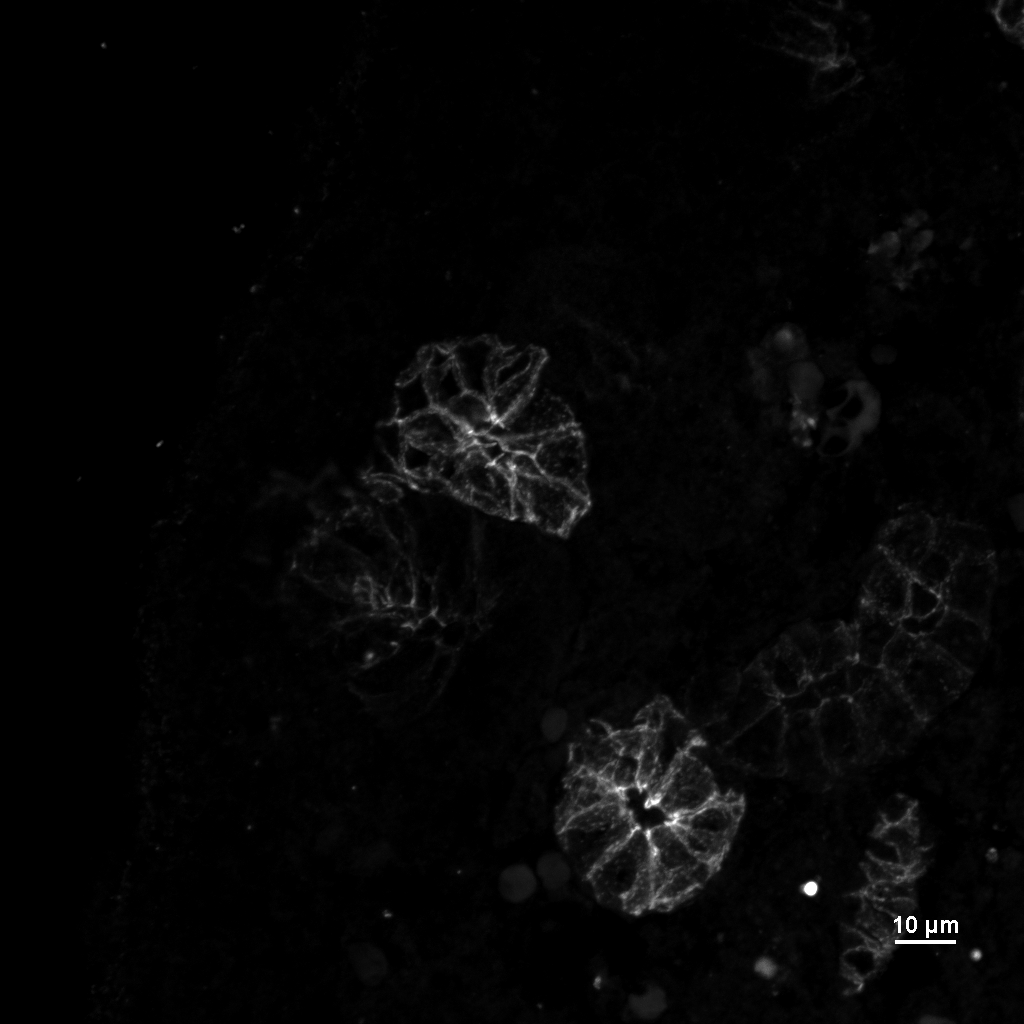

Supplement: Supplementary file 7 — Source Data Fig. 1 [file 44319_2023_19_MOESM7_ESM.zip › Fig.1/1E/Cep120-KO_Aldh1_g-tubulin_Ecad-MaxIP_RGB_561-SD.tif]

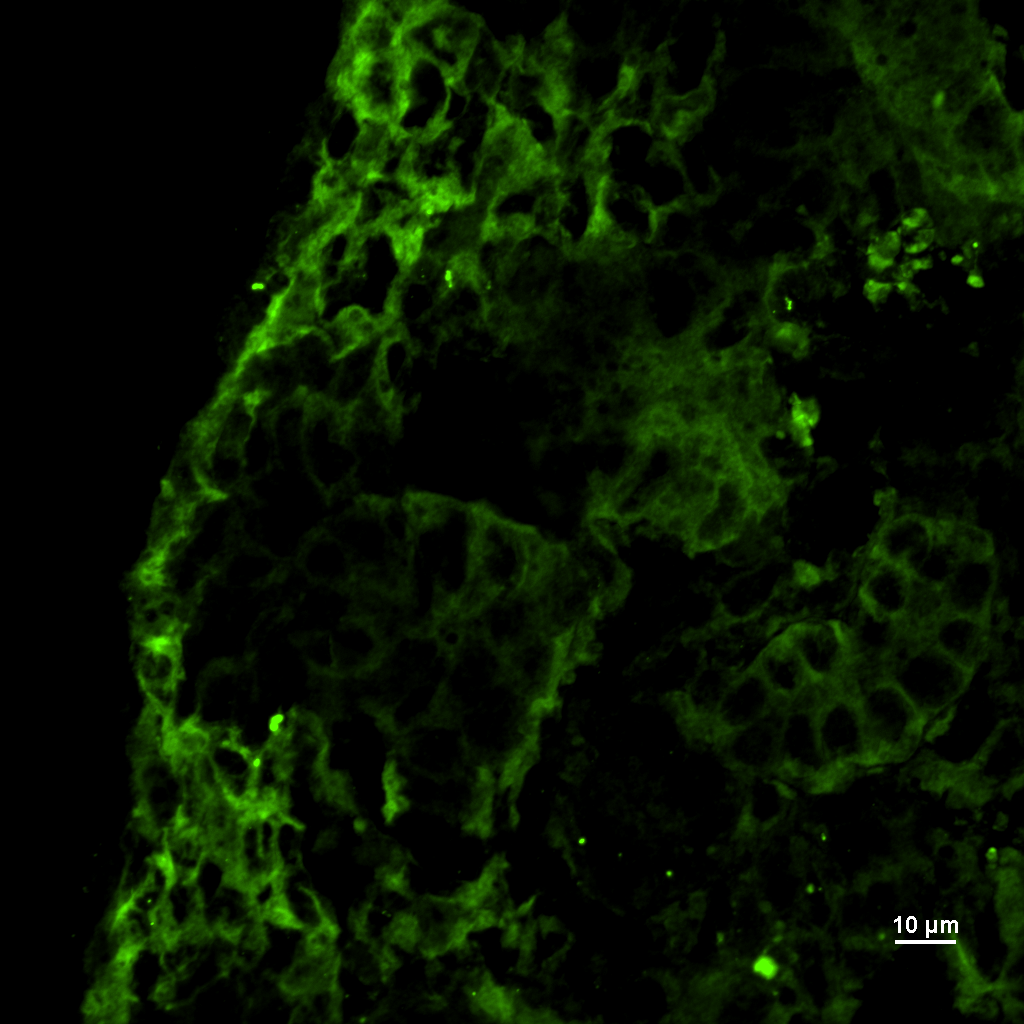

Supplement: Supplementary file 7 — Source Data Fig. 1 [file 44319_2023_19_MOESM7_ESM.zip › Fig.1/1E/Cep120-KO_Aldh1_g-tubulin_Ecad-MaxIP_RGB_488-SD.tif]

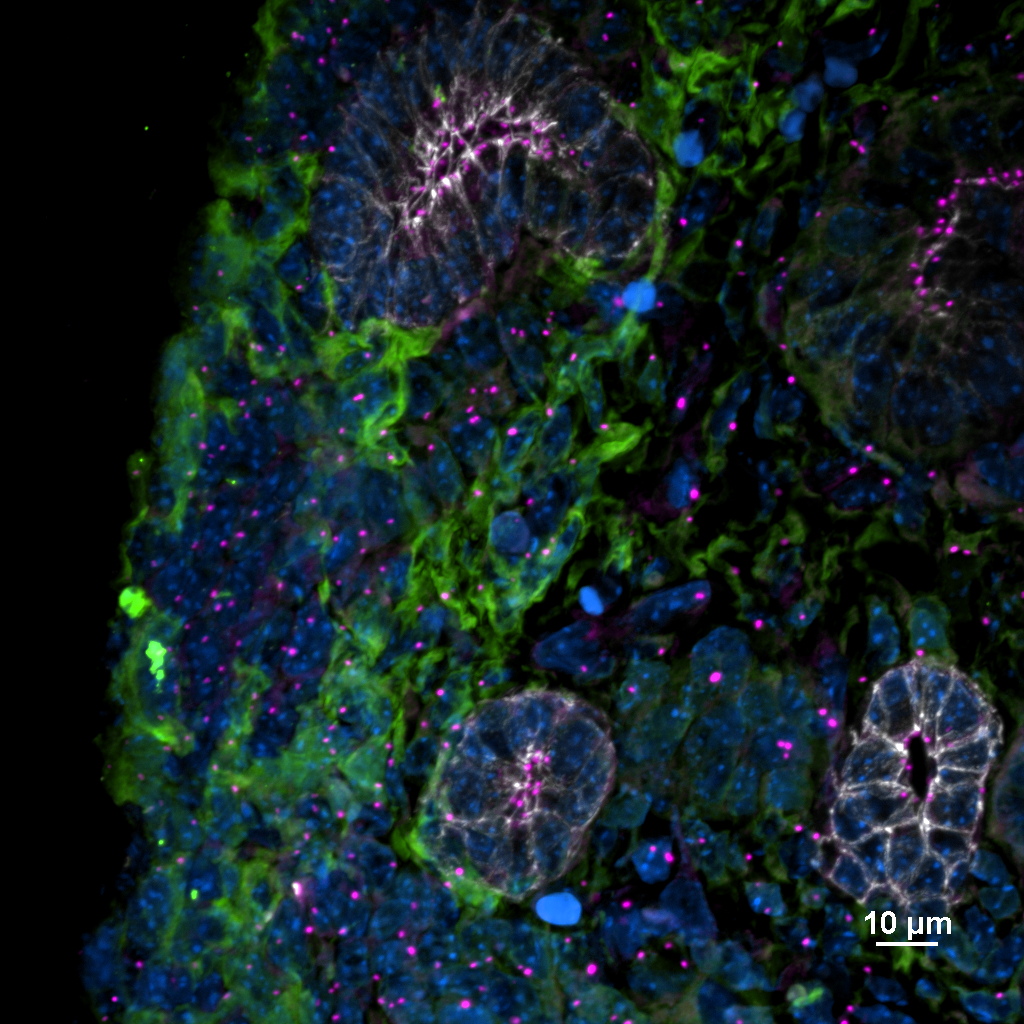

Supplement: Supplementary file 7 — Source Data Fig. 1 [file 44319_2023_19_MOESM7_ESM.zip › Fig.1/1E/Ctrl_Aldh1_g- tubulin_Ecad_-MaxIP_RGB.tif]

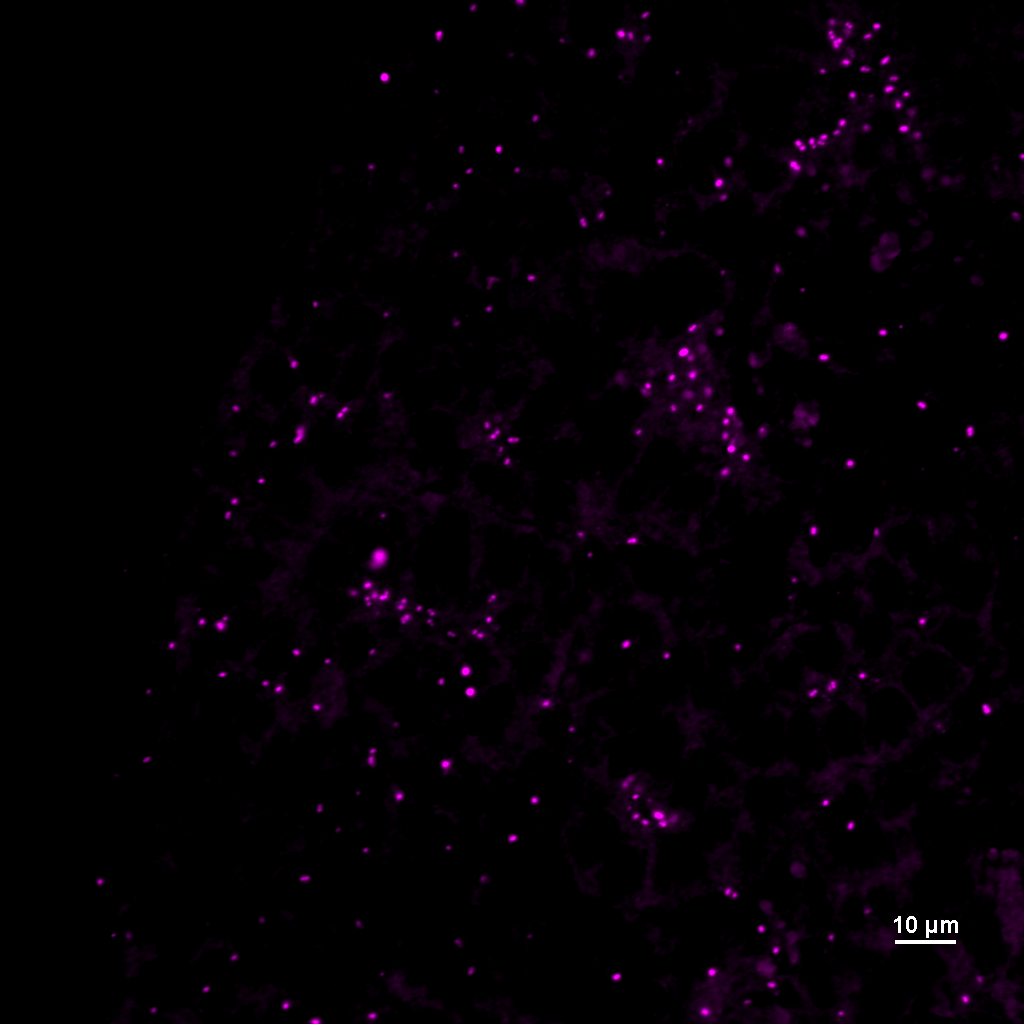

Supplement: Supplementary file 7 — Source Data Fig. 1 [file 44319_2023_19_MOESM7_ESM.zip › Fig.1/1E/Cep120-KO_Aldh1_g-tubulin_Ecad-MaxIP_RGB_640-SD.tif]

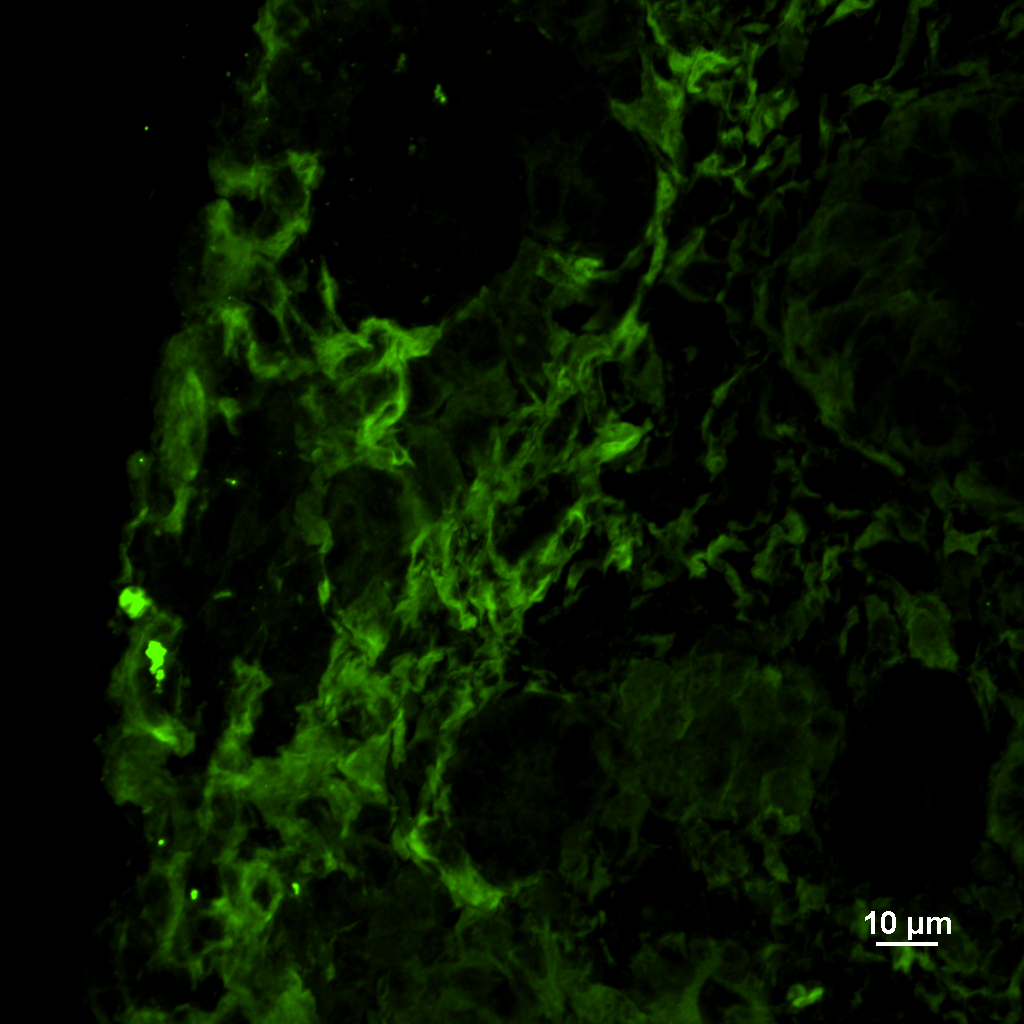

Supplement: Supplementary file 7 — Source Data Fig. 1 [file 44319_2023_19_MOESM7_ESM.zip › Fig.1/1E/Ctrl_Aldh1_g- tubulin_Ecad_-MaxIP_RGB_488-SD.tif]

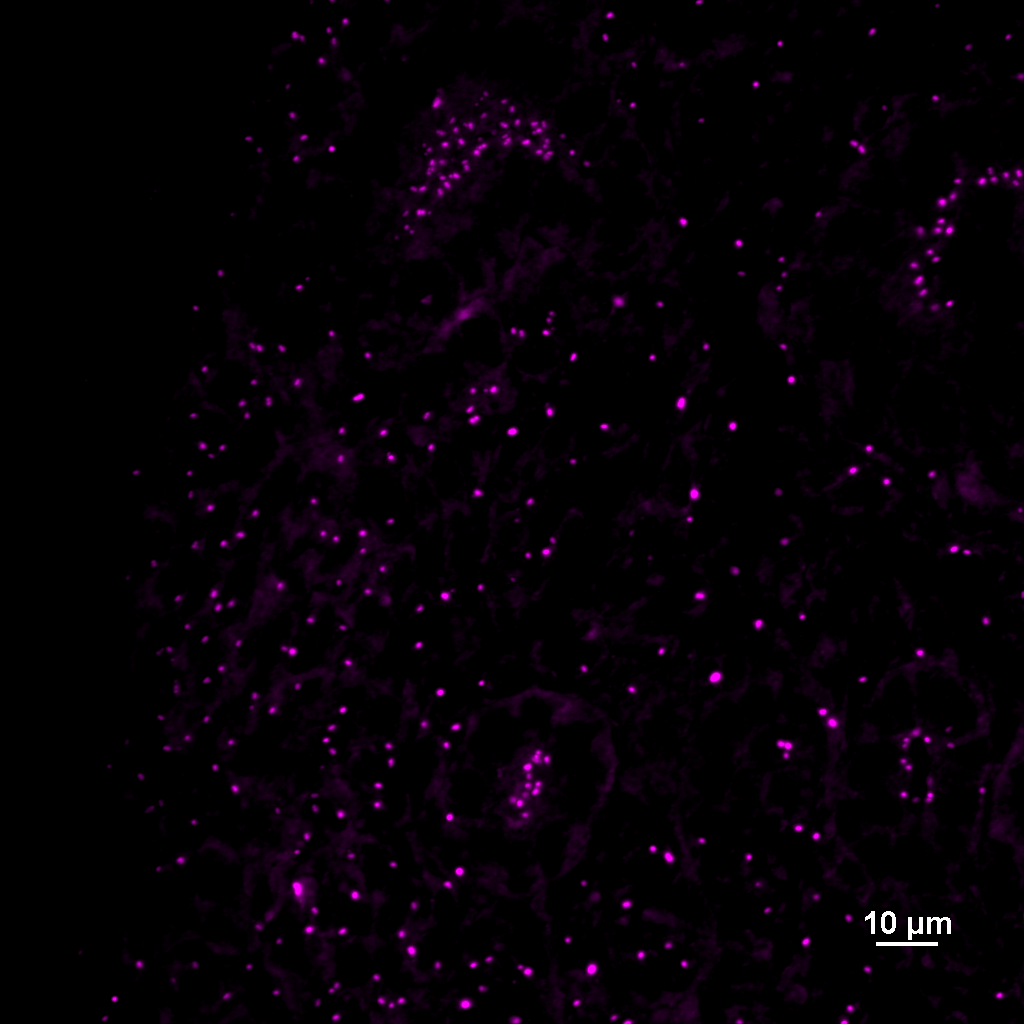

Supplement: Supplementary file 7 — Source Data Fig. 1 [file 44319_2023_19_MOESM7_ESM.zip › Fig.1/1E/Ctrl_Aldh1_g- tubulin_Ecad_-MaxIP_RGB_640-SD.tif]

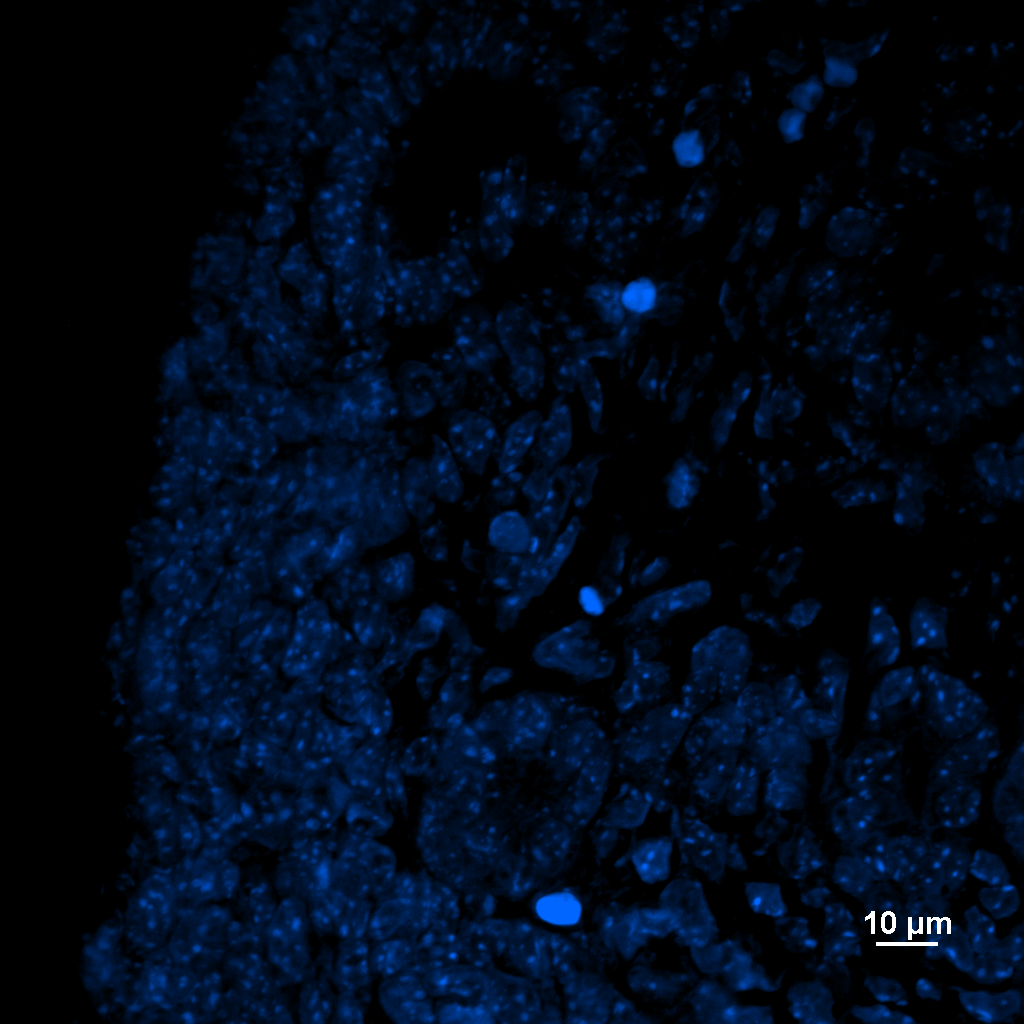

Supplement: Supplementary file 7 — Source Data Fig. 1 [file 44319_2023_19_MOESM7_ESM.zip › Fig.1/1E/Ctrl_Aldh1_g- tubulin_Ecad_-MaxIP_RGB_405-SD .tif]

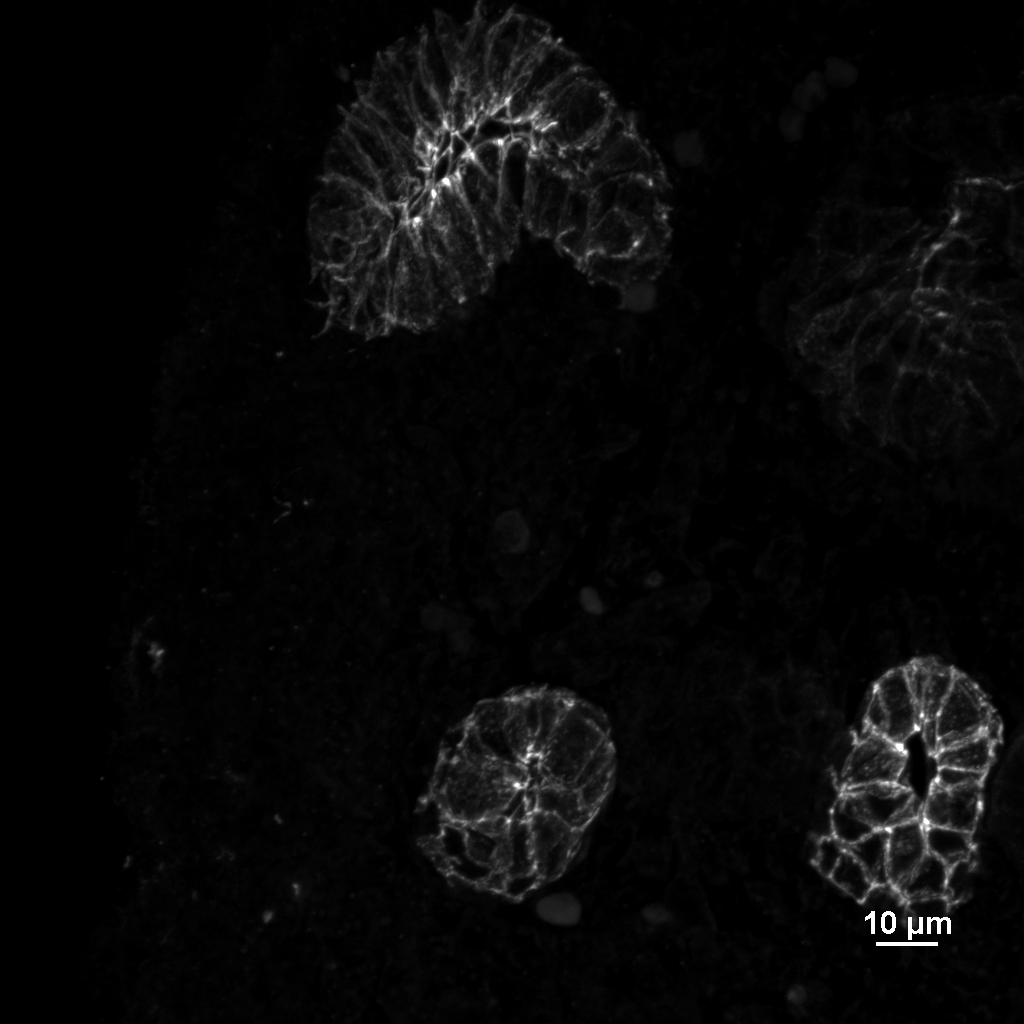

Supplement: Supplementary file 7 — Source Data Fig. 1 [file 44319_2023_19_MOESM7_ESM.zip › Fig.1/1E/Ctrl_Aldh1_g- tubulin_Ecad_-MaxIP_RGB_561-SD.tif]

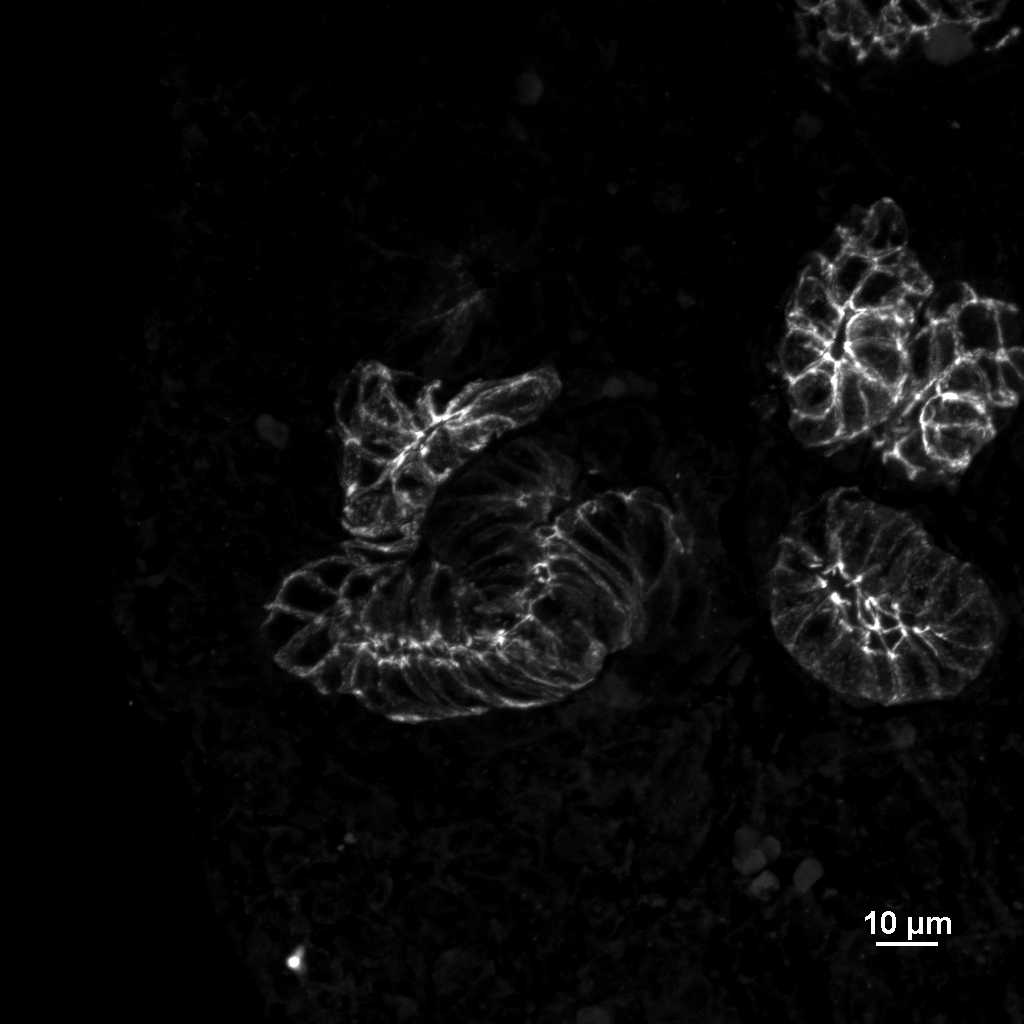

Supplement: Supplementary file 7 — Source Data Fig. 1 [file 44319_2023_19_MOESM7_ESM.zip › Fig.1/1C/Cep120-KO_Aldh1_Cep120_Ecad-MaxIP_RGB_561-SD.tif]

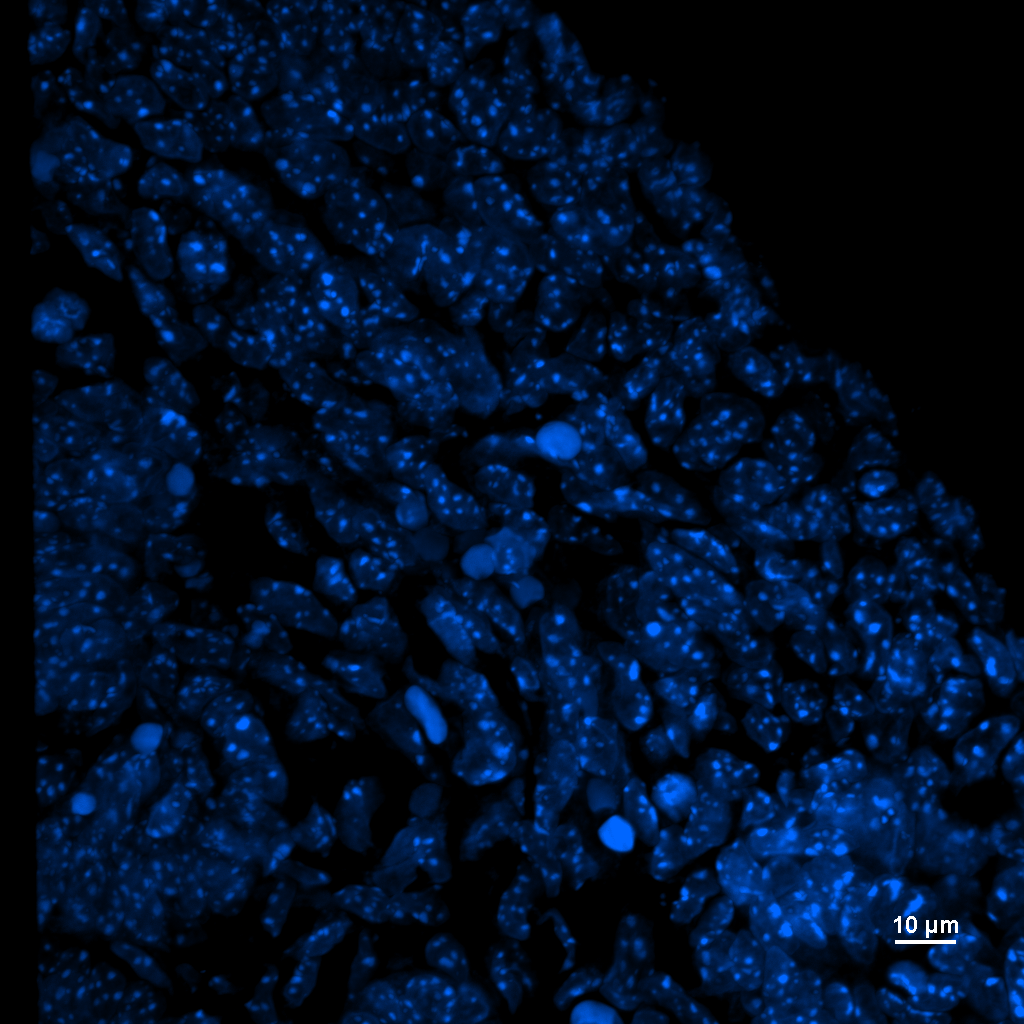

Supplement: Supplementary file 7 — Source Data Fig. 1 [file 44319_2023_19_MOESM7_ESM.zip › Fig.1/1C/Ctrl_Aldh1_Cep120_Ecad-MaxIP_RGB_405-SD .tif]

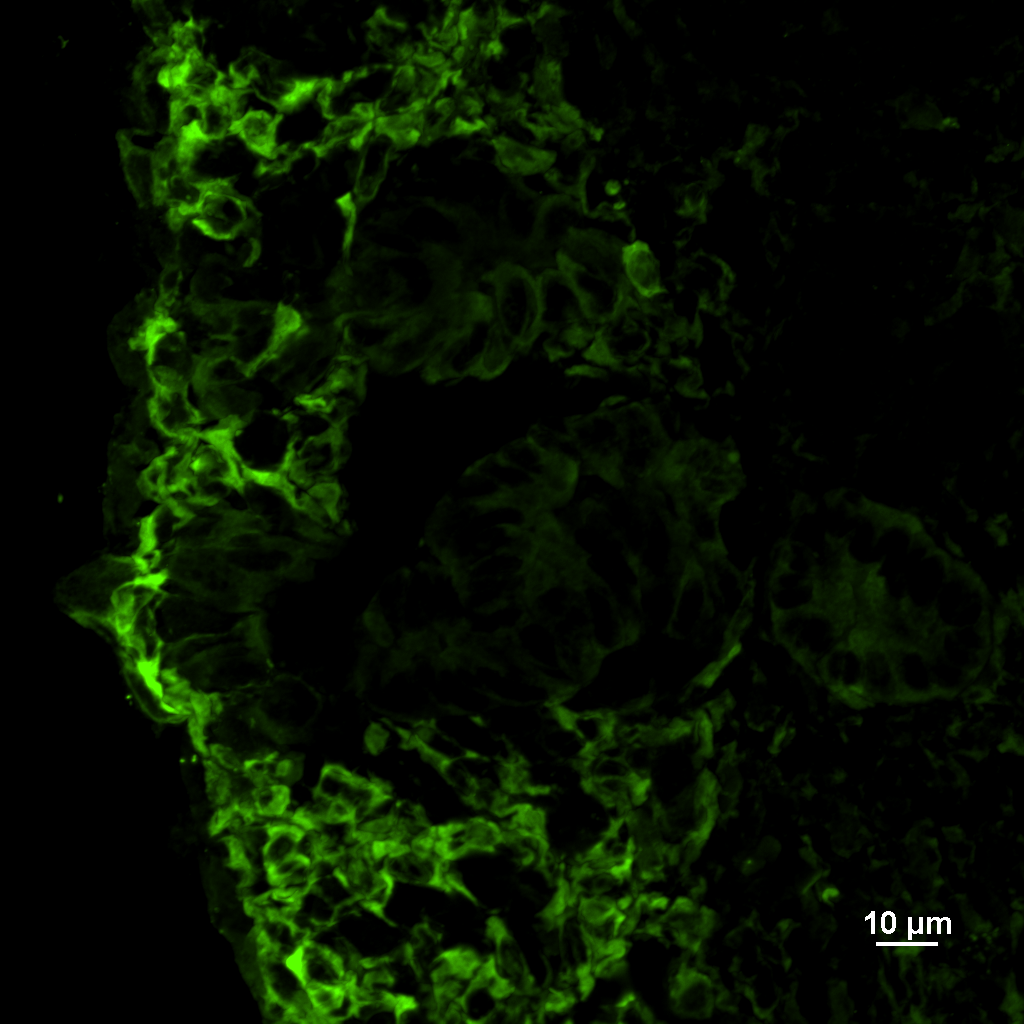

Supplement: Supplementary file 7 — Source Data Fig. 1 [file 44319_2023_19_MOESM7_ESM.zip › Fig.1/1C/Cep120-KO_Aldh1_Cep120_Ecad-MaxIP_RGB_488-SD.tif]

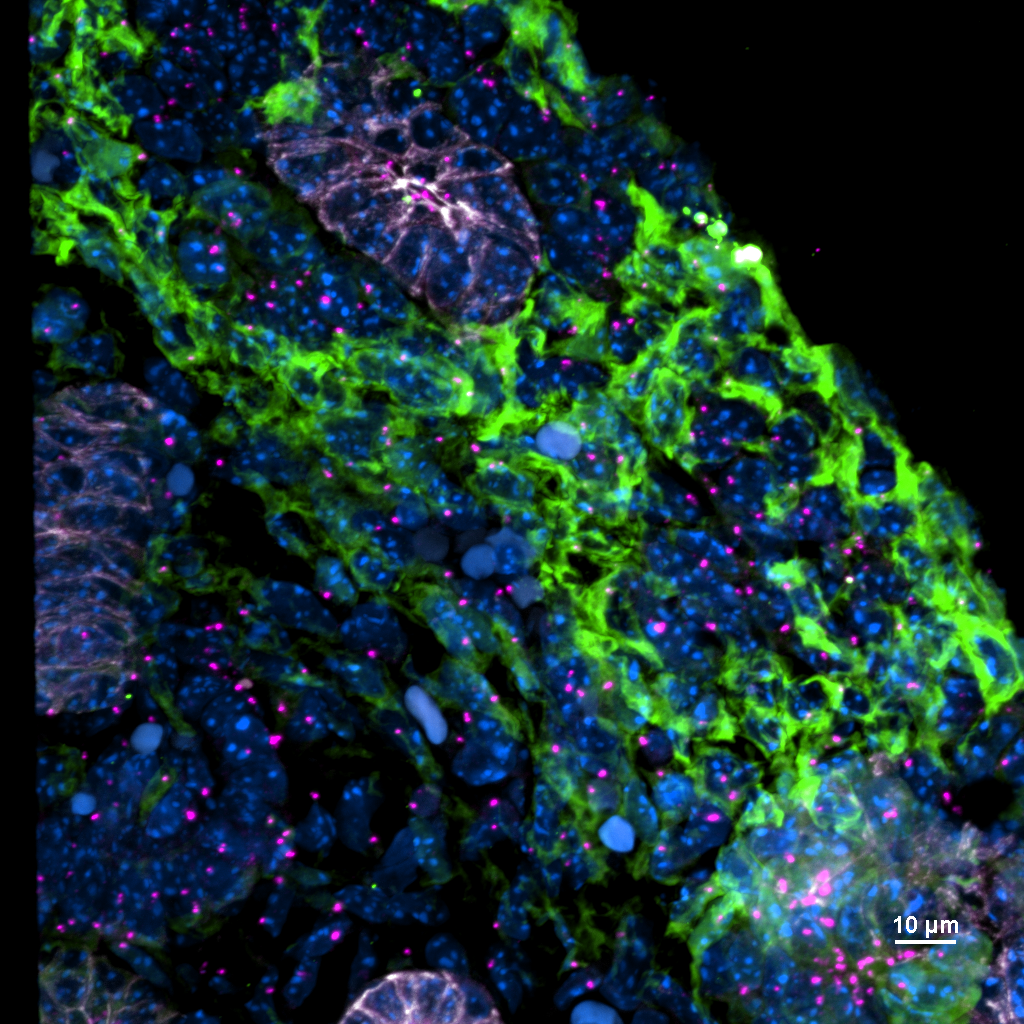

Supplement: Supplementary file 7 — Source Data Fig. 1 [file 44319_2023_19_MOESM7_ESM.zip › Fig.1/1C/Ctrl_Aldh1_Cep120_Ecad-MaxIP_RGB.tif]

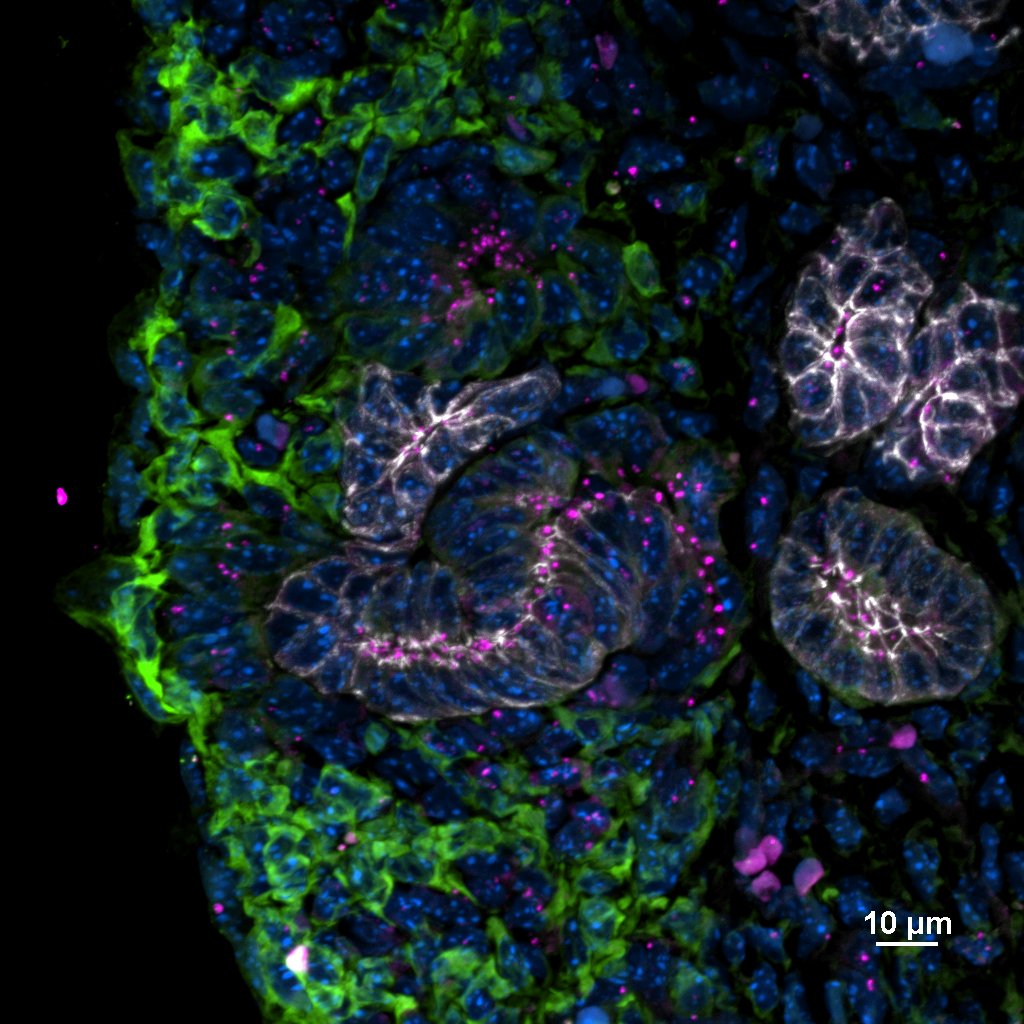

Supplement: Supplementary file 7 — Source Data Fig. 1 [file 44319_2023_19_MOESM7_ESM.zip › Fig.1/1C/Cep120-KO_Aldh1_Cep120_Ecad-MaxIP_RGB.tif]

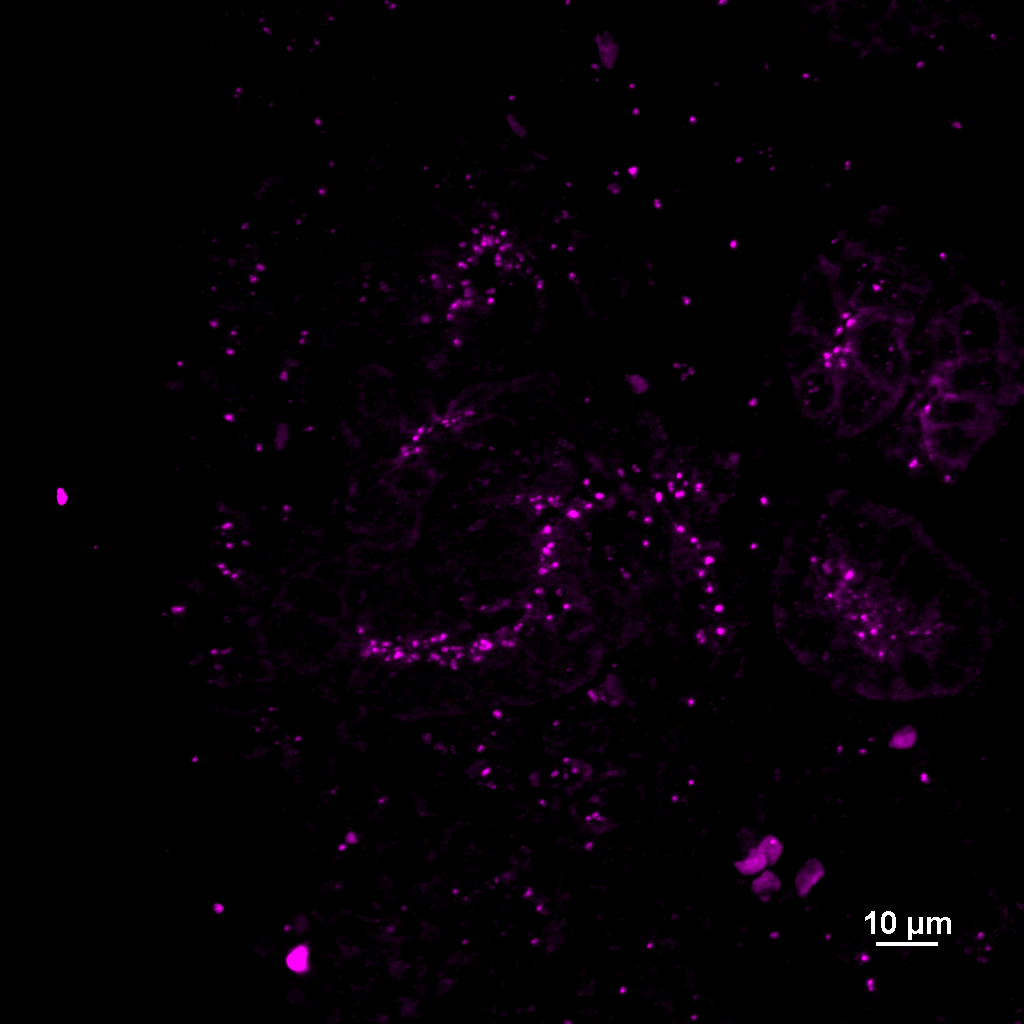

Supplement: Supplementary file 7 — Source Data Fig. 1 [file 44319_2023_19_MOESM7_ESM.zip › Fig.1/1C/Cep120-KO_Aldh1_Cep120_Ecad-MaxIP_RGB_640-SD.tif]

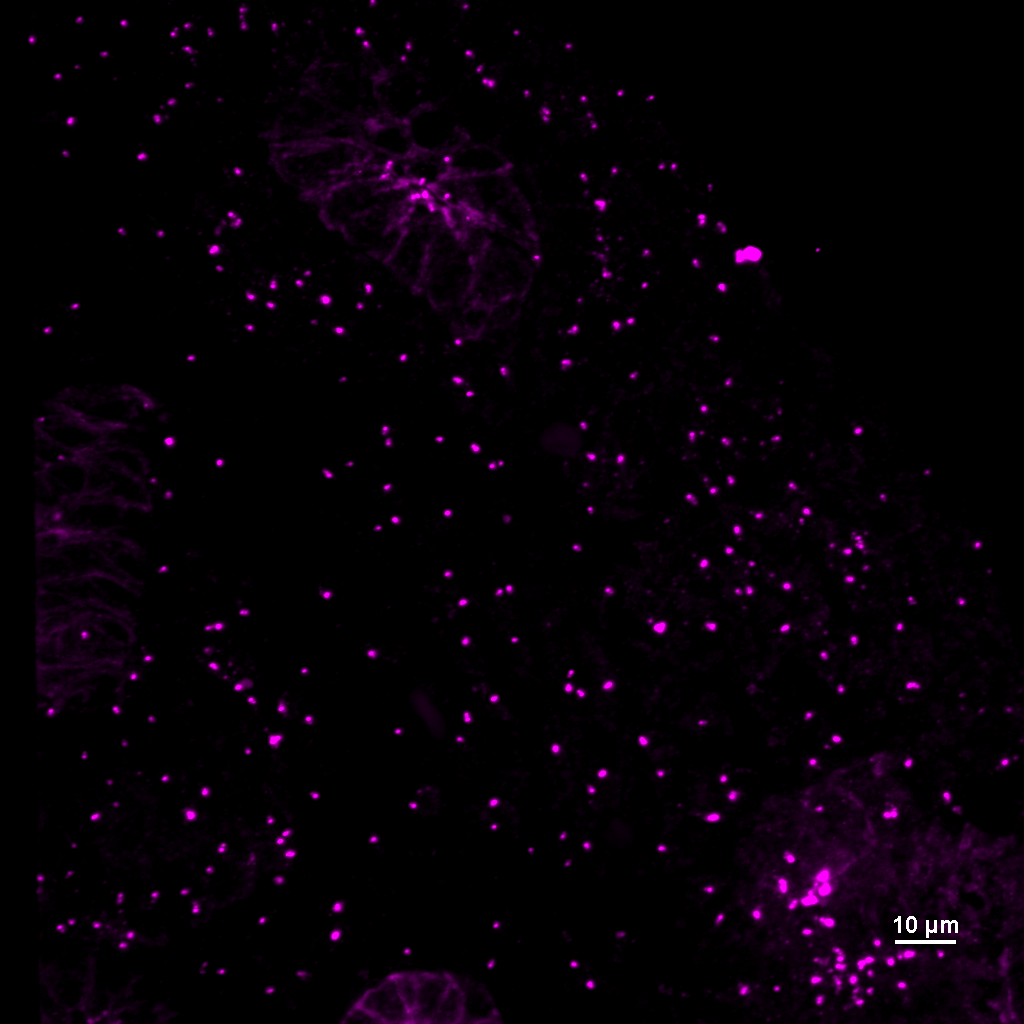

Supplement: Supplementary file 7 — Source Data Fig. 1 [file 44319_2023_19_MOESM7_ESM.zip › Fig.1/1C/Ctrl_Aldh1_Cep120_Ecad-MaxIP_RGB_640-SD.tif]

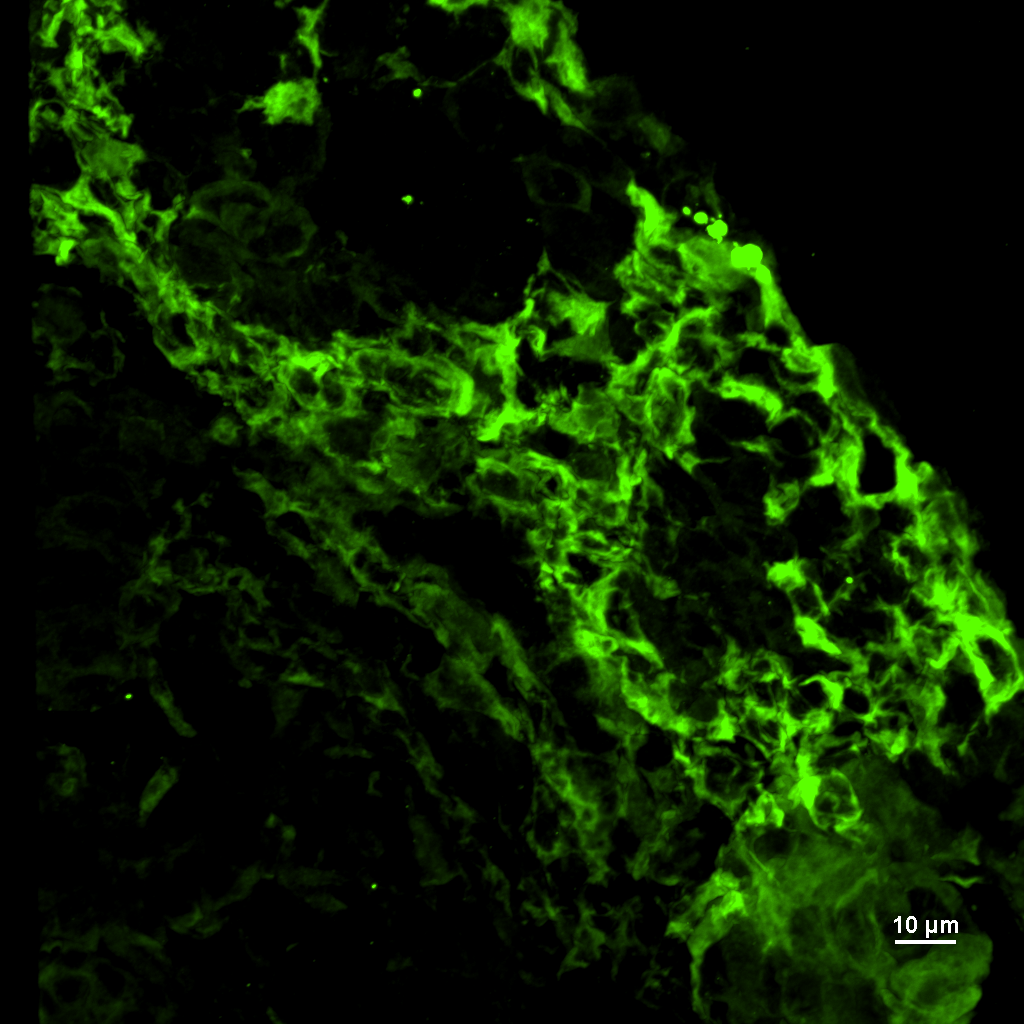

Supplement: Supplementary file 7 — Source Data Fig. 1 [file 44319_2023_19_MOESM7_ESM.zip › Fig.1/1C/Ctrl_Aldh1_Cep120_Ecad-MaxIP_RGB_488-SD.tif]

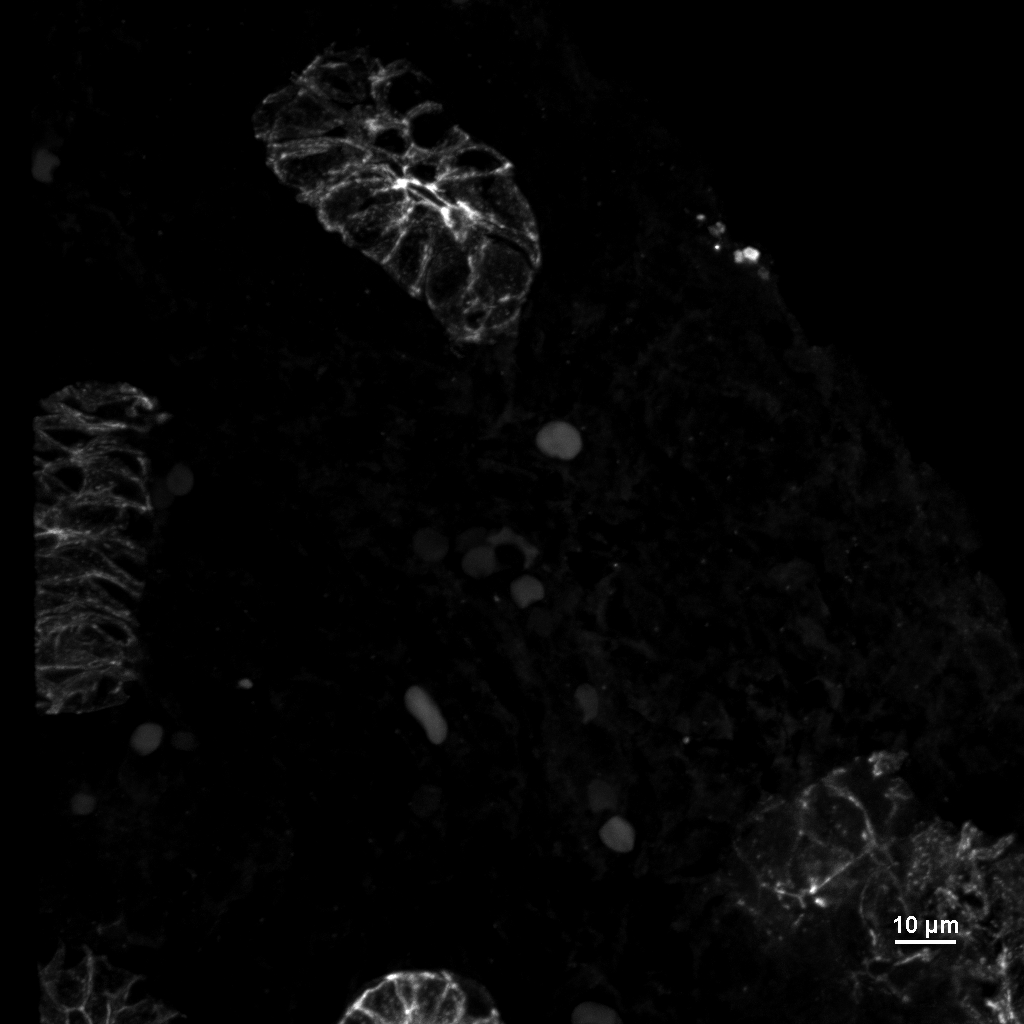

Supplement: Supplementary file 7 — Source Data Fig. 1 [file 44319_2023_19_MOESM7_ESM.zip › Fig.1/1C/Ctrl_Aldh1_Cep120_Ecad-MaxIP_RGB_561-SD.tif]

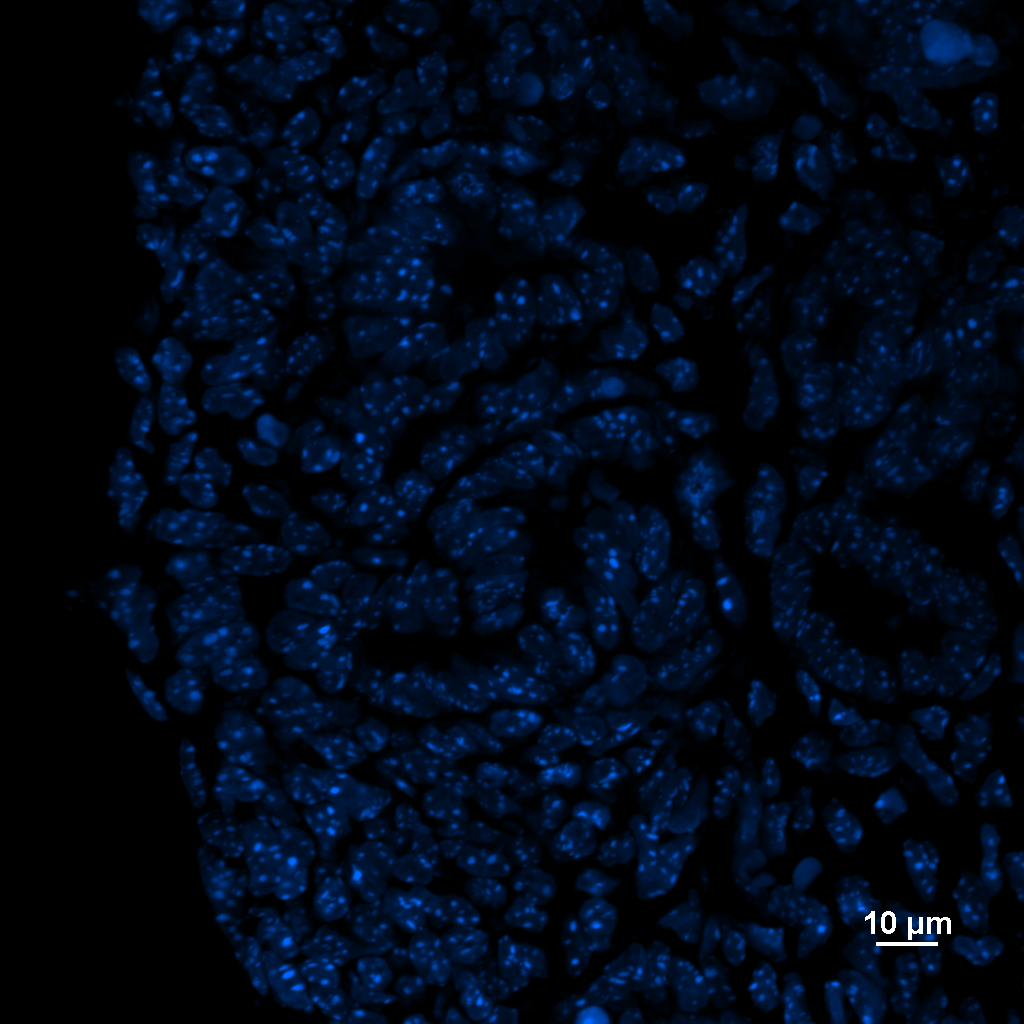

Supplement: Supplementary file 7 — Source Data Fig. 1 [file 44319_2023_19_MOESM7_ESM.zip › Fig.1/1C/Cep120-KO_Aldh1_Cep120_Ecad-MaxIP_RGB_405-SD .tif]

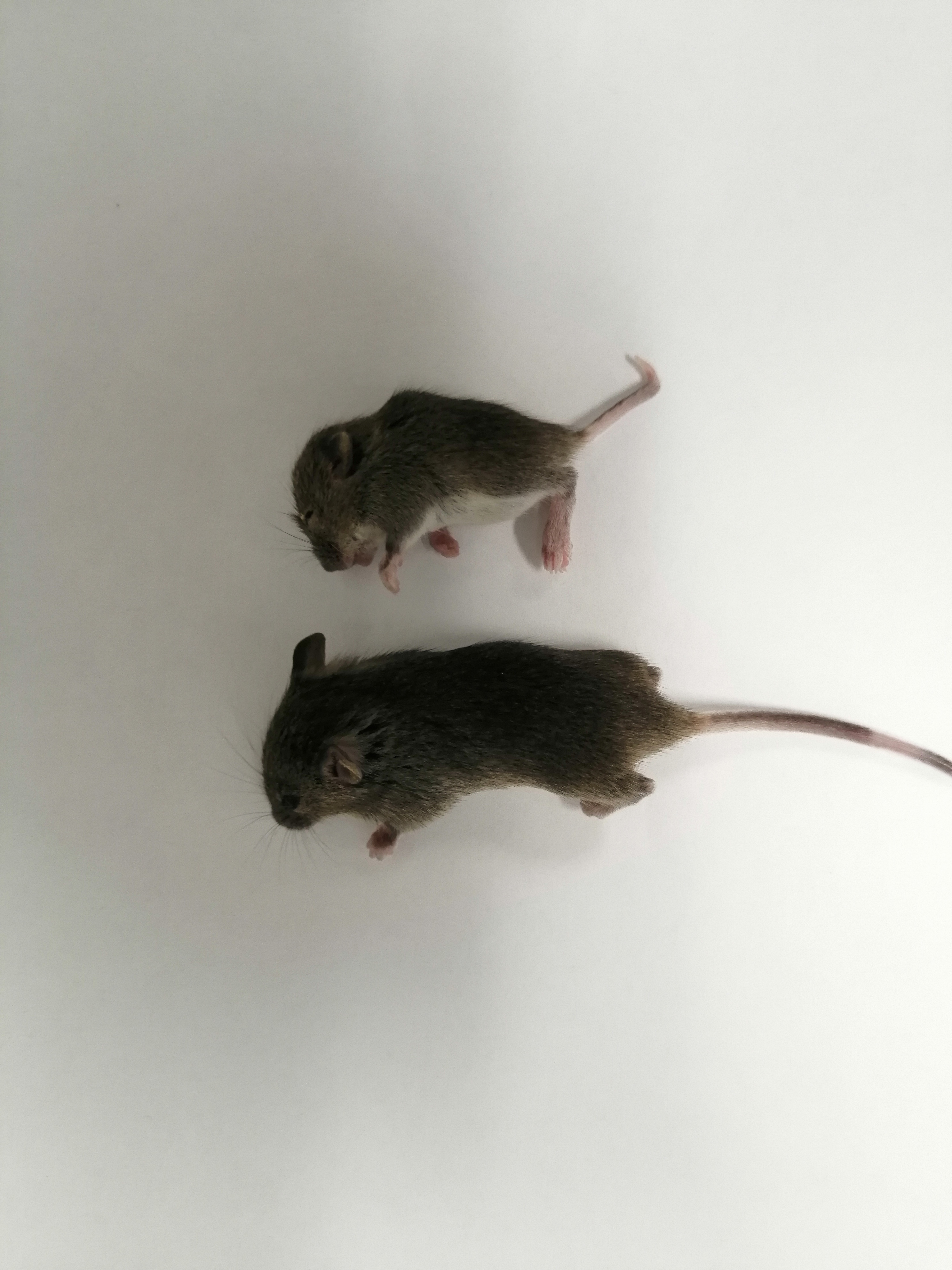

Supplement: Supplementary file 8 — Source Data Fig. 2 [file 44319_2023_19_MOESM8_ESM.zip › Fig.2/2A/image_Ctrl vs KO.jpg]

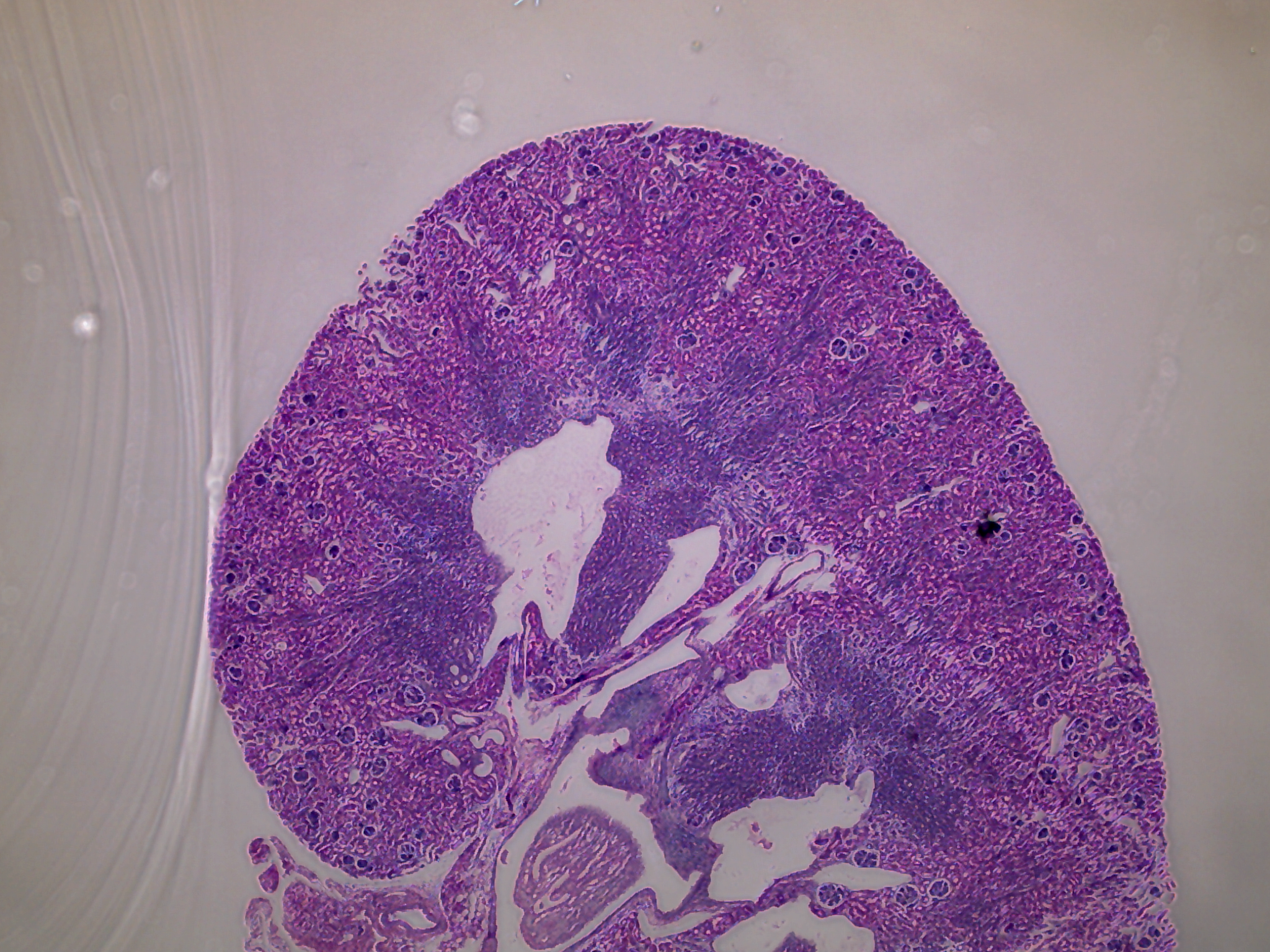

Supplement: Supplementary file 8 — Source Data Fig. 2 [file 44319_2023_19_MOESM8_ESM.zip › Fig.2/2C/KO-upper part.tif]

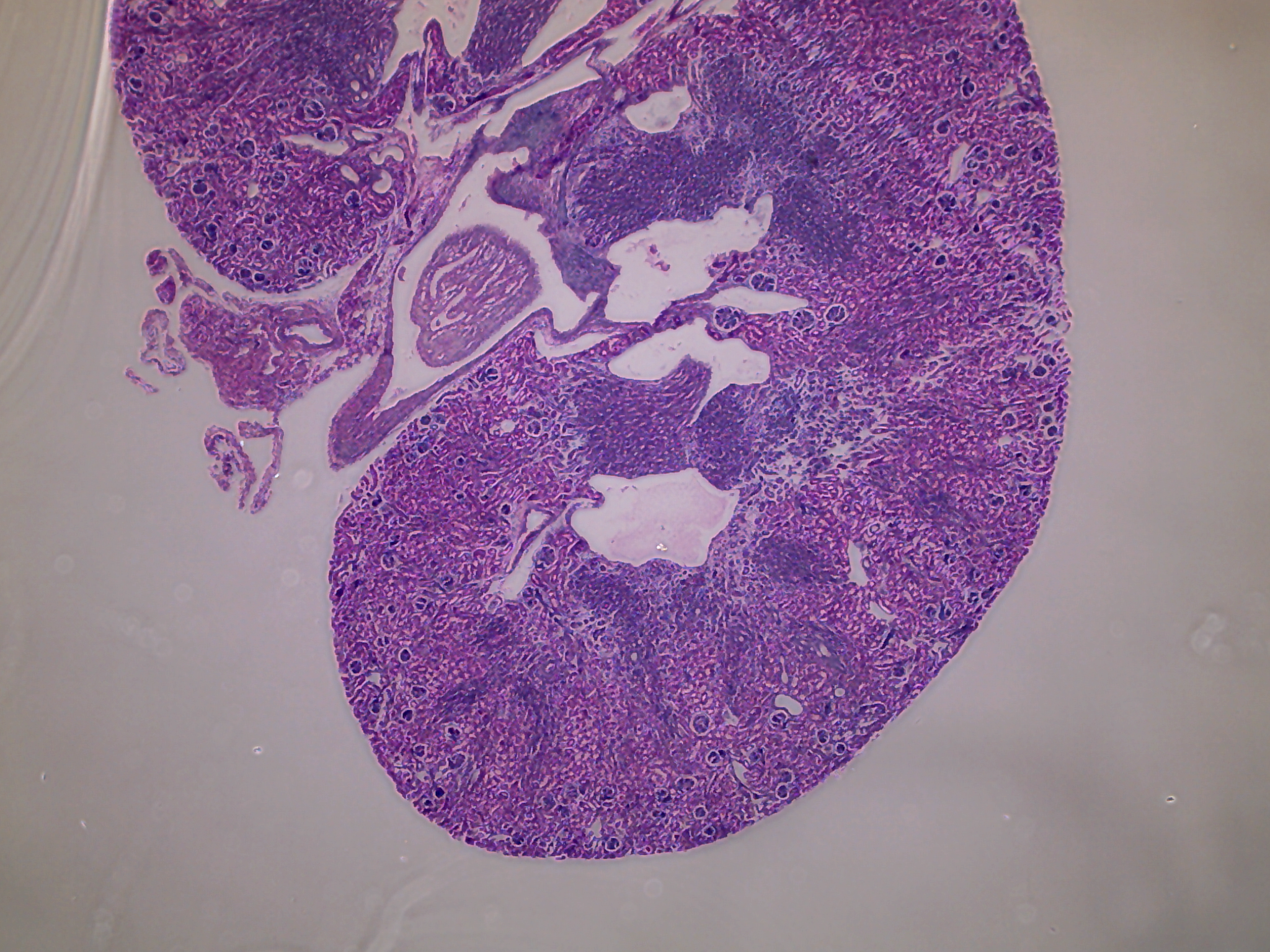

Supplement: Supplementary file 8 — Source Data Fig. 2 [file 44319_2023_19_MOESM8_ESM.zip › Fig.2/2C/KO-lower part.tif]

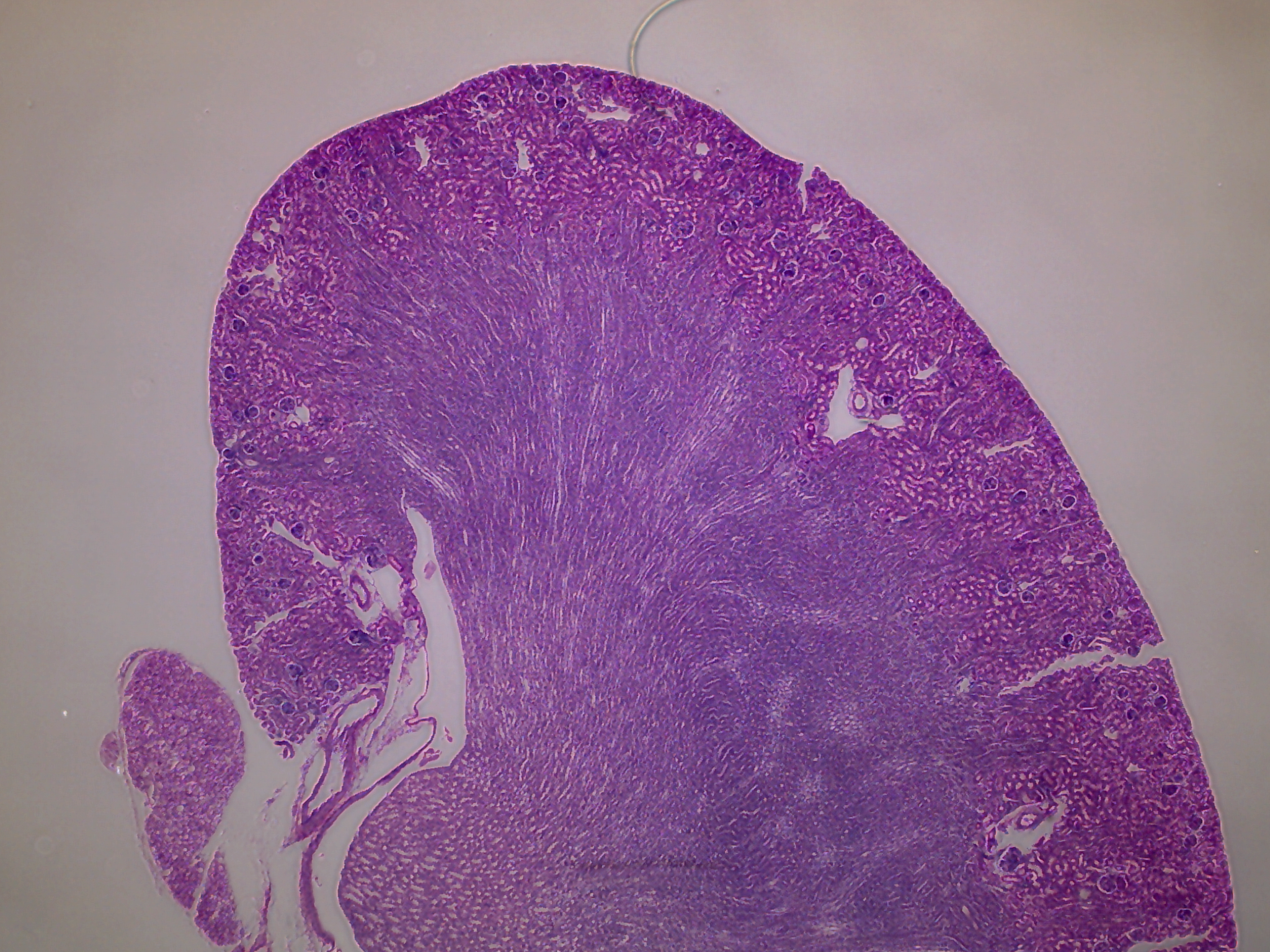

Supplement: Supplementary file 8 — Source Data Fig. 2 [file 44319_2023_19_MOESM8_ESM.zip › Fig.2/2C/Ctrl-upper part.tif]

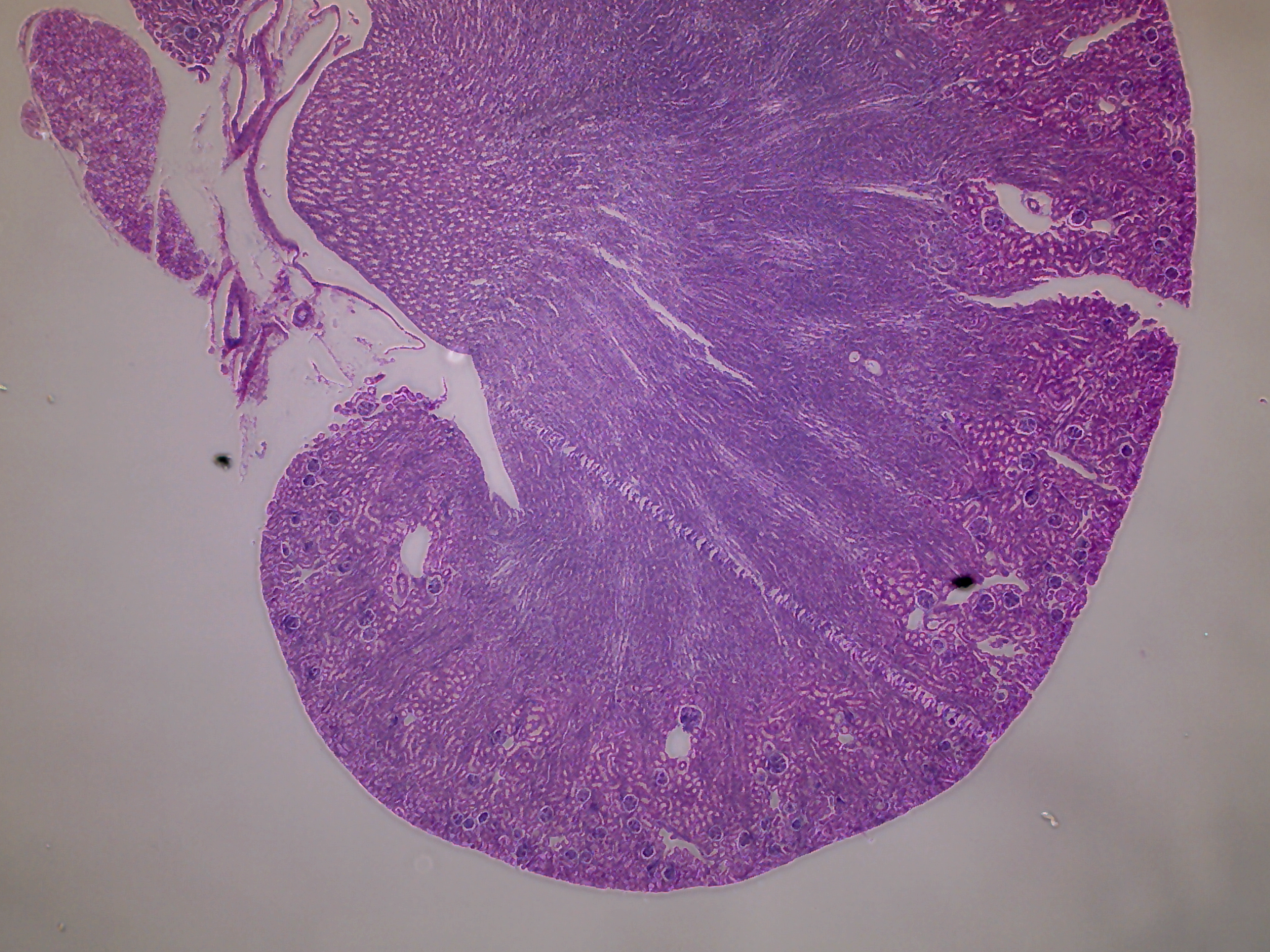

Supplement: Supplementary file 8 — Source Data Fig. 2 [file 44319_2023_19_MOESM8_ESM.zip › Fig.2/2C/Ctrl-lower part.tif]

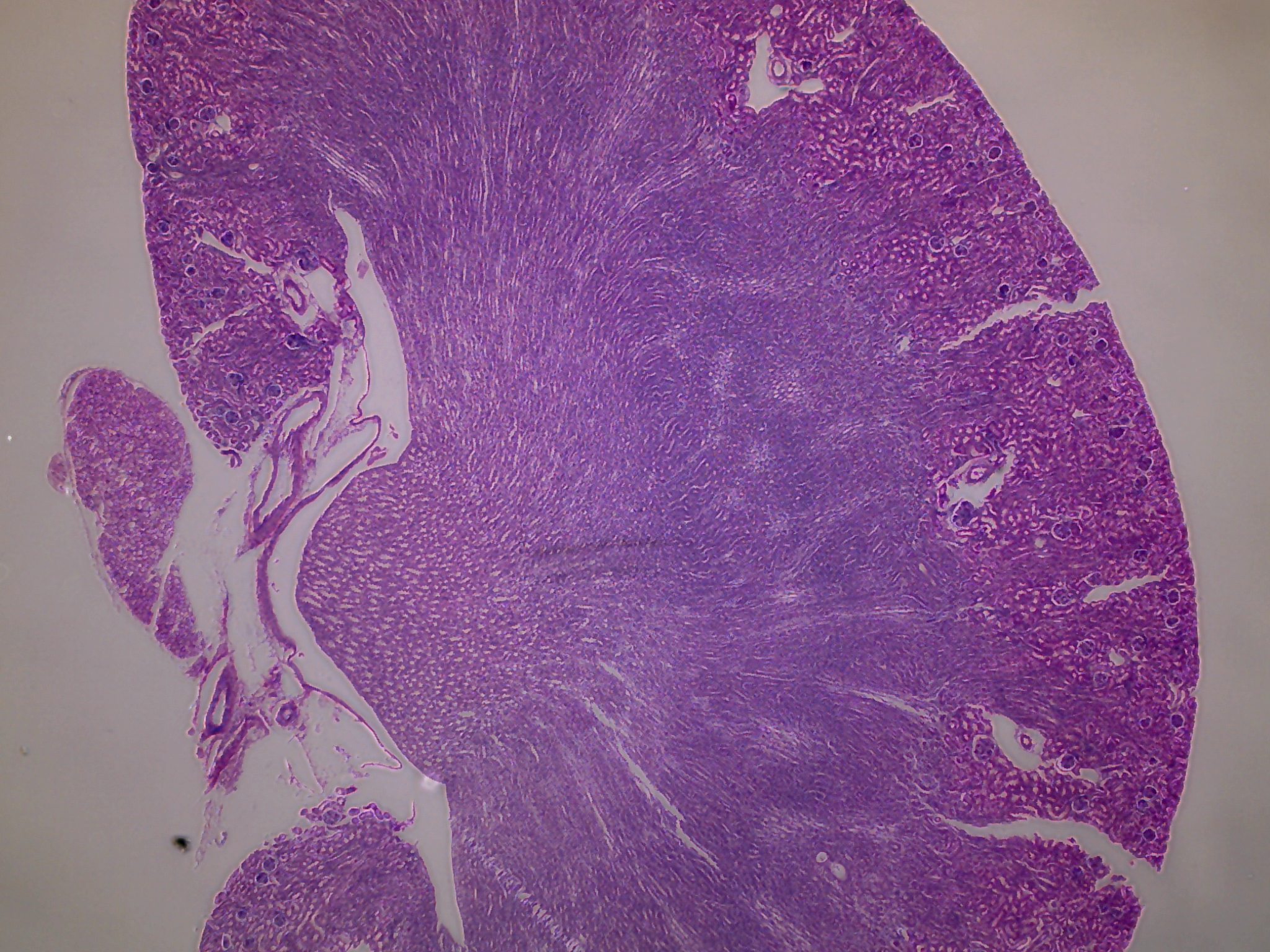

Supplement: Supplementary file 8 — Source Data Fig. 2 [file 44319_2023_19_MOESM8_ESM.zip › Fig.2/2C/Ctrl-middle part.tif]

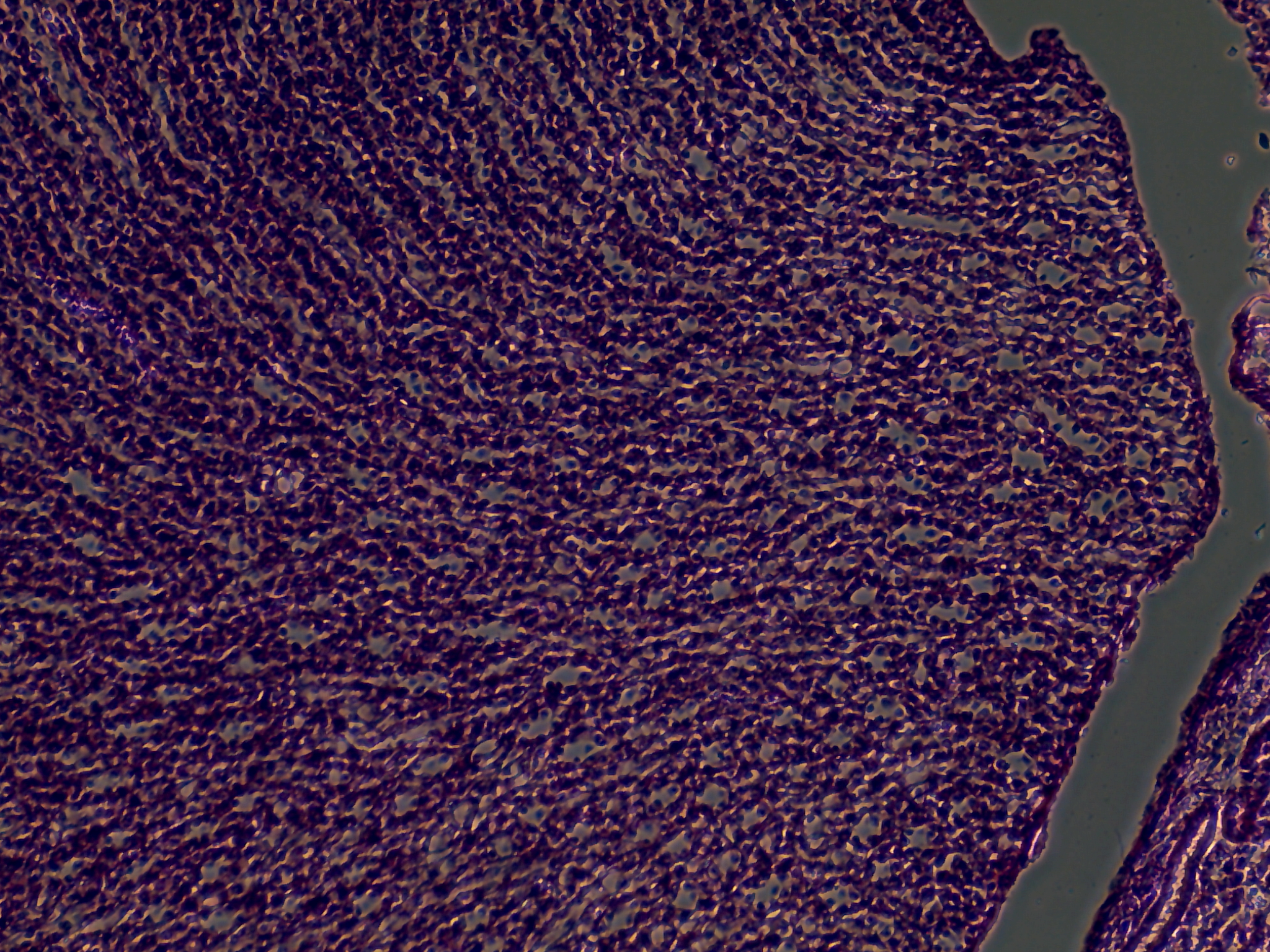

Supplement: Supplementary file 8 — Source Data Fig. 2 [file 44319_2023_19_MOESM8_ESM.zip › Fig.2/2D/Ctrl.tif]

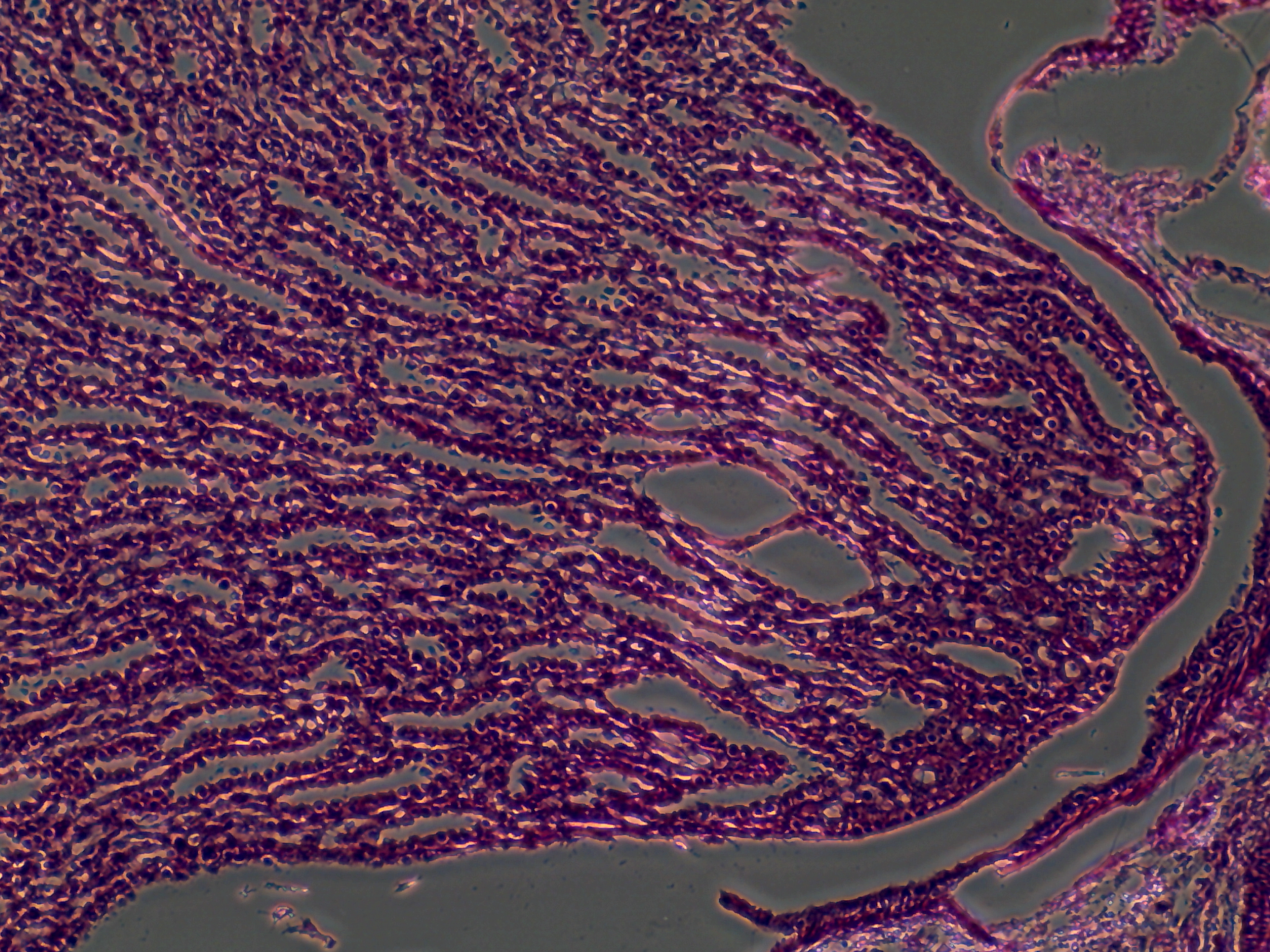

Supplement: Supplementary file 8 — Source Data Fig. 2 [file 44319_2023_19_MOESM8_ESM.zip › Fig.2/2D/KO.tif]

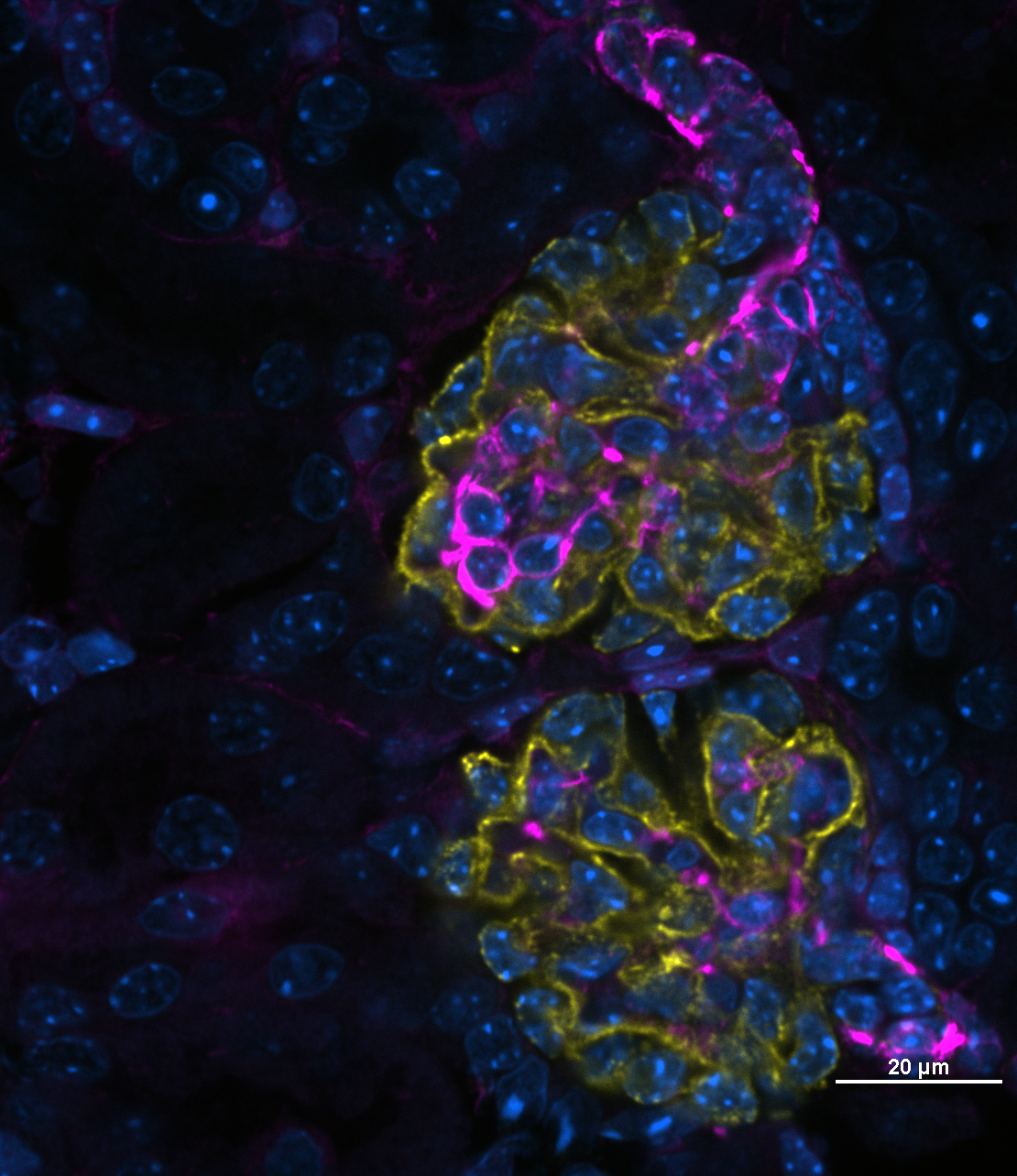

Supplement: Supplementary file 9 — Source Data Fig. 3 [file 44319_2023_19_MOESM9_ESM.zip › Fig.3/3D/Cep120-KO-Desmin_aSMA_SYNPO_RGB.tif]

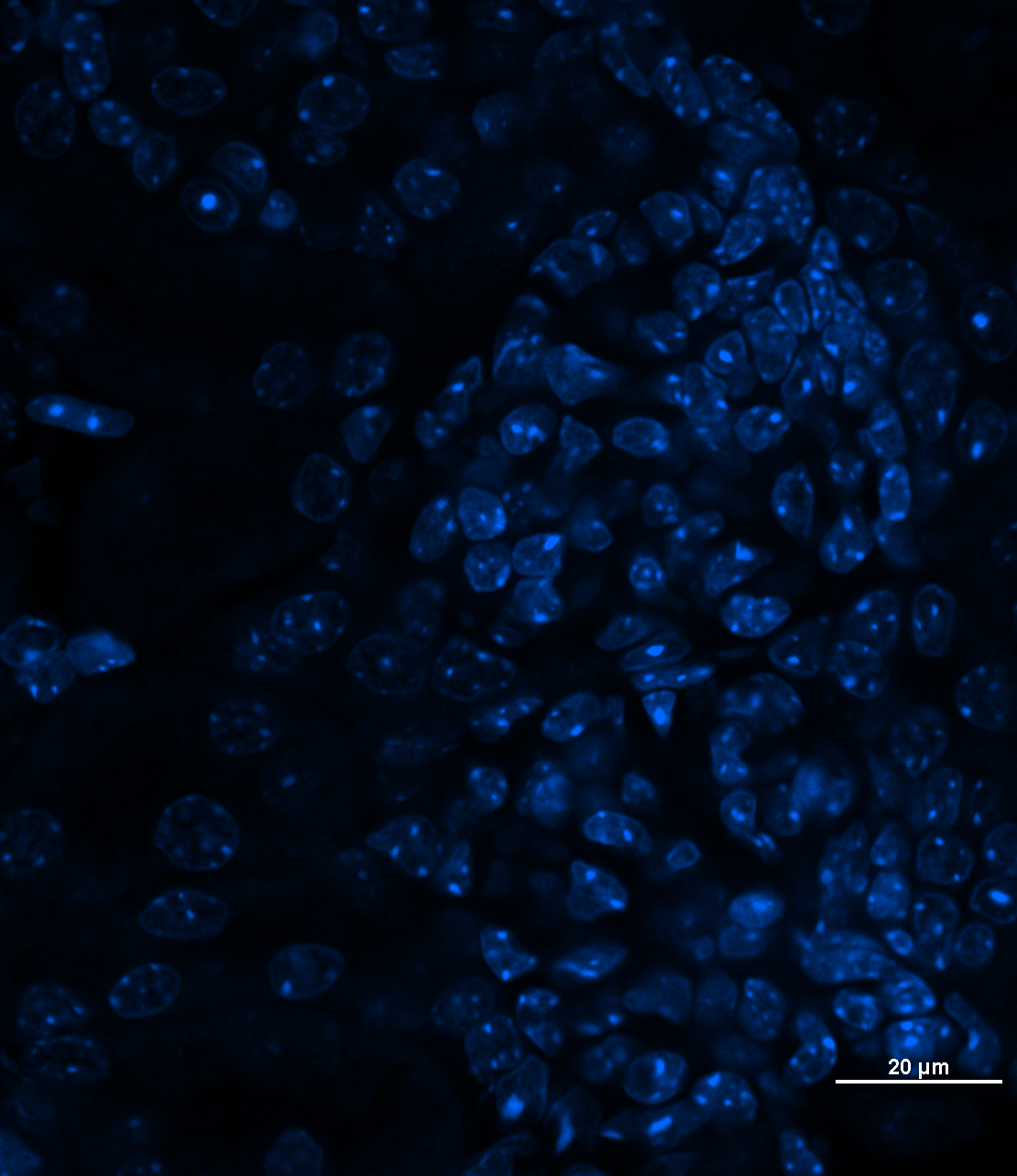

Supplement: Supplementary file 9 — Source Data Fig. 3 [file 44319_2023_19_MOESM9_ESM.zip › Fig.3/3D/Cep120-KO-Desmin_aSMA_SYNPO_RGB_405-SD .tif]

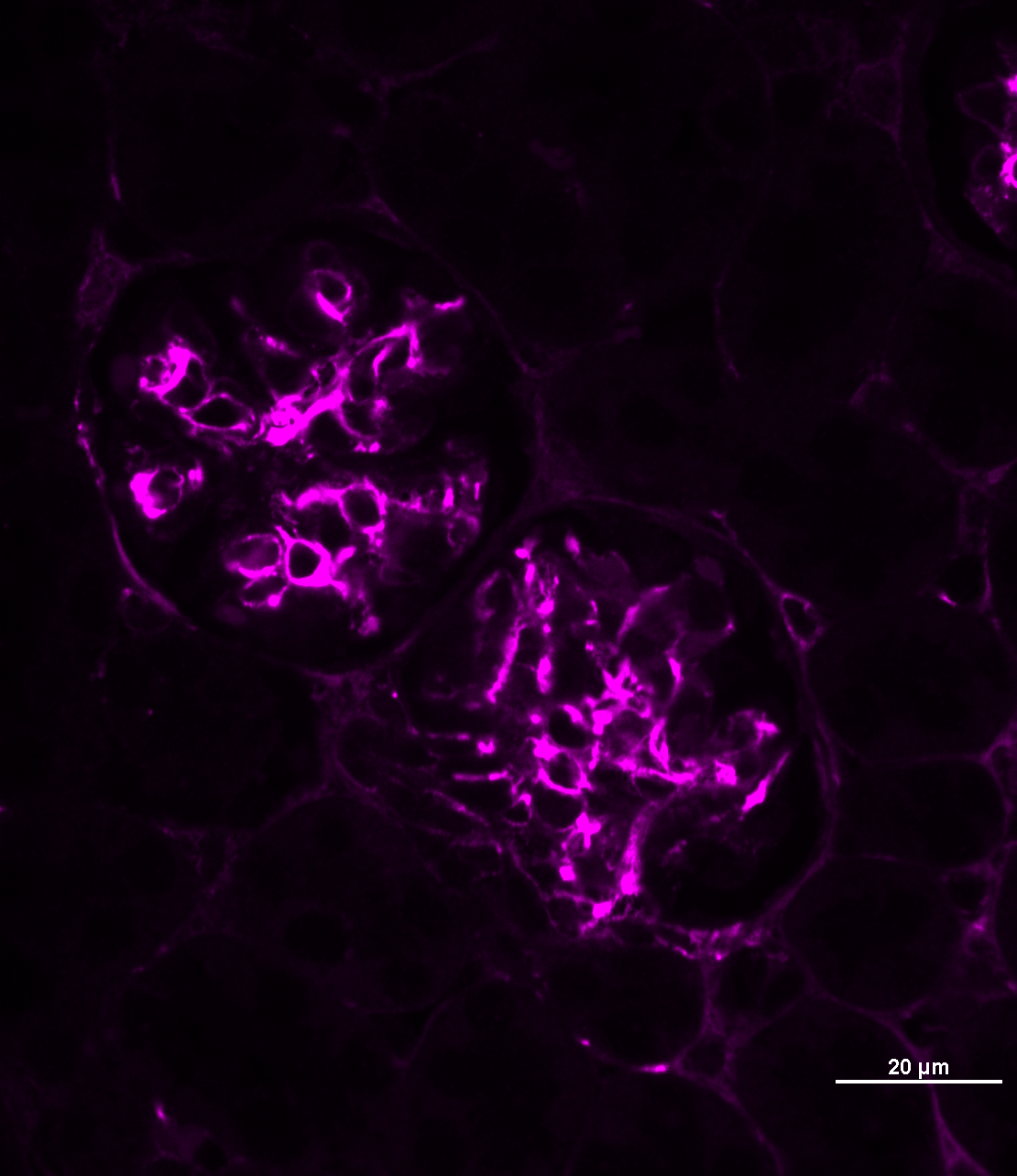

Supplement: Supplementary file 9 — Source Data Fig. 3 [file 44319_2023_19_MOESM9_ESM.zip › Fig.3/3D/Ctrl-Desmin_aSMA_SYNPO_RGB_488-SD.tif]

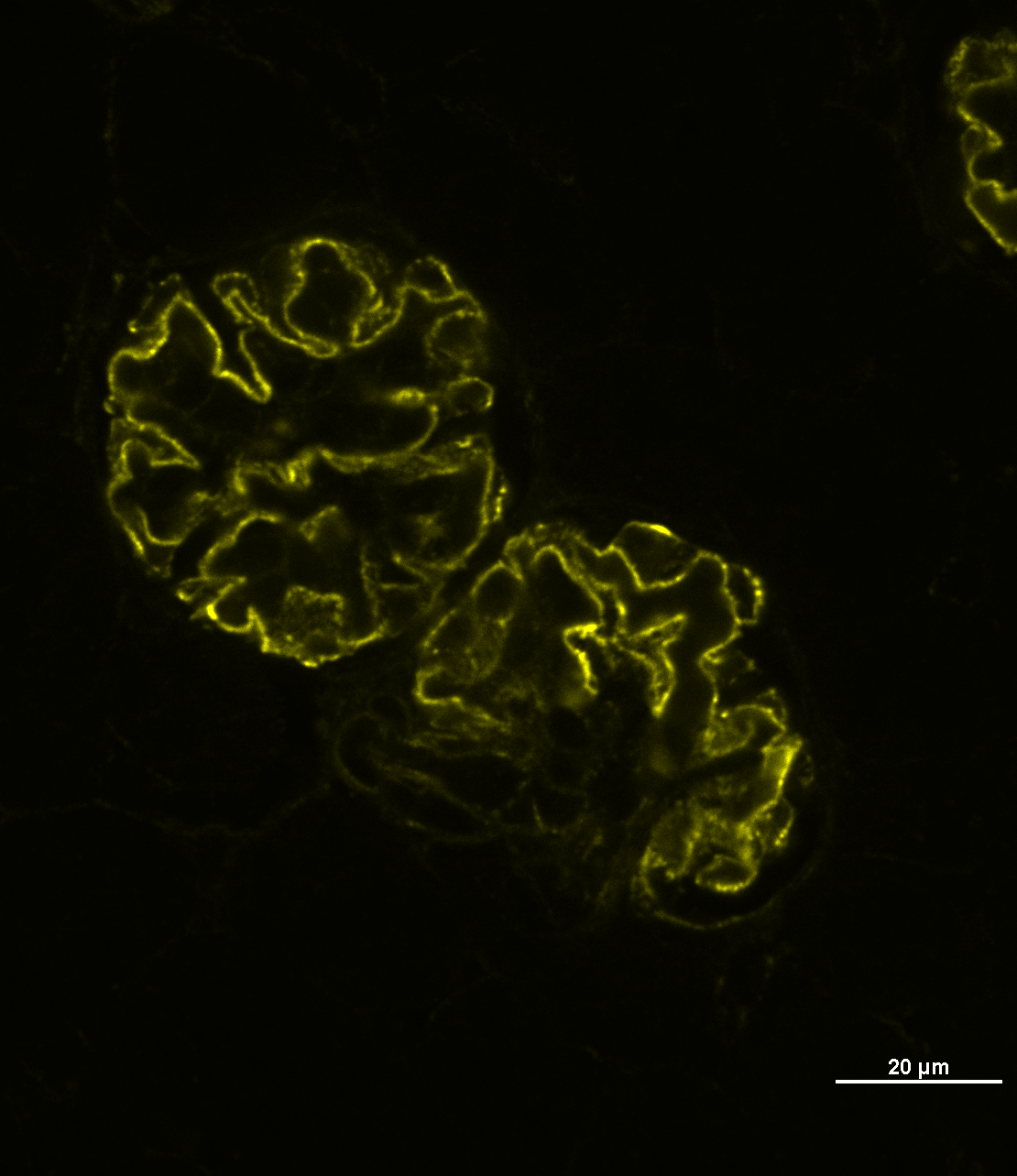

Supplement: Supplementary file 9 — Source Data Fig. 3 [file 44319_2023_19_MOESM9_ESM.zip › Fig.3/3D/Ctrl-Desmin_aSMA_SYNPO_RGB_640-SD.tif]

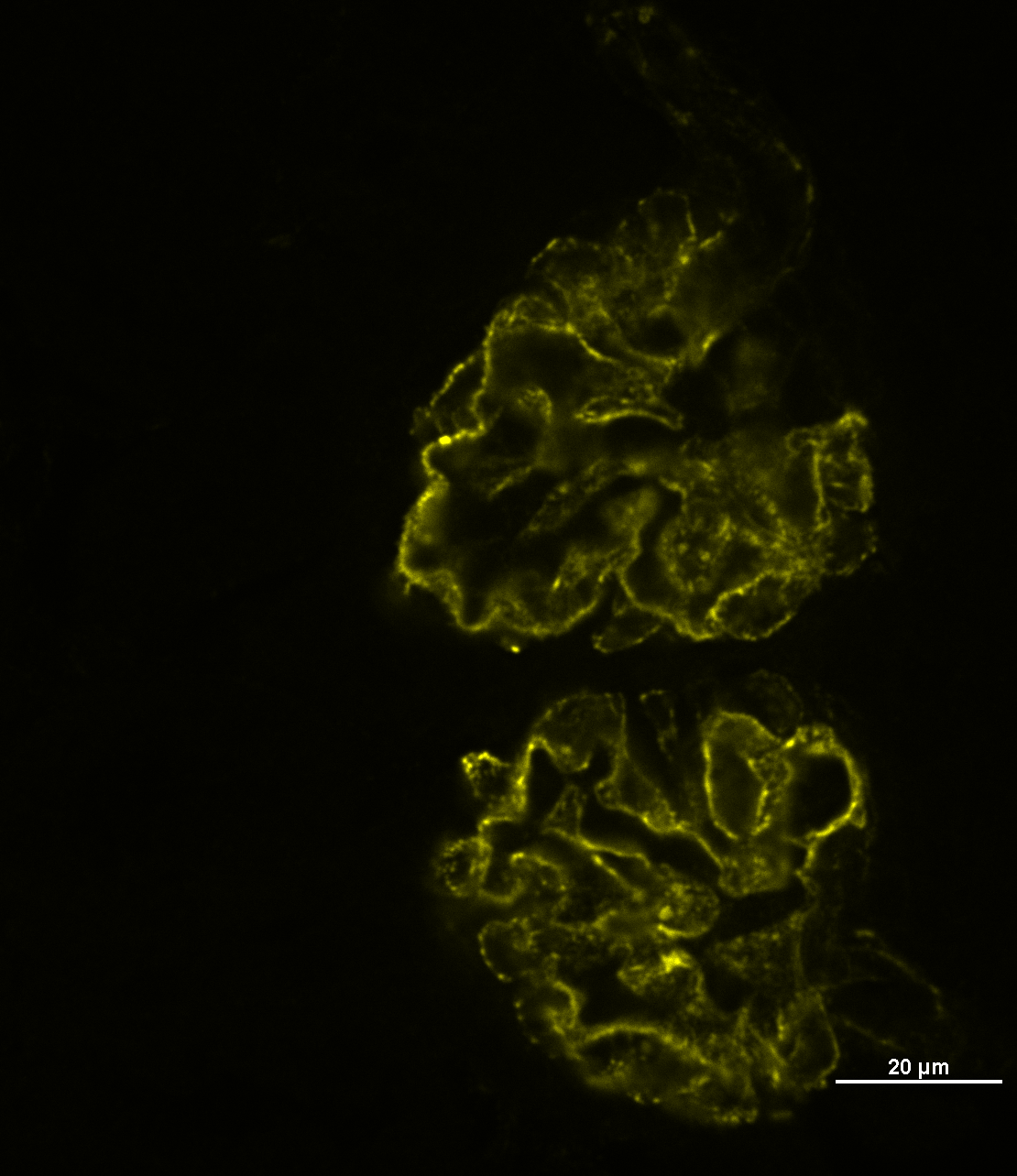

Supplement: Supplementary file 9 — Source Data Fig. 3 [file 44319_2023_19_MOESM9_ESM.zip › Fig.3/3D/Cep120-KO-Desmin_aSMA_SYNPO_RGB_640-SD.tif]

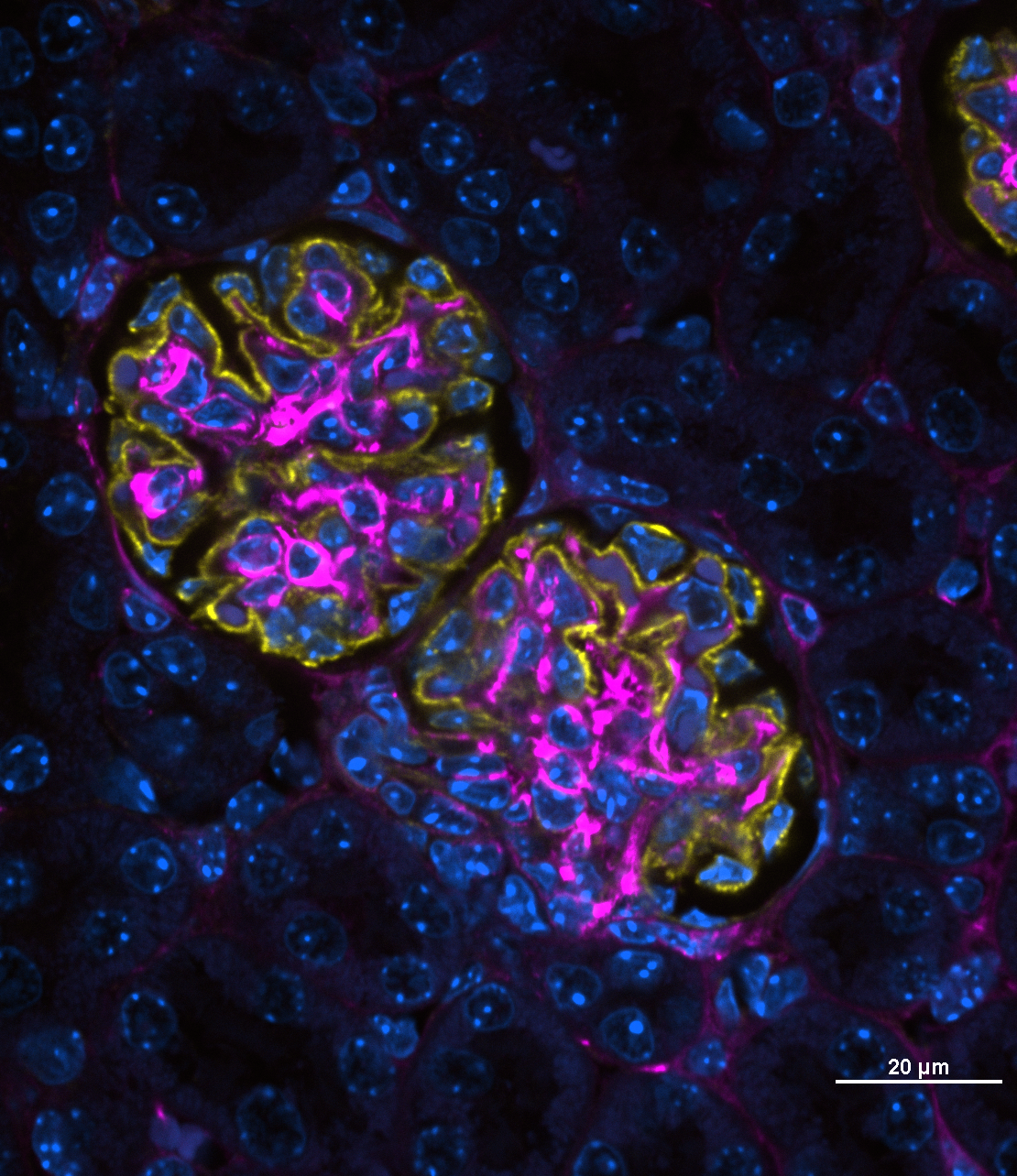

Supplement: Supplementary file 9 — Source Data Fig. 3 [file 44319_2023_19_MOESM9_ESM.zip › Fig.3/3D/Ctrl-Desmin_aSMA_SYNPO_RGB.tif]

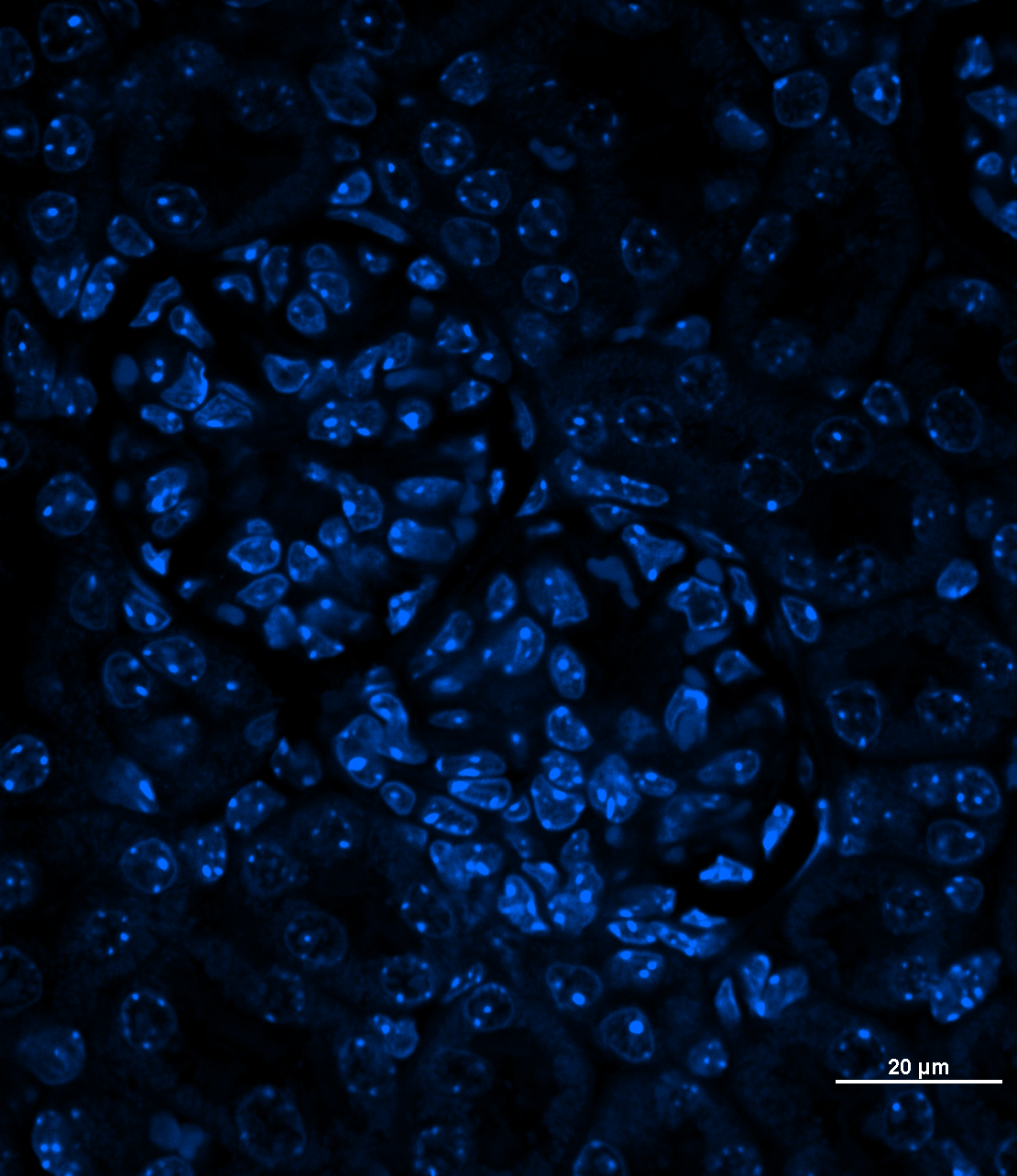

Supplement: Supplementary file 9 — Source Data Fig. 3 [file 44319_2023_19_MOESM9_ESM.zip › Fig.3/3D/Ctrl-Desmin_aSMA_SYNPO_RGB_405-SD .tif]

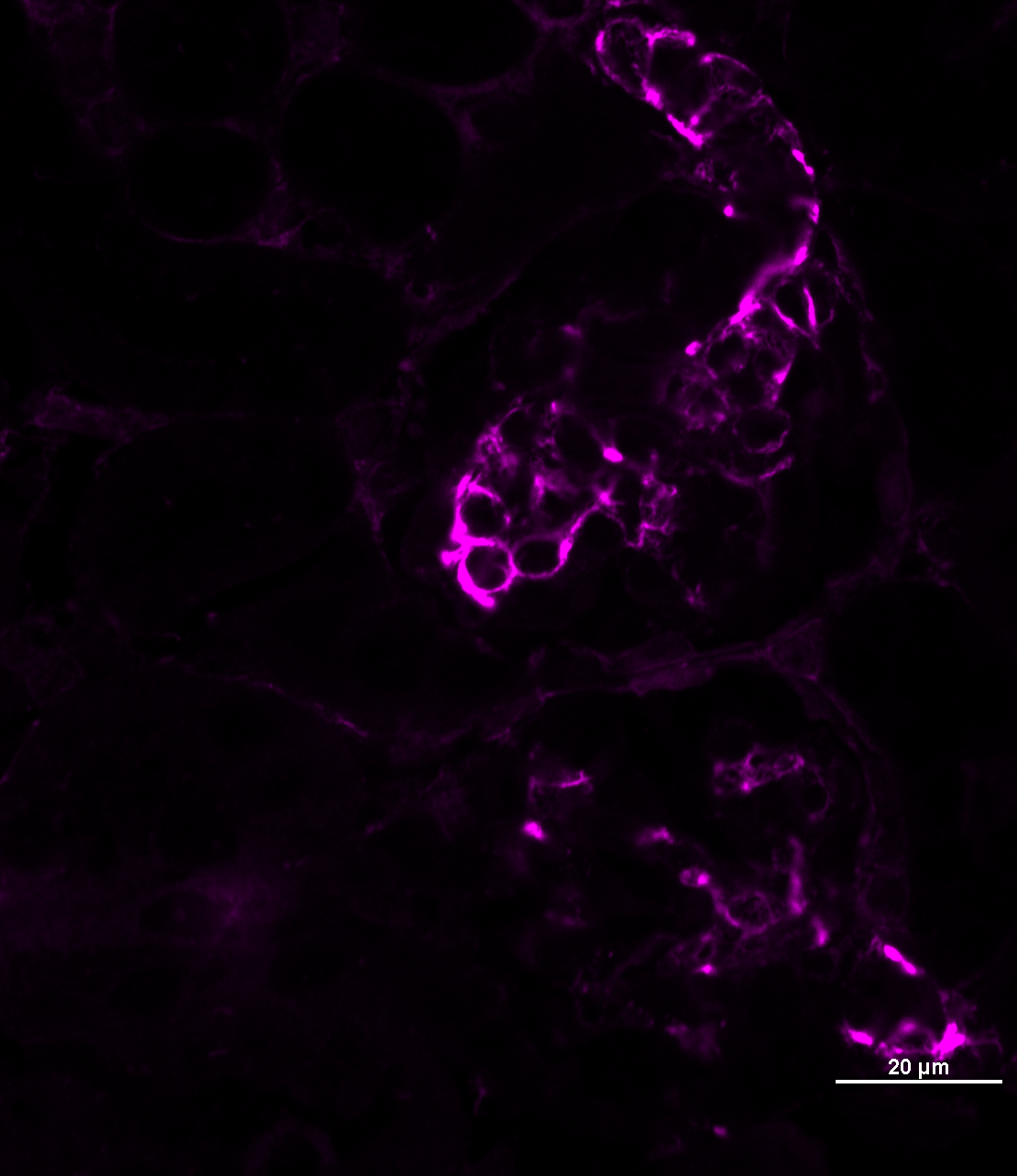

Supplement: Supplementary file 9 — Source Data Fig. 3 [file 44319_2023_19_MOESM9_ESM.zip › Fig.3/3D/Cep120-KO-Desmin_aSMA_SYNPO_RGB_488-SD.tif]

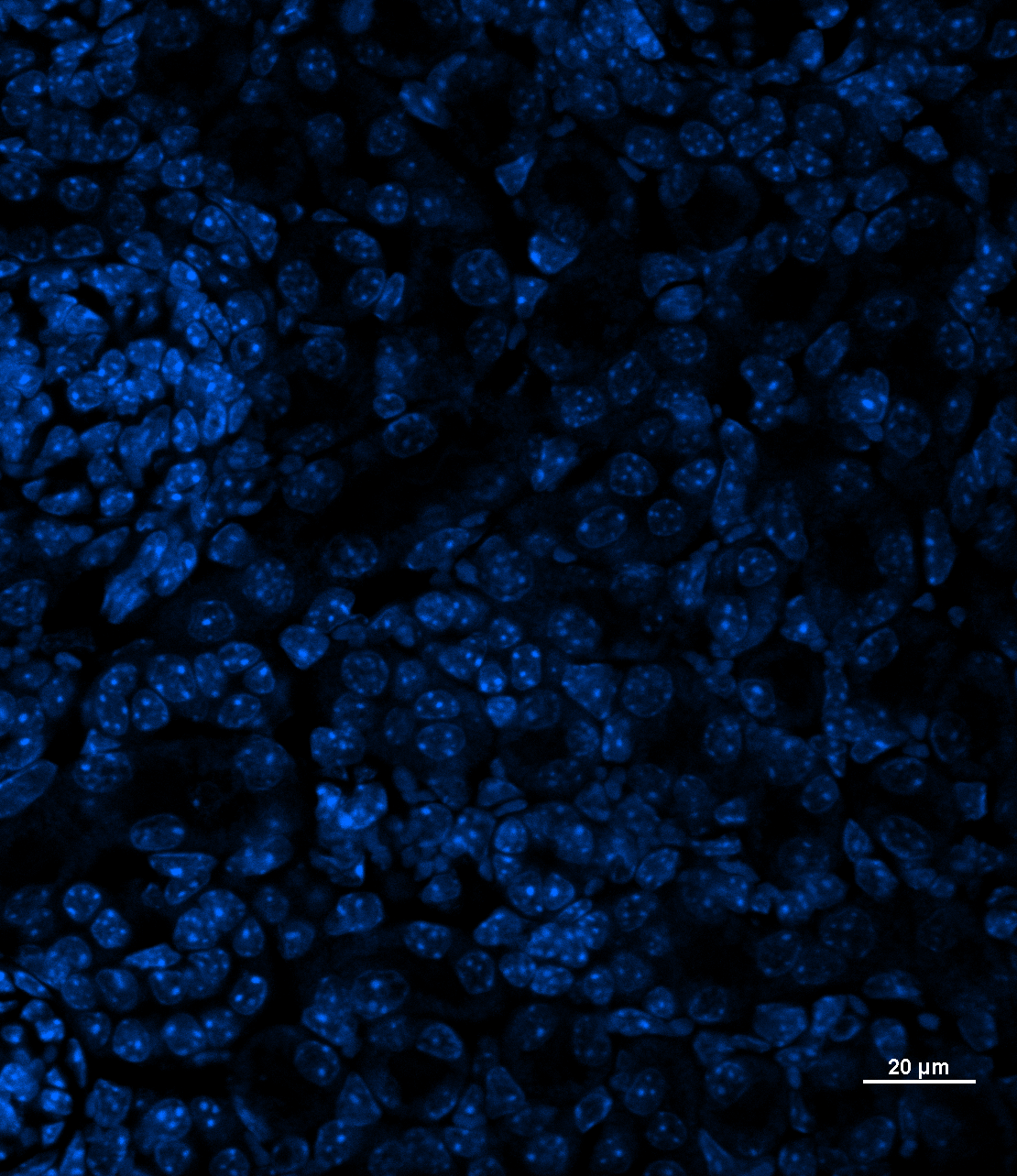

Supplement: Supplementary file 9 — Source Data Fig. 3 [file 44319_2023_19_MOESM9_ESM.zip › Fig.3/3A/Cep120_KO_PDGFRb_Desmin__aSMA-MaxIP_RGB_405-SD .tif]

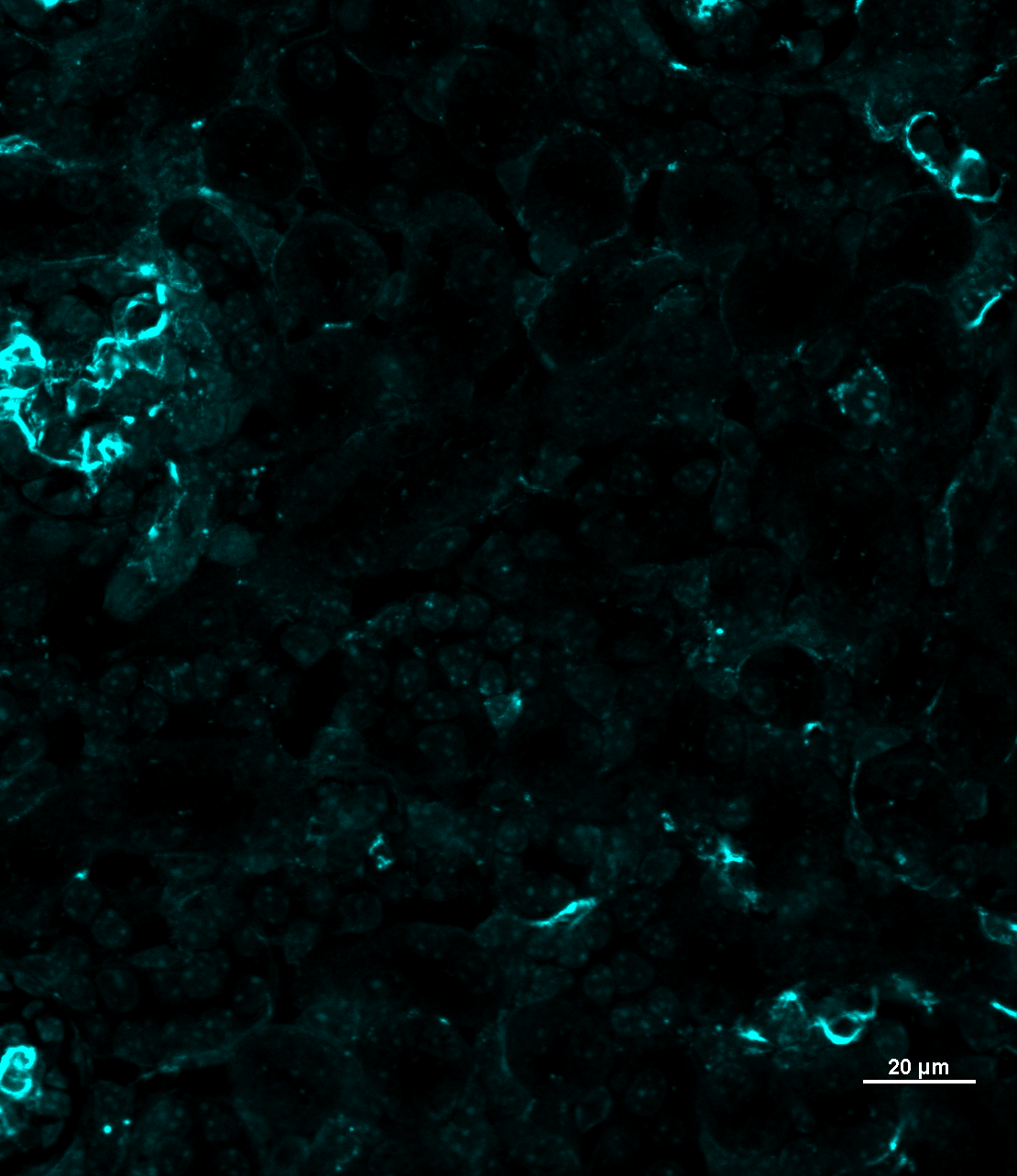

Supplement: Supplementary file 9 — Source Data Fig. 3 [file 44319_2023_19_MOESM9_ESM.zip › Fig.3/3A/Cep120_KO_PDGFRb_Desmin__aSMA-MaxIP_RGB_488-SD.tif]

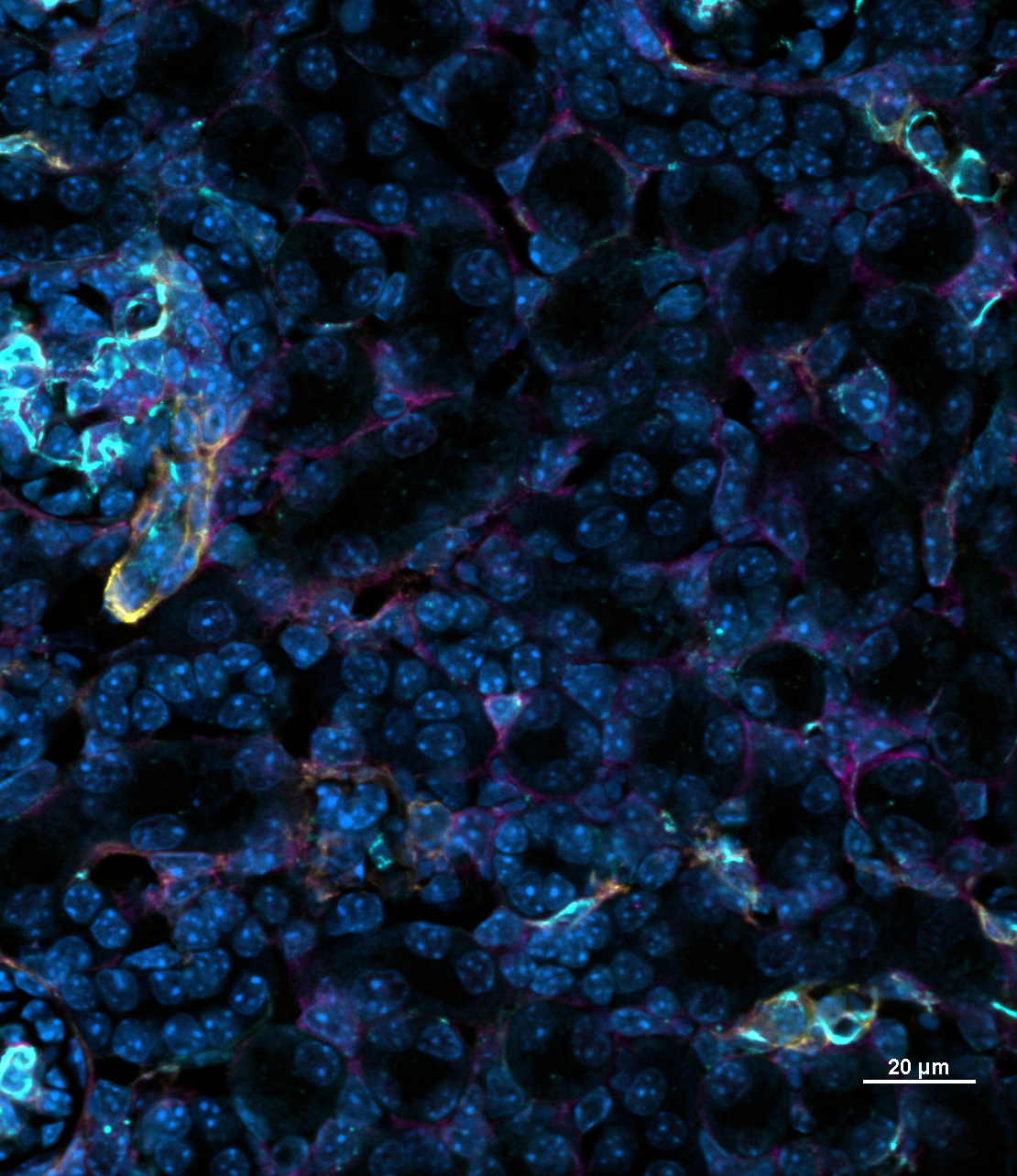

Supplement: Supplementary file 9 — Source Data Fig. 3 [file 44319_2023_19_MOESM9_ESM.zip › Fig.3/3A/Cep120_KO_PDGFRb_Desmin__aSMA-MaxIP_RGB.tif]

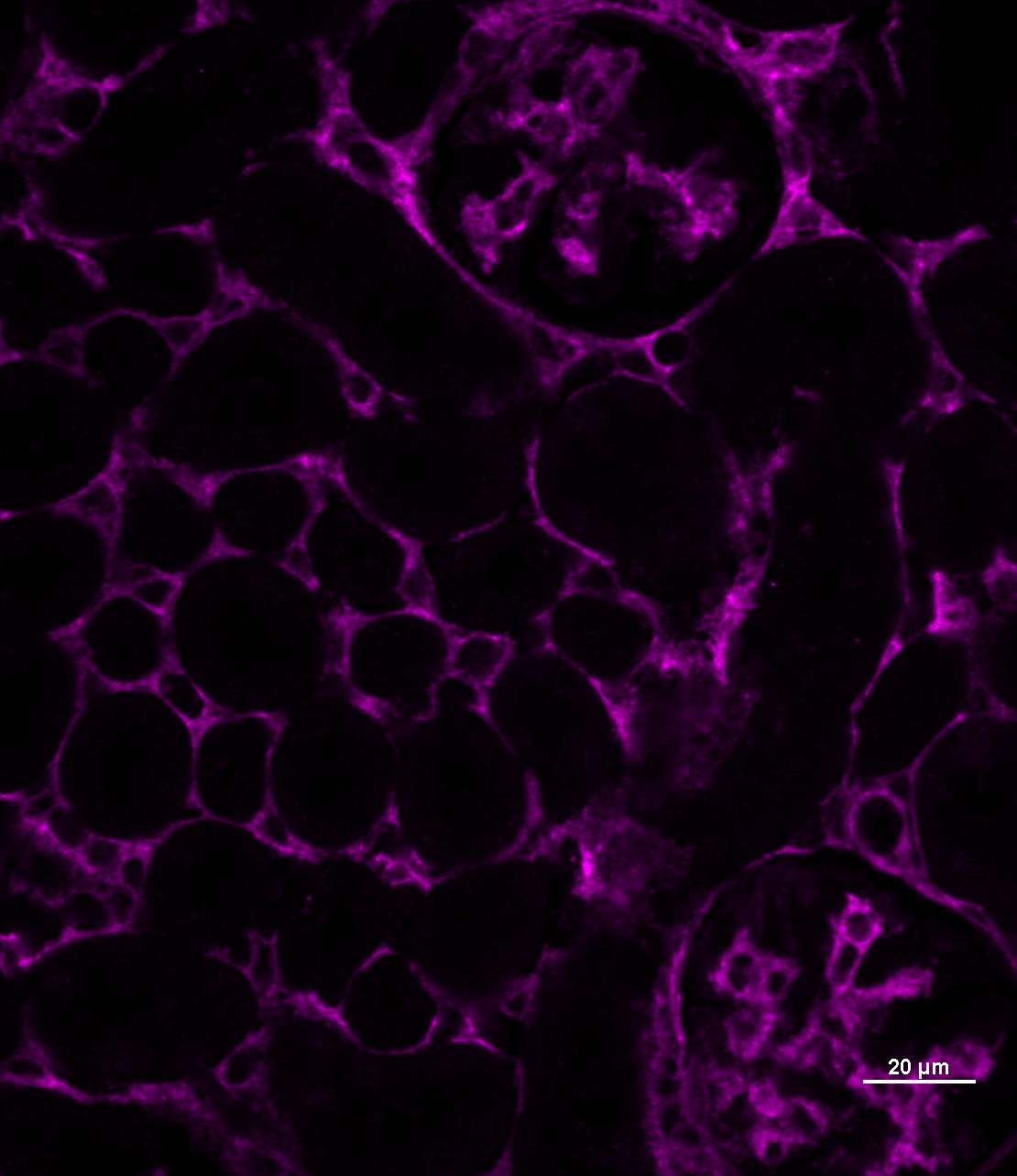

Supplement: Supplementary file 9 — Source Data Fig. 3 [file 44319_2023_19_MOESM9_ESM.zip › Fig.3/3A/Ctrl-_PDGFRb_Desmin__aSMA-MaxIP_RGB_640-SD.tif]

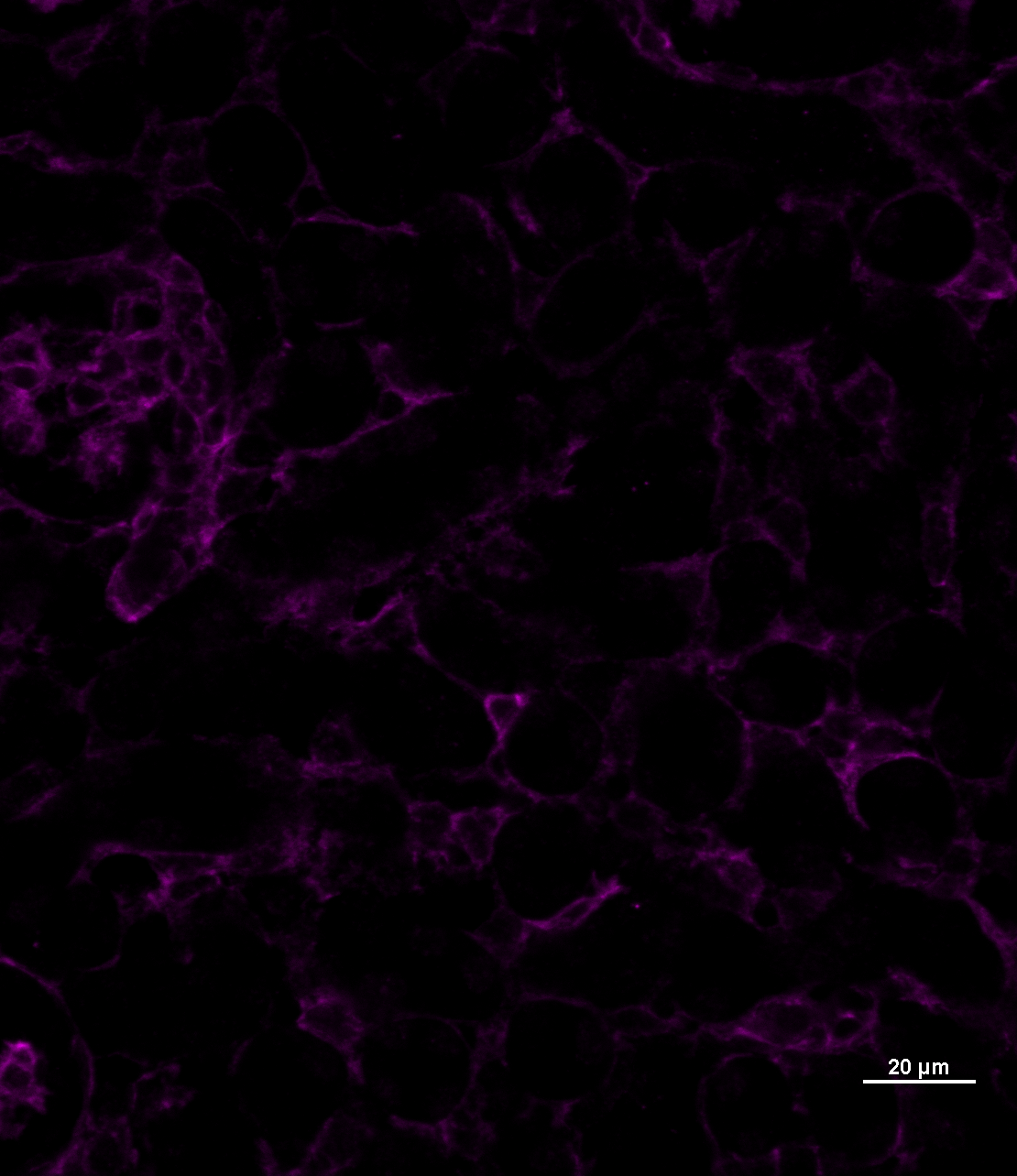

Supplement: Supplementary file 9 — Source Data Fig. 3 [file 44319_2023_19_MOESM9_ESM.zip › Fig.3/3A/Cep120_KO_PDGFRb_Desmin__aSMA-MaxIP_RGB_640-SD.tif]

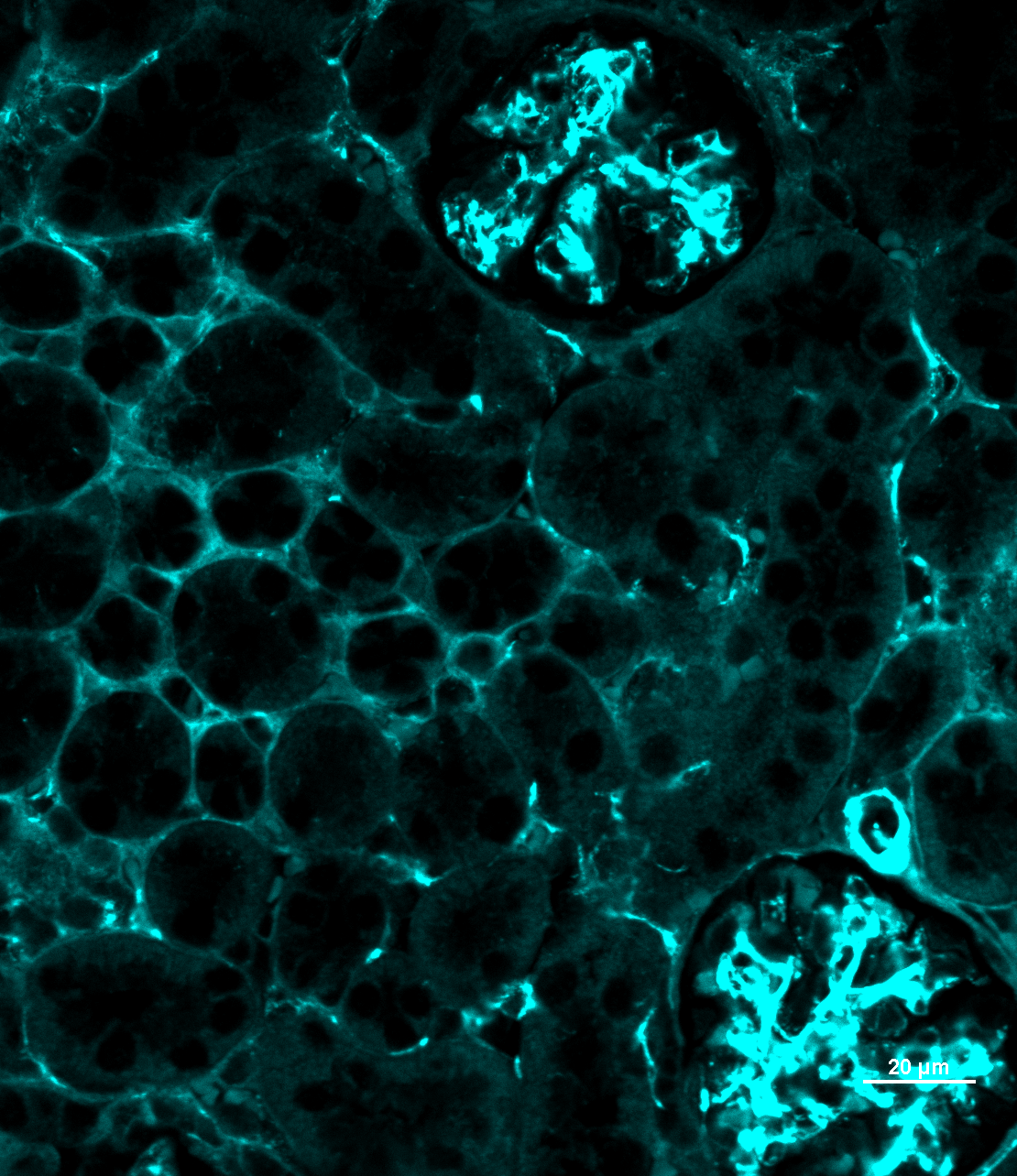

Supplement: Supplementary file 9 — Source Data Fig. 3 [file 44319_2023_19_MOESM9_ESM.zip › Fig.3/3A/Ctrl-_PDGFRb_Desmin__aSMA-MaxIP_RGB_488-SD.tif]

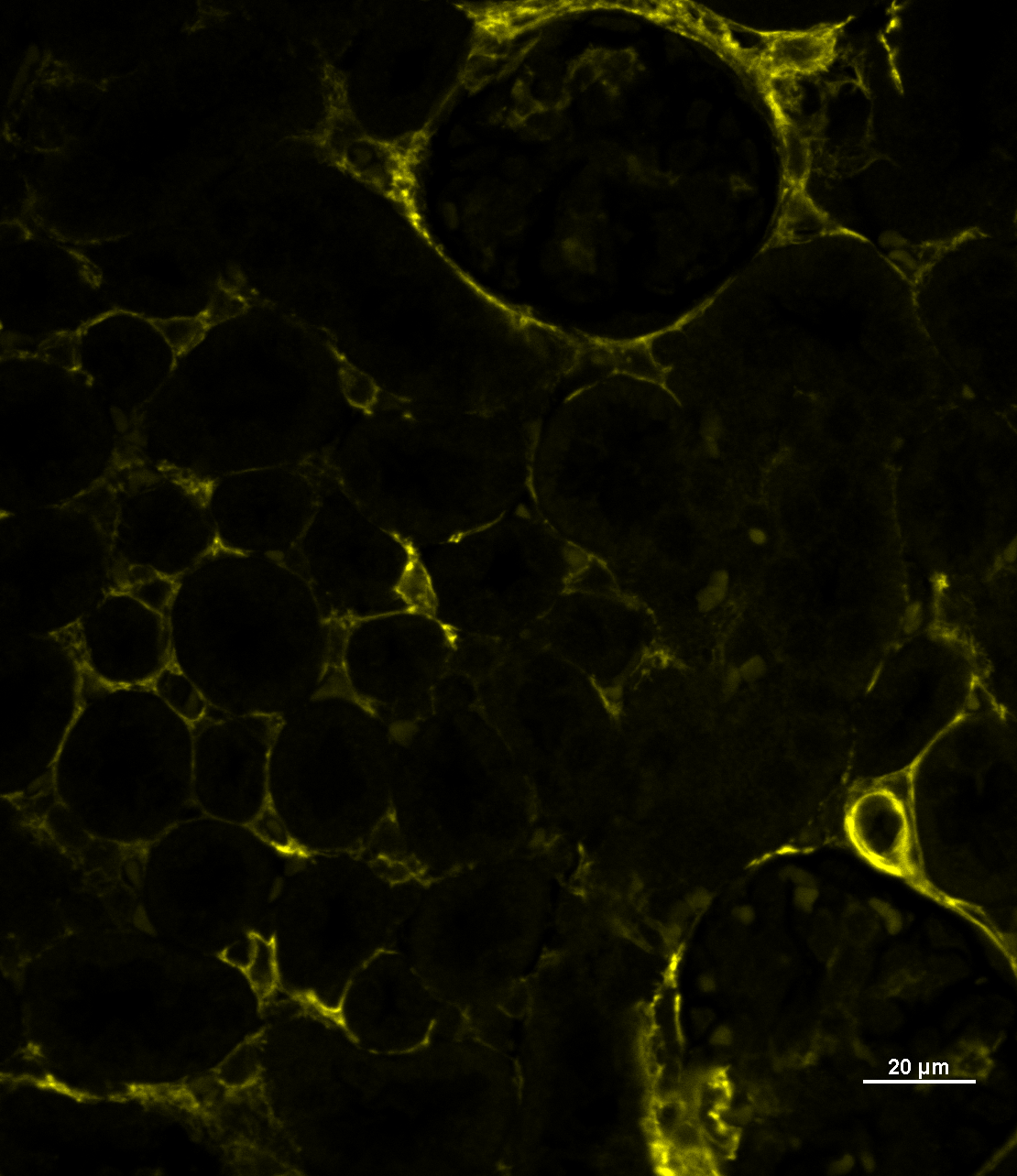

Supplement: Supplementary file 9 — Source Data Fig. 3 [file 44319_2023_19_MOESM9_ESM.zip › Fig.3/3A/Ctrl-_PDGFRb_Desmin__aSMA-MaxIP_RGB_561-SD.tif]

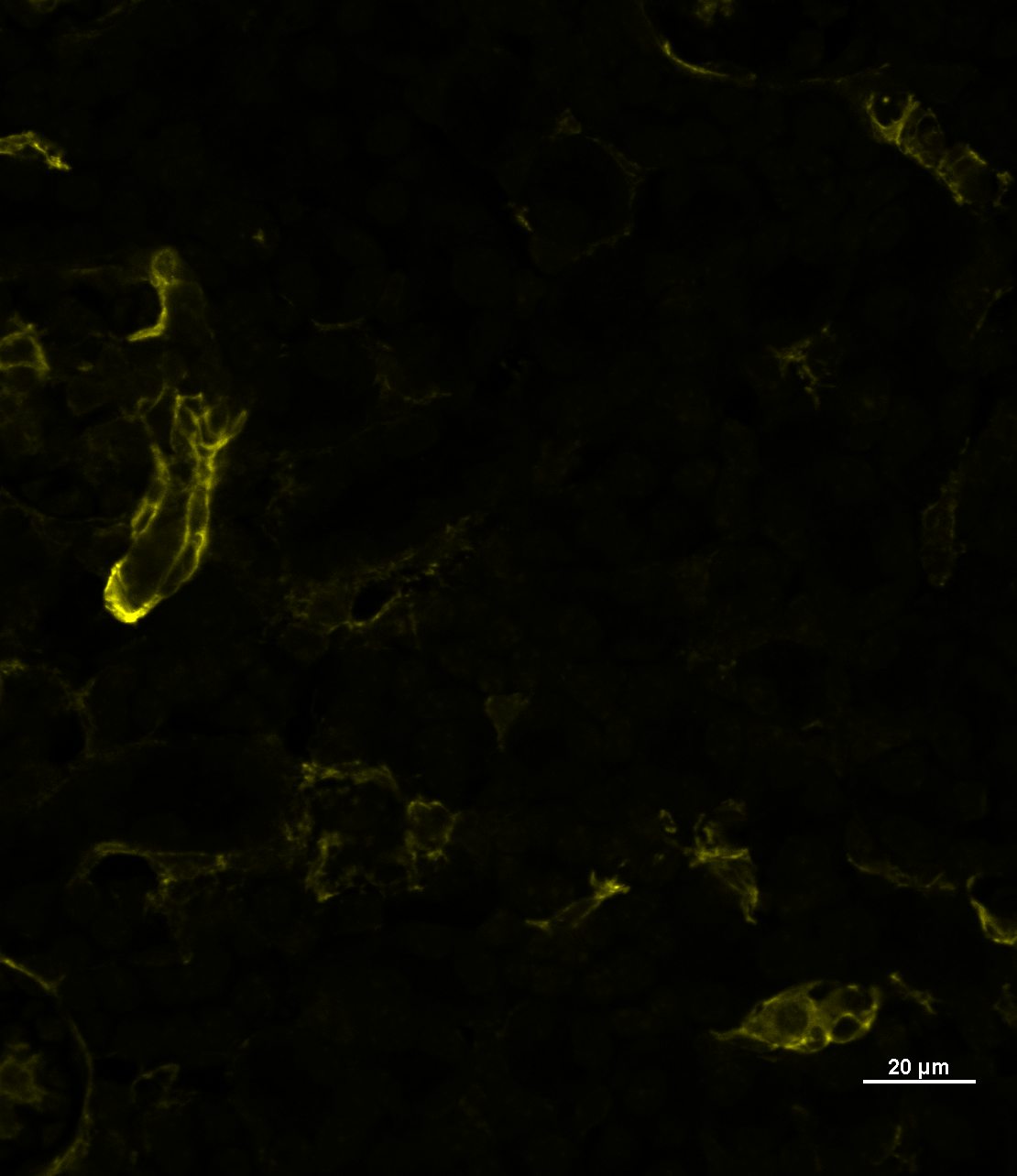

Supplement: Supplementary file 9 — Source Data Fig. 3 [file 44319_2023_19_MOESM9_ESM.zip › Fig.3/3A/Cep120_KO_PDGFRb_Desmin__aSMA-MaxIP_RGB_561-SD.tif]

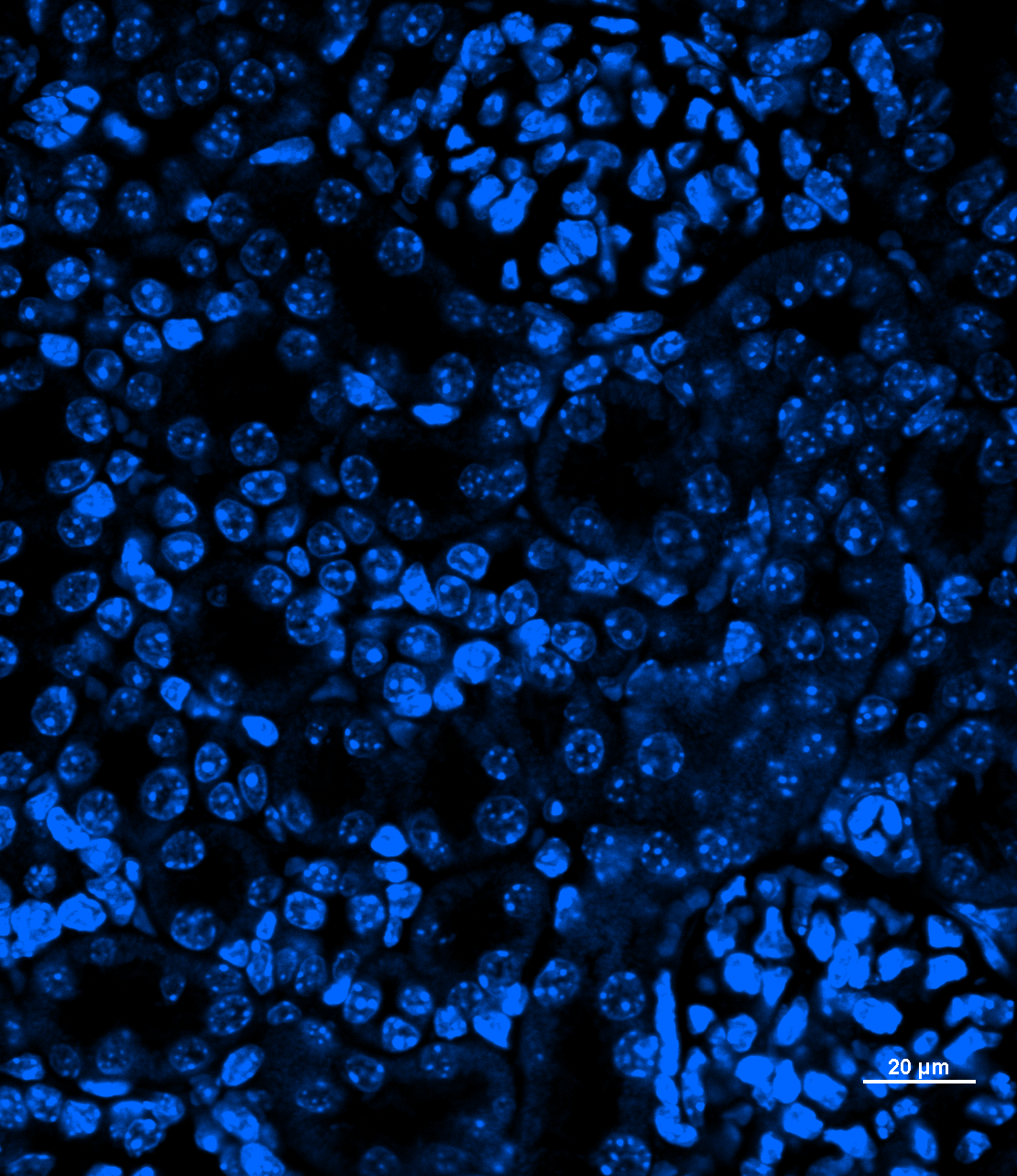

Supplement: Supplementary file 9 — Source Data Fig. 3 [file 44319_2023_19_MOESM9_ESM.zip › Fig.3/3A/Ctrl-_PDGFRb_Desmin__aSMA-MaxIP_RGB_405-SD .tif]

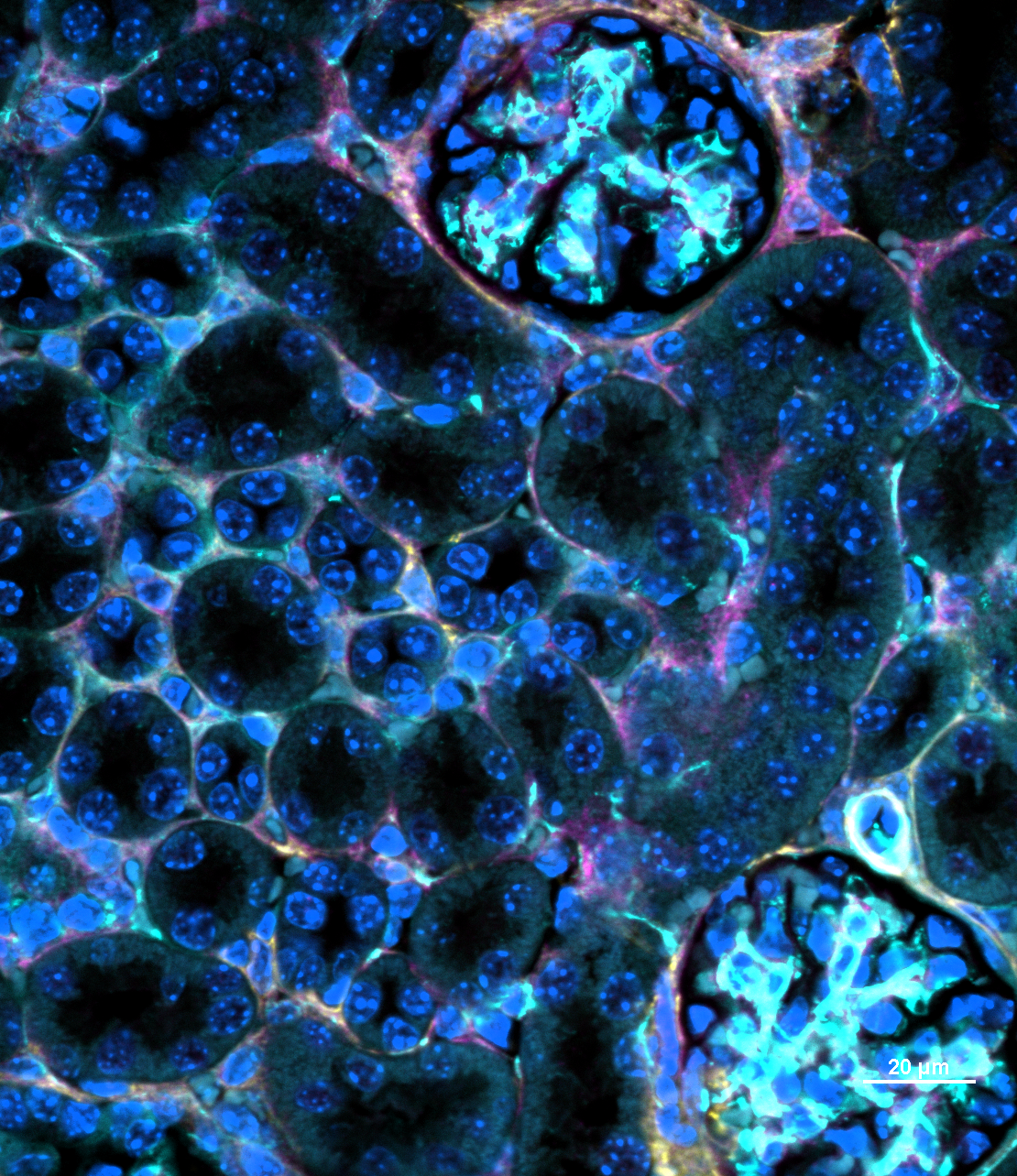

Supplement: Supplementary file 9 — Source Data Fig. 3 [file 44319_2023_19_MOESM9_ESM.zip › Fig.3/3A/Ctrl-_PDGFRb_Desmin__aSMA-MaxIP_RGB.tif]

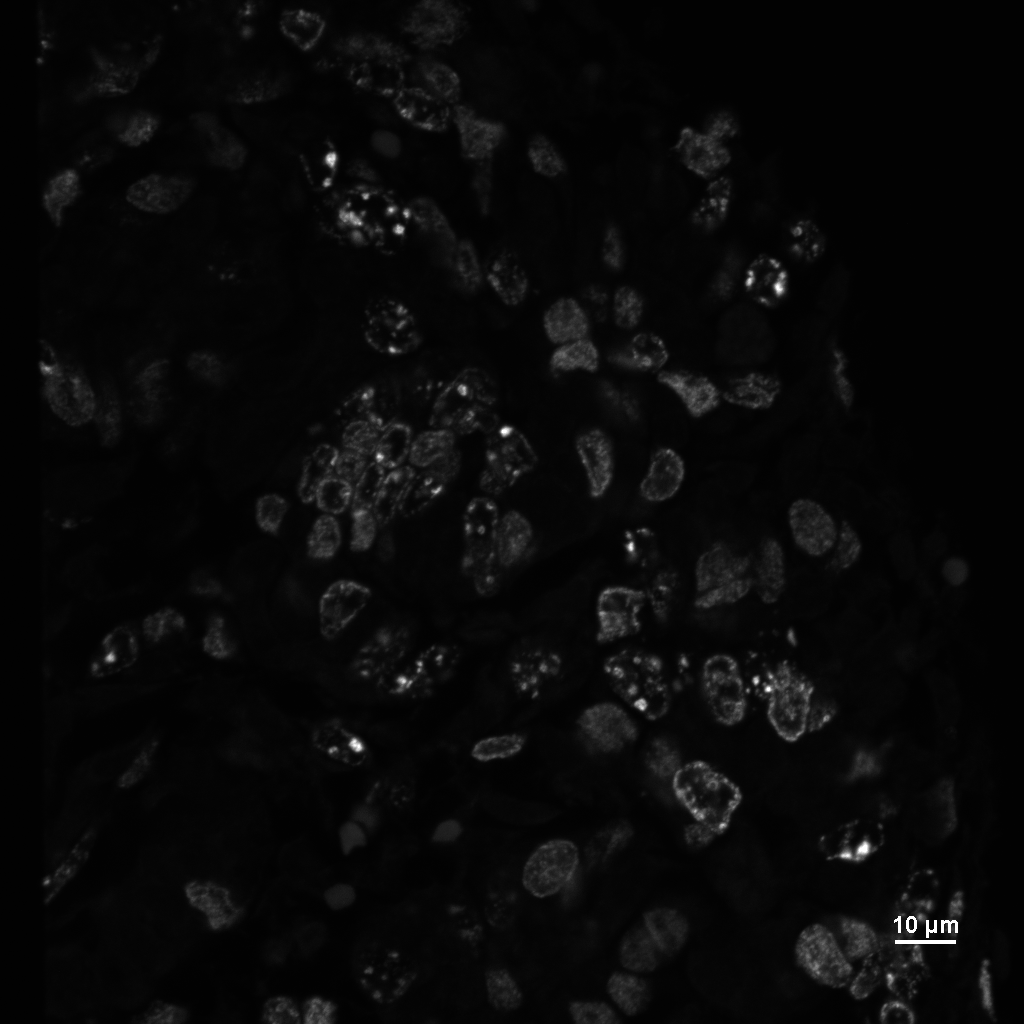

Supplement: Supplementary file 10 — Source Data Fig. 4 [file 44319_2023_19_MOESM10_ESM.zip › Fig.4/4C/Cep120E15.5-KO-Aldh1_EdU_pHH3-MaxIP_RGB_561-SD.tif]

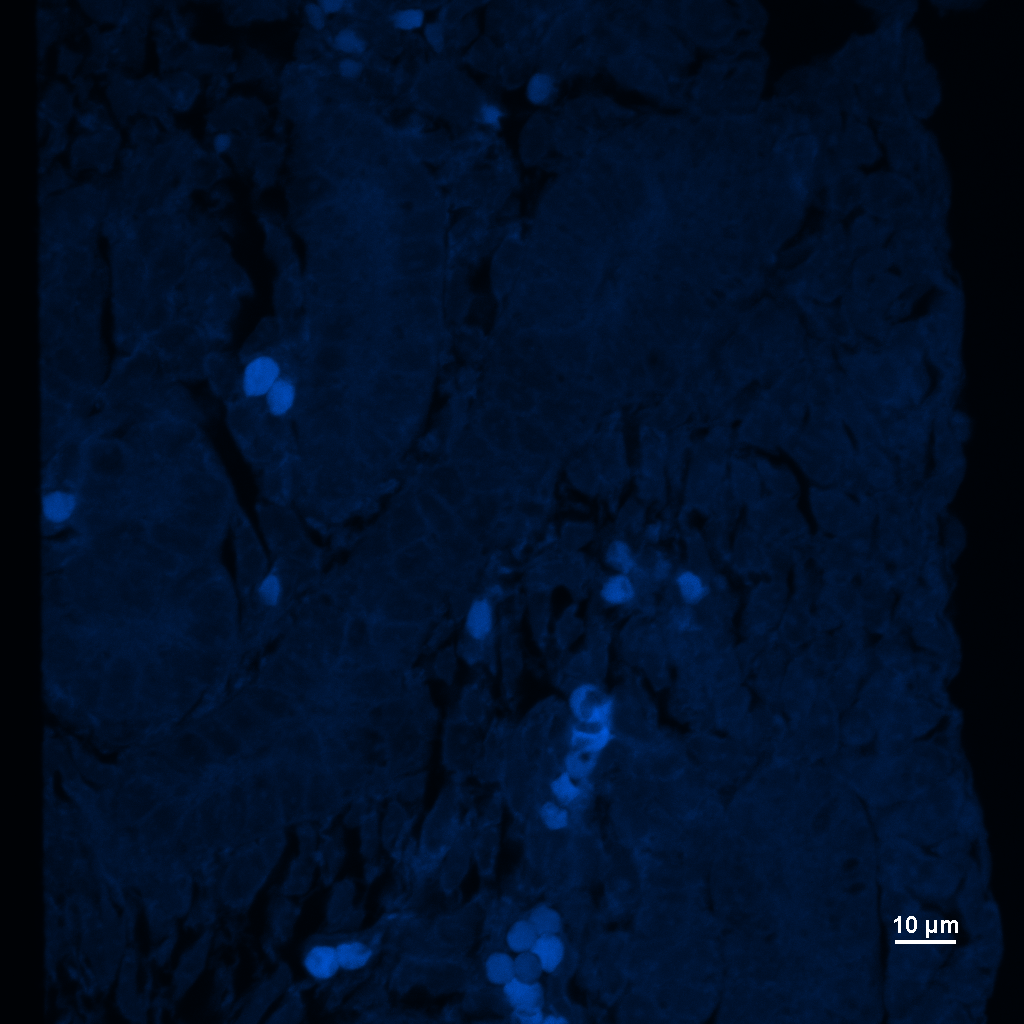

Supplement: Supplementary file 10 — Source Data Fig. 4 [file 44319_2023_19_MOESM10_ESM.zip › Fig.4/4C/Ctrl-Aldh1_EdU_pHH3-MaxIP_RGB_405-SD .tif]

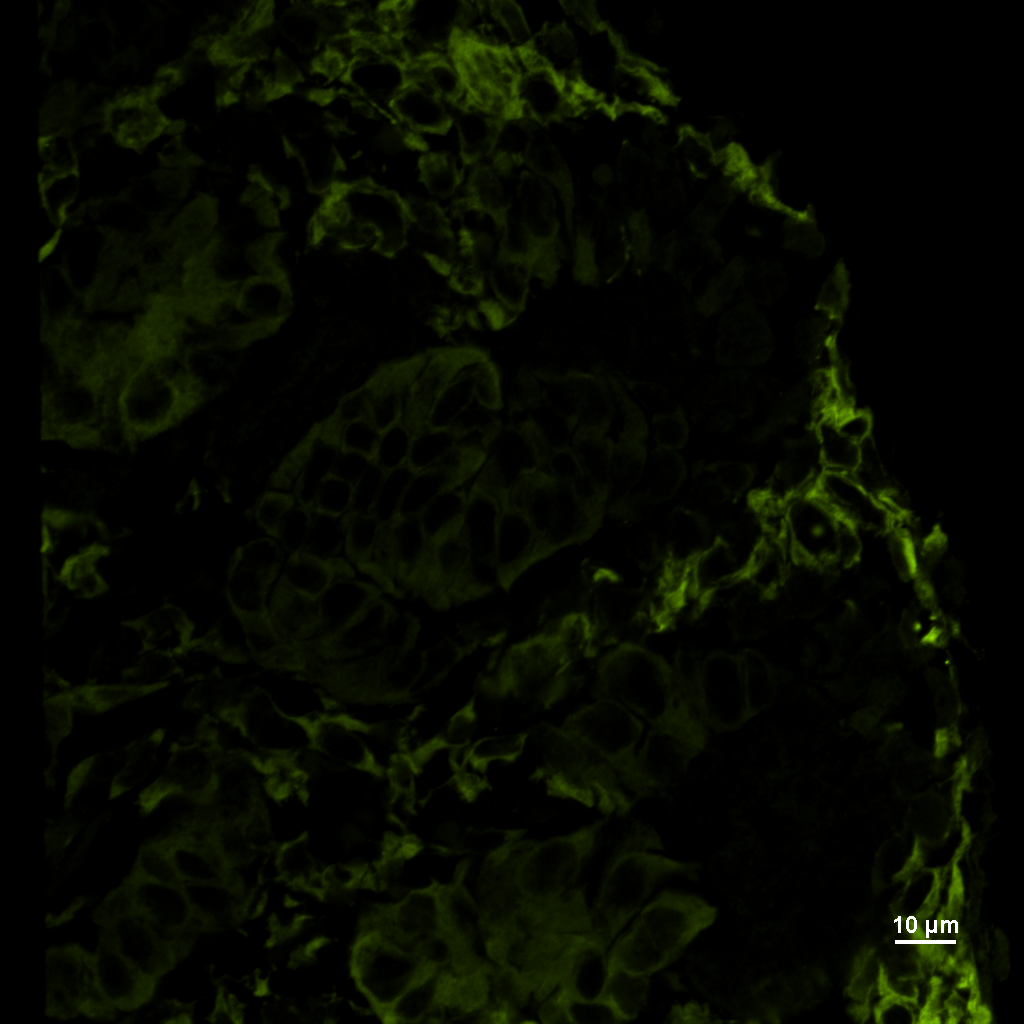

Supplement: Supplementary file 10 — Source Data Fig. 4 [file 44319_2023_19_MOESM10_ESM.zip › Fig.4/4C/Cep120E15.5-KO-Aldh1_EdU_pHH3-MaxIP_RGB_488-SD.tif]

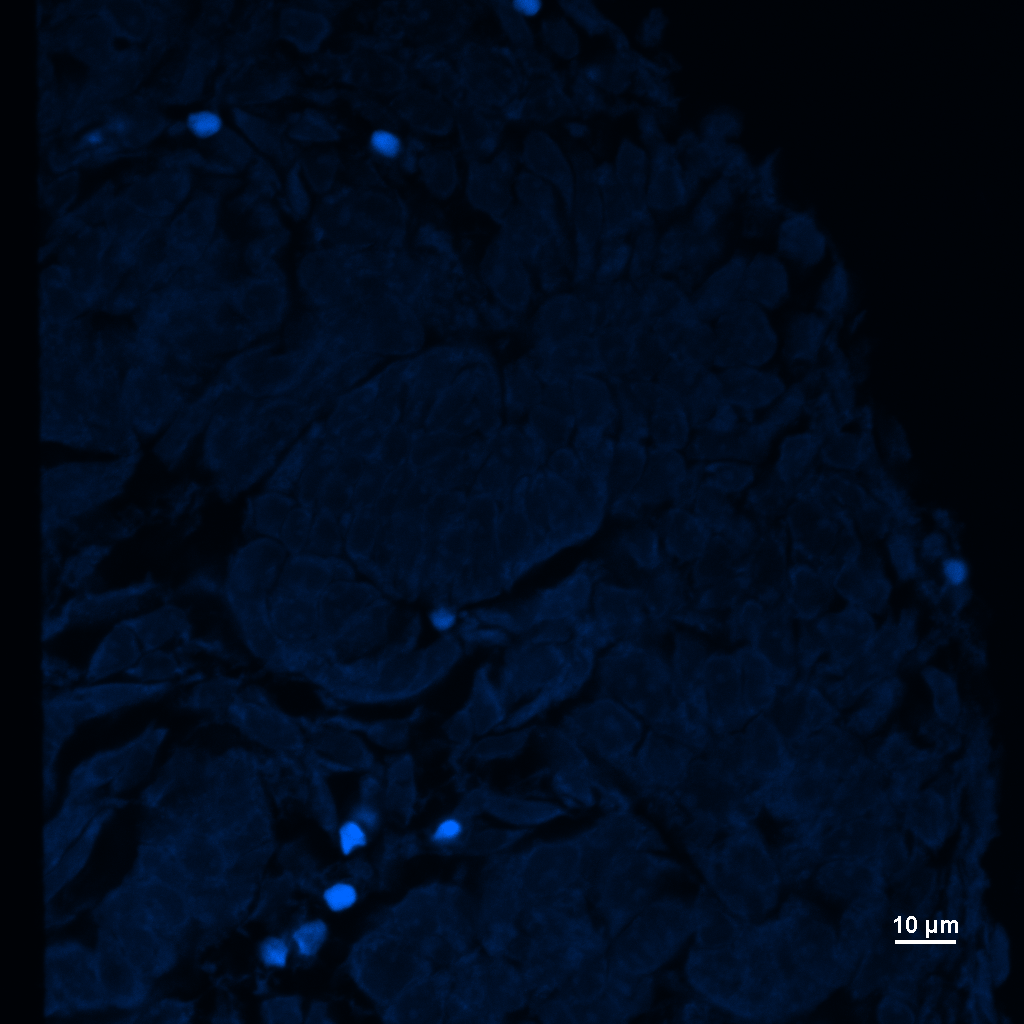

Supplement: Supplementary file 10 — Source Data Fig. 4 [file 44319_2023_19_MOESM10_ESM.zip › Fig.4/4C/Cep120E15.5-KO-Aldh1_EdU_pHH3-MaxIP_RGB_405-SD .tif]

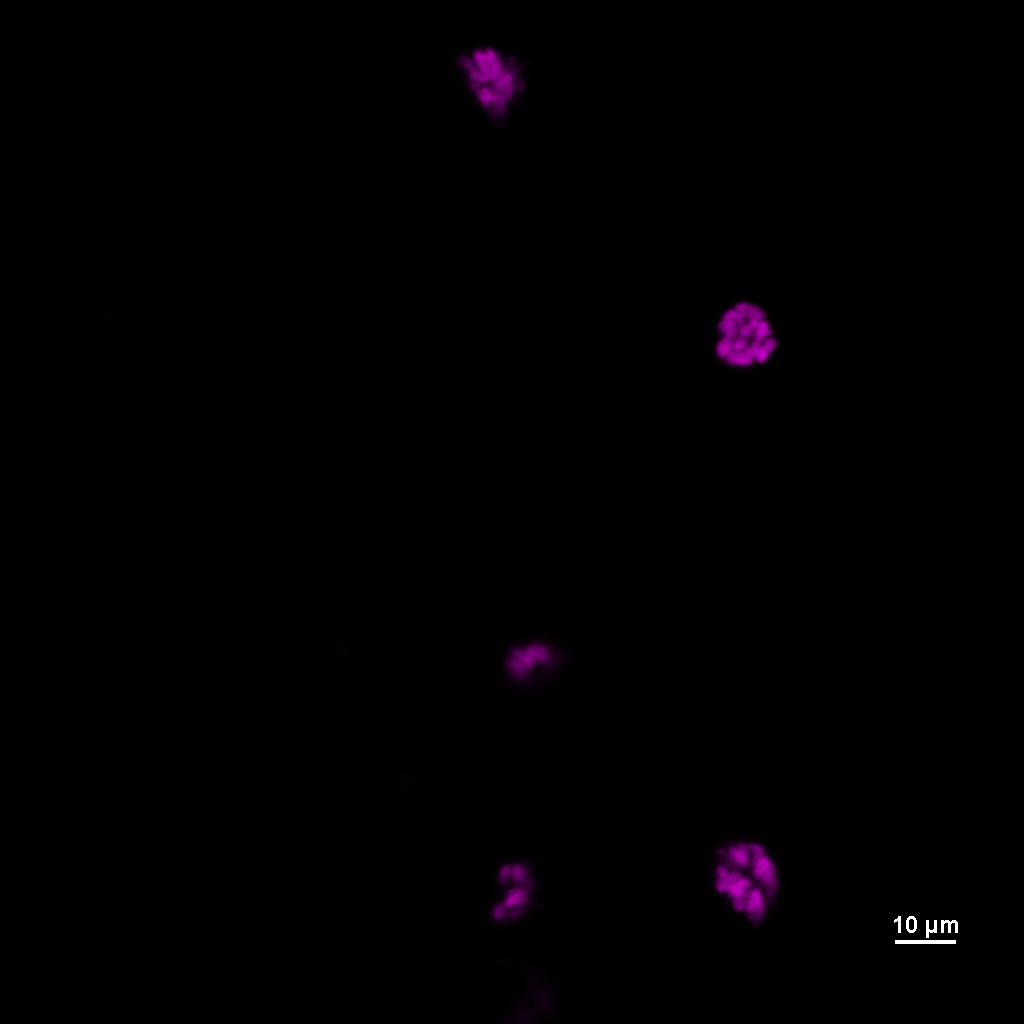

Supplement: Supplementary file 10 — Source Data Fig. 4 [file 44319_2023_19_MOESM10_ESM.zip › Fig.4/4C/Cep120E15.5-KO-Aldh1_EdU_pHH3-MaxIP_RGB_640-SD.tif]

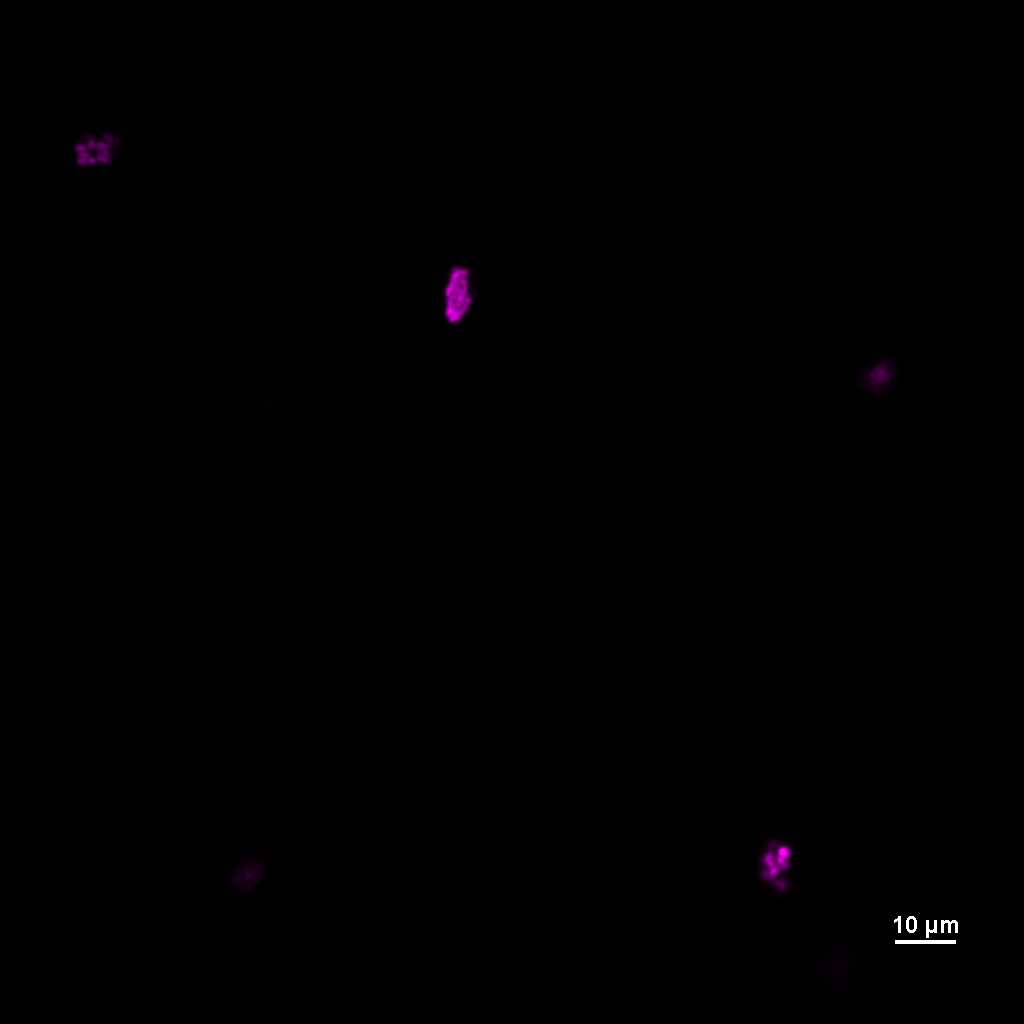

Supplement: Supplementary file 10 — Source Data Fig. 4 [file 44319_2023_19_MOESM10_ESM.zip › Fig.4/4C/Ctrl-Aldh1_EdU_pHH3-MaxIP_RGB_640-SD.tif]

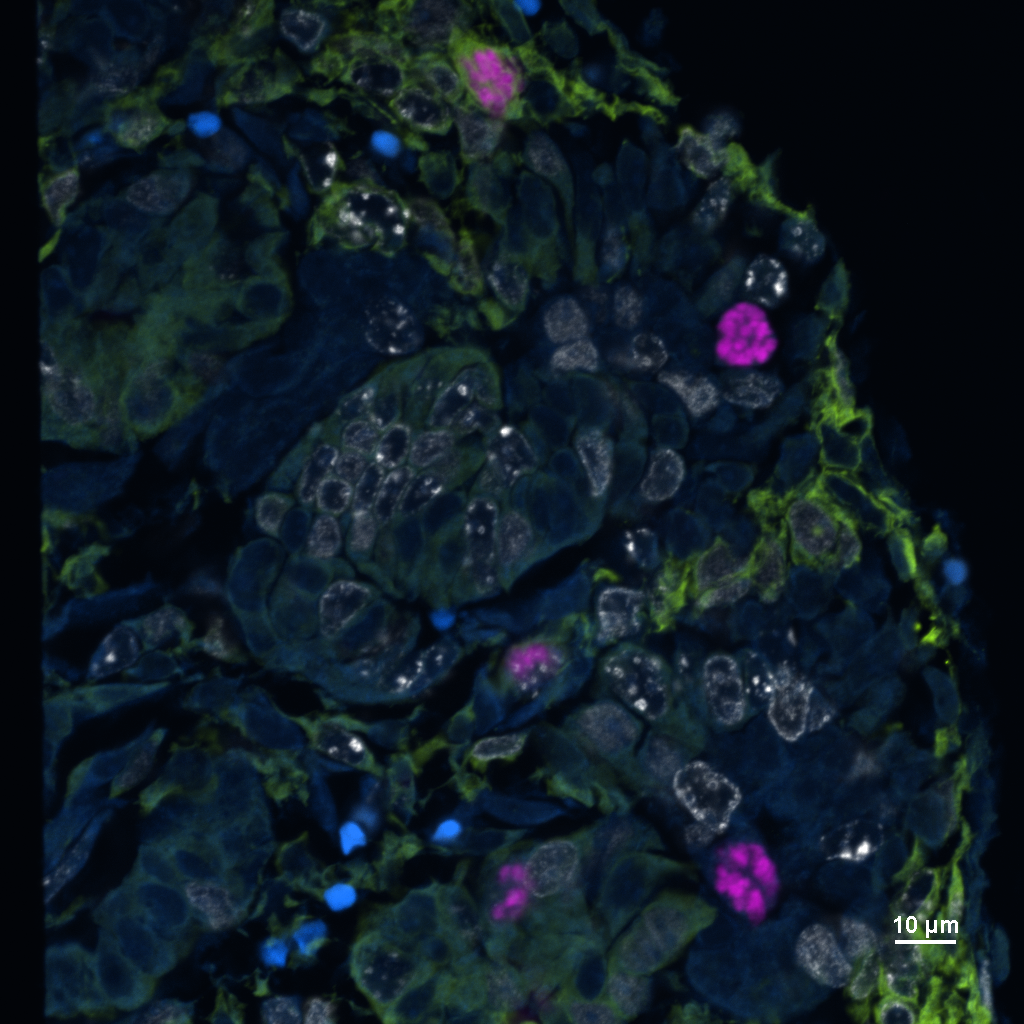

Supplement: Supplementary file 10 — Source Data Fig. 4 [file 44319_2023_19_MOESM10_ESM.zip › Fig.4/4C/Cep120E15.5-KO-Aldh1_EdU_pHH3-MaxIP_RGB.tif]

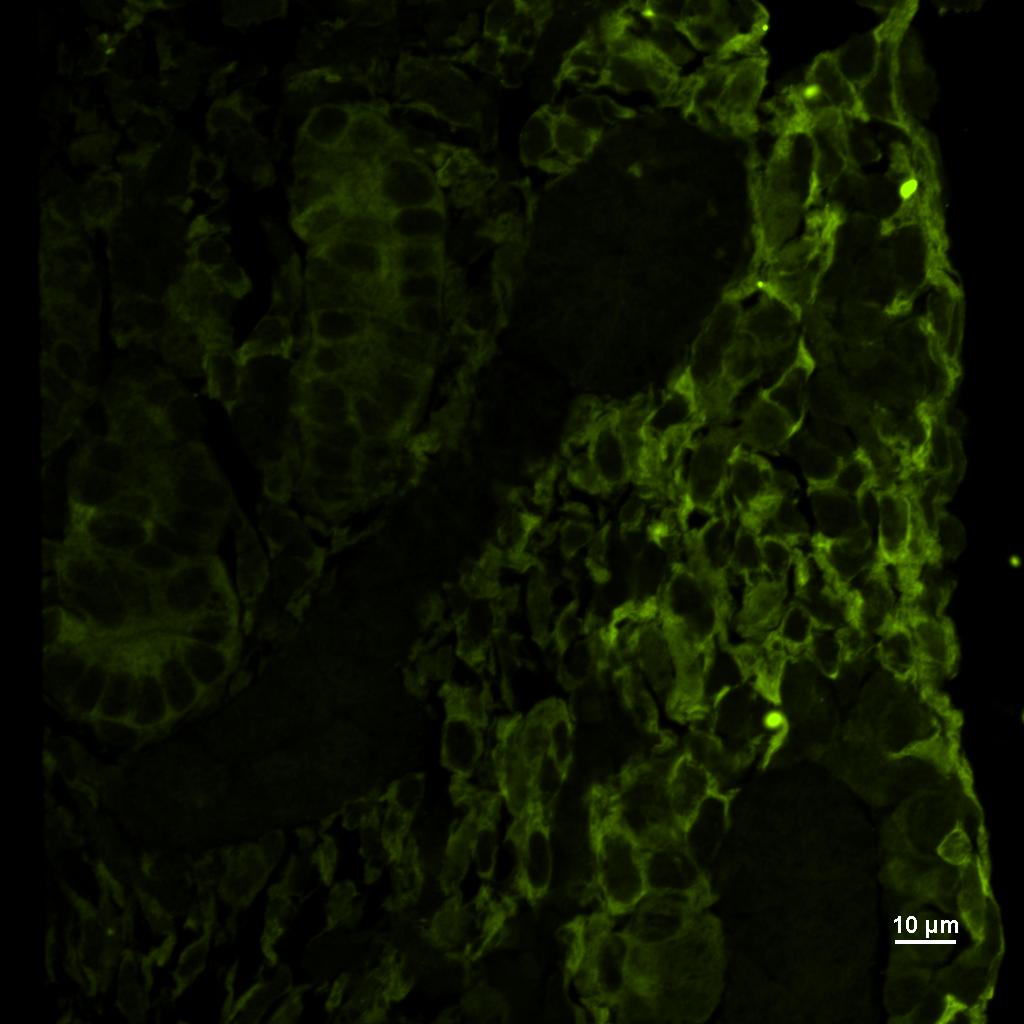

Supplement: Supplementary file 10 — Source Data Fig. 4 [file 44319_2023_19_MOESM10_ESM.zip › Fig.4/4C/Ctrl-Aldh1_EdU_pHH3-MaxIP_RGB_488-SD.tif]

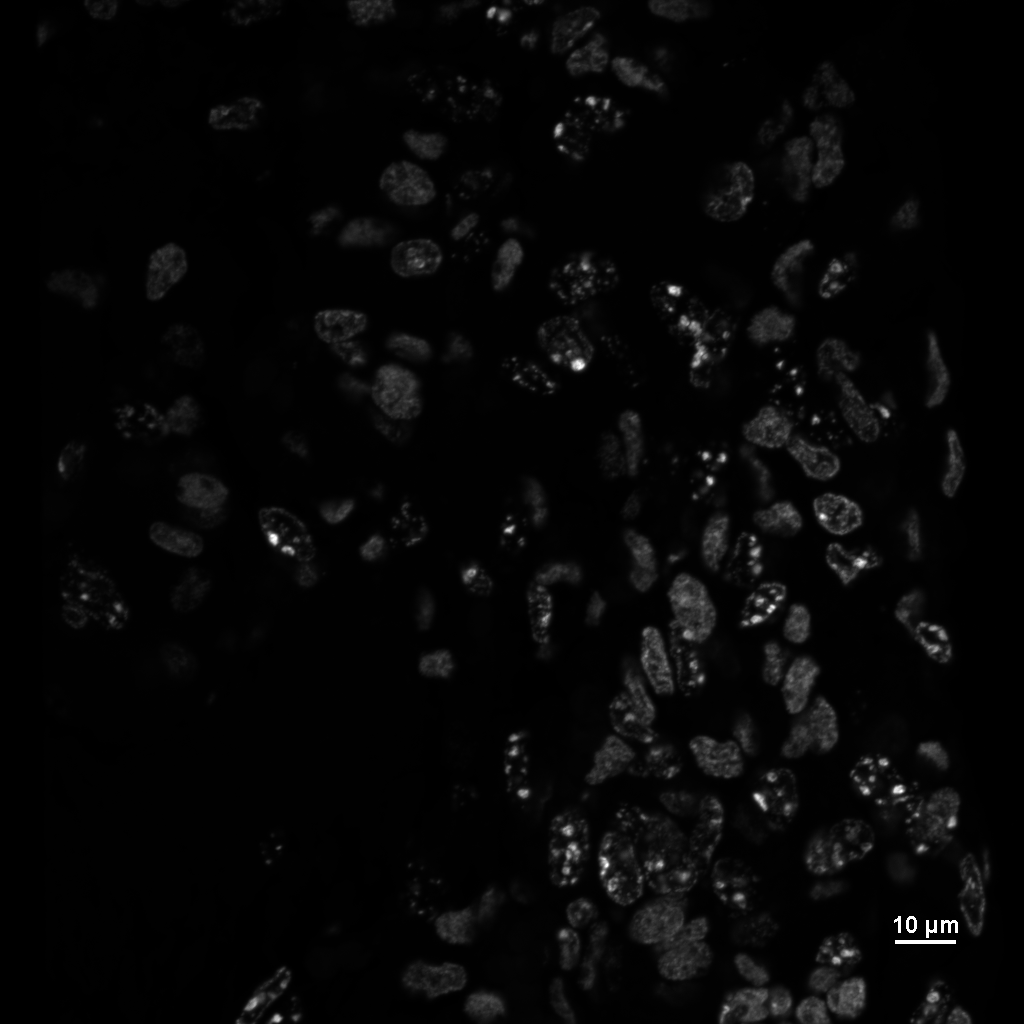

Supplement: Supplementary file 10 — Source Data Fig. 4 [file 44319_2023_19_MOESM10_ESM.zip › Fig.4/4C/Ctrl-Aldh1_EdU_pHH3-MaxIP_RGB_561-SD.tif]

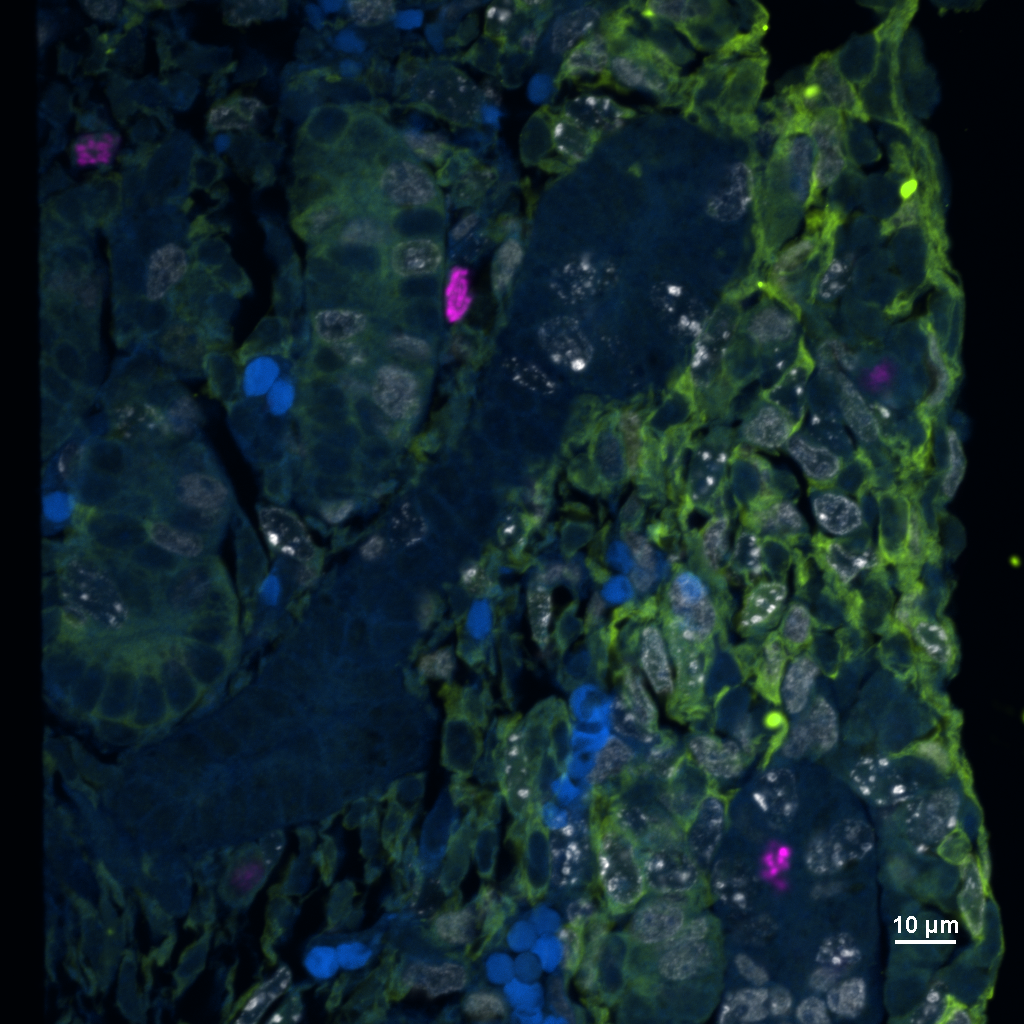

Supplement: Supplementary file 10 — Source Data Fig. 4 [file 44319_2023_19_MOESM10_ESM.zip › Fig.4/4C/Ctrl-Aldh1_EdU_pHH3-MaxIP_RGB.tif]

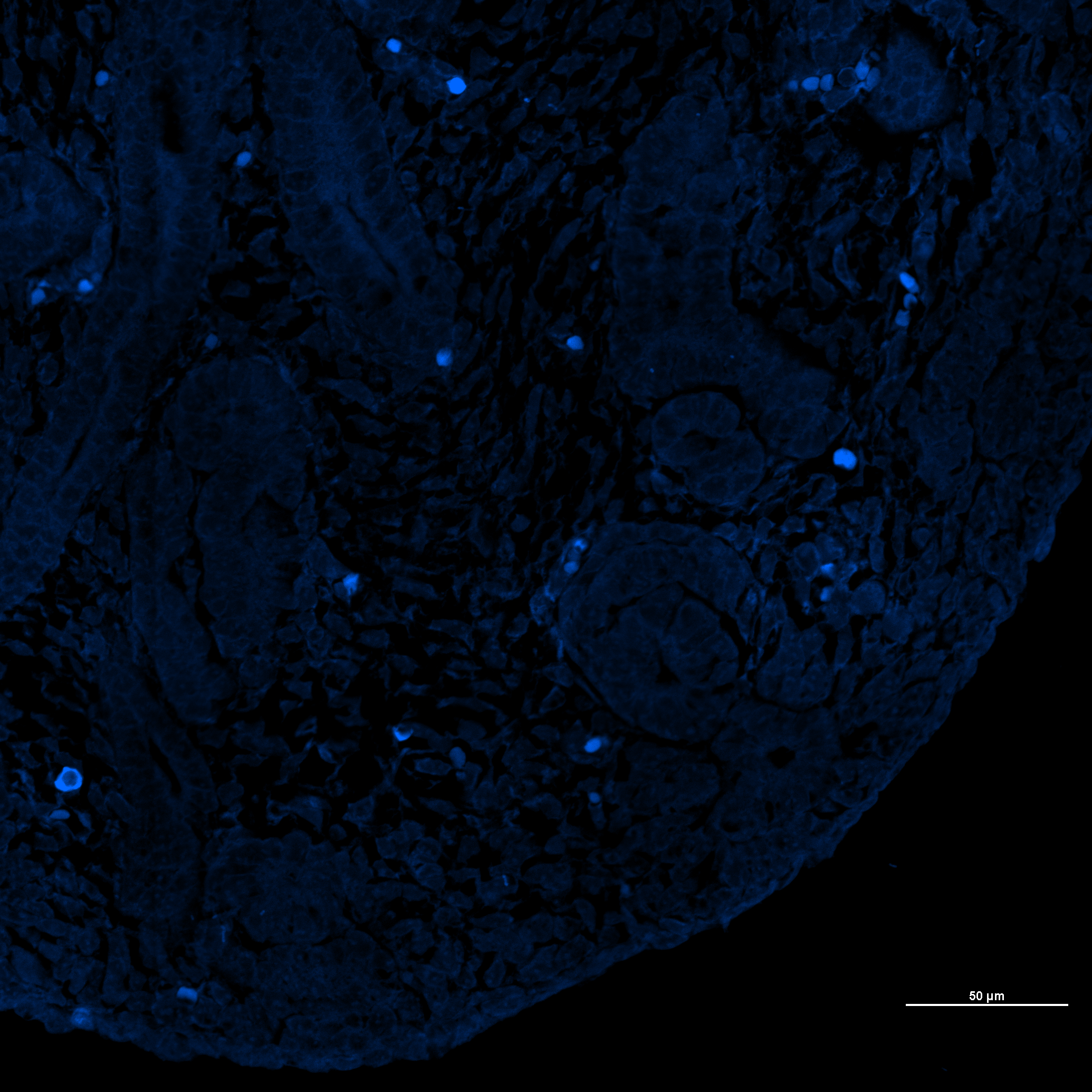

Supplement: Supplementary file 10 — Source Data Fig. 4 [file 44319_2023_19_MOESM10_ESM.zip › Fig.4/4I/Ctrl_Meis1_CC3_E-cad_RGB_405-SD .tif]

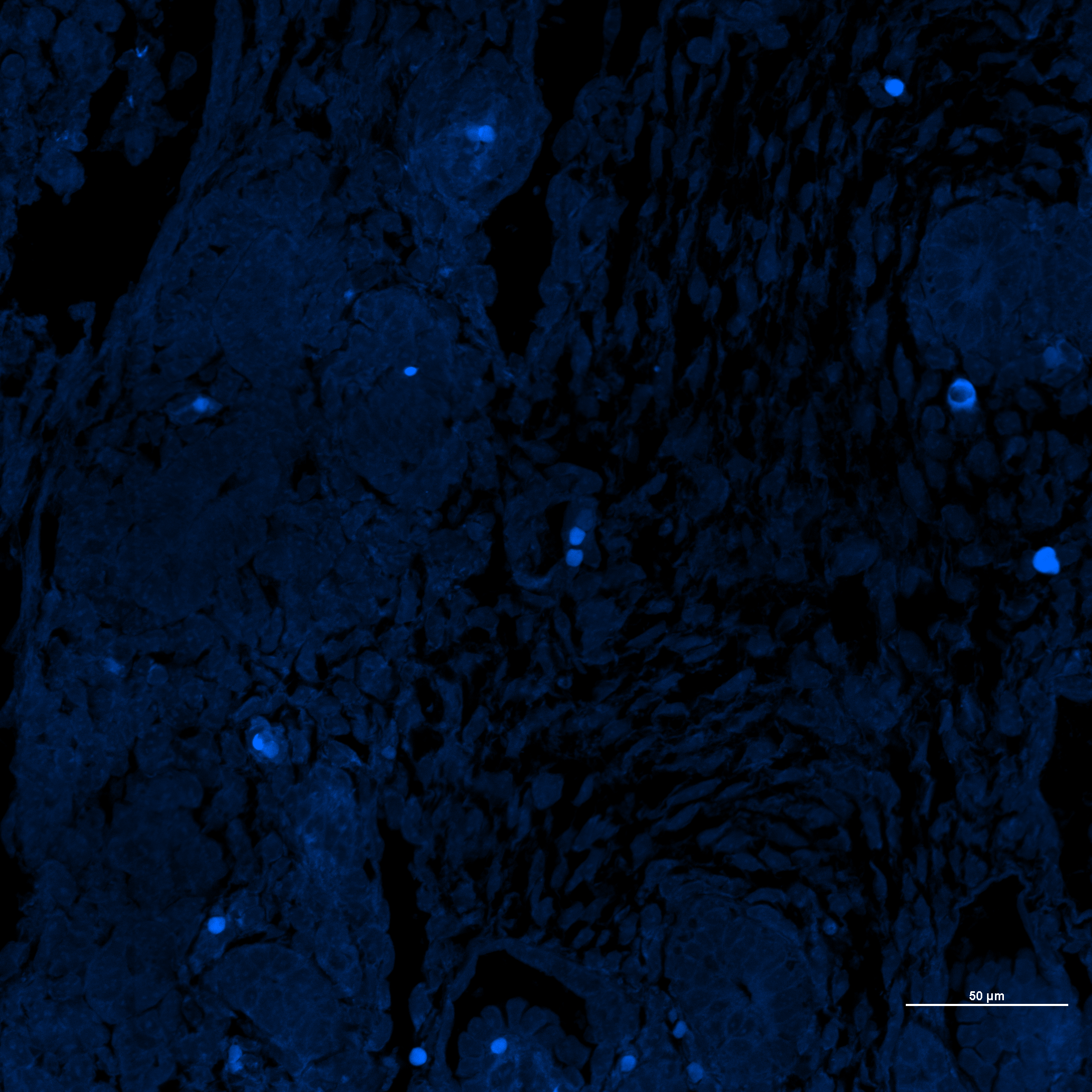

Supplement: Supplementary file 10 — Source Data Fig. 4 [file 44319_2023_19_MOESM10_ESM.zip › Fig.4/4I/Cep120-KO_Meis1_CC3_E-cad_RGB_405-SD .tif]

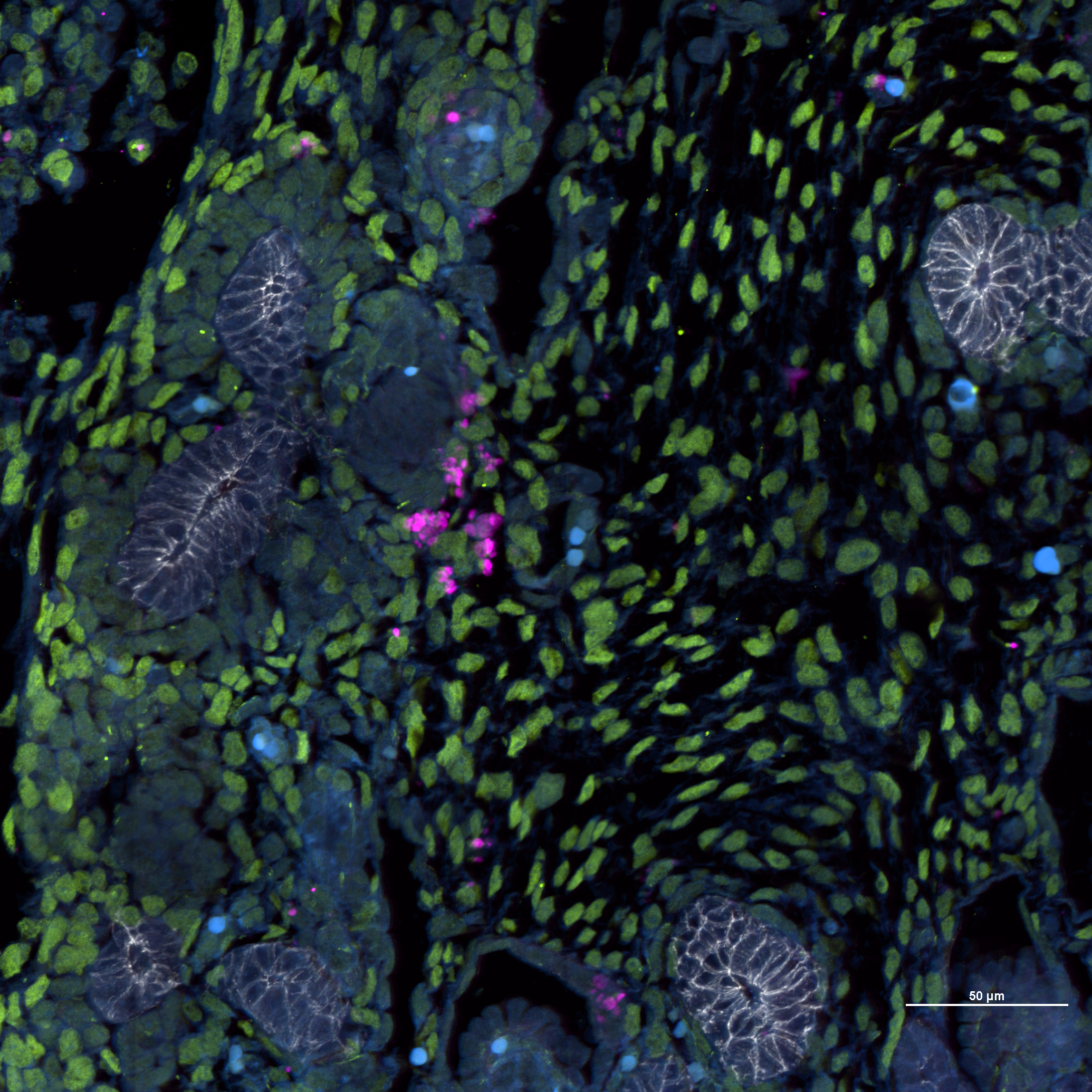

Supplement: Supplementary file 10 — Source Data Fig. 4 [file 44319_2023_19_MOESM10_ESM.zip › Fig.4/4I/Cep120-KO_Meis1_CC3_E-cad_RGB.tif]

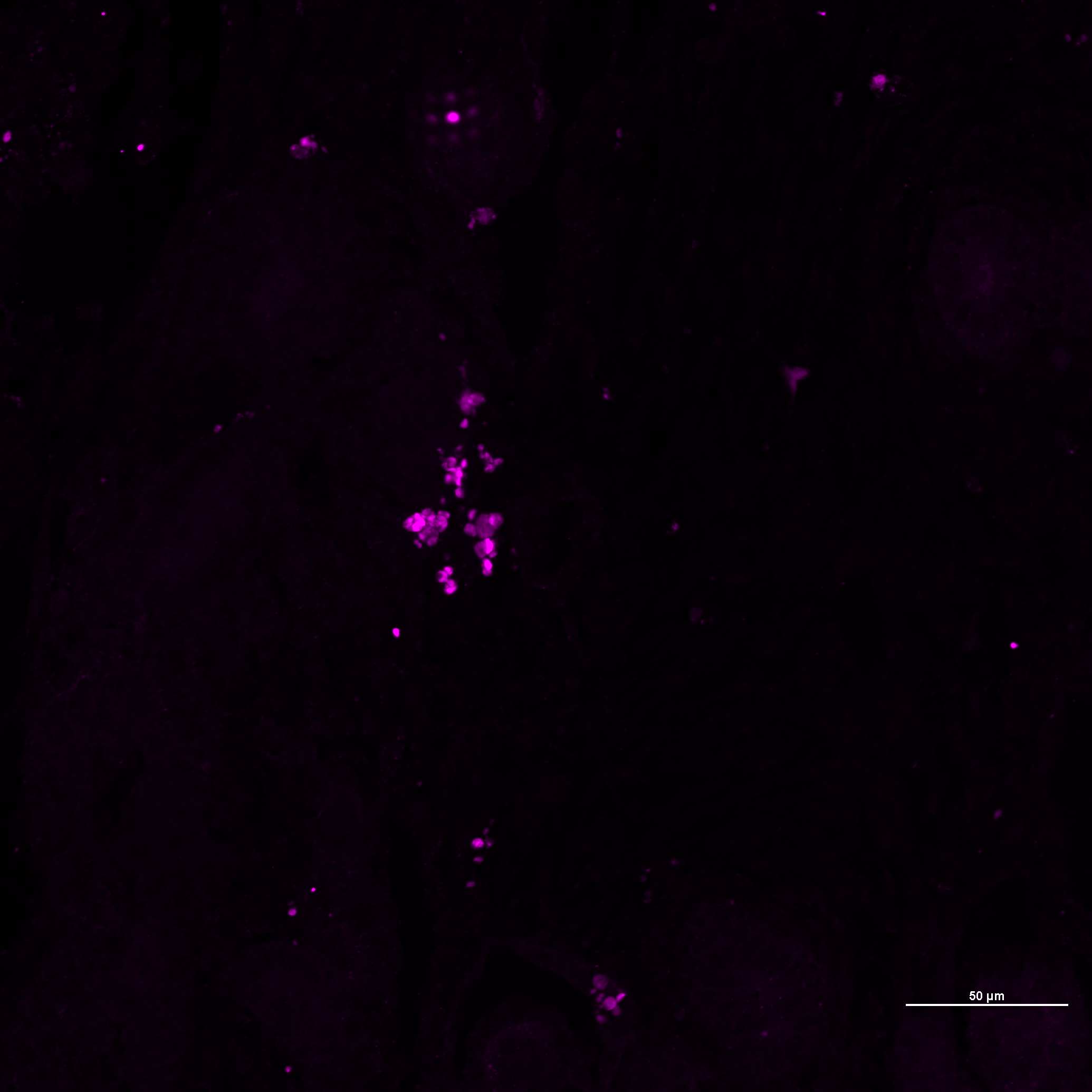

Supplement: Supplementary file 10 — Source Data Fig. 4 [file 44319_2023_19_MOESM10_ESM.zip › Fig.4/4I/Cep120-KO_Meis1_CC3_E-cad_RGB_640-SD.tif]

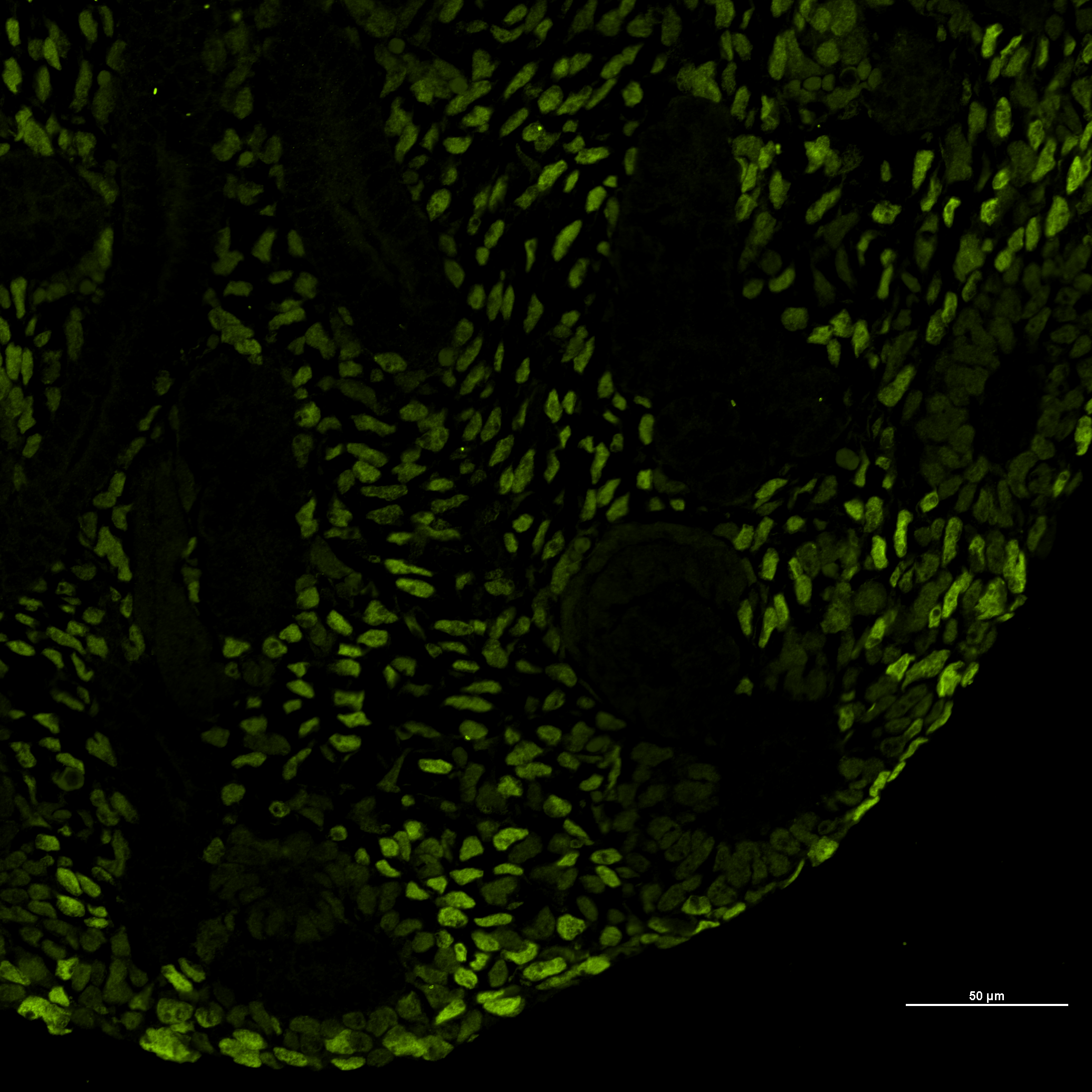

Supplement: Supplementary file 10 — Source Data Fig. 4 [file 44319_2023_19_MOESM10_ESM.zip › Fig.4/4I/Ctrl_Meis1_CC3_E-cad_RGB_488-SD.tif]

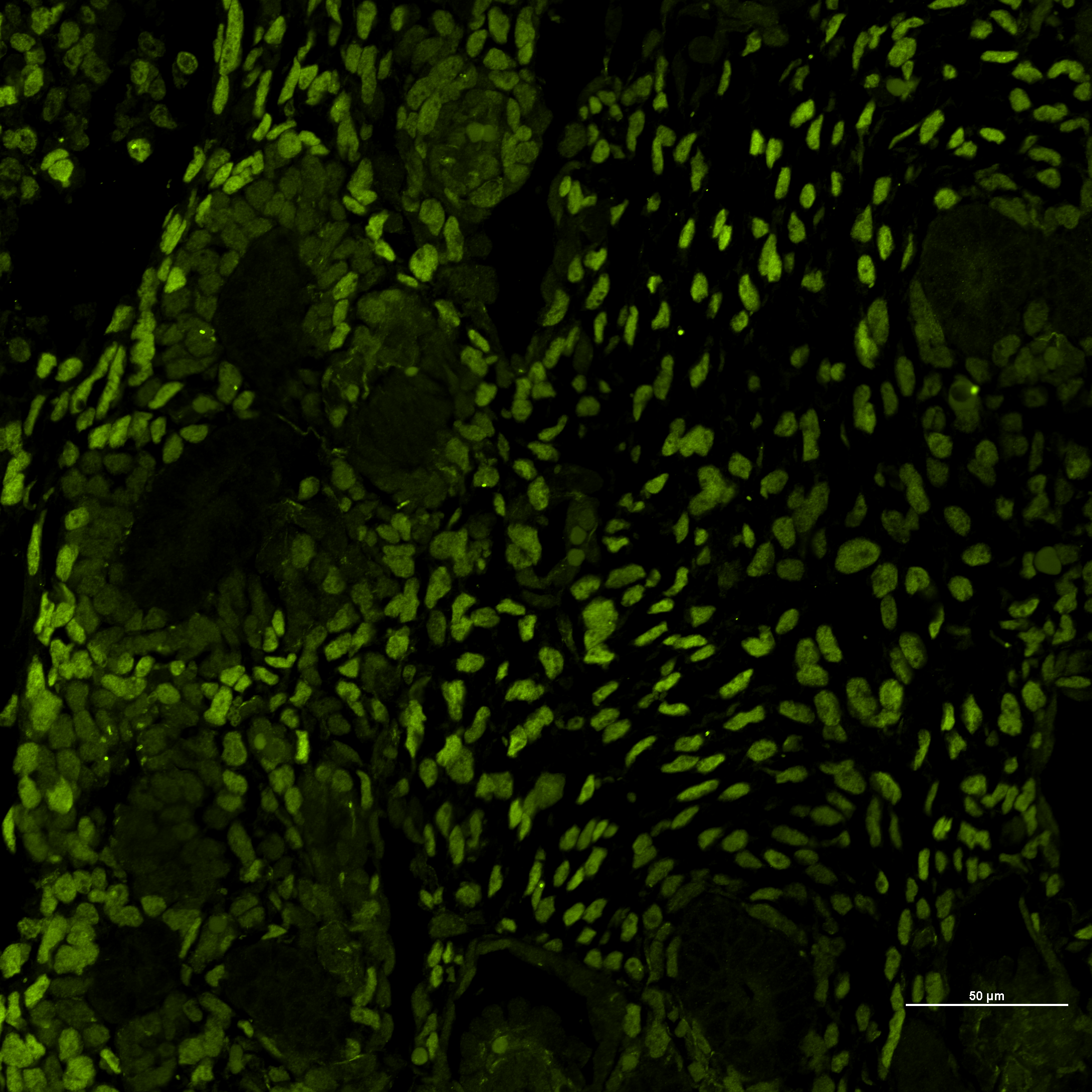

Supplement: Supplementary file 10 — Source Data Fig. 4 [file 44319_2023_19_MOESM10_ESM.zip › Fig.4/4I/Cep120-KO_Meis1_CC3_E-cad_RGB_488-SD.tif]

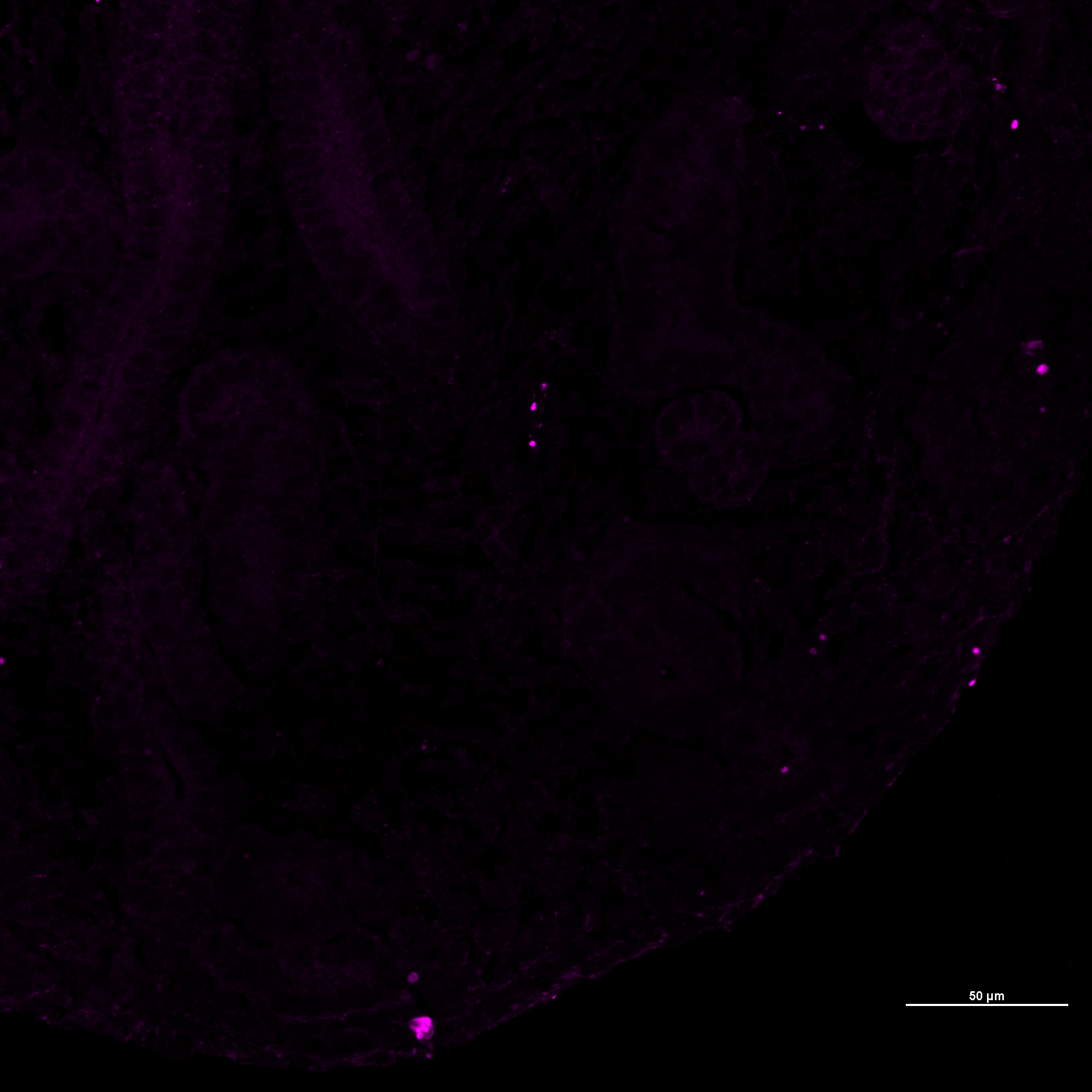

Supplement: Supplementary file 10 — Source Data Fig. 4 [file 44319_2023_19_MOESM10_ESM.zip › Fig.4/4I/Ctrl_Meis1_CC3_E-cad_RGB_640-SD.tif]

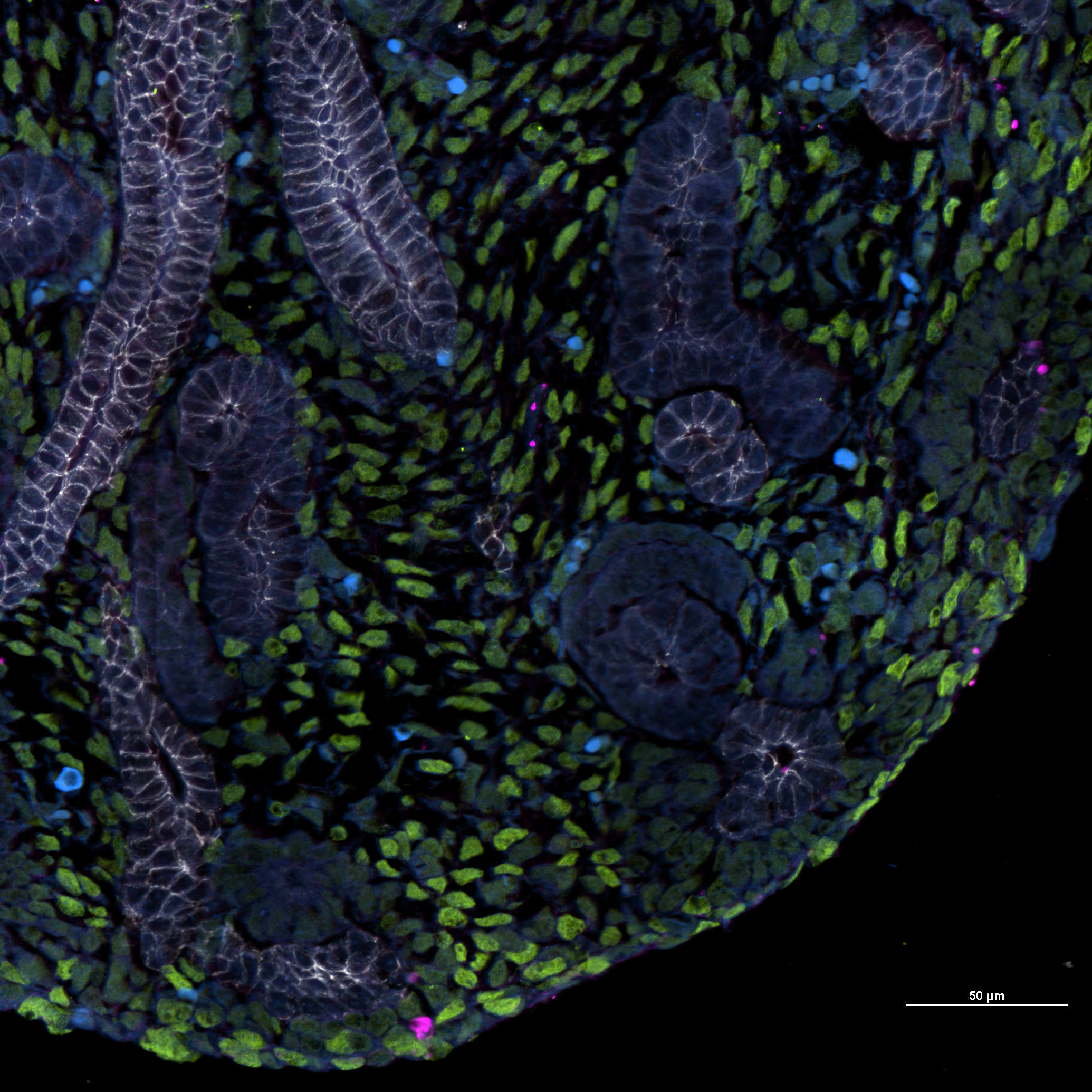

Supplement: Supplementary file 10 — Source Data Fig. 4 [file 44319_2023_19_MOESM10_ESM.zip › Fig.4/4I/Ctrl_Meis1_CC3_E-cad_RGB.tif]

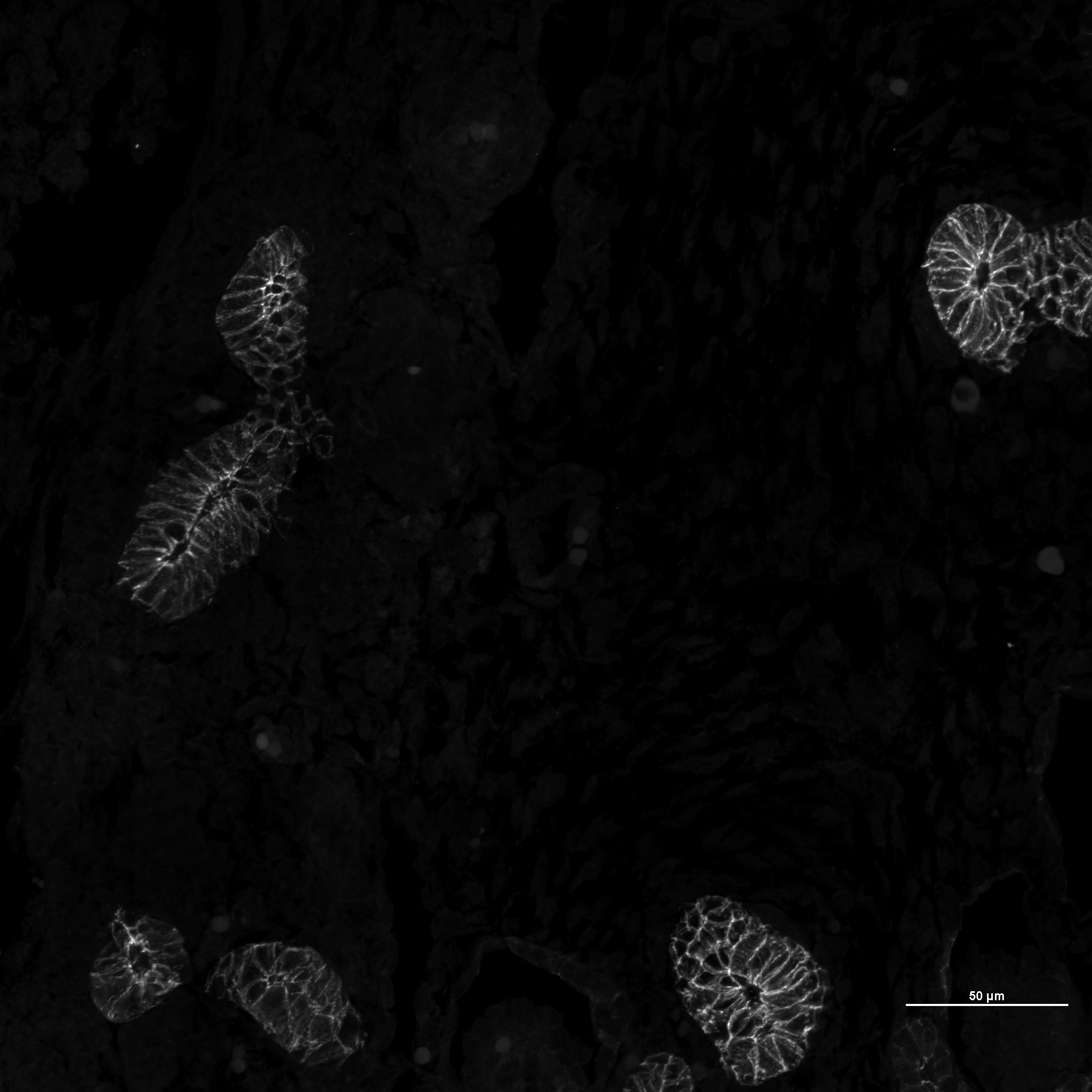

Supplement: Supplementary file 10 — Source Data Fig. 4 [file 44319_2023_19_MOESM10_ESM.zip › Fig.4/4I/Cep120-KO_Meis1_CC3_E-cad_RGB_561-SD.tif]

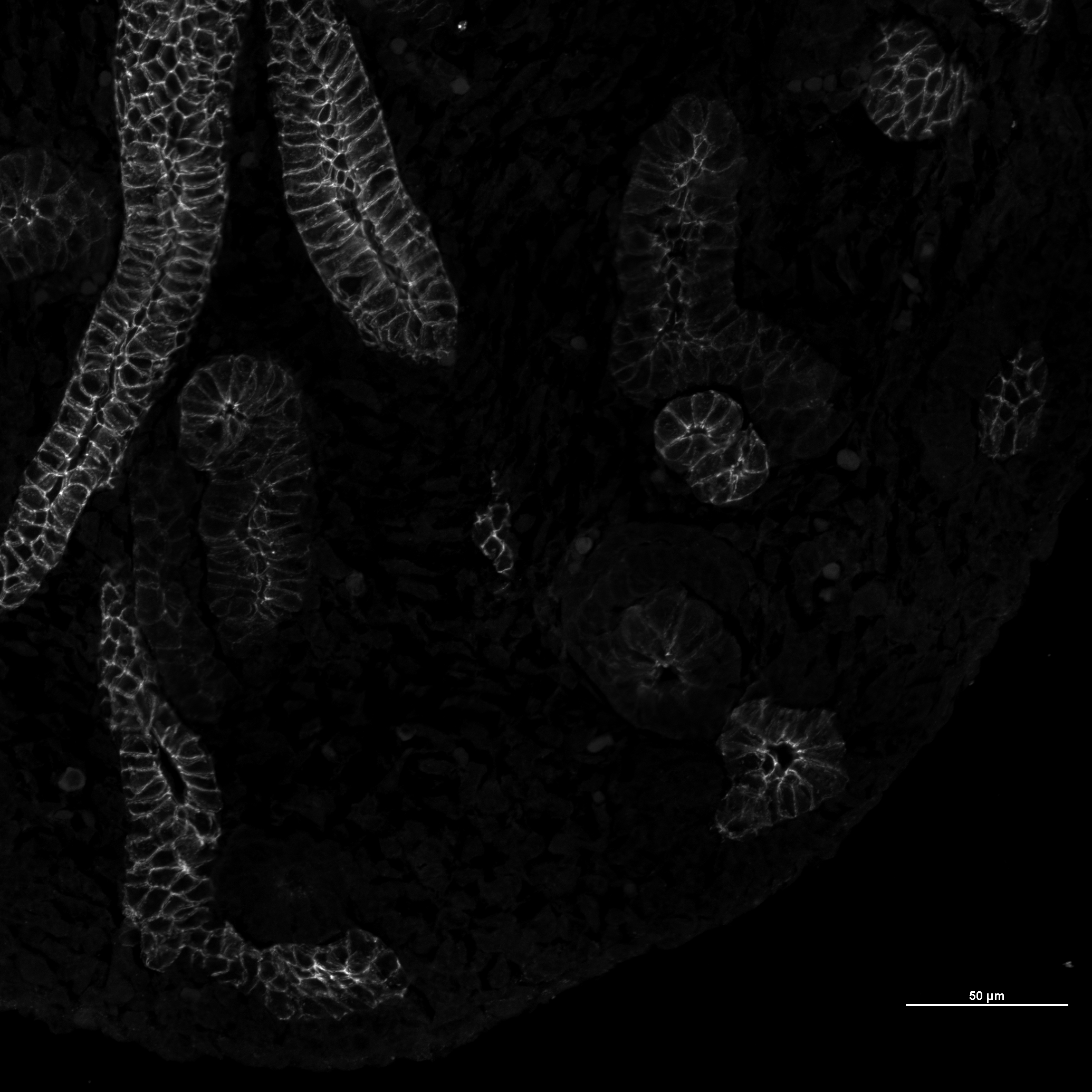

Supplement: Supplementary file 10 — Source Data Fig. 4 [file 44319_2023_19_MOESM10_ESM.zip › Fig.4/4I/Ctrl_Meis1_CC3_E-cad_RGB_561-SD.tif]

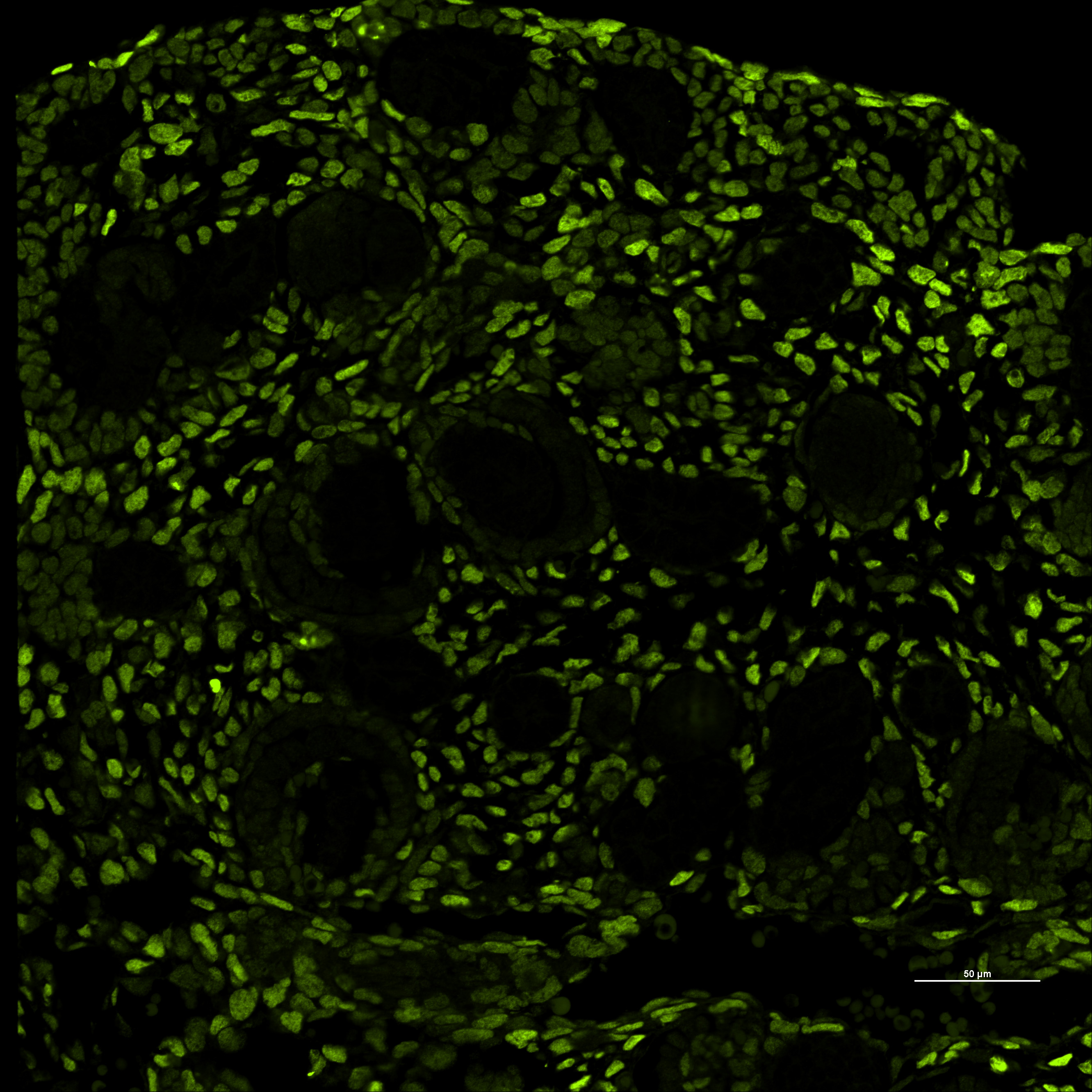

Supplement: Supplementary file 10 — Source Data Fig. 4 [file 44319_2023_19_MOESM10_ESM.zip › Fig.4/4G/Cep120-KO-Meis1_E-cad_p53-MaxIP_RGB_488-SD copy.jpg]

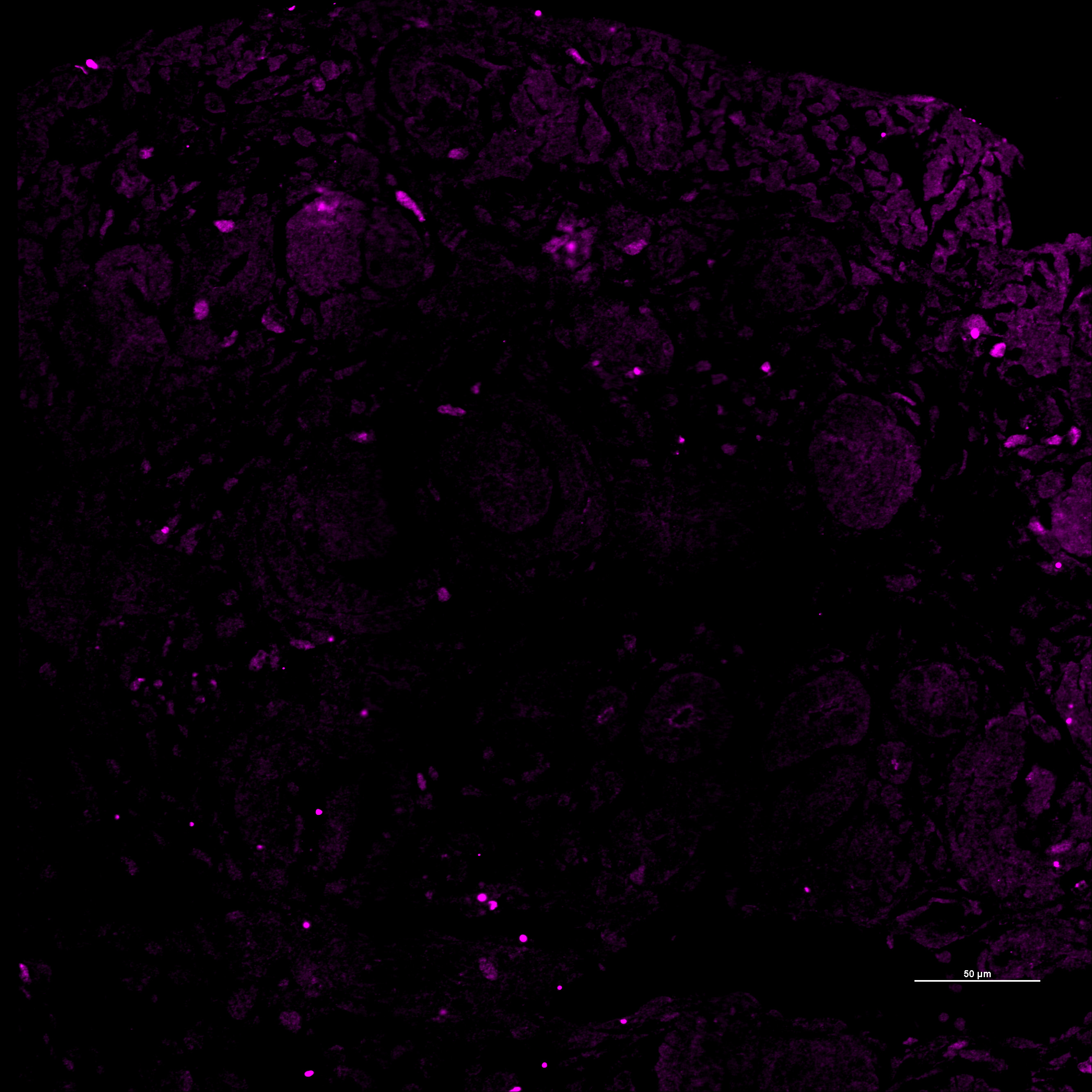

Supplement: Supplementary file 10 — Source Data Fig. 4 [file 44319_2023_19_MOESM10_ESM.zip › Fig.4/4G/Cep120-KO-Meis1_E-cad_p53-MaxIP_RGB_640-SD copy.jpg]

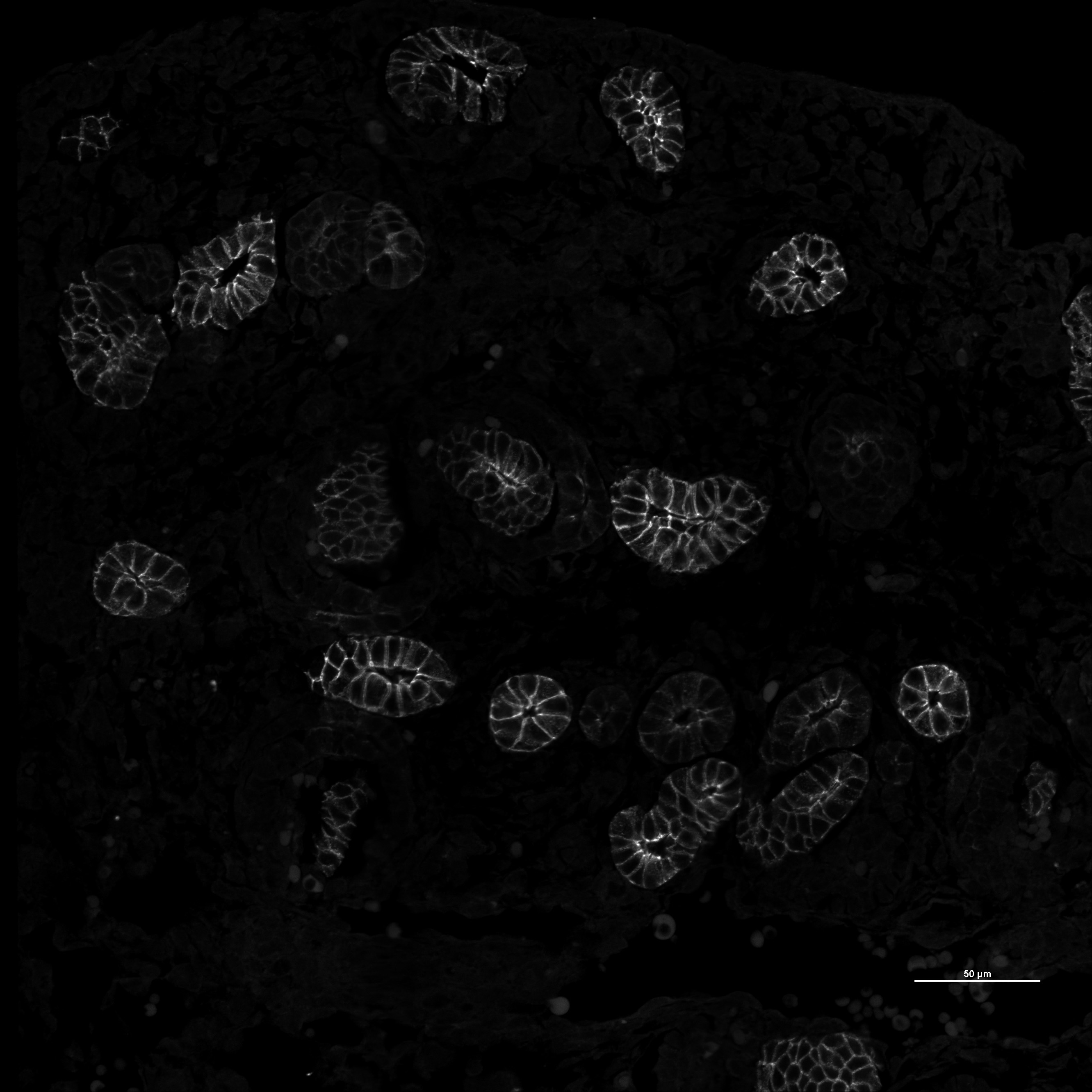

Supplement: Supplementary file 10 — Source Data Fig. 4 [file 44319_2023_19_MOESM10_ESM.zip › Fig.4/4G/Cep120-KO-Meis1_E-cad_p53-MaxIP_RGB_561-SD copy.jpg]

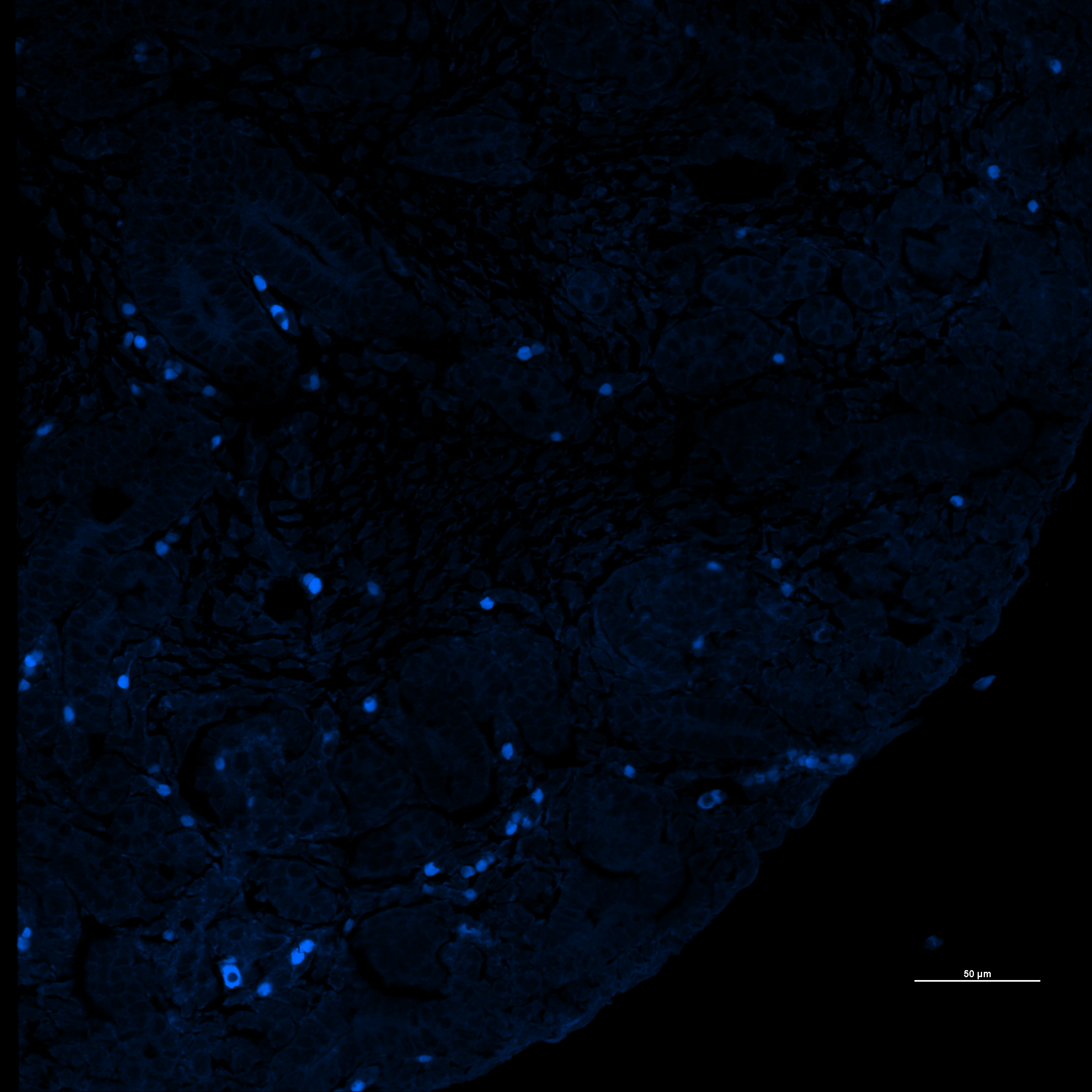

Supplement: Supplementary file 10 — Source Data Fig. 4 [file 44319_2023_19_MOESM10_ESM.zip › Fig.4/4G/Ctrl-Meis1_E-cad_p53-MaxIP_RGB_405-SD .tif]

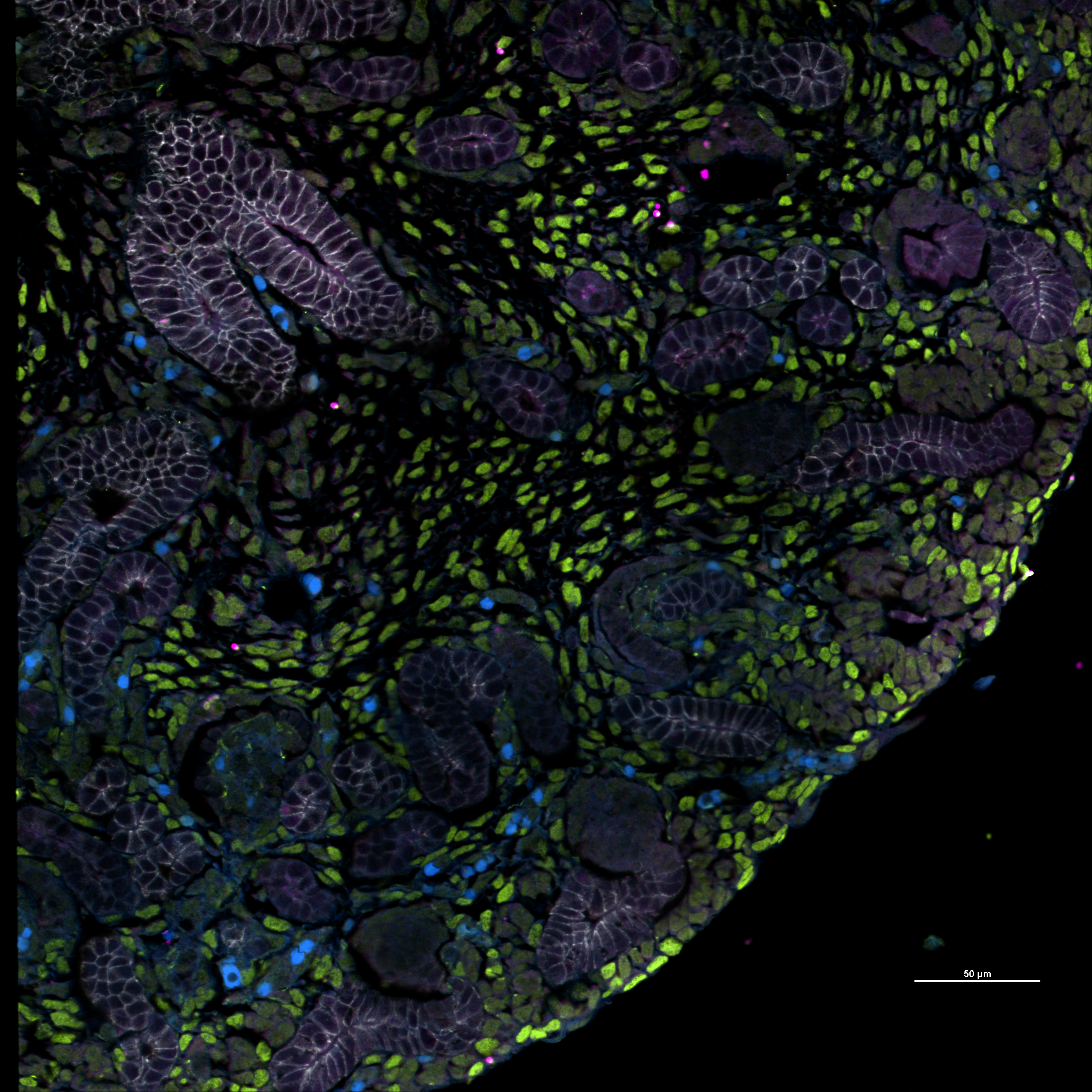

Supplement: Supplementary file 10 — Source Data Fig. 4 [file 44319_2023_19_MOESM10_ESM.zip › Fig.4/4G/Ctrl-Meis1_E-cad_p53-MaxIP_RGB.tif]

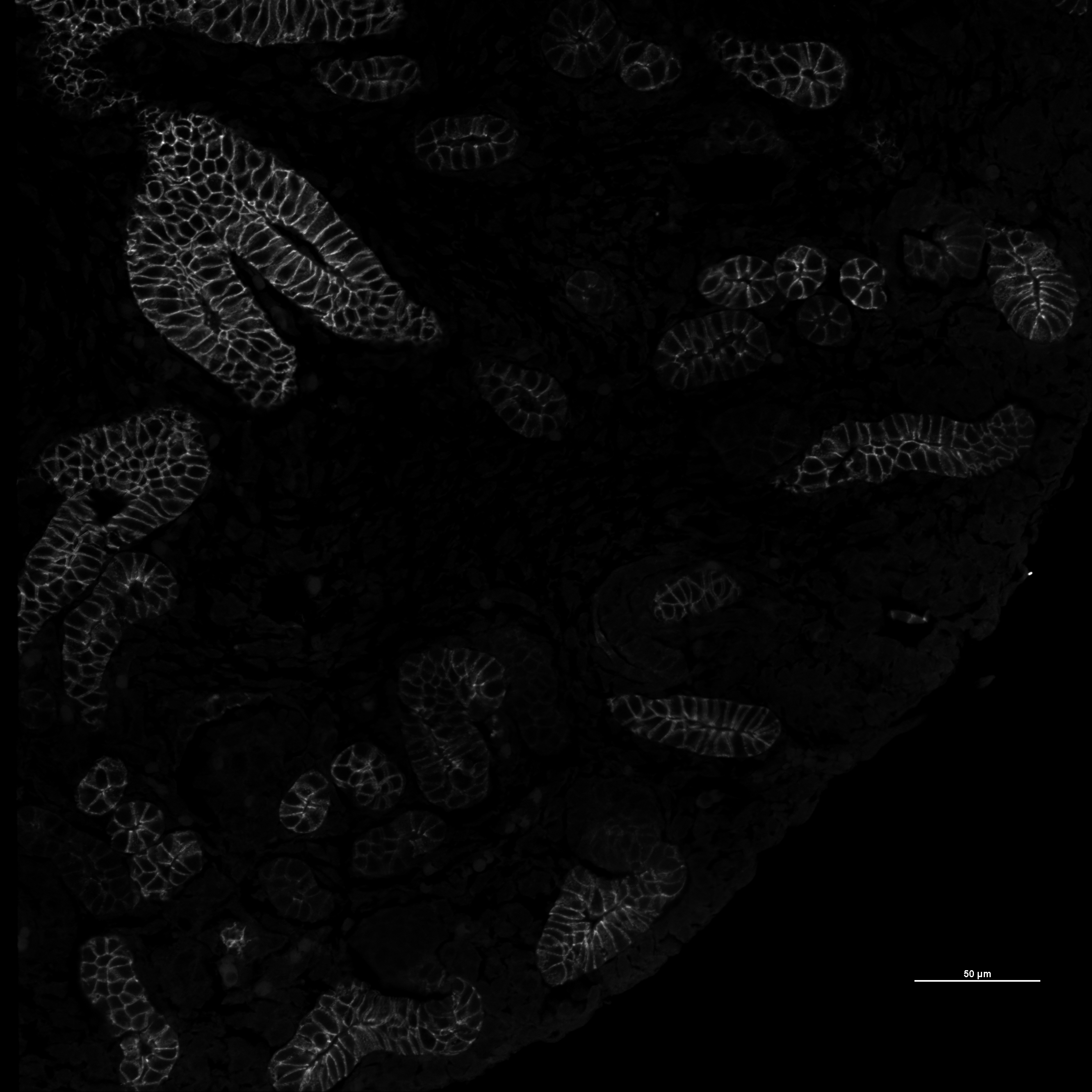

Supplement: Supplementary file 10 — Source Data Fig. 4 [file 44319_2023_19_MOESM10_ESM.zip › Fig.4/4G/Ctrl-Meis1_E-cad_p53-MaxIP_RGB_561-SD.tif]

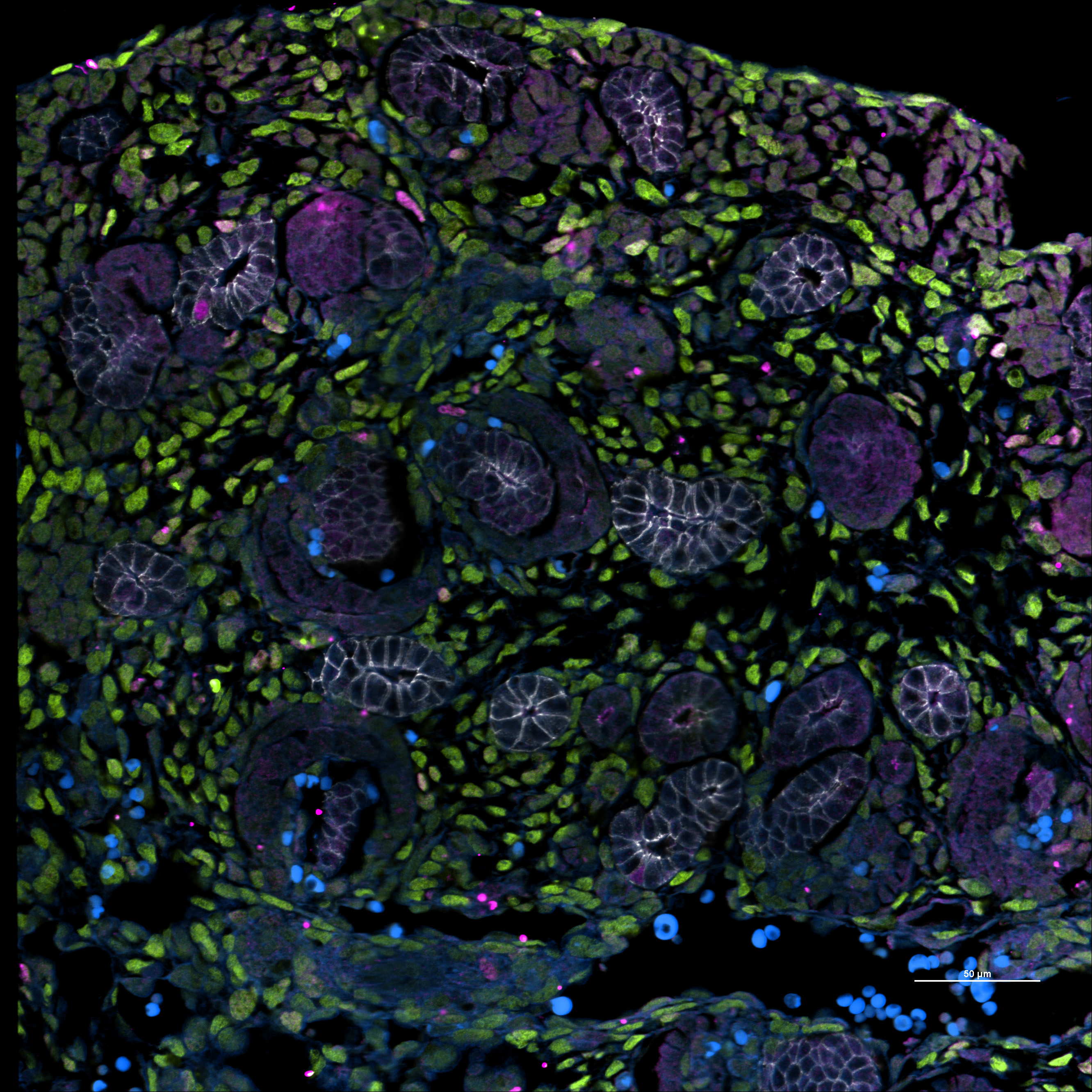

Supplement: Supplementary file 10 — Source Data Fig. 4 [file 44319_2023_19_MOESM10_ESM.zip › Fig.4/4G/Cep120-KO-Meis1_E-cad_p53-MaxIP_RGB copy.jpg]

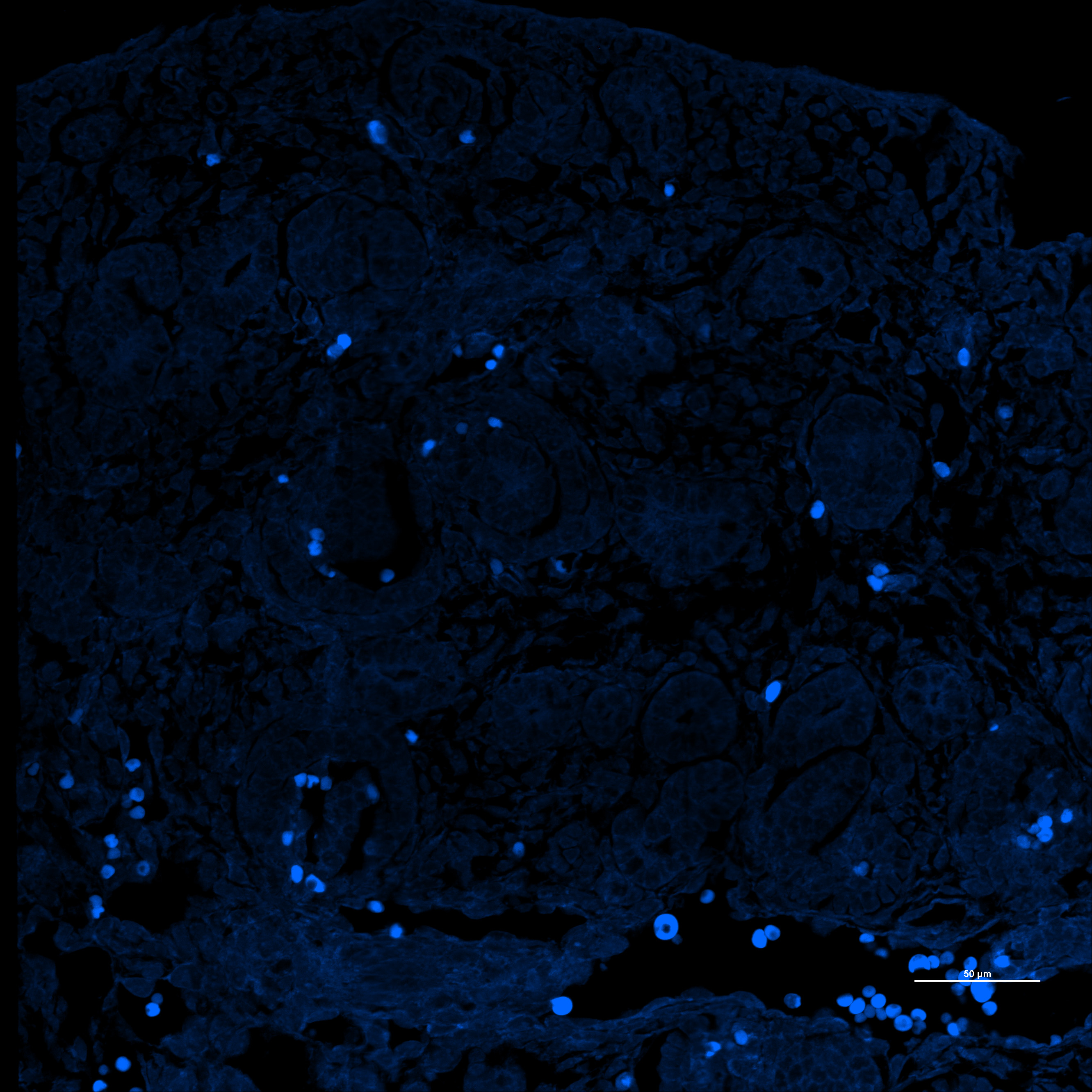

Supplement: Supplementary file 10 — Source Data Fig. 4 [file 44319_2023_19_MOESM10_ESM.zip › Fig.4/4G/Cep120-KO-Meis1_E-cad_p53-MaxIP_RGB_405-SD copy.jpg]

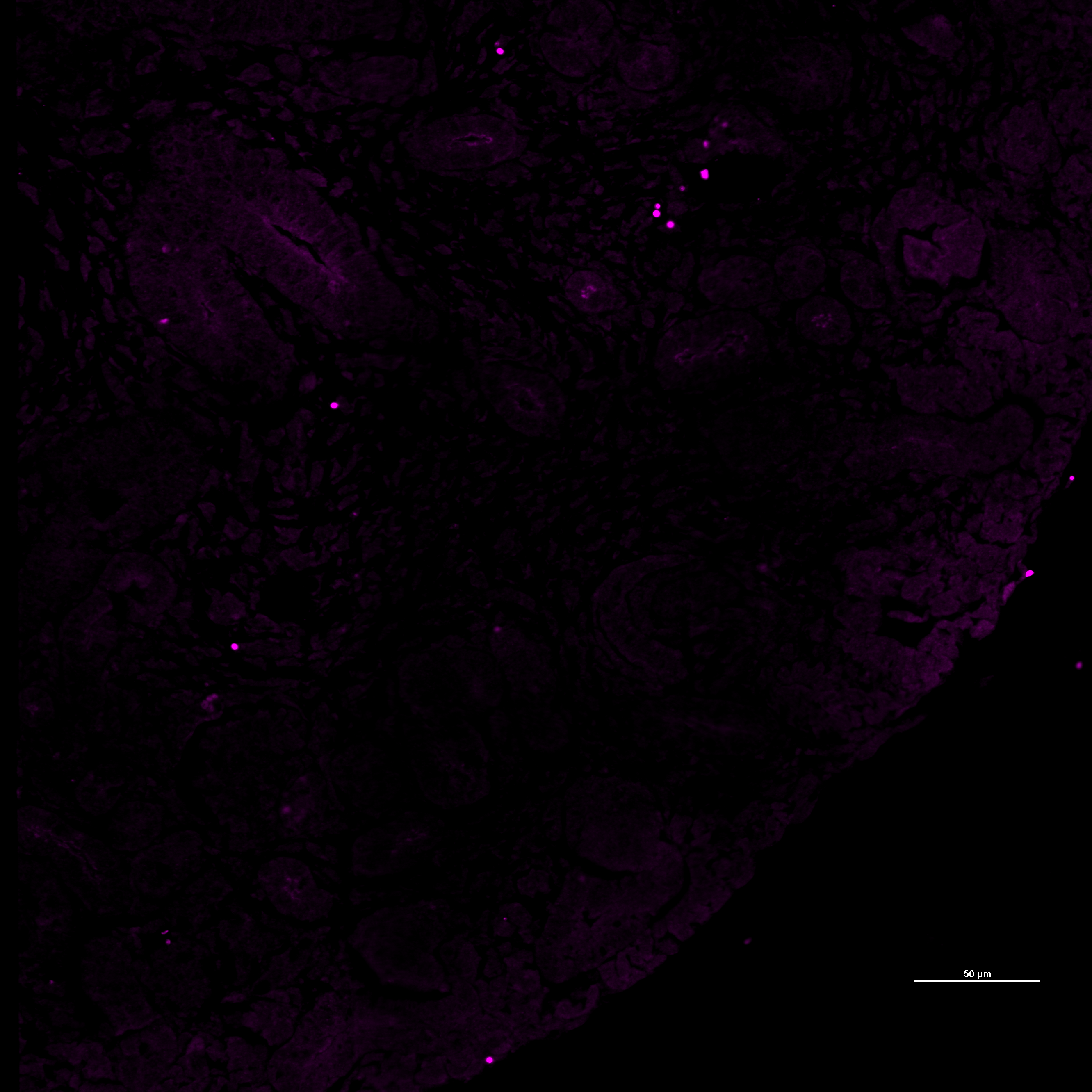

Supplement: Supplementary file 10 — Source Data Fig. 4 [file 44319_2023_19_MOESM10_ESM.zip › Fig.4/4G/Ctrl-Meis1_E-cad_p53-MaxIP_RGB_640-SD.tif]

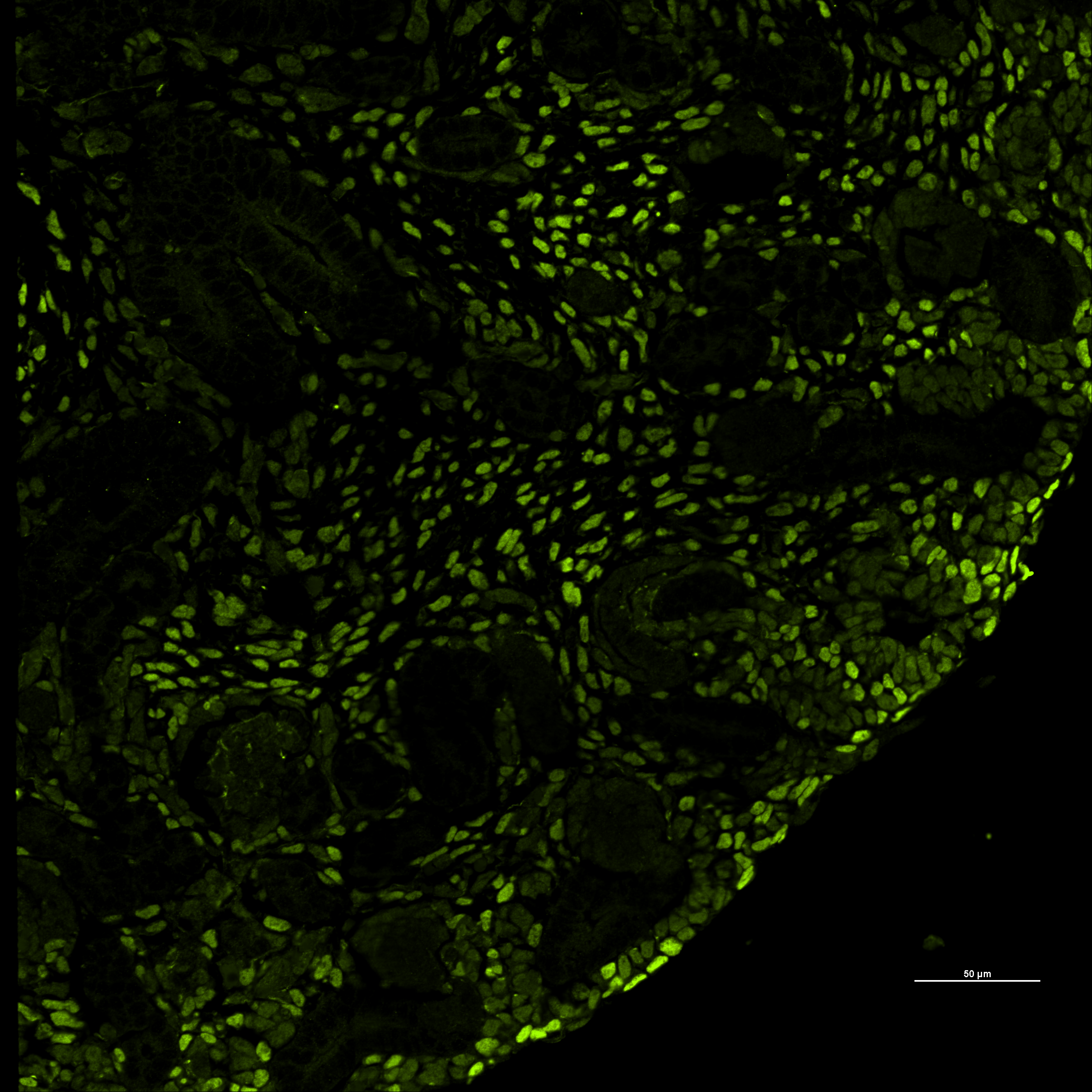

Supplement: Supplementary file 10 — Source Data Fig. 4 [file 44319_2023_19_MOESM10_ESM.zip › Fig.4/4G/Ctrl-Meis1_E-cad_p53-MaxIP_RGB_488-SD.tif]

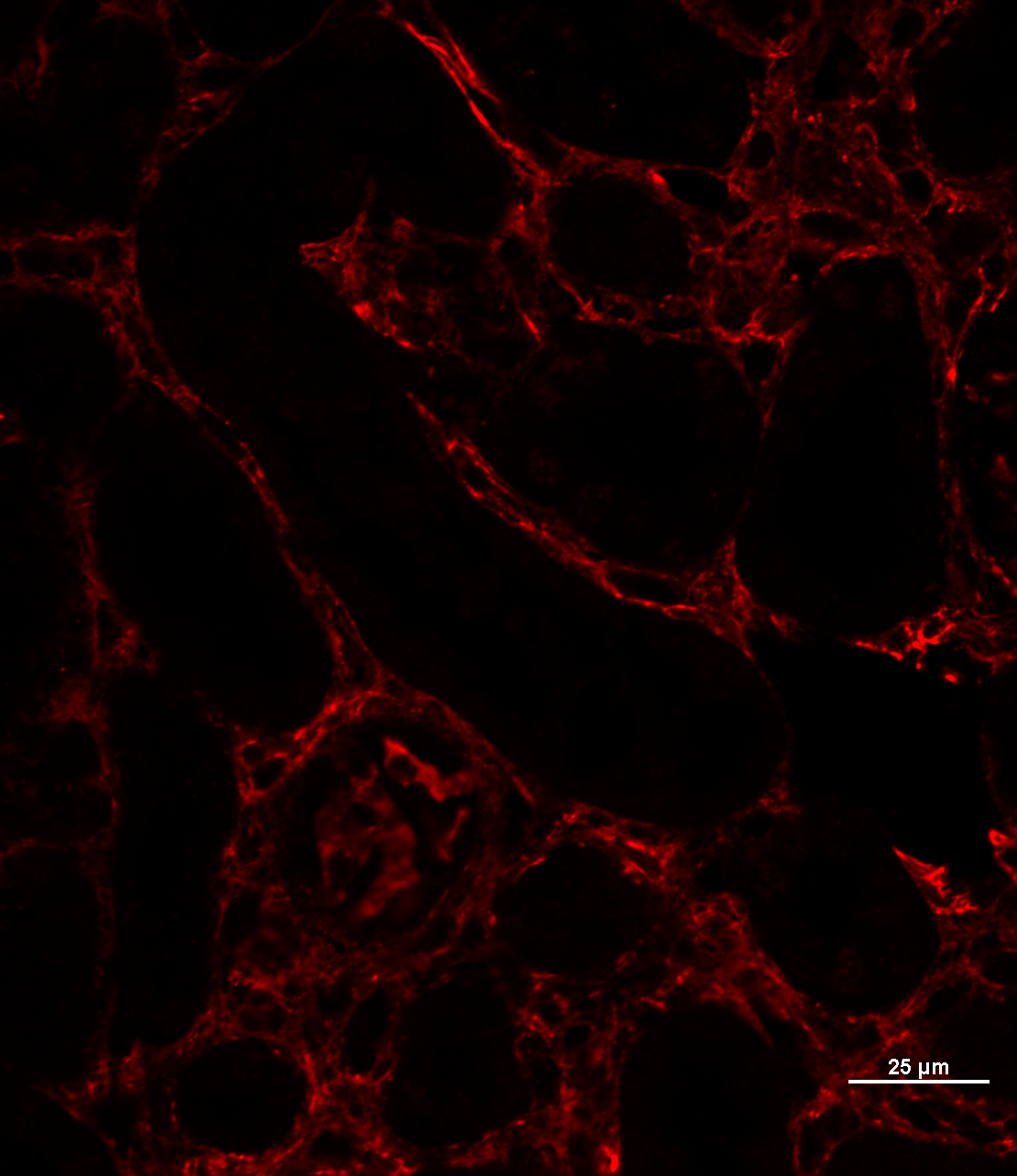

Supplement: Supplementary file 12 — Source Data Fig. 6 [file 44319_2023_19_MOESM12_ESM.zip › Fig.6/6F/Ctrl-UUO_FN1_RGB_488-SD.tif]

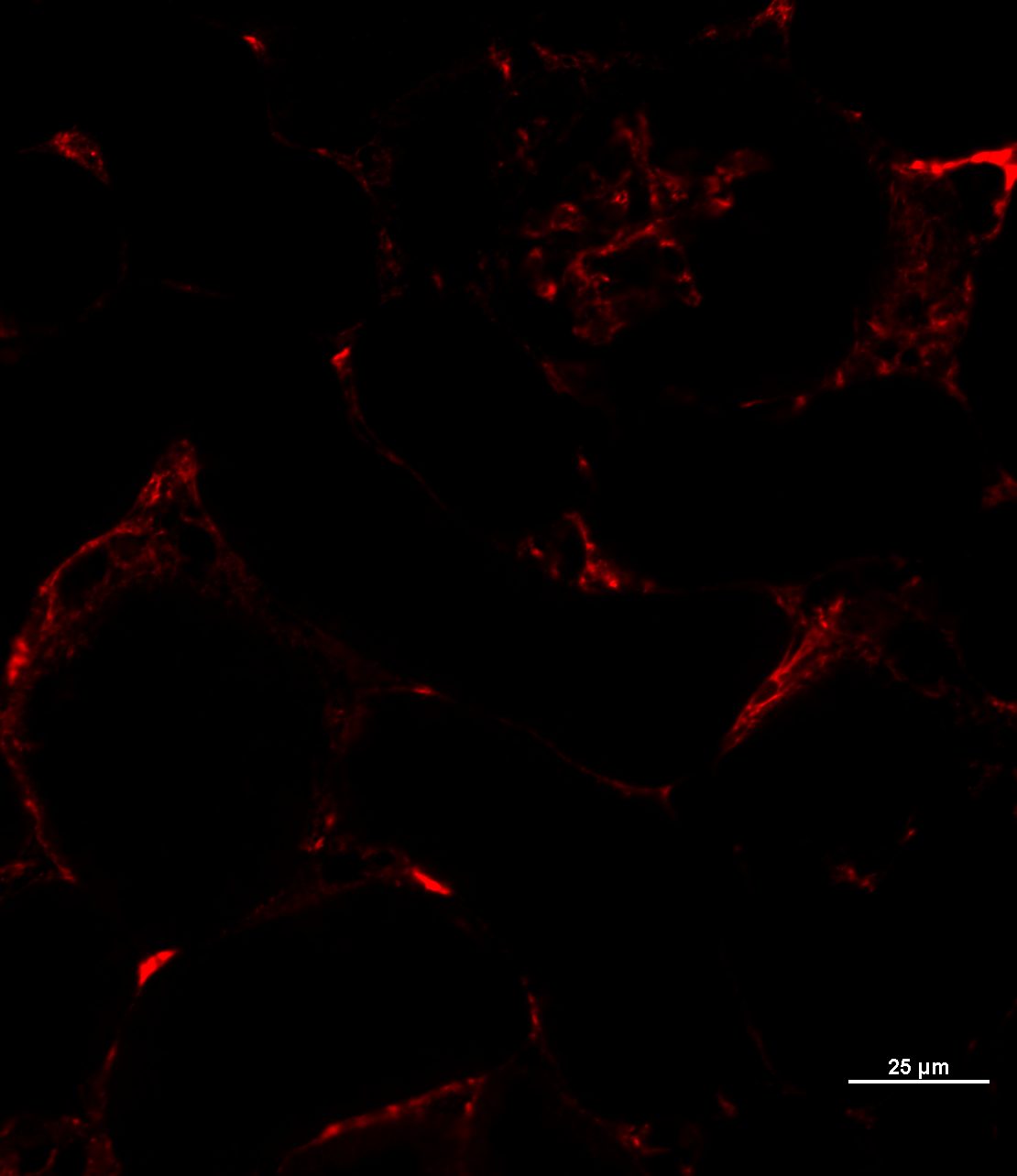

Supplement: Supplementary file 12 — Source Data Fig. 6 [file 44319_2023_19_MOESM12_ESM.zip › Fig.6/6F/Ctrl-sham_FN1_RGB_488-SD.tif]

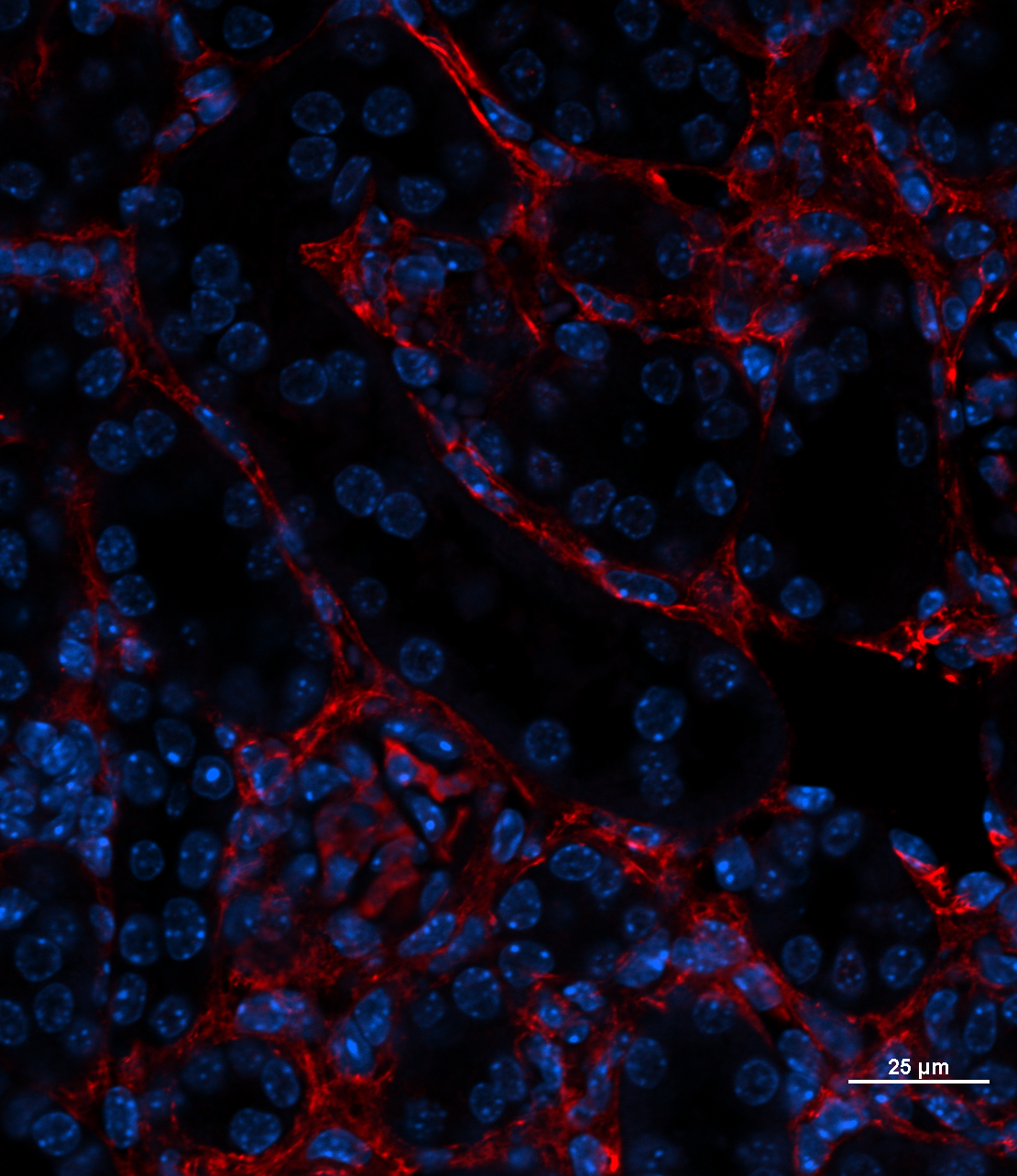

Supplement: Supplementary file 12 — Source Data Fig. 6 [file 44319_2023_19_MOESM12_ESM.zip › Fig.6/6F/Ctrl-UUO_FN1_RGB.tif]

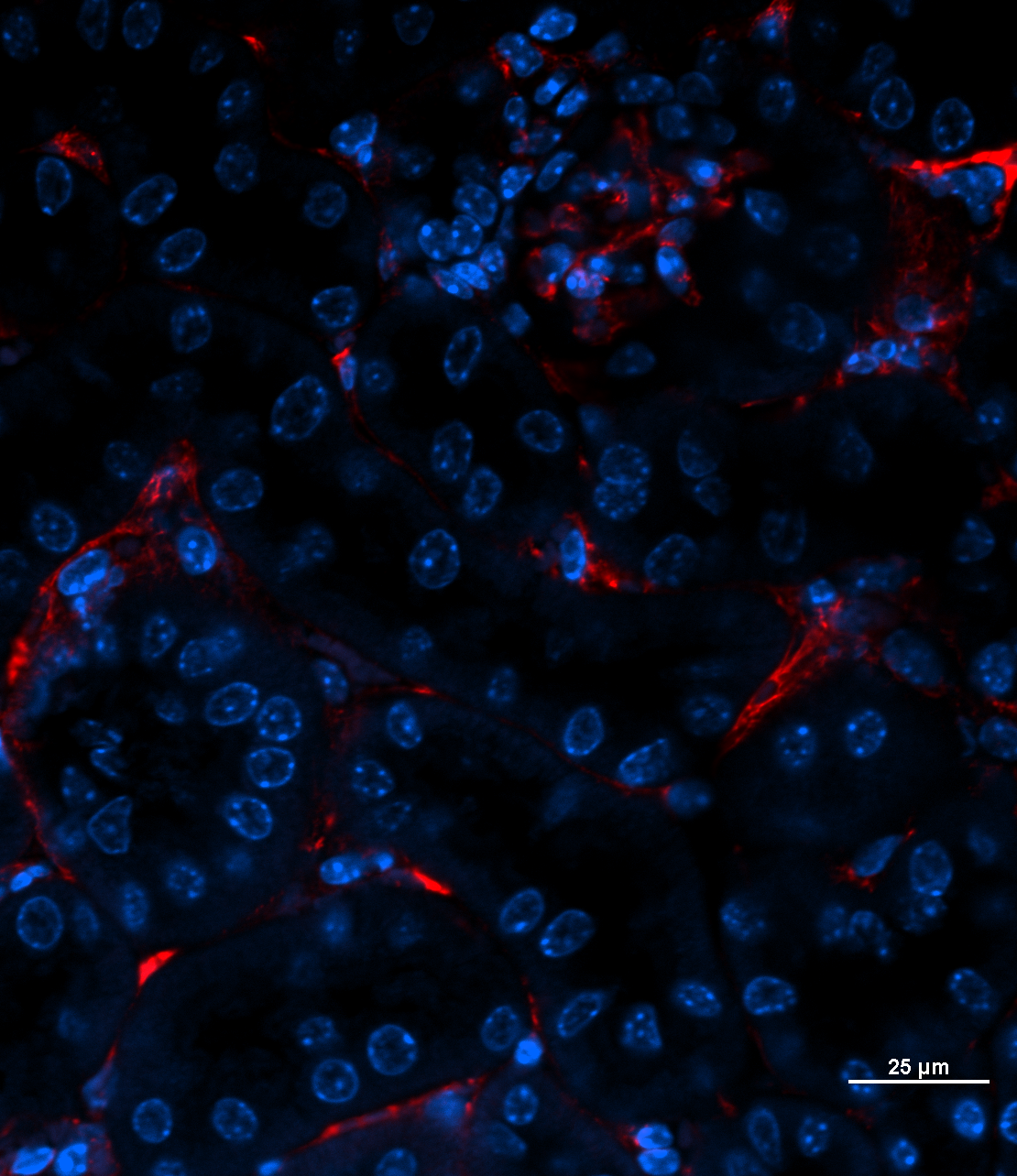

Supplement: Supplementary file 12 — Source Data Fig. 6 [file 44319_2023_19_MOESM12_ESM.zip › Fig.6/6F/Ctrl-sham_FN1_RGB.tif]

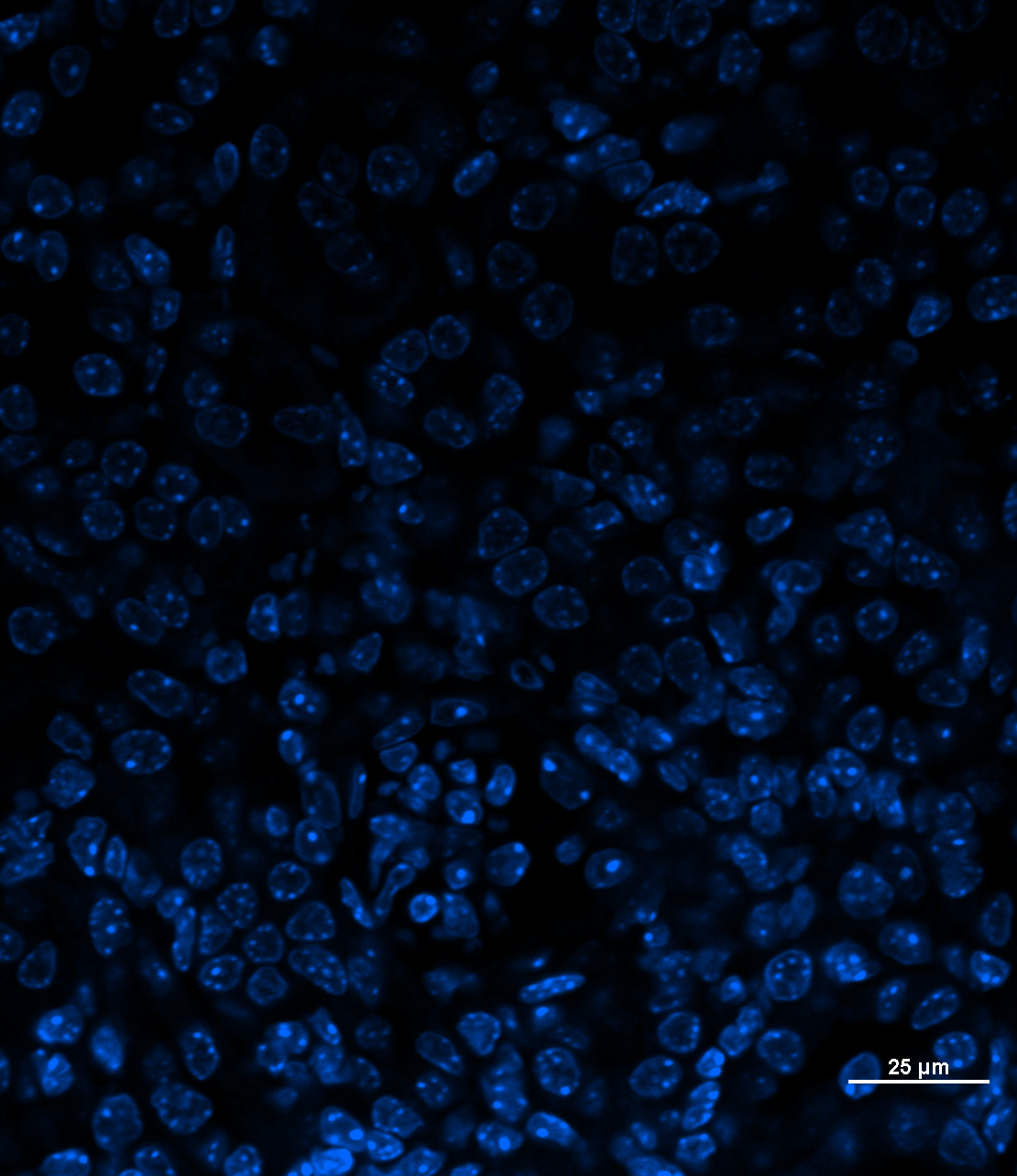

Supplement: Supplementary file 12 — Source Data Fig. 6 [file 44319_2023_19_MOESM12_ESM.zip › Fig.6/6F/Cep120-KO-UUO_FN1_RGB_405-SD .tif]

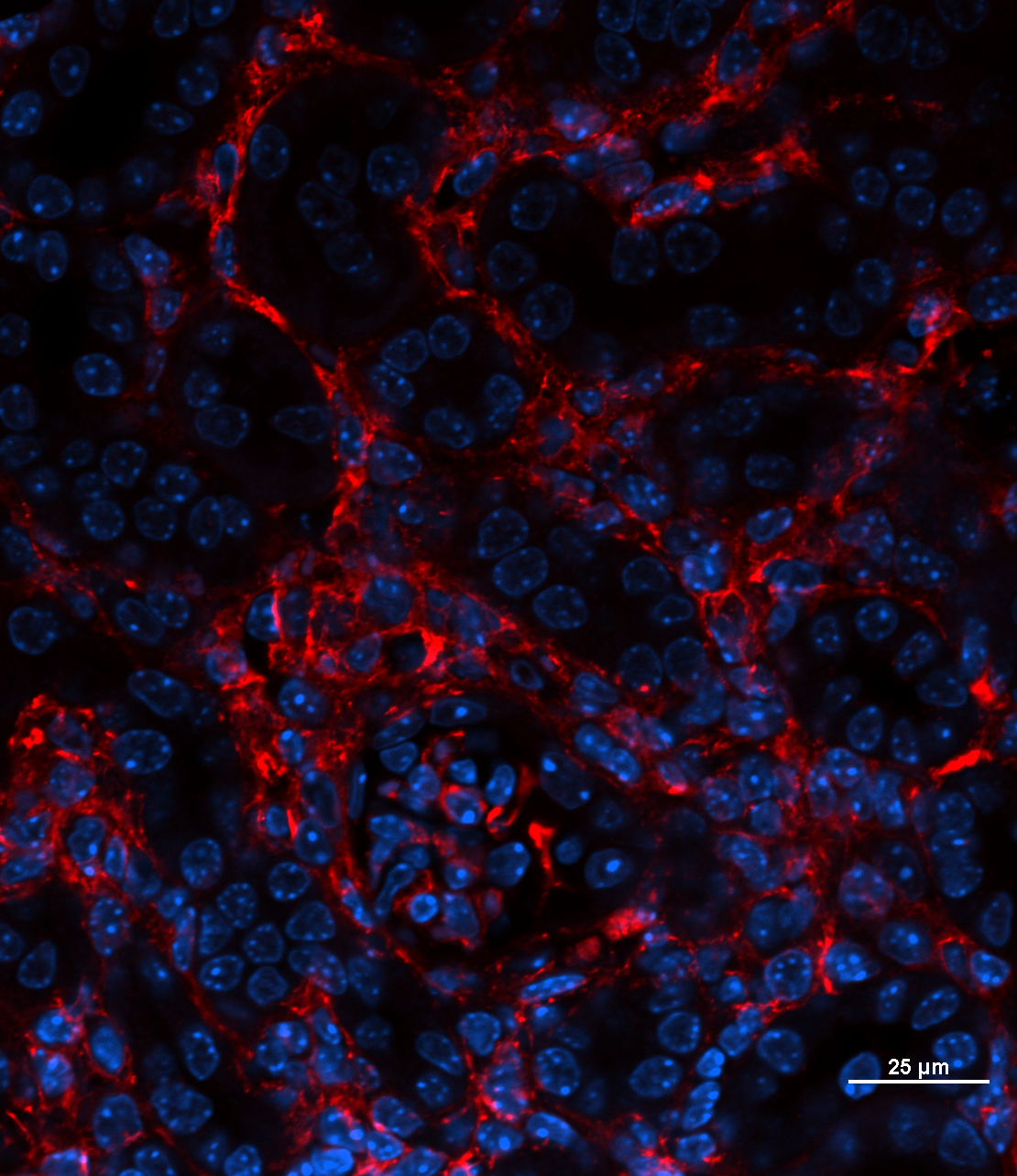

Supplement: Supplementary file 12 — Source Data Fig. 6 [file 44319_2023_19_MOESM12_ESM.zip › Fig.6/6F/Cep120-KO-UUO_FN1_RGB.tif]

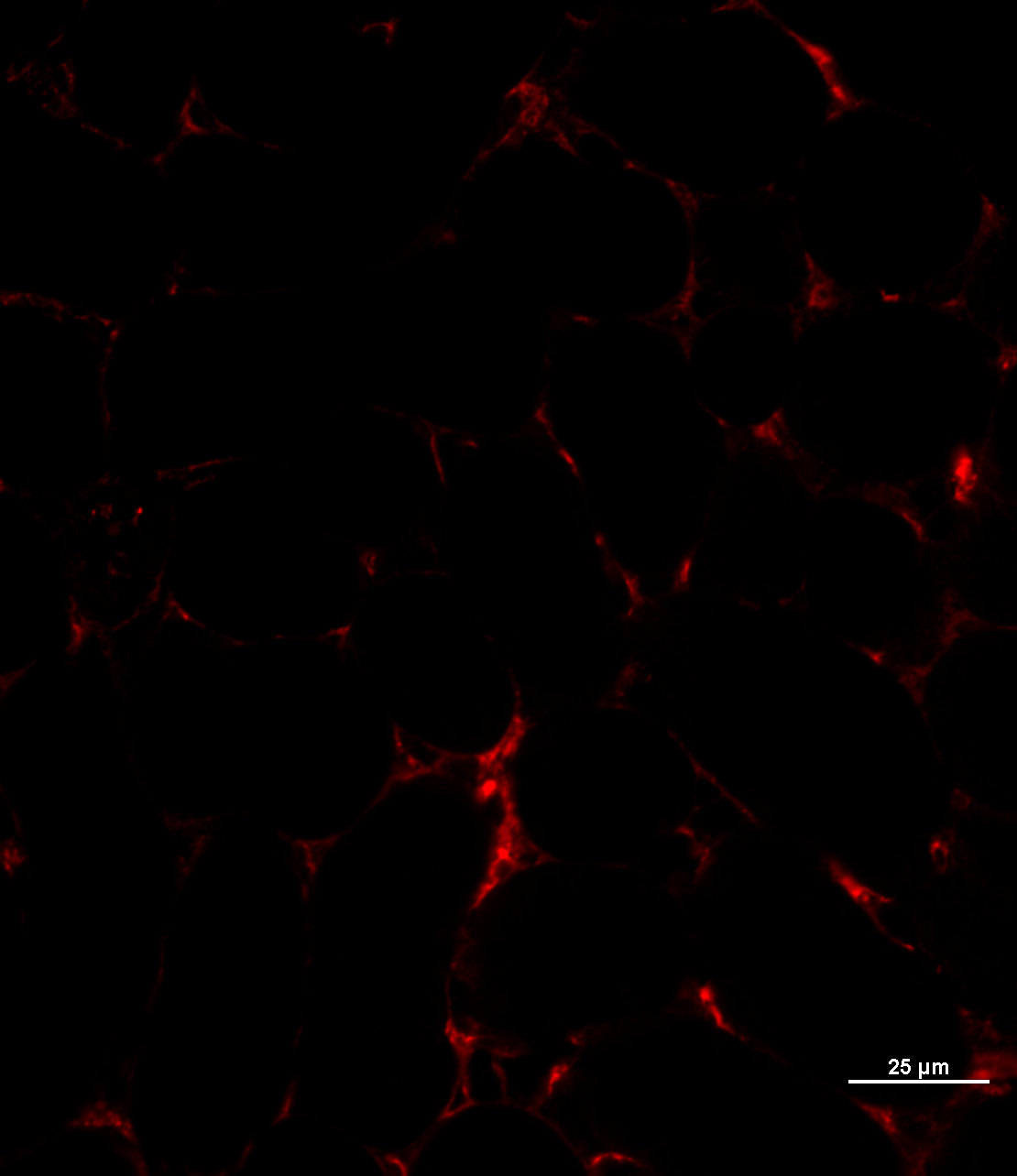

Supplement: Supplementary file 12 — Source Data Fig. 6 [file 44319_2023_19_MOESM12_ESM.zip › Fig.6/6F/Cep120-KO-sham_FN1_RGB_488-SD.tif]

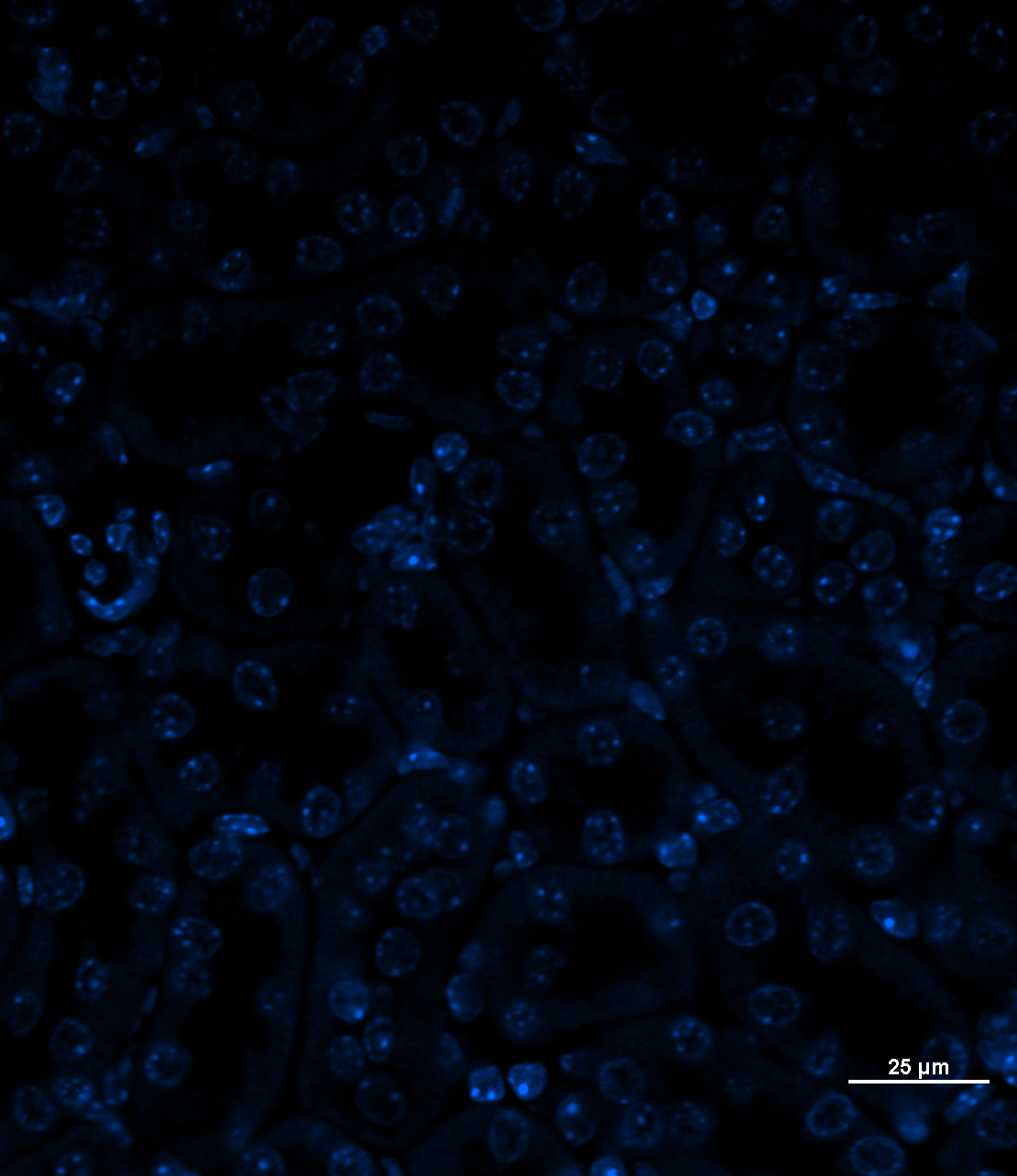

Supplement: Supplementary file 12 — Source Data Fig. 6 [file 44319_2023_19_MOESM12_ESM.zip › Fig.6/6F/Cep120-KO-sham_FN1_RGB_405-SD .tif]

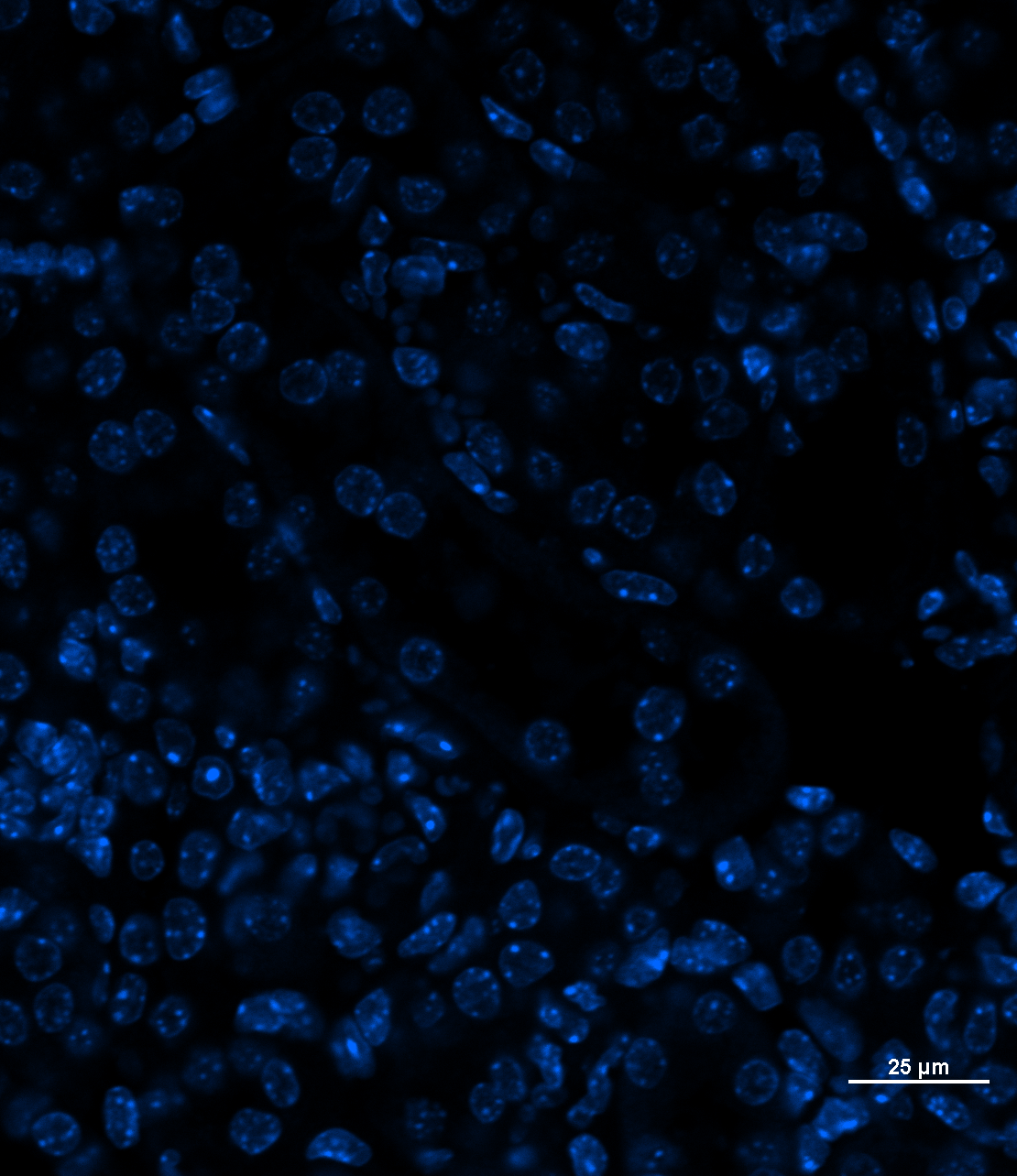

Supplement: Supplementary file 12 — Source Data Fig. 6 [file 44319_2023_19_MOESM12_ESM.zip › Fig.6/6F/Ctrl-UUO_FN1_RGB_405-SD .tif]

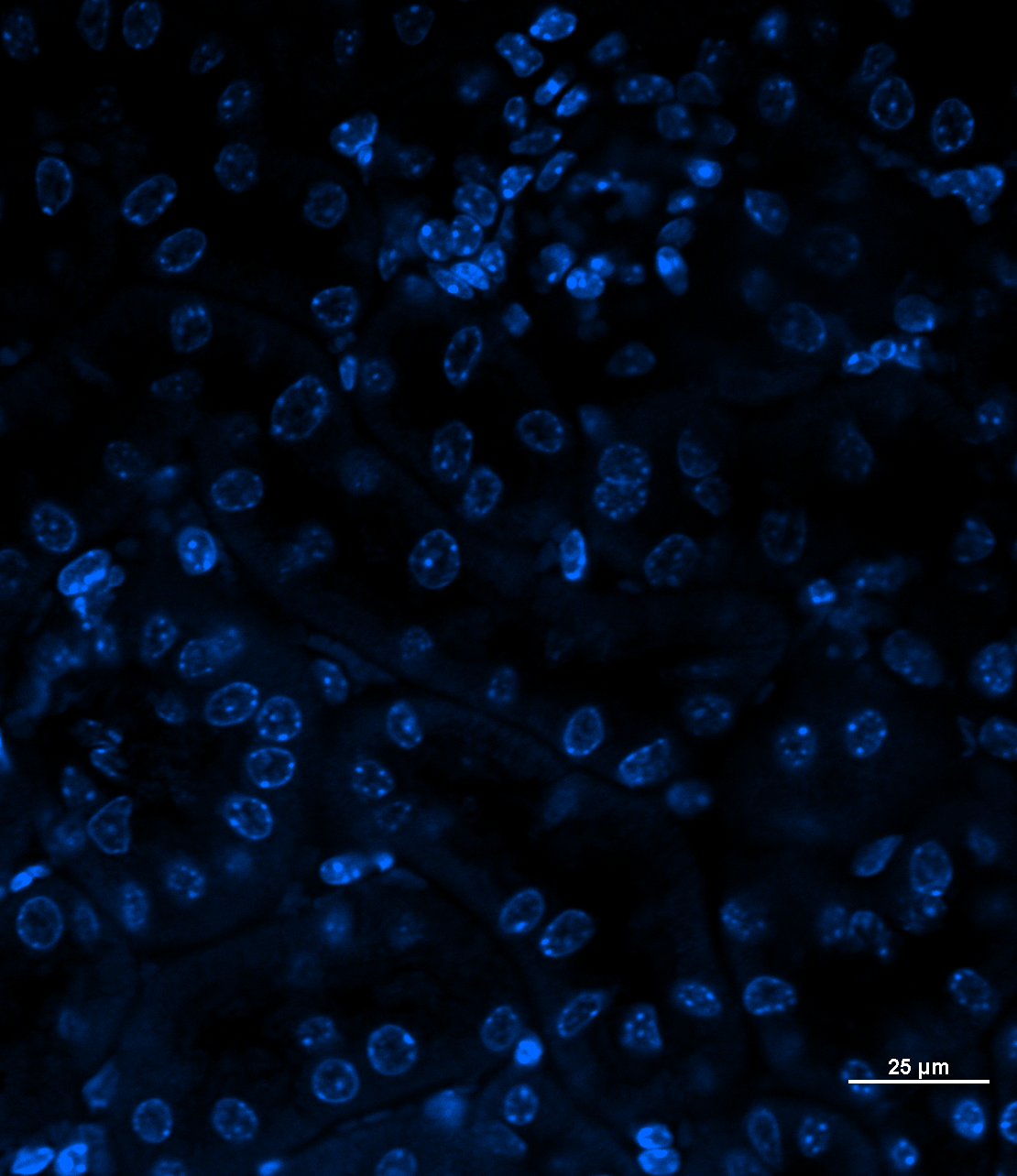

Supplement: Supplementary file 12 — Source Data Fig. 6 [file 44319_2023_19_MOESM12_ESM.zip › Fig.6/6F/Ctrl-sham_FN1_RGB_405-SD .tif]

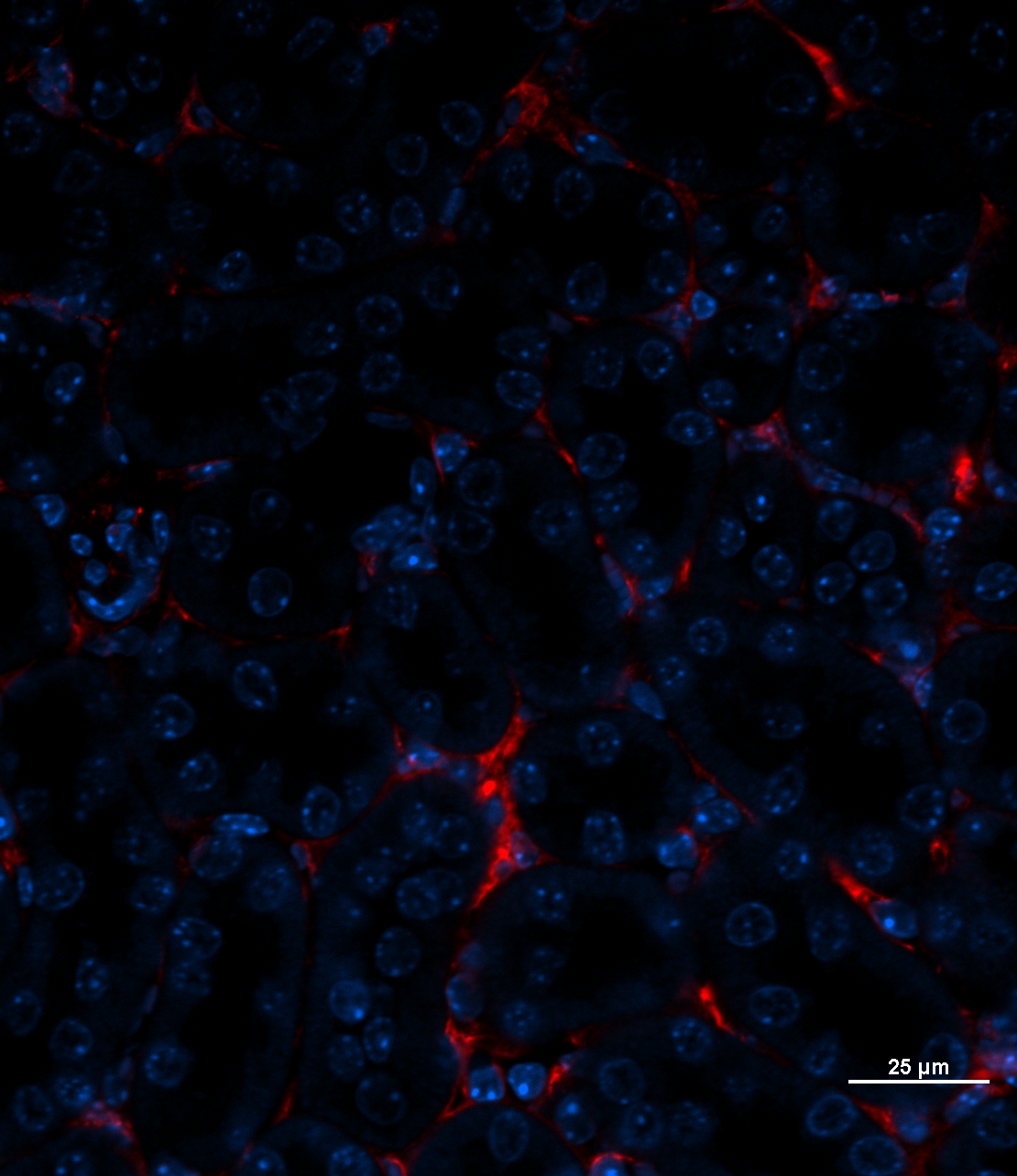

Supplement: Supplementary file 12 — Source Data Fig. 6 [file 44319_2023_19_MOESM12_ESM.zip › Fig.6/6F/Cep120-KO-sham_FN1_RGB.tif]

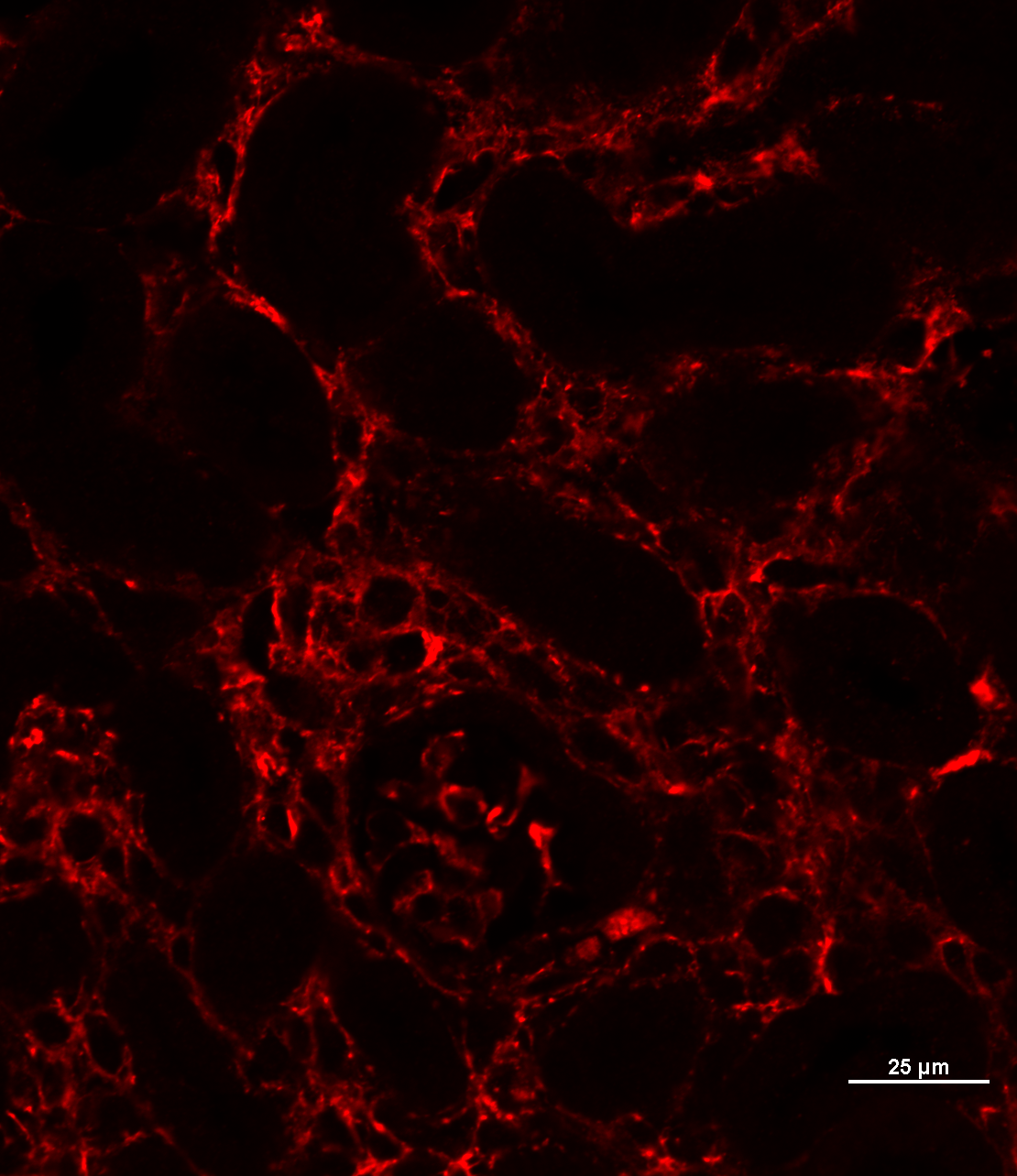

Supplement: Supplementary file 12 — Source Data Fig. 6 [file 44319_2023_19_MOESM12_ESM.zip › Fig.6/6F/Cep120-KO-UUO_FN1_RGB_488-SD.tif]

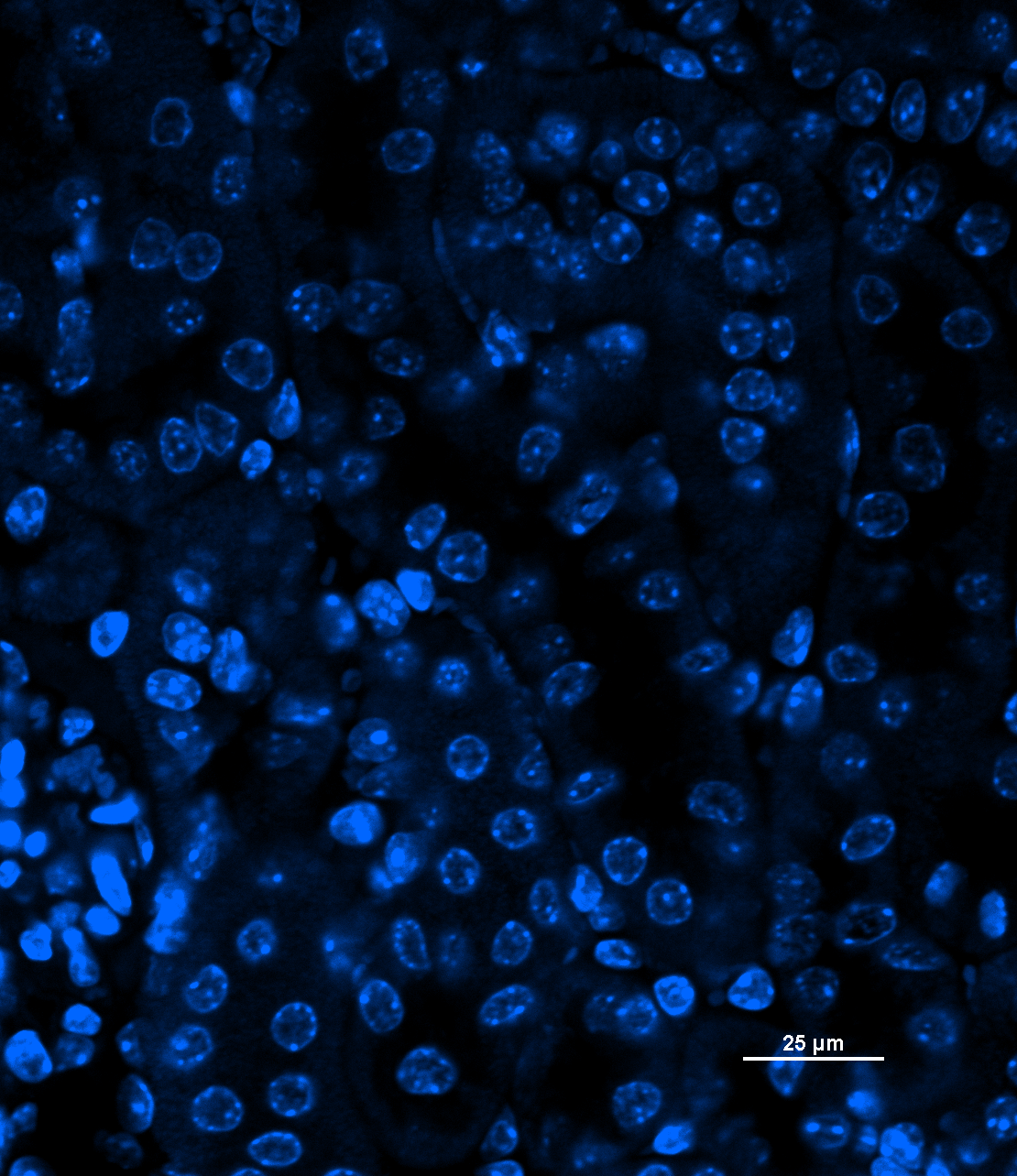

Supplement: Supplementary file 12 — Source Data Fig. 6 [file 44319_2023_19_MOESM12_ESM.zip › Fig.6/6E/Cep120-KO-sham_Des_RGB_405-SD .tif]

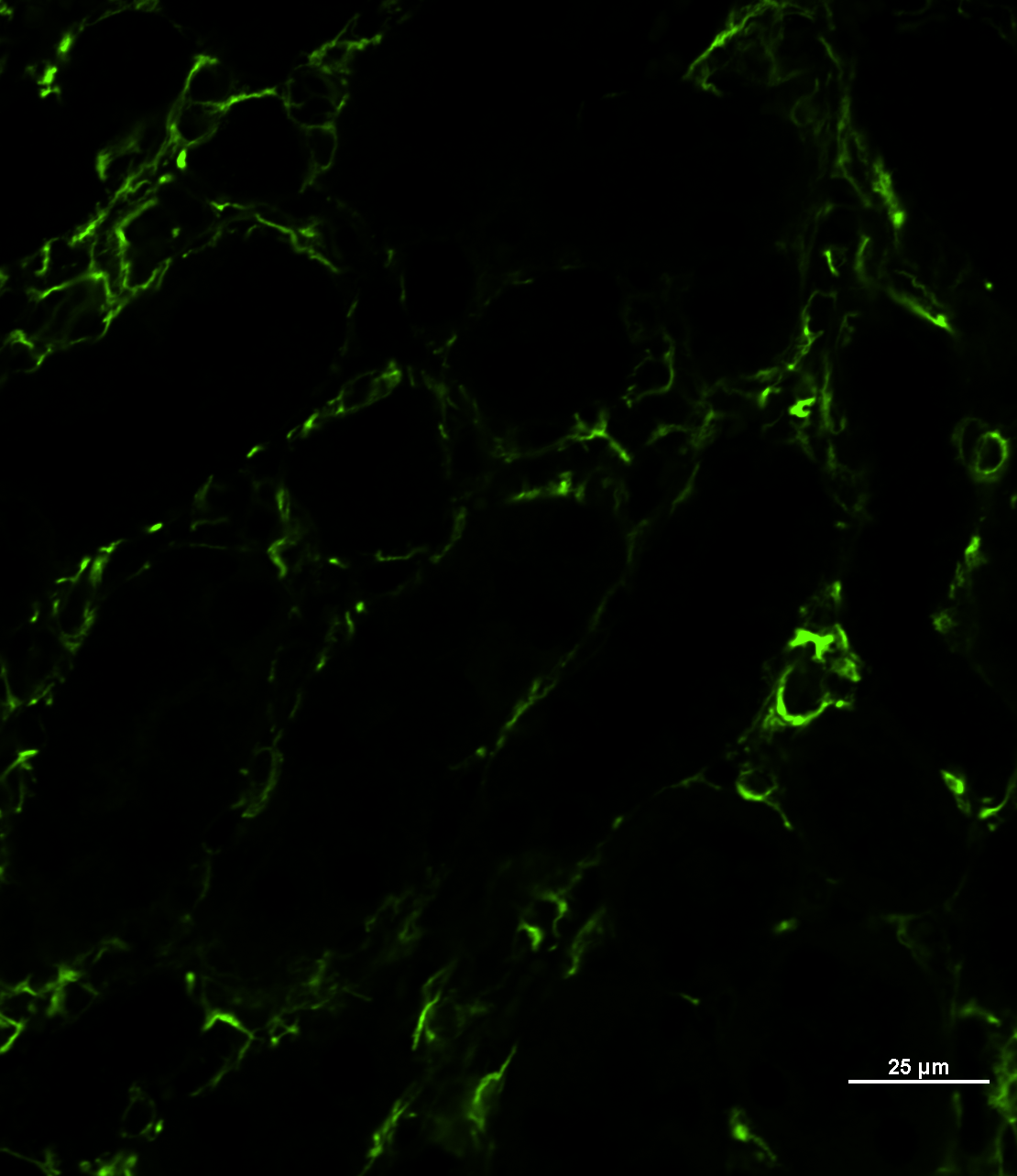

Supplement: Supplementary file 12 — Source Data Fig. 6 [file 44319_2023_19_MOESM12_ESM.zip › Fig.6/6E/Ctrl-UUO_Des_RGB_488-SD.tif]
