# Supplementary material for: Cep120 is essential for kidney stromal progenitor cell growth and differentiation
Source: EMBO Rep. 2023 Dec 20;25(1):24. doi: 10.1038/s44319-023-00019-z (PMC10897188; doi:10.1038/s44319-023-00019-z)
Supplement: Supplementary file 12 — Source Data Fig. 6 [file 44319_2023_19_MOESM12_ESM.zip › Fig.6/6B/Blots.pptx]

## Slide 1
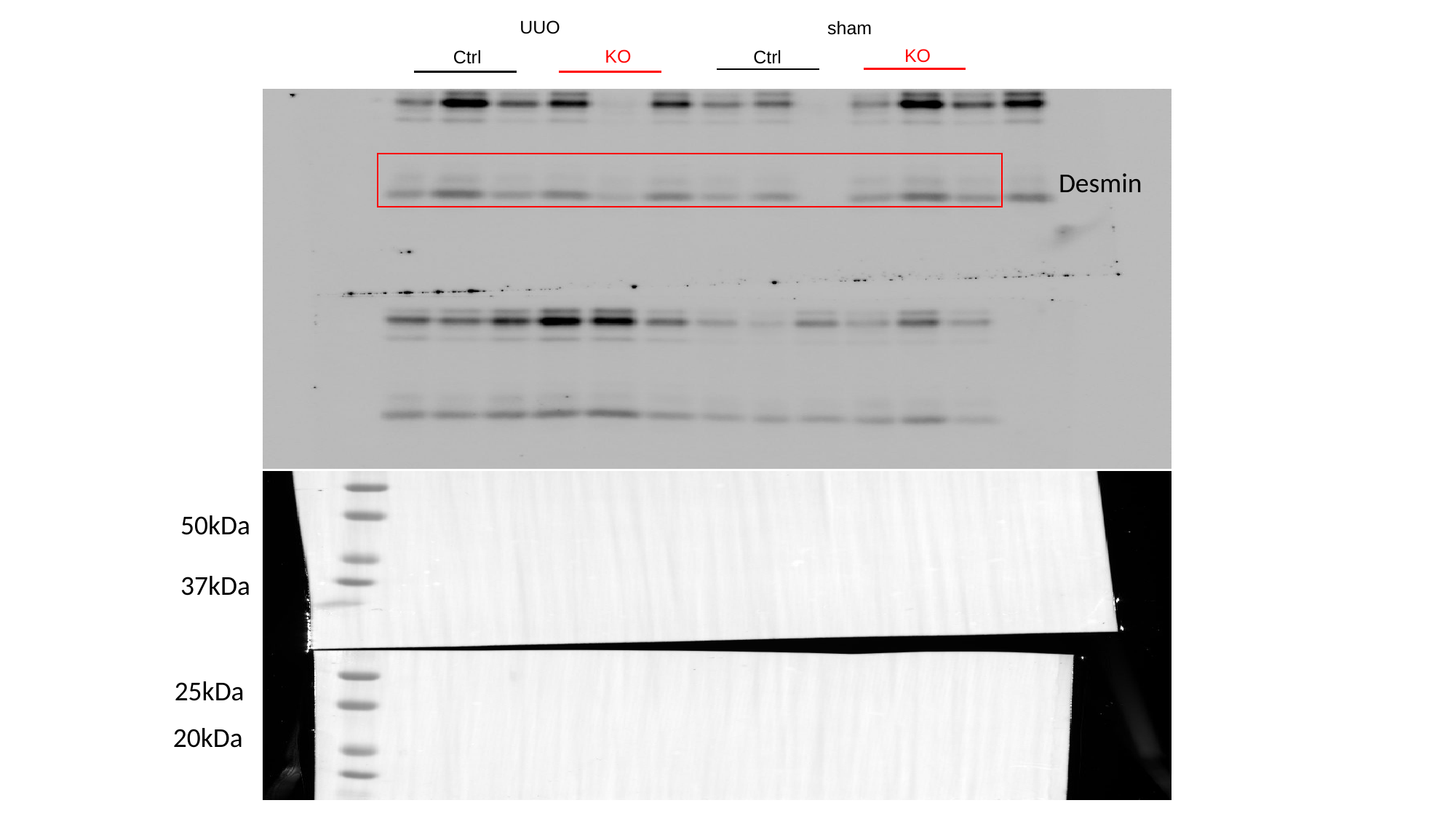

UUO
sham
KO
KO
Ctrl
Ctrl
Desmin
50kDa
37kDa
25kDa
20kDa

## Slide 2
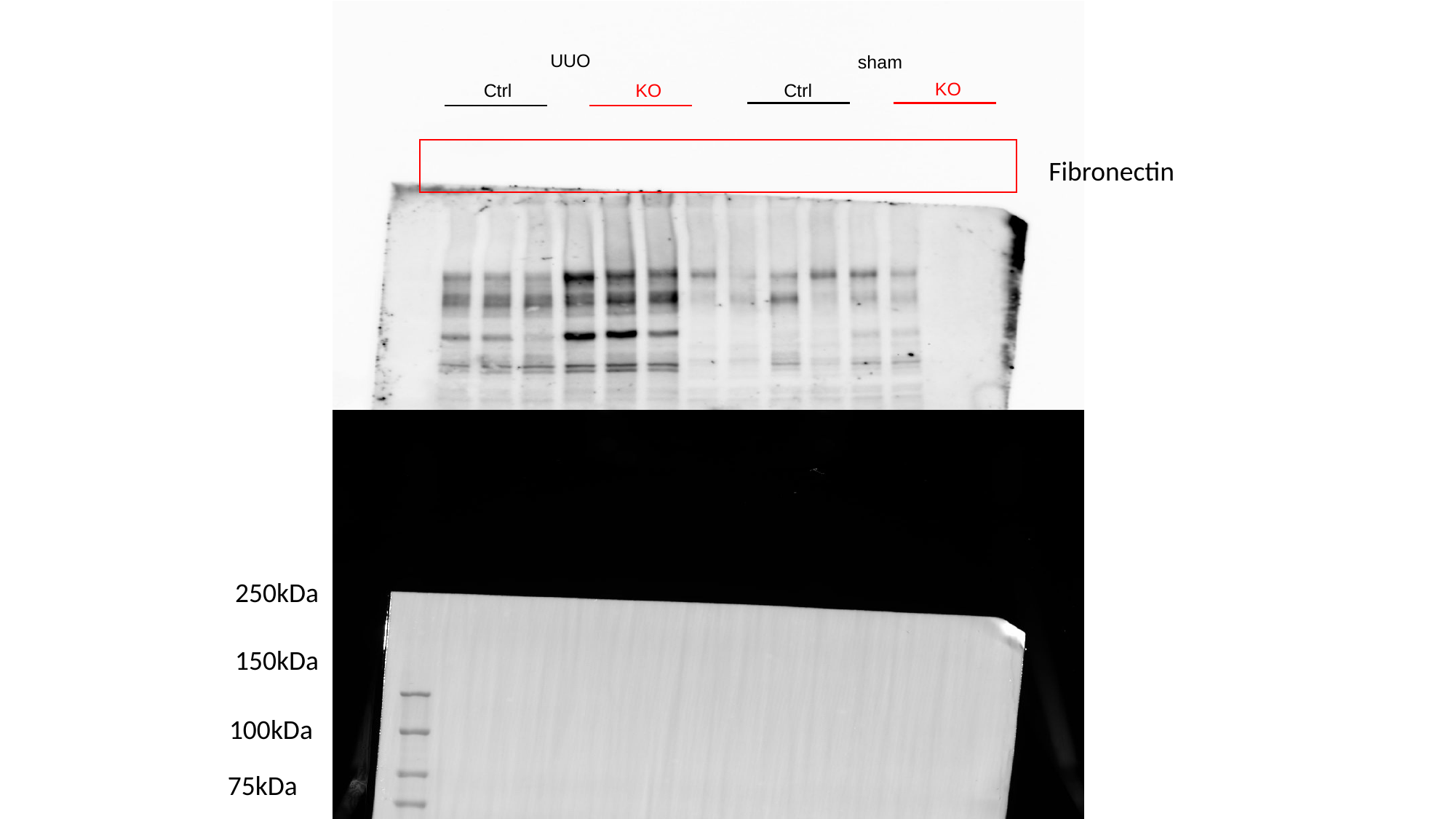

UUO
sham
KO
KO
Ctrl
Ctrl
Fibronectin
250kDa
150kDa
100kDa
75kDa

## Slide 3
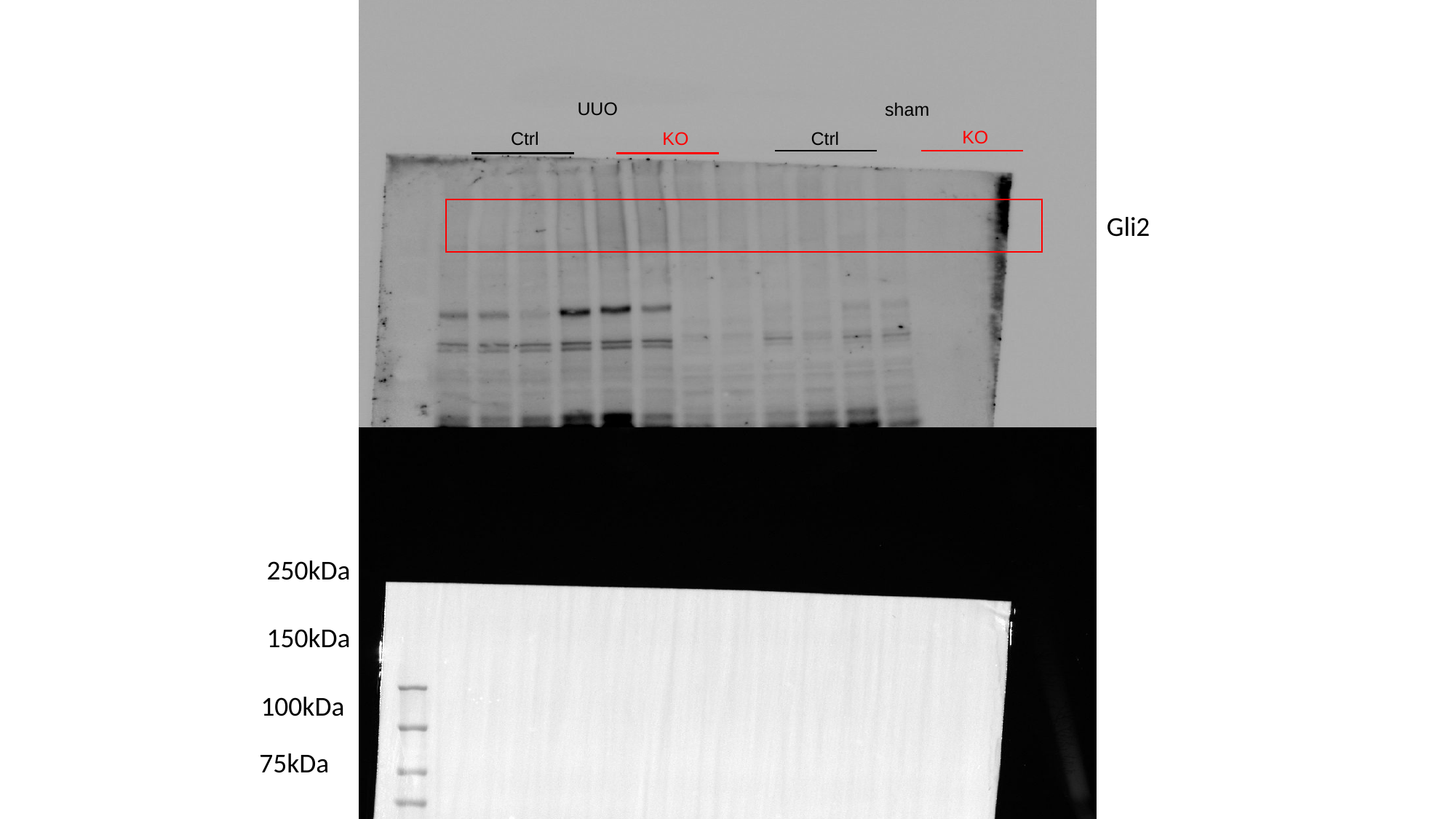

UUO
sham
KO
KO
Ctrl
Ctrl
Gli2
250kDa
150kDa
100kDa
75kDa

## Slide 4
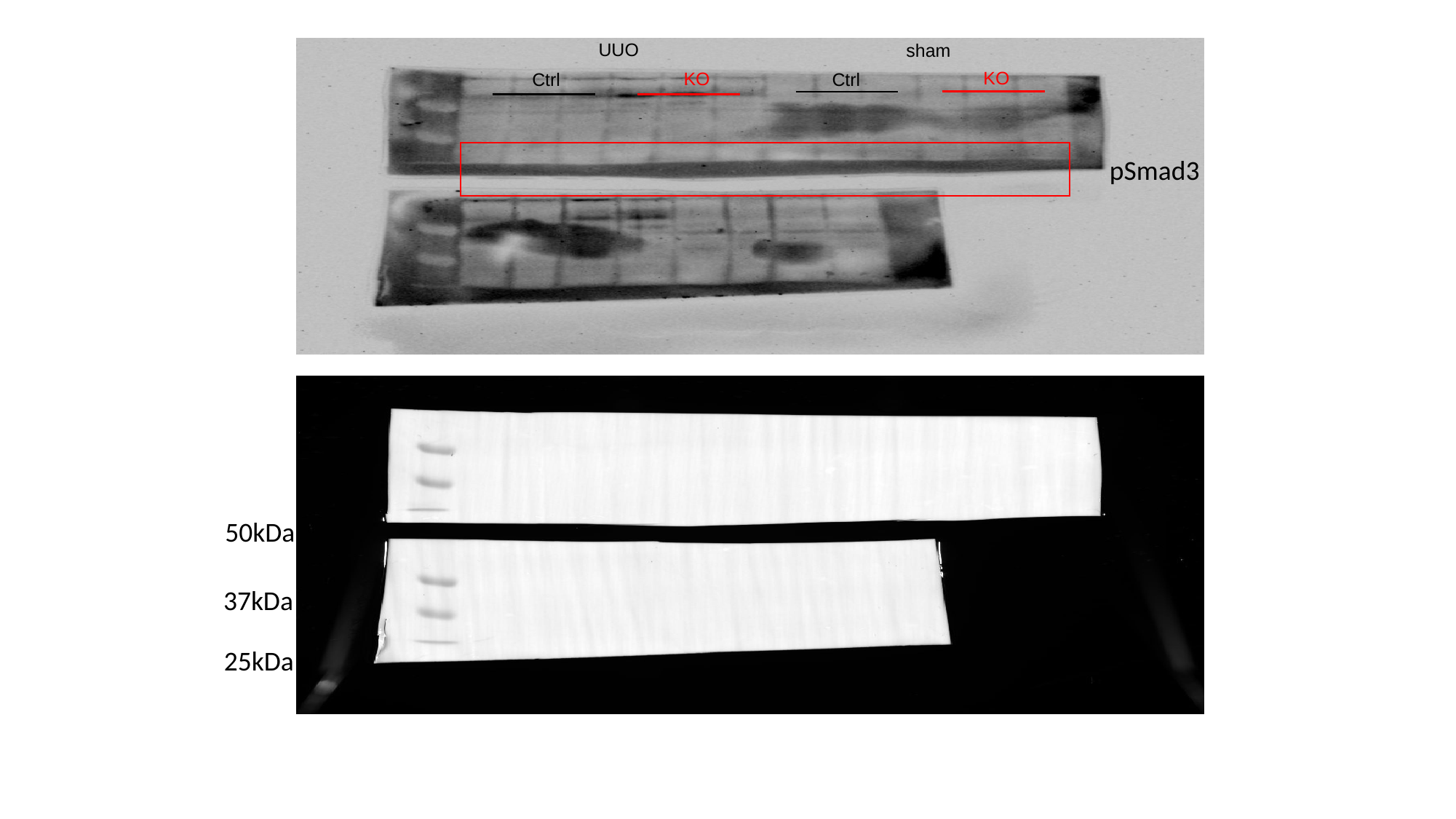

UUO
sham
KO
KO
Ctrl
Ctrl
pSmad3
50kDa
37kDa
25kDa

## Slide 5
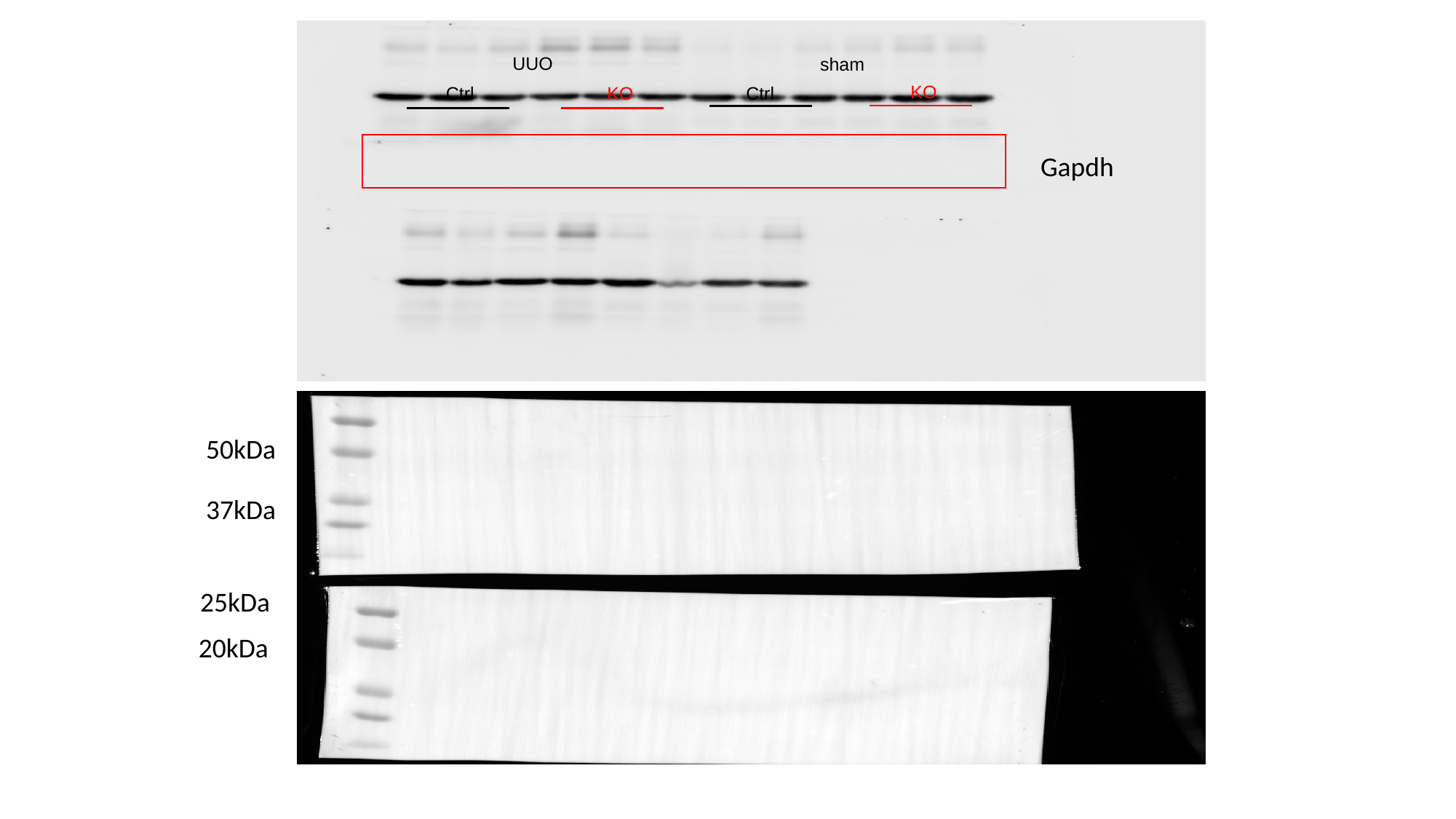

UUO
sham
KO
KO
Ctrl
Ctrl
Gapdh
50kDa
37kDa
25kDa
20kDa
