# Supplementary figures and images for: Cep120 is essential for kidney stromal progenitor cell growth and differentiation (part 2 of 2)
Source: EMBO Rep. 2023 Dec 20;25(1):24. doi: 10.1038/s44319-023-00019-z (PMC10897188; doi:10.1038/s44319-023-00019-z)

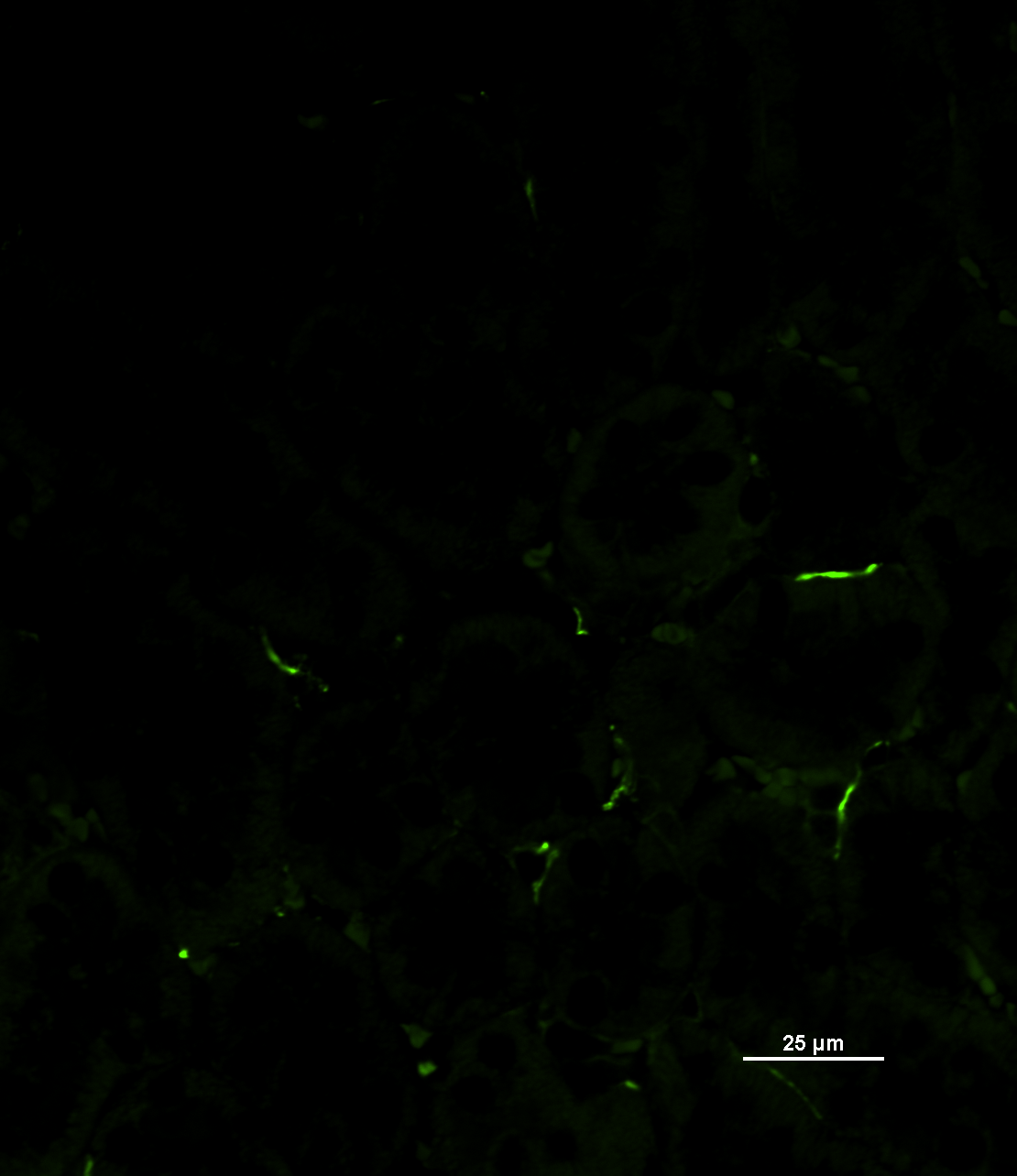

Supplement: Supplementary file 12 — Source Data Fig. 6 [file 44319_2023_19_MOESM12_ESM.zip › Fig.6/6E/Ctrl-sham_Des_RGB_488-SD.tif]

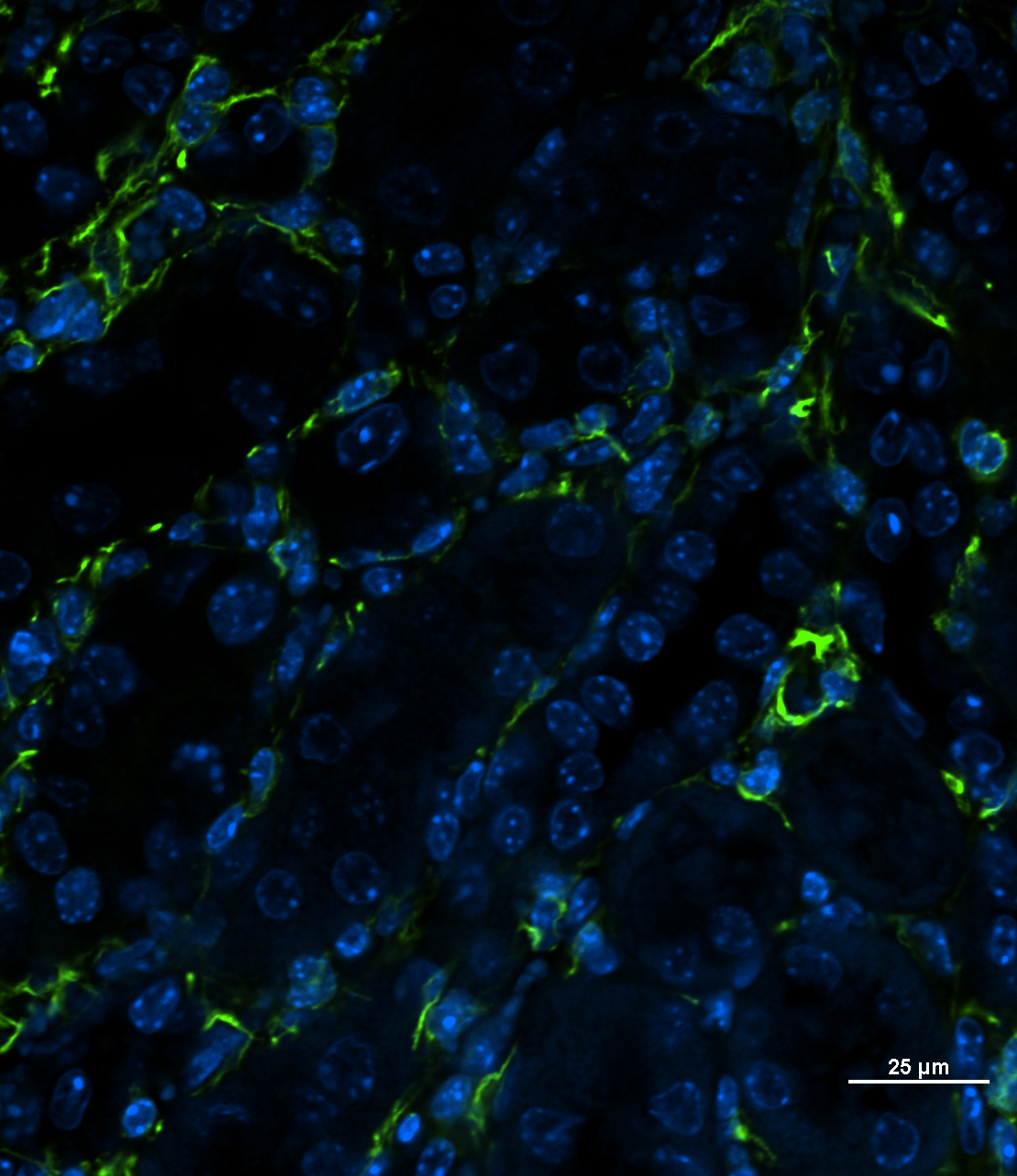

Supplement: Supplementary file 12 — Source Data Fig. 6 [file 44319_2023_19_MOESM12_ESM.zip › Fig.6/6E/Ctrl-UUO_Des_RGB.tif]

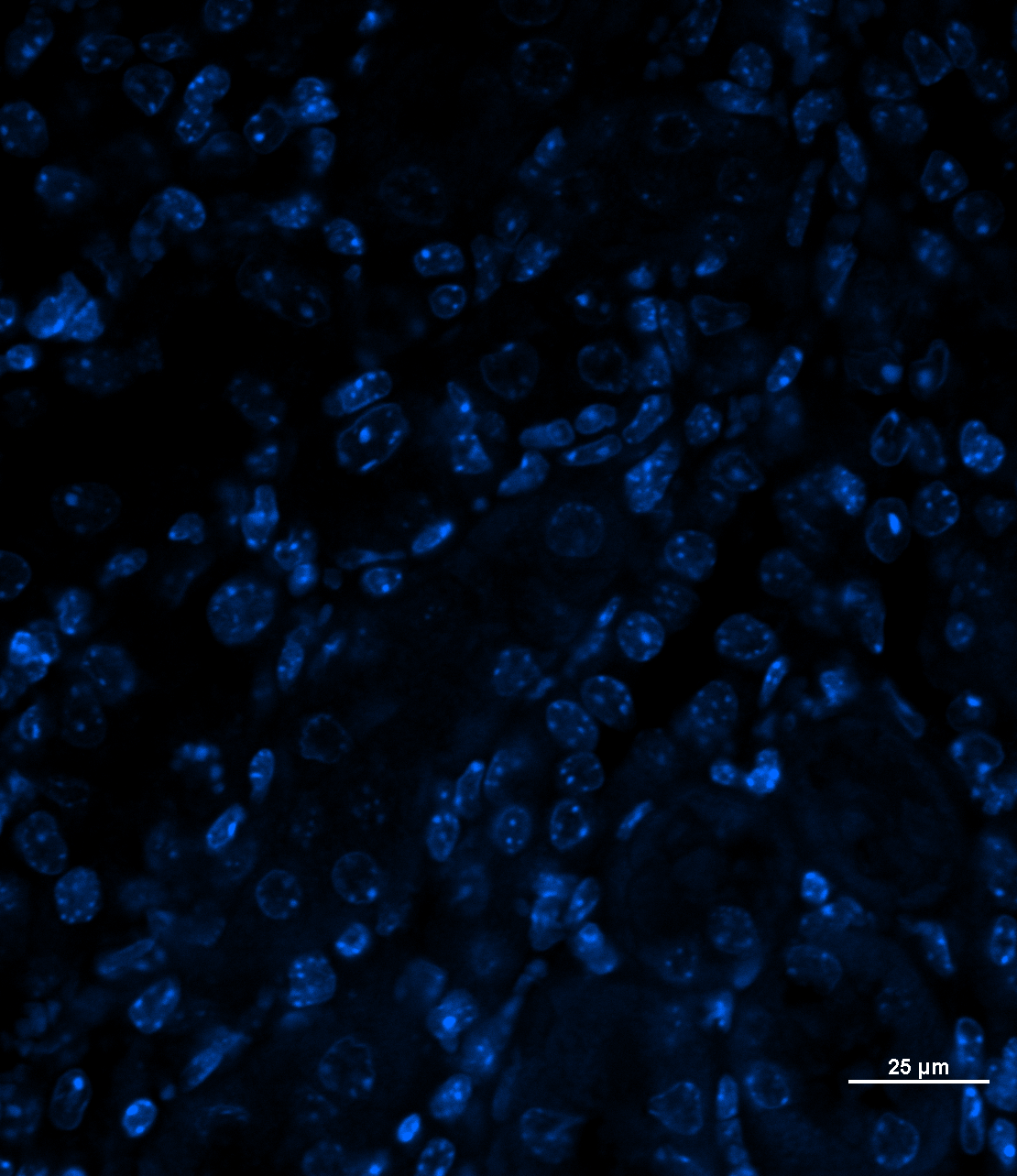

Supplement: Supplementary file 12 — Source Data Fig. 6 [file 44319_2023_19_MOESM12_ESM.zip › Fig.6/6E/Ctrl-UUO_Des_RGB_405-SD .tif]

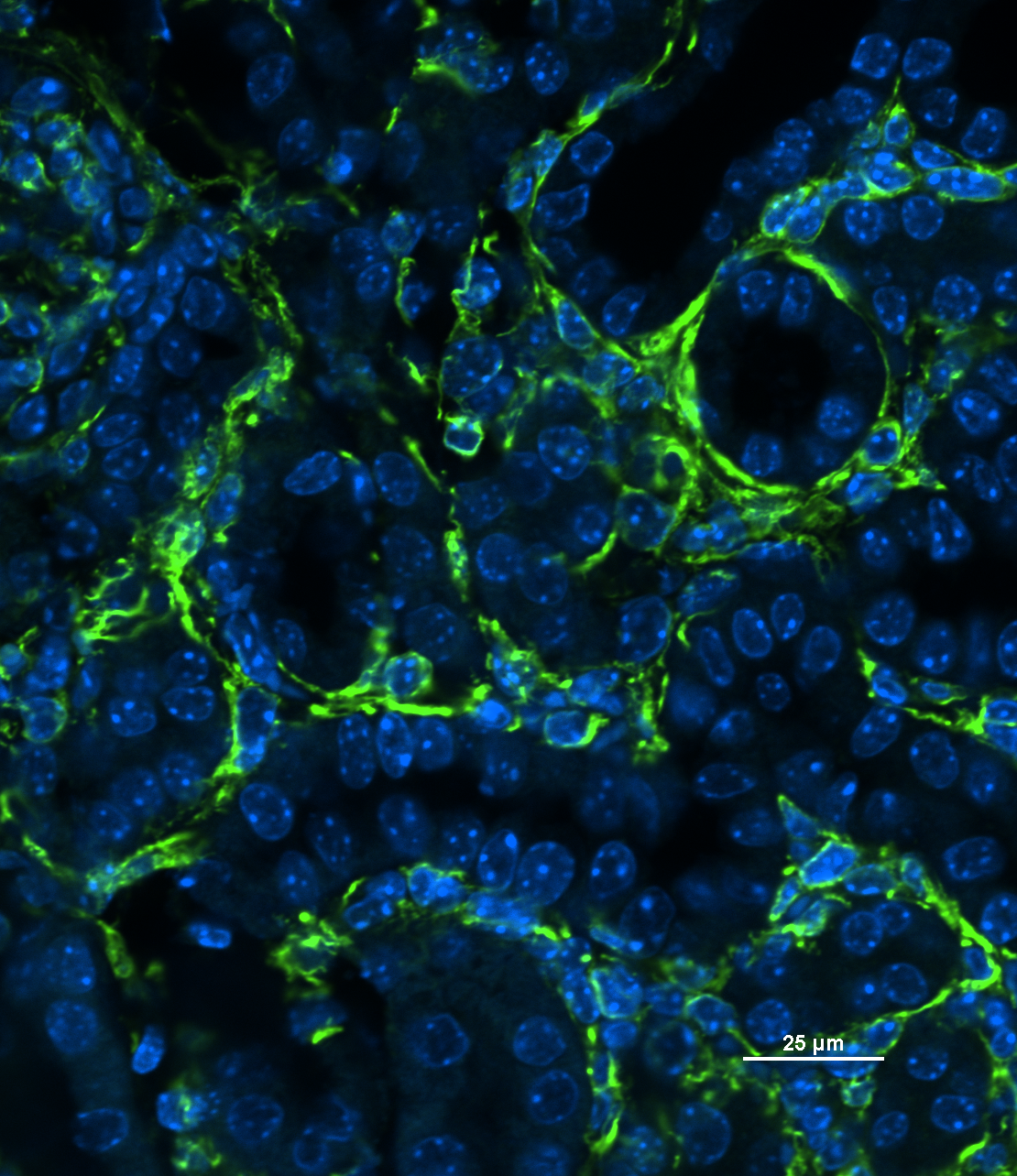

Supplement: Supplementary file 12 — Source Data Fig. 6 [file 44319_2023_19_MOESM12_ESM.zip › Fig.6/6E/Cep120-KO-UUO_Des_RGB.tif]

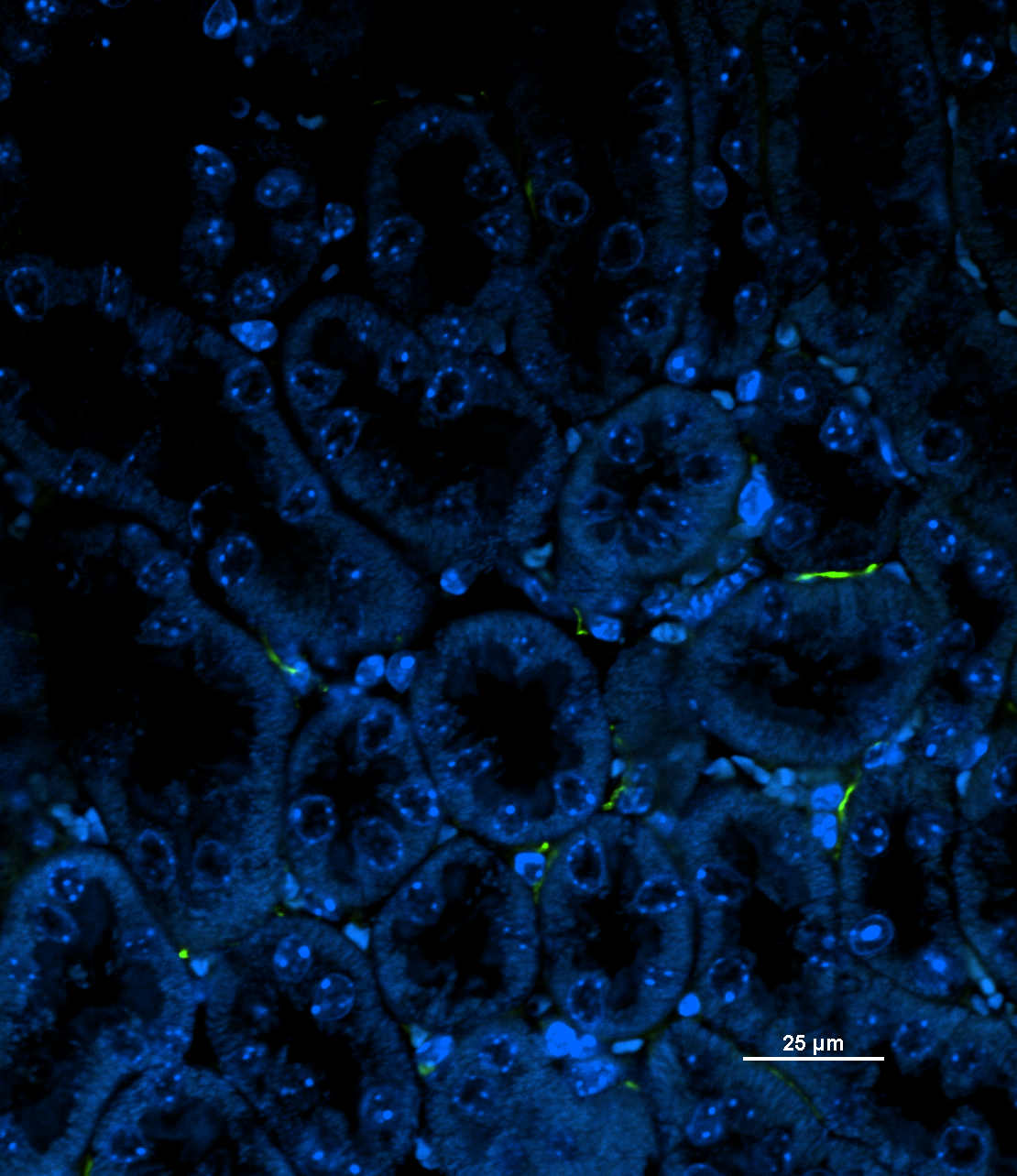

Supplement: Supplementary file 12 — Source Data Fig. 6 [file 44319_2023_19_MOESM12_ESM.zip › Fig.6/6E/Ctrl-sham_Des_RGB.tif]

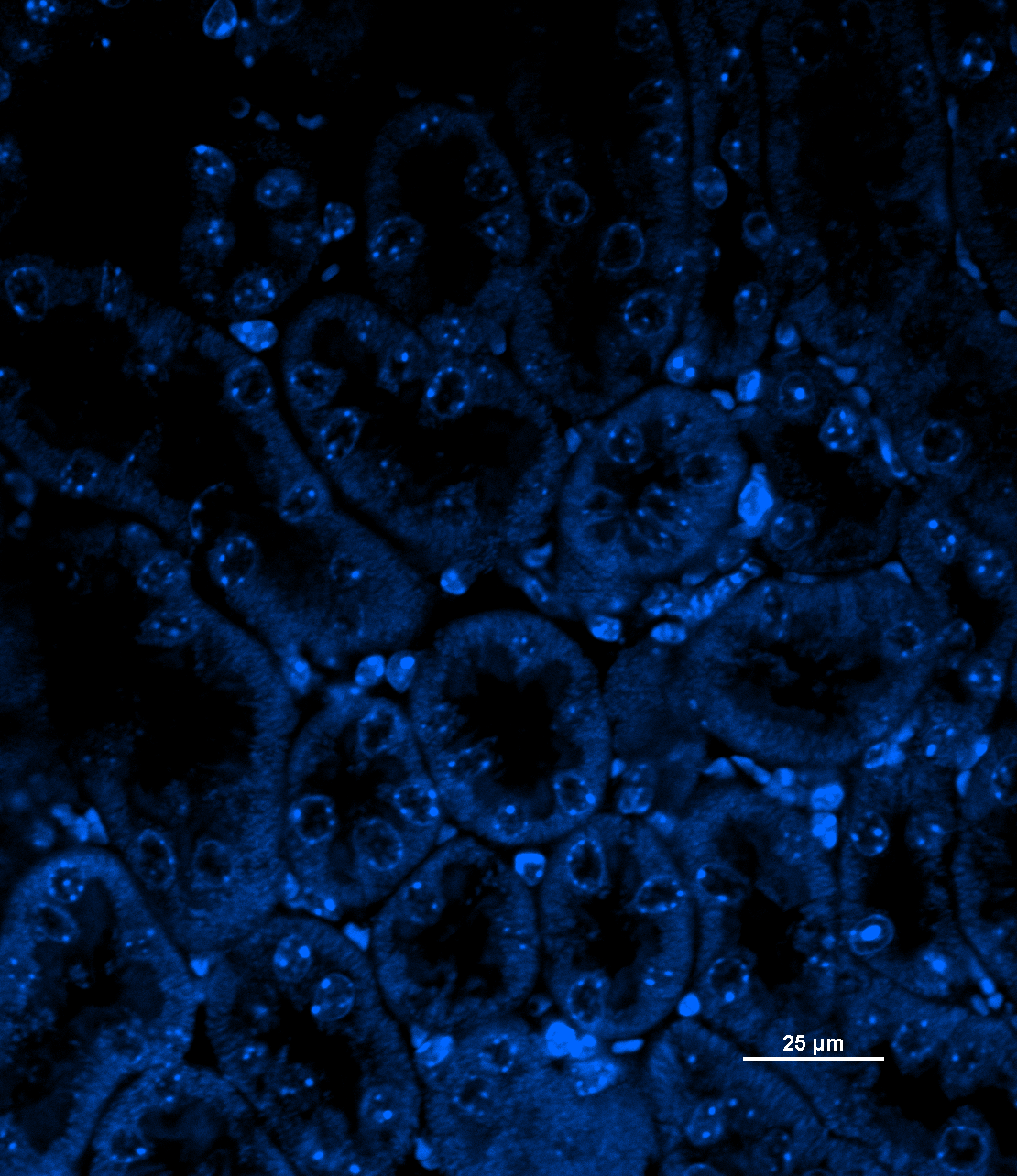

Supplement: Supplementary file 12 — Source Data Fig. 6 [file 44319_2023_19_MOESM12_ESM.zip › Fig.6/6E/Ctrl-sham_Des_RGB_405-SD .tif]

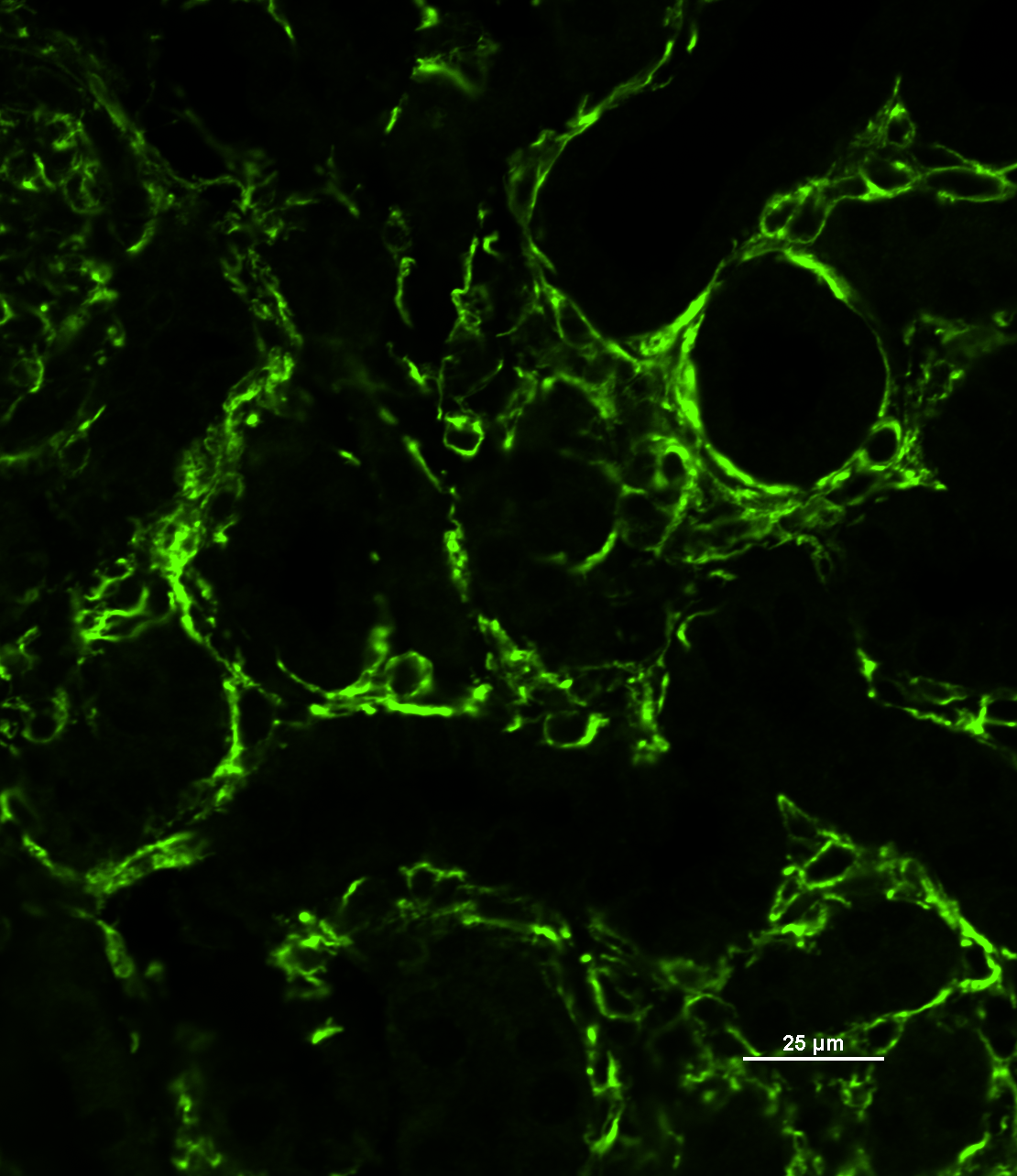

Supplement: Supplementary file 12 — Source Data Fig. 6 [file 44319_2023_19_MOESM12_ESM.zip › Fig.6/6E/Cep120-KO-UUO_Des_RGB_488-SD.tif]

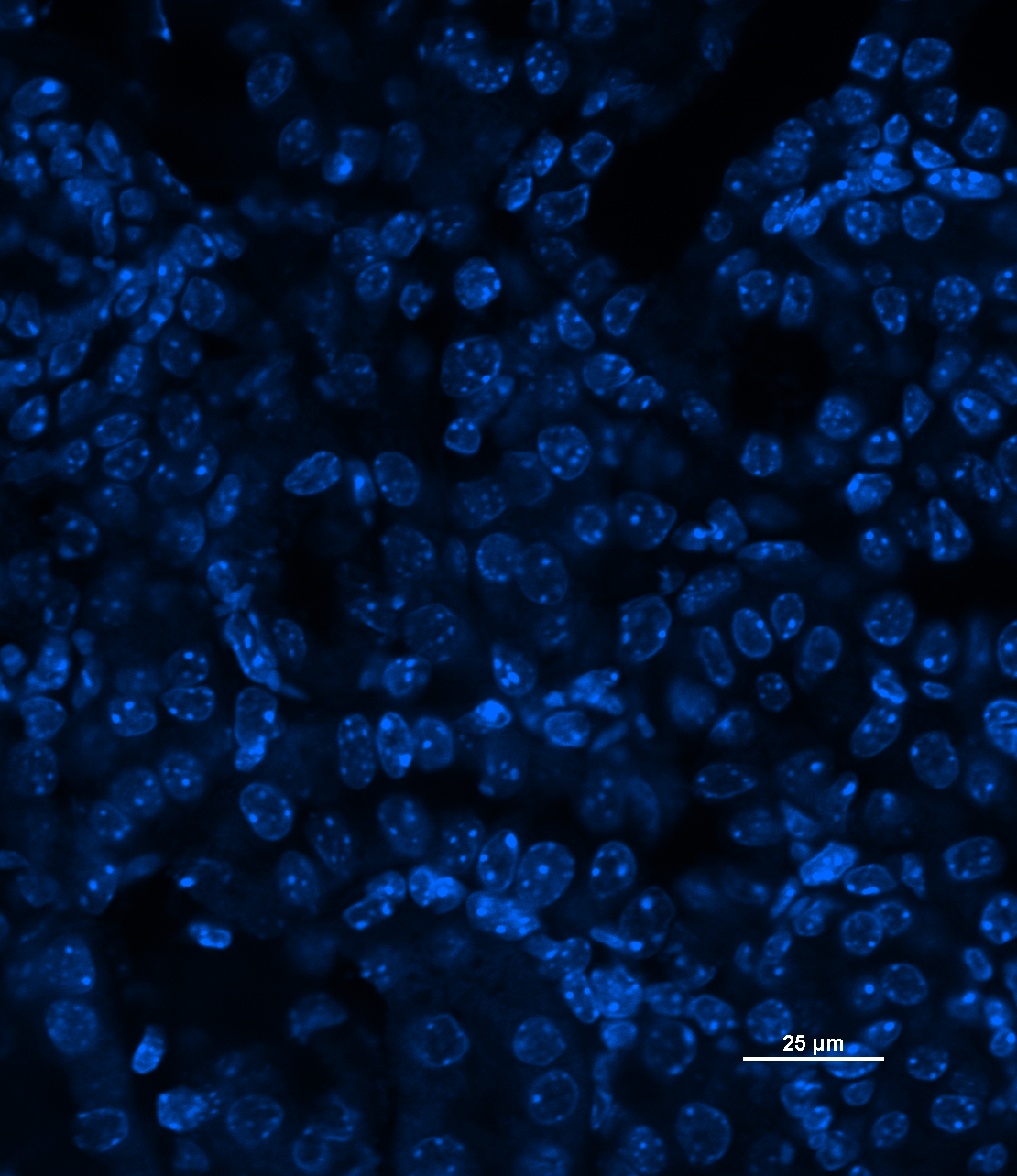

Supplement: Supplementary file 12 — Source Data Fig. 6 [file 44319_2023_19_MOESM12_ESM.zip › Fig.6/6E/Cep120-KO-UUO_Des_RGB_405-SD .tif]

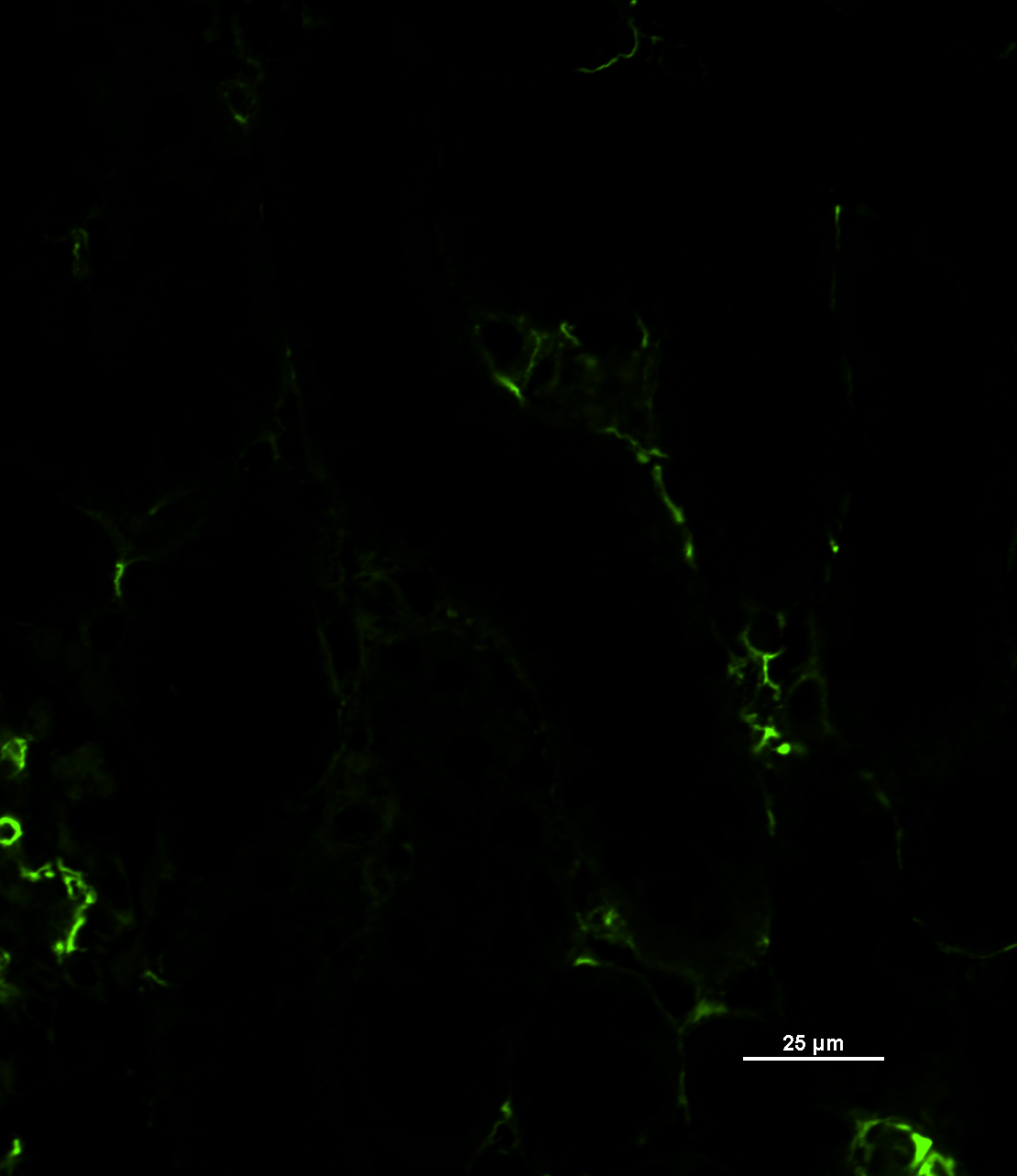

Supplement: Supplementary file 12 — Source Data Fig. 6 [file 44319_2023_19_MOESM12_ESM.zip › Fig.6/6E/Cep120-KO-sham_Des_RGB_488-SD.tif]

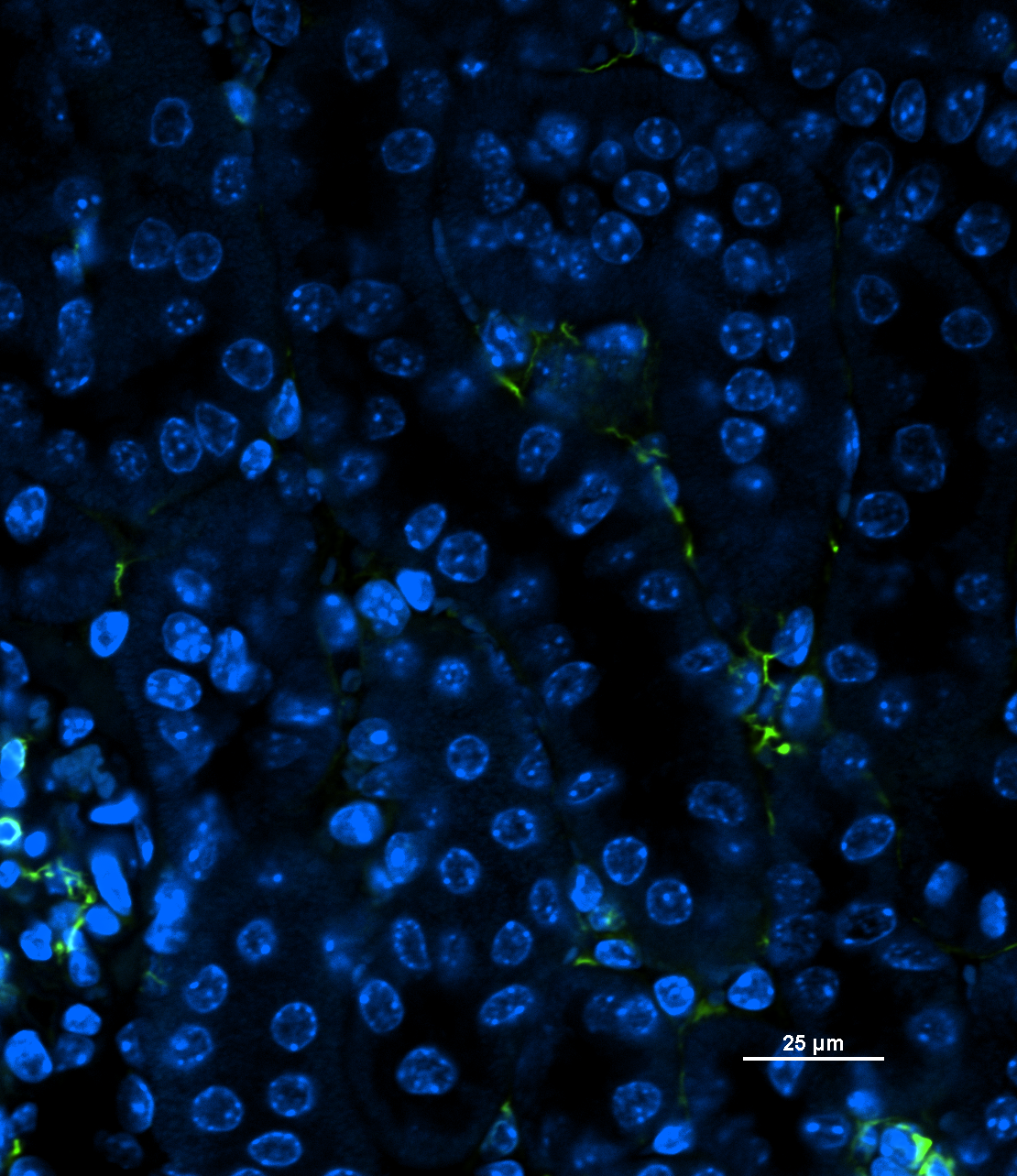

Supplement: Supplementary file 12 — Source Data Fig. 6 [file 44319_2023_19_MOESM12_ESM.zip › Fig.6/6E/Cep120-KO-sham_Des_RGB.tif]
