# Supplementary material for: Cep120 is essential for kidney stromal progenitor cell growth and differentiation
Source: EMBO Rep. 2023 Dec 20;25(1):24. doi: 10.1038/s44319-023-00019-z (PMC10897188; doi:10.1038/s44319-023-00019-z)
Supplement: Supplementary file 13 — Expanded View Figures [file 44319_2023_19_MOESM13_ESM.pdf]

Expanded View Figures

Figure EV1. Cep120 depletion in stromal progenitors causes centrosome loss in the derived cell types.

(**A**) Immunofluorescence staining of kidney sections from control and Cep120-KO mice at P15 with antibodies to mark Cep120, centrosomes (γ-tubulin) and pericytes (PDGFR-β). (**B**, **C**) Quantification of the percentage of PDGFR-β-positive pericytes with (**B**) Cep120 expression and (**C**) centrosomes at P15. $N = 680$ cells (Ctrl) and $N = 421$ (Cep120-KO). (**D**) Immunofluorescence staining of P15 kidney sections with antibodies to mark Cep120, mesangial cells (GATA3) and podocytes (synaptopodin). (**E**, **F**) Quantification of the percentage of GATA3-positive mesangial cells expressing (**E**) Cep120 and (**F**) Cep135 (centrosomes) at P15. (**E**) $N = 1079$ cells (Ctrl) and $N = 817$ (Cep120-KO). (**F**) $N = 720$ cells (Ctrl) and $N = 696$ (Cep120-KO). (**G**, **H**) Quantification of the percentage of α-SMA-positive vascular smooth muscle cells (VSMC) expressing (**G**) Cep120 and (**H**) Cep135 at P15. (**G**) $N = 1025$ cells (Ctrl) and $N = 881$ (Cep120-KO). (**H**) $N = 576$ cells (Ctrl) and $N = 588$ (Cep120-KO). (**I**, **J**) Quantification of the percentage of CD31-positive endothelial cells (EC) expressing Cep120 (**I**) and Cep135 (**J**). (**I**) $N = 764$ cells (Ctrl) and $N = 572$ (Cep120-KO). (**J**) $N = 515$ cells (Ctrl) and $N = 620$ (Cep120-KO). Data information: $N \geq 5$ mice per group. A two-tailed unpaired t test was used for analyses and p value denoted as follows: ***$p < 0.001$, ****$p < 0.0001$. The vertical segments in the box plots show the first quartile, median, and third quartile. The whiskers on both ends represents the maximum and minimum for each dataset analyzed.

© The Author(s)

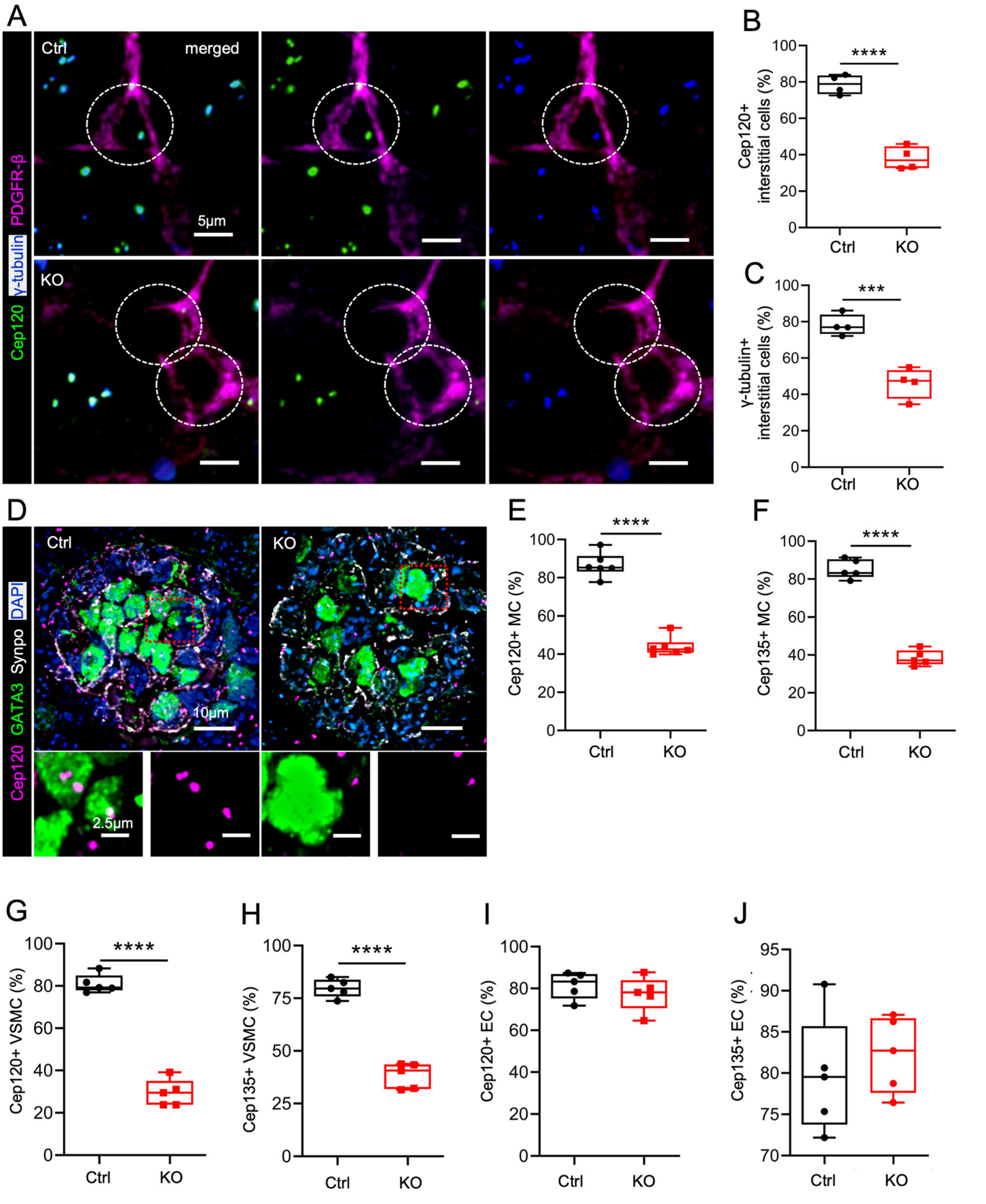
A
Ctrl
merged
5μm
KO
Cep120 γ-tubulin PDGFR-β
B
Cep120+ interstitial cells (%)
****
Ctrl
KO
C
γ-tubulin+ interstitial cells (%)
***
D
Ctrl
KO
10μm
2.5μm
Cep120 GATA3 Synpo DAPI
E
Cep120+ MC (%)
****
F
Cep135+ MC (%)
****
G
Cep120+ VSMC (%)
****
H
Cep135+ VSMC (%)
****
I
Cep120+ EC (%)
J
Cep135+ EC (%)

 © The Author(s)

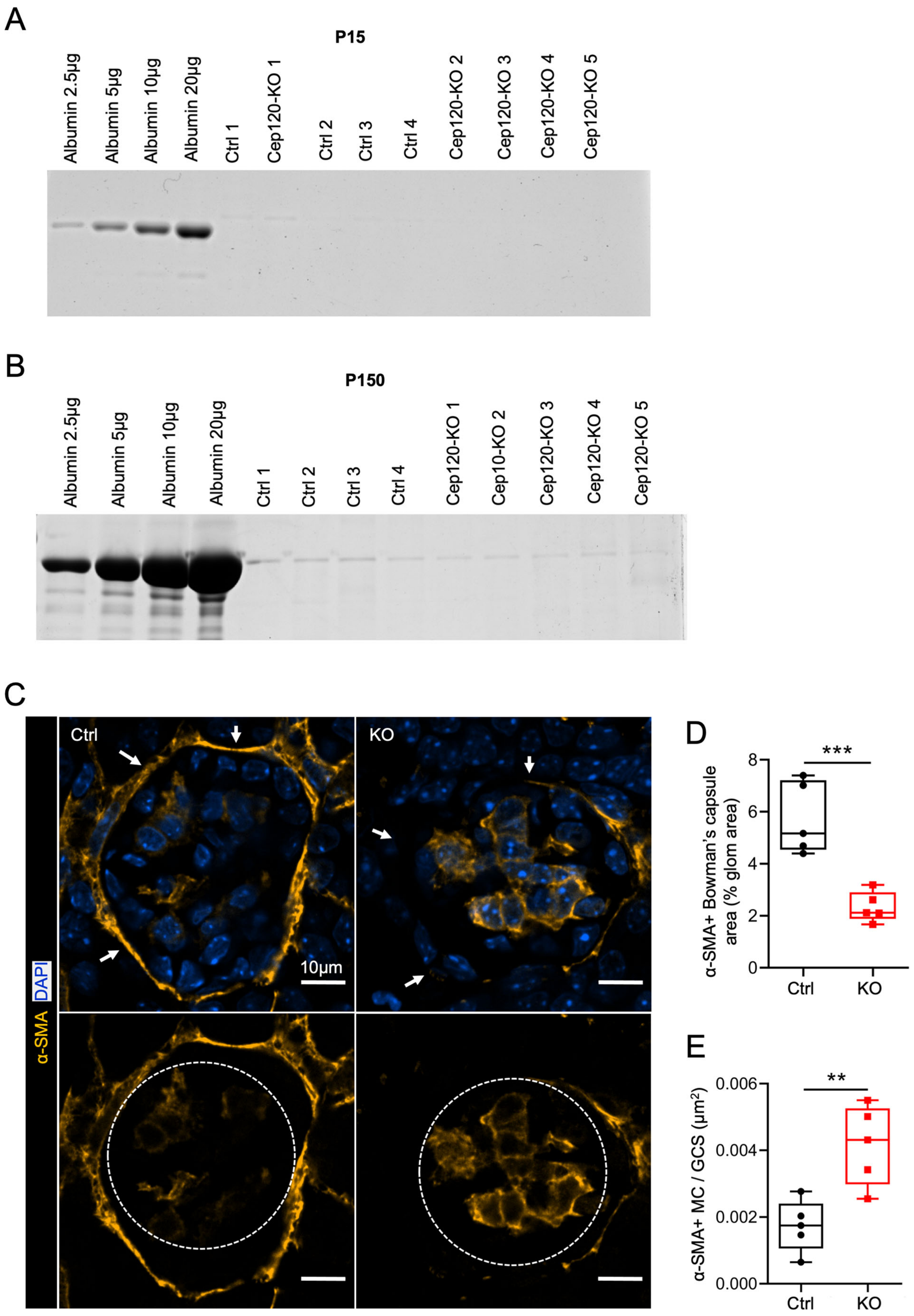
A
P15
Albumin 2.5µg
Albumin 5µg
Albumin 10µg
Albumin 20µg
Ctrl 1
Cep120-KO 1
Ctrl 2
Ctrl 3
Ctrl 4
Cep120-KO 2
Cep120-KO 3
Cep120-KO 4
Cep120-KO 5
B
P150
Albumin 2.5µg
Albumin 5µg
Albumin 10µg
Albumin 20µg
Ctrl 1
Ctrl 2
Ctrl 3
Ctrl 4
Cep120-KO 1
Cep10-KO 2
Cep120-KO 3
Cep120-KO 4
Cep120-KO 5
C
α-SMA DAPI
Ctrl
KO
10µm
D
α-SMA+ Bowman's capsule area (% glom area)
***
8
6
4
2
0
Ctrl
KO
E
α-SMA+ MC / GCS (µm²)
**
0.006
0.004
0.002
0.000
Ctrl
KO

Figure EV2. Cep120 and centrosome loss causes defects in FoxD1-derived pericytes surrounding the Bowman's capsule and glomerular mesangial cells.

(**A**, **B**) Coomassie gel of urine samples from control and Cep120-KO mice at (**A**) P15 and (**B**) P150. Bovine serum albumin (BSA) standards (2.5, 5, 10 and 20 μg) were also run on both gels. (**C**) Immunofluorescence staining of P15 kidney sections with anti α-smooth muscle actin (α-SMA) antibodies to mark pericytes surrounding Bowman's capsule (marked with white arrows; upper panel) and mesangial cells (lower panel). (**D**) Quantification of α-SMA-positive Bowman's capsule area expressed as percentage of total glomerular area. $N = 65$ glomeruli (Ctrl) and $N = 58$ (Cep120-KO). (**E**) Quantification of α-SMA-positive mesangial cell density per glomerular cross-sectional area. $N = 303$ cells (Ctrl) and $N = 613$ (Cep120-KO). Data information: $N = 5$ mice per group. A two-tailed unpaired *t* test was used for analyses and *p* value denoted as follows: $^{**}p < 0.01$, $^{***}p < 0.001$. The vertical segments in the box plots show the first quartile, median, and third quartile. The whiskers on both ends represents the maximum and minimum for each dataset analyzed.

 © The Author(s)

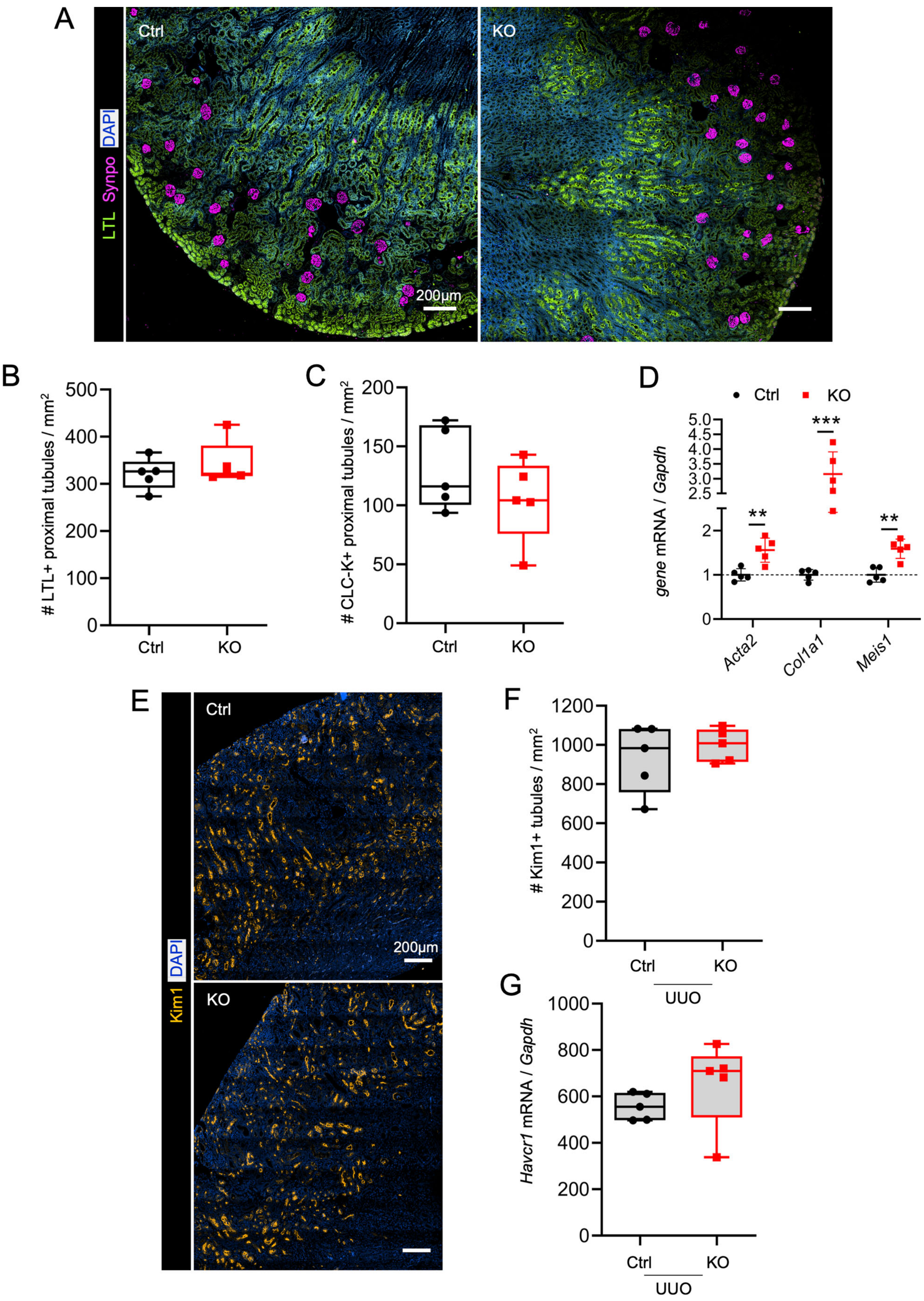
A
Ctrl
KO
LTL Synpo DAPI
200µm
B
LTL+ proximal tubules / mm²
500
400
300
200
100
0
Ctrl
KO
C
CLC-K+ proximal tubules / mm²
200
150
100
50
0
Ctrl
KO
D
Ctrl
KO
gene mRNA / Gapdh
5.0
4.5
4.0
3.5
3.0
2.5
2
1
0
**
***
**
Acta2
Col1a1
Meis1
E
Ctrl
KO
Kim1 DAPI
200µm
F
Kim1+ tubules / mm²
1200
1000
800
600
400
200
0
Ctrl
KO
UUO
G
Havcr1 mRNA / Gapdh
1000
800
600
400
200
0
Ctrl
KO
UUO

Figure EV3. Analysis of nephron number and maturation, as well as transcriptional expression of profibrotic factors in long-term Cep120-KO survivors.

(**A**) Immunofluorescence staining of P150 control and Cep120-KO kidney sections with antibodies to mark glomeruli (synaptopodin) and proximal tubules (LTL). (**B**, **C**) Quantification of LTL-positive proximal tubules and CLC-K-positive distal tubules per unit area. (**B**) $N = 1457$ tubules (Ctrl) and N = 1557 (Cep120-KO). (**C**) $N = 598$ tubules (Ctrl) and $N = 477$ (Cep120-KO). (**D**) qPCR-based quantification of the relative change in gene expression levels of Acta2, Col1a1 and Meis1 in control and Cep120-KO kidneys at P150. (**E**) Immunofluorescence staining of P60 kidney sections with antibodies to kidney injury marker 1 (Kim1) in control and Cep120-KO mice following UUO injury. (**F**) Quantification of Kim1-positive tubule number per unit area. $N = 938$ tubules (Ctrl) and $N = 991$ (Cep120-KO). (**G**) qPCR-based quantification of the relative change in gene expression levels of Havcr1 in control and Cep120-KO kidneys after 7 days of injury (UUO) and without injury (sham), expressed as fold change of sham-control. UUO was performed in 2 months old mice. Data information: $N = 5$ mice per group. A two-tailed unpaired t test was used for analyses, and p-value denoted as follows: $^{**}p < 0.01$, $^{***}p < 0.001$. The vertical segments in the box plots show the first quartile, median, and third quartile. The whiskers on both ends represents the maximum and minimum for each dataset analyzed.

 © The Author(s)

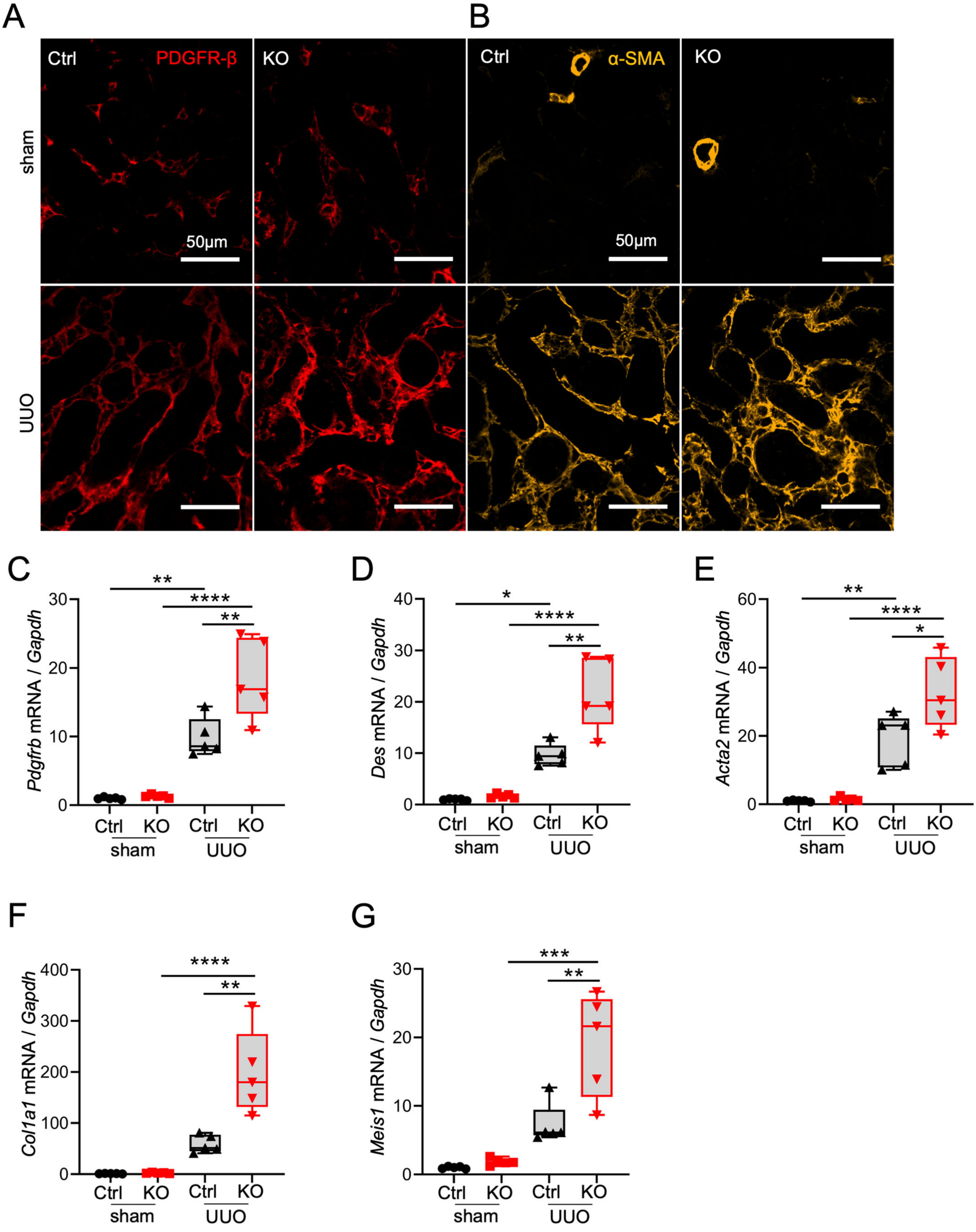
A
B
Ctrl
PDGFR-β
KO
Ctrl
α-SMA
KO
sham
UUO
50µm
50µm
C
D
E
F
G
Pdgfrb mRNA / Gapdh
Des mRNA / Gapdh
Acta2 mRNA / Gapdh
Col1a1 mRNA / Gapdh
Meis1 mRNA / Gapdh
Ctrl KO Ctrl KO
sham UUO
**
****
*
***

Figure EV4. Defective centrosome biogenesis in the stroma accelerates injury-induced fibrosis.

(**A**, **B**) Immunofluorescence staining of P60 kidney sections with antibodies to mark (**A**) pericytes/fibroblasts (PDGFR-β) and (**B**) myofibroblasts (α-SMA) in sham (upper panel) and UUO kidneys (lower panel). (**C–G**) qPCR-based quantification of the relative change in gene expression levels of (**C**) Pdgfrb, (**D**) desmin, (**E**) Acta2, (**F**) Col1a1, and (**G**) Meis1 in control and Cep120-KO kidneys after 7 days of injury (UUO) and without (sham), expressed as fold change relative to sham-control. Data information: $N = 5$ mice per group. A one-way ANOVA test followed by multiple-group comparison analysis with Tukey correction was used for analyses, and p-value denoted as follows: *$p < 0.05$, **$p < 0.01$, ***$p < 0.001$, ****$p < 0.0001$. The vertical segments in the box plots show the first quartile, median, and third quartile. The whiskers on both ends represents the maximum and minimum for each dataset analyzed.

 © The Author(s)

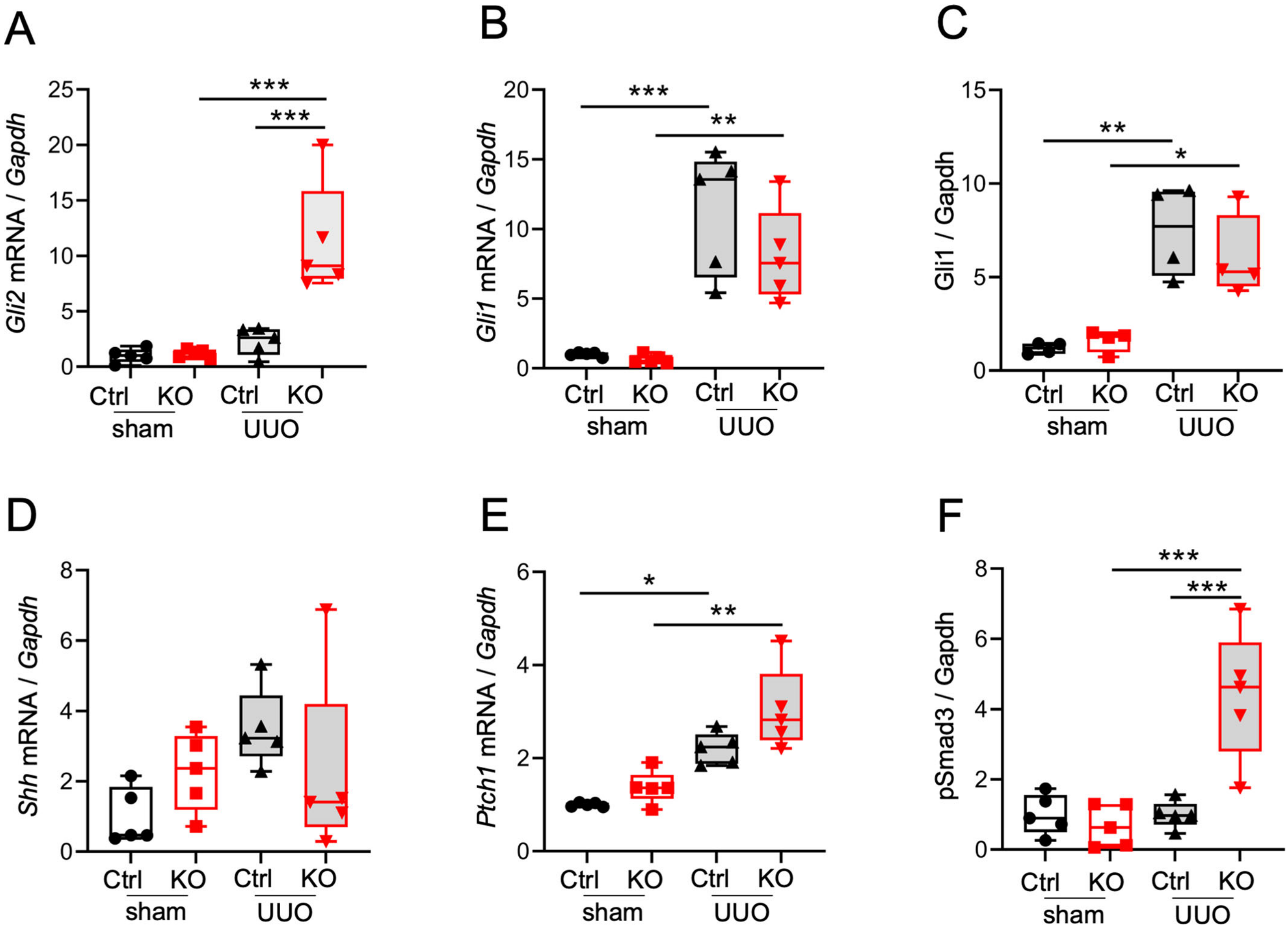

Figure EV5. Quantification of signaling factors implicated in injury-induced fibrosis.

(**A**, **B**) qPCR-based quantification of the relative change in gene expression levels of (**A**) Gli2 and (**B**) Gli1 in control and Cep120-KO kidneys after 7 days of injury (UUO) and without injury (sham), expressed as fold change relative to sham-control. (**C**) Quantification of Gli1 protein levels normalized to Gapdh, expressed as fold change relative to sham-control. (**D**, **E**) qPCR-based quantification of the change in gene expression levels of (**D**) Shh and (**E**) Ptch1 in control and Cep120-KO kidneys after 7 days of injury (UUO) and without (sham), expressed as fold change relative to sham-control. (**F**) Quantification of pSmad3 protein levels normalized to Gapdh, expressed as fold change of sham-control. Data information: $N = 5$ mice per group. A one-way ANOVA test followed by multiple-group comparison analysis with Tukey correction was used for analyses, and p value denoted as follows: *$p < 0.05$, **$p < 0.01$, ***$p < 0.001$. The vertical segments in the box plots show the first quartile, median, and third quartile. The whiskers on both ends represents the maximum and minimum for each dataset analyzed.

© The Author(s)
